# Single-cell RNA sequencing and traditional RNA sequencing reveals the role of cancer-associated fibroblasts in oral squamous cell carcinoma cohort

**DOI:** 10.3389/fonc.2023.1195520

**Published:** 2023-05-10

**Authors:** Lin Wu, Jun Yang, Peng She, Fanzhi Kong, Zhenwei Mao, Shengjun Wang

**Affiliations:** ^1^ Department of Stomatology, Affiliated People’s Hospital of Jiangsu University, Zhenjiang, China; ^2^ Department of Laboratory Medicine, Affiliated People’s Hospital of Jiangsu University, Zhenjiang, China

**Keywords:** cancer-associated fibroblast, single-cell RNA sequencing, periodontal disease, oral squamous cell carcinoma, lasso regression analysis

## Abstract

Chronic inflammation of the alveolar bones and connective tissues supporting teeth causes periodontal disease, one of the most prevalent infectious diseases in humans. It was previously reported that oral cancer was the sixth most common cancer in the world, followed by squamous cell carcinoma. Periodontal disease has been linked to an increased risk for oral cancer in some studies, and these studies have found a positive relationship between oral cancer and periodontal disease. In this work, we aimed to explore the potential correlation between oral squamous cell carcinoma (OSCC) and Periodontal disease. The single-cell RNA sequence analysis was applied to explore the genes that were closely associated with cancer-associated fibroblasts (CAFs). the head and neck squamous cell carcinoma. The Single sample Gene Set Enrichment Analysis (ssGSEA) algorithm was applied to explore the scores of CAFs. Subsequently, the differentially expressed analysis was applied to explore the CAFs-related genes that play a key role in the OSCC cohort. The LASSO regression analysis and the COX regression analysis were applied to construct the CAFs-based periodontal disease-related risk model. In addition, the correlation analysis was used to explore the correlation between the risk model and clinical features, immune-related cells, and immune-related genes. By using the single-cell RNA sequence analysis, we successfully obtained the biomarkers for the CAFs. Finally, we successfully obtained a six-CAFs-related genes risk model. The ROC curve and survival analysis revealed that the risk model showed good predictive value in OSCC patients. Our analysis successfully provided a new direction for the treatment and prognosis of OSCC patients.

## Introduction

Among the most prevalent infectious diseases affecting humans is periodontal disease, which results in the destruction of the alveolar bones and connective tissues supporting teeth due to chronic inflammation (1). As a result of the diverse microbiota associated with periodontal biofilms, pathological inflammation occurs. There is a growing understanding of the pathophysiology of periodontal disease, based on the identification of key bacteria in biofilms, host immune responses, and environmental factors (2). Oral health examinations conducted by the National Health Survey show a high prevalence of periodontitis in the United States. It is estimated that 47% of adults over the age of 30 have periodontitis, and the number of adults over the age of 65 has increased to 70% (3). Smokers are more likely to suffer from severe periodontal disease, bone attachment and tooth loss, gingival recession, and periodontal pockets compared with nonsmokers (4). It was also highlighted that passive smoking could lead to disease and death in non-smokers because studies have shown that passive smoking can result in disease and death (5).

Globally, oral cancer kills 2.7 people per 100,000 in 2012. An estimated 300,378 new cases are reported each year (6). In recent years, young and middle-aged populations have been experiencing an increase in incidence (7). Previously, it was reported that oral cancer was the sixth most common cancer in the world, and squamous cell carcinoma was the most common form. It is thought that oral cancer is a multifactorial disease, with tobacco, alcohol, and betel nut being the main risk factors (8). There is a significant epidemiological correlation between smoking and oral cancer, which plays a crucial role in its development and occurrence (9). The risk of developing oral cancer in smokers is 7 to 10 times higher than that of non-smokers, and the risk of developing second primary cancers is 3 times higher than that of non-smokers (10). The presence of the periodontal disease may be an independent risk factor for oral cancer, according to some studies that have found a positive association between periodontal disease and oral cancer (11).

The function of fibroblasts is critical to disease progression, tissue homeostasis, cancer progression, inflammatory and fibrotic conditions, and wound healing (12). The complex nature of cancer-associated fibroblasts (CAFs) could provide prognostic and therapeutic information, which could help stratify and tailor therapy (13). Among the main components of the stroma are CAFs, which secrete growth factors and extracellular matrix proteins that promote the proliferation of tumors, resistance to therapy, and immune rejection (14). These reasons have historically led to CAFs being viewed as tumor-promoting agents. As a result of the new understanding of CAF heterogeneity in solid tumors, findings in one cancer type may have a broader impact on the field of oncology (15). As is reported by a previous study, through stromal lncRNA-CAF/interleukin-33 signaling, fibroblasts are reprogrammed to promote oral squamous cell carcinoma growth (16).

In the latest research, tobacco has been shown to significantly increase the prevalence of oral cancer and aggravate the damage caused by periodontal disease. The prevalence of oral cancer may be linked to periodontal disease, but there is limited evidence for this association. It is essential to investigate further the mechanisms by which tobacco contributes to periodontal disease exacerbation and progression, as well as the specific molecular mechanisms of periodontal disease and oral cancer. Therefore, in this work, we aim to explore the potential correlation between oral cancer and periodontal disease. In addition, we also explore the potential relationship between CAF and oral cancer.

## Methods

### The dataset downloaded

In this study, oral cancer RNA-seq data and corresponding clinical information were obtained from The Cancer Genome Atlas (TCGA) dataset (https://portal.gdc.com). A Limma package of R software was used to study mRNA differential expression. A scRNA-seq database focused on tumor microenvironment is called Tumor Immune Single-cell Hub 2 (TISCH2). With TISCH2, you can explore TME across different cancer types by annotating every single cell type at the single-cell level. The Chemical-Gene-Protein Database (CTD, http://ctdbase.org/) is a robust database that provides human-curated information on chemical-gene/protein interactions, chemical diseases, and gene diseases.

### Pathways enrichment analysis

In order to further confirm the potential functions of the potential targets, functional enrichment was used to analyze the data. Annotating genes with functions using Gene Ontology (GO) is a widely used method, especially for molecular functions (MF), biological pathways (BP), and components of cells (CC). Gene function and high-level genome function information can be analyzed by KEGG enrichment analysis. An analysis of GO functions and enrichment of KEGG pathways of potential mRNAs was done using the ClusterProfiler package in R.

### The single sample gene set enrichment analysis algorithm evaluates the CAF-related scores in the oral squamous cell carcinoma cohort

In this study, tumor cases were scored using the ssGSEA method, which calculates overexpression measures based on a rank-based method. Various pathways-related signatures have been used in previous studies to calculate ssGSEA scores.

### The differentially expressed analysis

In order to obtain RNAseq data and corresponding clinical information for OSCC, we consulted the TCGA database (https://portal.gdc.com). A TCGA correction was applied to correct for false positive results when analyzing the differential expression of mRNA using the Limma package of R software. The mRNA differential expression was defined by “adjusted P value < 0.05 and log_2_ (fold change) > 0.5 or log_2_ (fold change) < -0.5” as the threshold.

### The immune cell infiltration analysis

In R, various algorithms were applied to RNA-seq data of different subgroups of oral squamous cell carcinoma patients. A Spearman correlation analysis was performed on gene expression and expression to estimate the relative proportion of multiple immune infiltrating cells. In addition, statistical significance requires P < 0.05.

### Construction of the prognostic prediction model in the oral squamous cell carcinoma cohort

The gene expression data and the clinical-related data were obtained from the TCGA database. Subsequently, in order to explore the genes that are closely associated with the prognosis of oral squamous cell carcinoma patients, the Lasso regression analysis, and univariate and multivariate COX regression analysis were applied to construct the prognostic prediction model in oral squamous cell carcinoma (OSCC) cohort.

### Gene set variation analysis of key genes

In order to evaluate the gene set enrichment, GSVA scores gene sets associated with individual genes. This analysis interprets gene-level changes as pathway-level changes, then assesses the sample’s biological function using the molecular signature as a database. Based on the GSVA algorithm, a comprehensive assessment of possible biological function changes was conducted.

### Cell culture

In our laboratory, human oral gingival epithelial cell line HOEC and OSCC cell line SCC-4 has previously been stored. Penicillin/streptomycin and 10% fetal bovine serum were added to the cell lines during incubation at 37°C and 5% CO_2_.

### Quantitative real-time PCR

HOEC cells and SCC-4 cells were gathered and total RNA was isolated using TRIzol reagent. To synthesize cDNA, RNA (as a template) was used along with the PrimeScriptTM RT Kit (TaKaRa). Once reverse transcription was completed, PCR amplification was carried out with SYBR Green qPCR Master Mix. The experimental data was calculated using the 2 ^−△△CT^ method, and GAPDH was used as the internal control. The primers for the genes are as follows: IL-10, Forward Primer, 5’-GACTTTAAGGGTTACCTGGGTTG-3’, Reverse Primer, 5’-TCACATGCGCCTTGATGTCTG-3’.

### Statical analysis

To assess the prognostic significance of multiple clinicopathological features associated with OSCC, data were analyzed using the R package and ROC curve analysis. Kaplan-Meier survival analysis was used to assess the survival benefit of clinical features by using Cox proportional hazards regression, and Cox proportional hazards regression was used to assess their independent prognostic value for OS. The significance level was set at P value < 0.05.

## Results

### The single-cell RNA sequencing analysis revealed the genes that are closely associated with the CAFs

**Figure 1:**
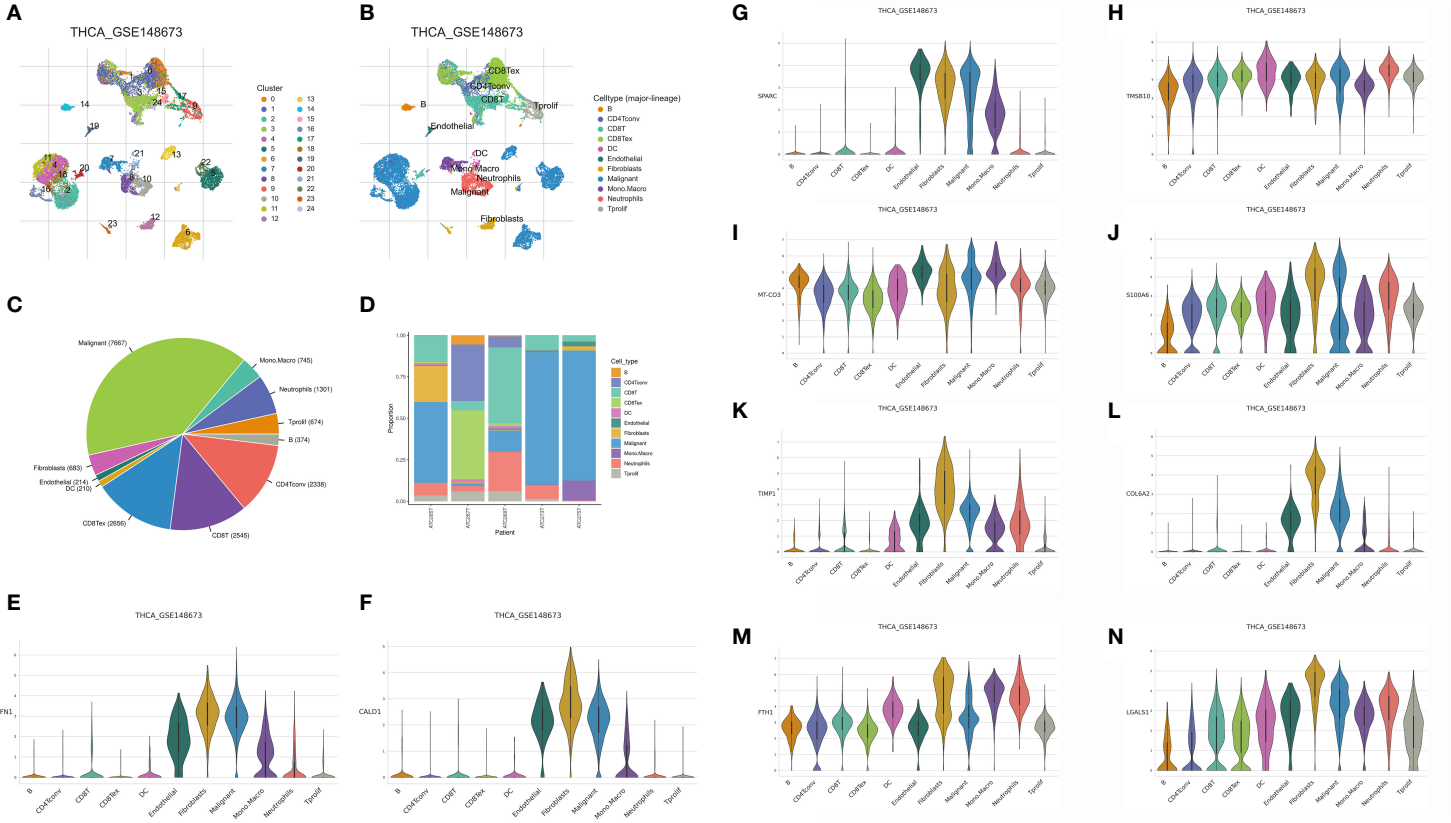
**(A, B)** The single-cell demonstrated the different cell types in the GSE148673; **(C, D)** The cell proportion of different cell types in the GSE148673; The expression level of FN1 **(E)**, CALD1 **(F)**, SPARC **(G)**, TMSB10 **(H)**, MT-CO3 **(I)**, S100A6 **(J)**, TIMP **(K)**, COL6A2 **(L)**, FTH1 **(M)** and LGALS1 **(N)** in the GSE148673.

With TISCH2, we successfully obtained the genes that are closely associated with the CAFs in head and neck squamous cell carcinoma samples. In this work, the GSE148673 was taken into analysis. After the cell clustering analysis, a total of 11 cell types were involved in the analysis, which includes B cells, CD4Tconv cells, CD8T cells, CD8tex cells, DC, endothelial cells, fibroblasts, malignant cells, mono and macro cells, neutrophils and Tprolif (**Figures 1A, B**). In addition, we also evaluate the proportion of different cells in the different samples (**Figures 1C, D**). Finally, a total of ten genes were considered to be closely associated with the fibroblasts, such as FN1, CALD1, SPARC, TMSB10, MT-CO3, S100A6, TIMP1, COL6A2, FTH1 and LGALS1 (**Figures 1E–N**).

### The ssGSEA was applied to evaluate the scores of multiple immune-related indexes and fibroblasts in the OSCC cohort

**Figure 2:**
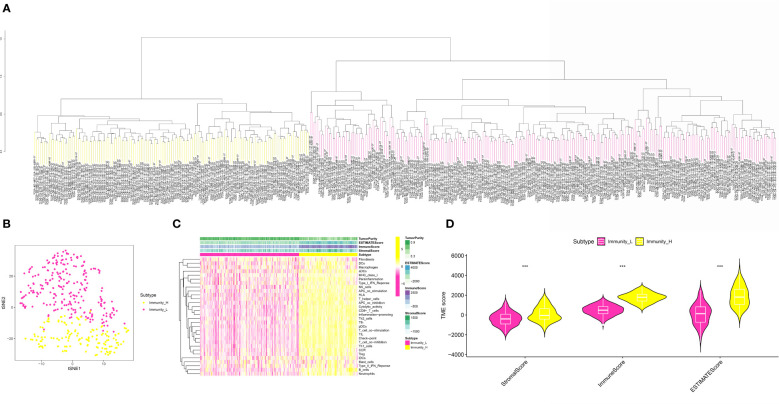
**(A)** The ssGSEA analysis was applied to evaluate the immune-related score and the fibroblasts-related score in OSCC cohort; **(B)** The OSCC cohort was divided into low- and high-immune related groups; **(C)** The heatmap demonstrated the immune-related score and the fibroblasts-related score in OSCC cohort; **(D)** The correlation analysis between low- and high-risk groups in OSCC cohort. ***P < 0.001.

In order to obtain the scores of multiple immune-related indexes and fibroblasts, we then performed the ssGSEA (**Figure 2A**). The heatmap demonstrated that the OSCC cohort was divided into immune-H and immune-L groups based on the multiple immune-related indexes, such as aDCs, APC co-inhibition, APC co-stimulation, B cells, CCR, CD8+ T cells, Check-point, Cytolytic activity, DCs, HLA, iDCs, Inflammation promoting, Macrophages, Mast cells, MHC class I, Neutrophils, NK cells, Parainflammation, pDCs, T cell co-inhibition, T cell co-stimulation, T helper cells, Tfh, Th1 cells, Th2 cells, TIL, Treg, Type I IFN Response, Type II IFN Response and Fibroblasts (**Figure 2C**). In addition, OSCC patients with low immune-related scores are also associated with low fibroblast-related scores (**Figure 2D**). On the basis of the immune-related scores, the OSCC cohort was successfully divided into immune-low and immune-high groups (**Figure 2B**).

### Evaluation of the correlation between immune-related scores and tumor mutational burden, human leukocyte antigen and immune checkpoint therapy

**Figure 3:**
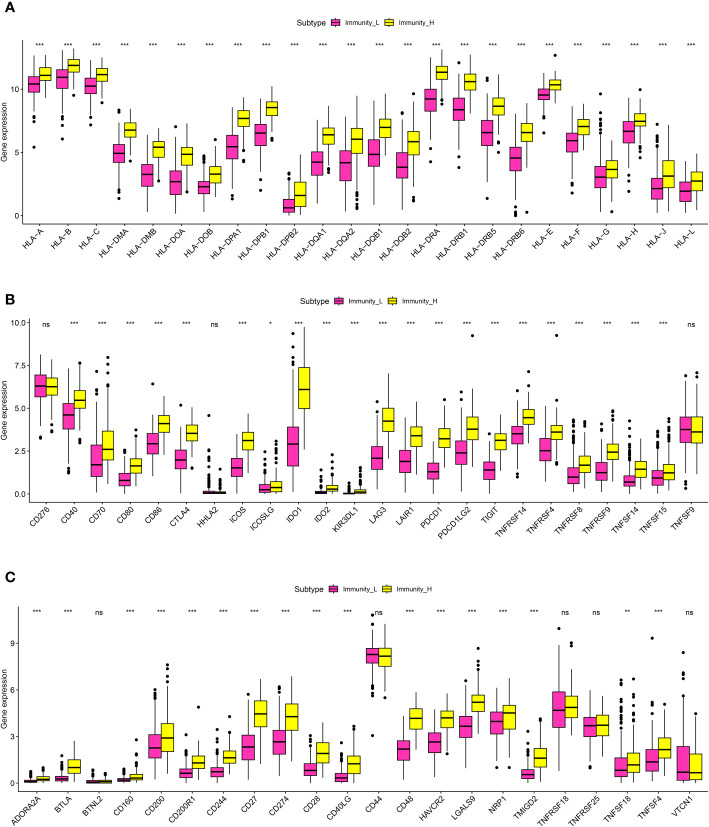
**(A)** The correlation analysis between the expression level of HLA-related genes and the risk scores; **(B, C)** The correlation analysis between the expression level of immune checkpoint-related genes and risk scores. *P < 0.05; **P < 0.01; ***P < 0.001. ns, results were not statistically significant.

In order to further explore the association between immune-related scores and some immune-related indexes, we then performed the correlation analysis. For TME, the results demonstrated that the higher immune-related scores were also correlated with the higher stromal score, immune score, and estimate score. In terms of the HLA-related genes, the correlation analysis suggested that the higher immune-related score was highly correlated with the higher expression level of HLA-related genes, such as HLA-A, HLA-B, HLA-C, HLA-DMA and et al. (**Figure 3A**). Finally, in order to explore the role of immune-related scores in immune checkpoint therapy, we then performed the correlation analysis. The results demonstrated that the expression level of immune checkpoint-related genes was significantly associated with the immune-related scores. The results demonstrated that the immune-related scores may also be able to guide immune checkpoint-related therapy (**Figures 3B, C**).

### Explore the genes that are closely associated with the periodontal disease and CAFs

**Figure 4:**
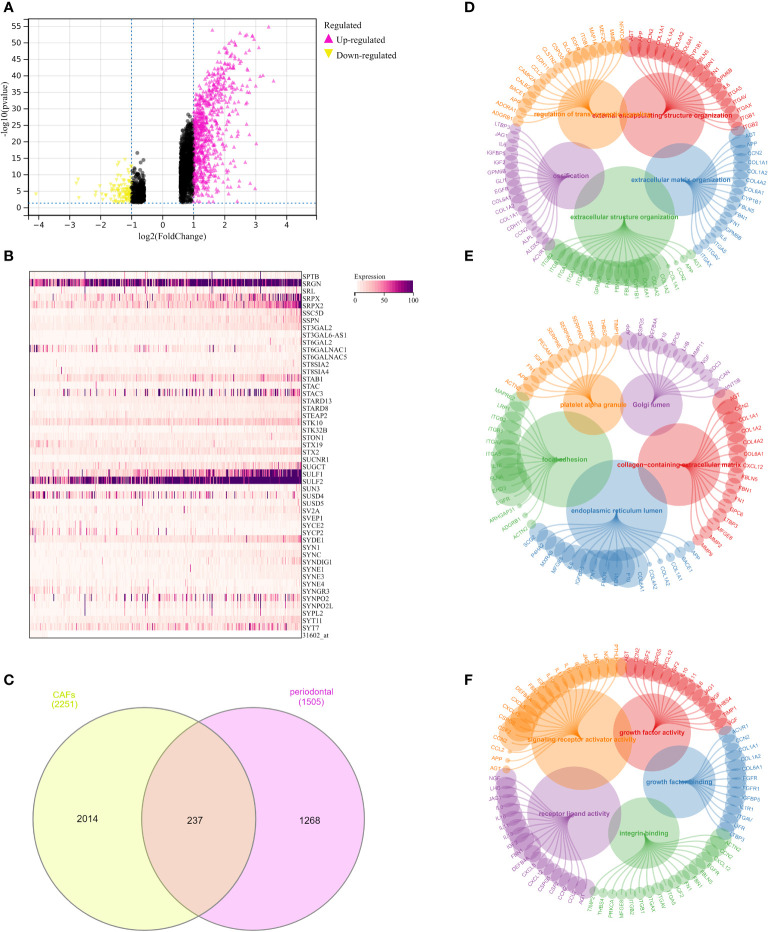
**(A)** The differentially expressed analysis between low- and high-fibroblasts groups; **(B)** The heatmap demonstrated 50 key genes in OSCC cohort; **(C)** The Venn diagram demonstrated the genes that were associated with the CAFs and periodontal disease; **(D)** The GO BP enrichment analysis; **(E)** The GO CC enrichment analysis; **(F)** The GO MF enrichment analysis.

In order to further explore the genes that were closely correlated with the CAFs, we then performed the differentially expressed analysis between CAFs-low and CAFs-high groups. The results demonstrated that a total of 2251 genes were regarded as differentially expressed genes. Among them, 1759 of them were considered up-regulated genes. In addition, 492 of the were regarded as down-regulated genes (**Figure 4A**). The heatmap demonstrated 50 of the differentially expressed genes in the OSCC cohort (**Figure 4B**). Subsequently, we obtained the periodontal disease-related genes from the CTD database. Genes with more the 15 inference scores were taken into consideration. We finally obtained 1505 periodontal disease-related genes. The Venn diagram demonstrated that a total of 237 genes were closely associated with periodontal disease and CAFs (**Figure 4C**).

### Exploration of the potential pathways that were closely associated with the key genes linked with CAFs and periodontal disease

Further, we performed the GO enrichment analysis, the results demonstrated many pathways were significantly enriched. For GO BP, the results demonstrated that extracellular matrix organization, extracellular structure organization, external encapsulating structure organization, ossification, and regulation of trans-synaptic signaling were the most enriched pathways (**Figure 4D**). In terms of the GO CC, the results demonstrated that collagen-containing extracellular matrix, endoplasmic reticulum lumen, platelet alpha granule, focal adhesion, and Golgi lumen were the most enriched pathways (**Figure 4E**). In addition, the GO MF enrichment analysis demonstrated that integrin binding, signaling receptor activator activity, receptor-ligand activity, growth factor activity, and growth factor binding were the most enriched pathways (**Figure 4F**).

### The construction of the risk model based on the CAFs and periodontal disease in the OSCC cohort

**Figure 5:**
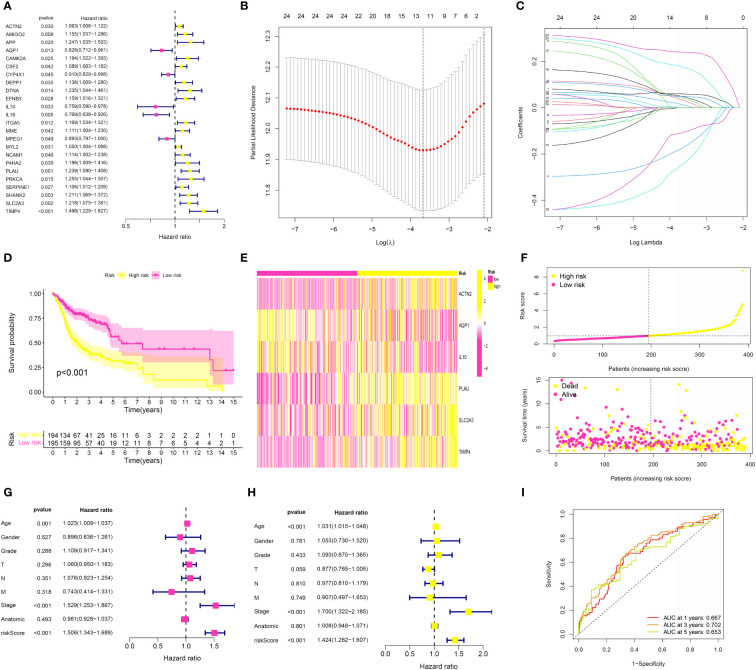
**(A)** The univariate COX regression analysis was applied to evaluate the prognosis-related genes; **(B, C)** The LASSO regression analysis was further applied to evaluate the prognosis-related genes; **(D)** The survival analysis was performed between low- and high-risk groups; **(E)** The heatmap of 6 genes involved in risk model in OSCC cohort; **(F)** The risk plot of 6 genes involved in risk model in OSCC cohort; **(G)** The univariate independent prognosis analysis based on the risk score and clinical features; **(H)** The multivariate independent prognosis analysis based on the risk score and clinical features; **(I)** The ROC curve demonstrated the predictive value in OSCC cohort.

Based on the above analysis, we successfully obtained the genes that were closely associated with CAFs and periodontal disease. In order to further explore the genes that were closely correlated with the prognosis in OSCC patients, we first performed the univariate COX regression analysis, the results demonstrated that a total of 24 CAFs and periodontal disease-related genes were closely associated with the prognosis of OSCC patients (**Figure 5A**). Then, we performed the LASSO regression analysis (**Figures 5B, C**) and the multivariate COX regression analysis. The results demonstrated that ACTN2, AQP1, IL10, PLAU, SLC2A3 and TIMP4 were the key prognostic factors in the OSCC cohort, which were involved in the risk model. In addition, each OSCC patient was assigned the risk score as follows: Risk score = ACTN2 * 0.0893092372500448 + AQP1 * -0.288187341626548 + IL10 * -0.428014707494522 + PLAU * 0.137656068510232 + SLC2A3 * 0.248403275463445 + TIMP4 * 0.321091675329555. The heatmap demonstrated the expression level of 6 key genes in the OSCC cohort. In addition, the risk plots revealed that the OSCC patients were averagely divided into low- and high-risk groups based on the median risk score (**Figures 5E, F**). The survival analysis suggested that OSCC patients with higher risk scores were correlated with lower overall survival (OS) (**Figure 5D**). Subsequently, we also performed the univariate and multivariate independent prognostic analysis. For univariate independent analysis, the age, stage, and risk score were the independent risk factors in the OSCC cohort (**Figure 5G**). In addition, for multivariate independent prognostic analysis, the results demonstrated that age, stage, and risk score were the independent risk factors in the OSCC cohort (**Figure 5H**). Finally, the time-dependent ROC curve revealed that the risk model showed good predictive value in the OSCC cohort (**Figure 5I**).

### Identification of the immune cell and immune checkpoint-related genes in the risk model

**Figure 6:**
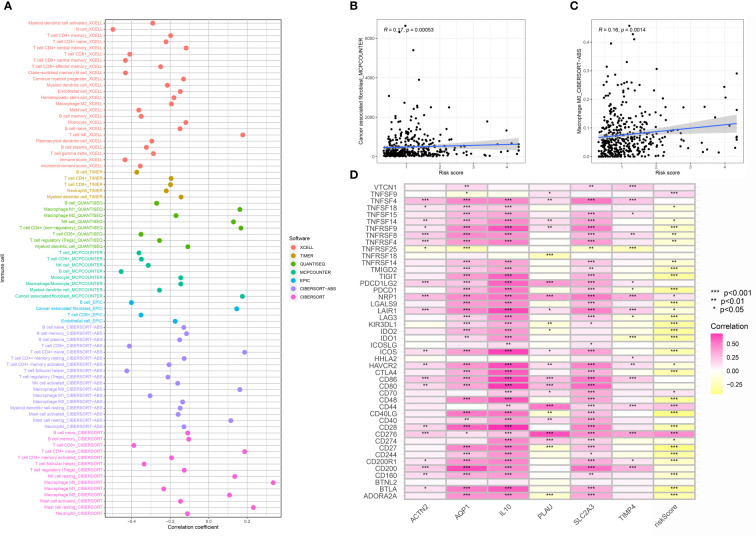
**(A)** The immune cell infiltration analysis; **(B)** The correlation analysis between risk score and cancer-associated fibroblasts; **(C)** The correlation analysis between risk score and M0 macrophages; **(D)** The correlation analysis between risk score and immune checkpoint-related genes.

Furth, we performed the immune cell infiltration analysis in the OSCC cohort to evaluate the correlation between immune-related cells and the risk score. The results demonstrated that multiple immune-related cells were closely correlated with the CAFs-based periodontal disease-related risk model, such as NK T cell, M1 macrophage, M0 macrophage, M2 macrophage cancer-associated fibroblasts and mast cell (**Figures 6A–C**). Additionally, we also evaluate the correlation between risk score and the immune checkpoint-related genes. The results demonstrated that the expression level of many immune checkpoint-related genes was also closely related to the risk scores, which may be able to guide immune checkpoint-related therapy (**Figure 6D**).

### Construction of the risk models-based nomogram and evaluation of the correlation between risk score and clinical features

**Figure 7:**
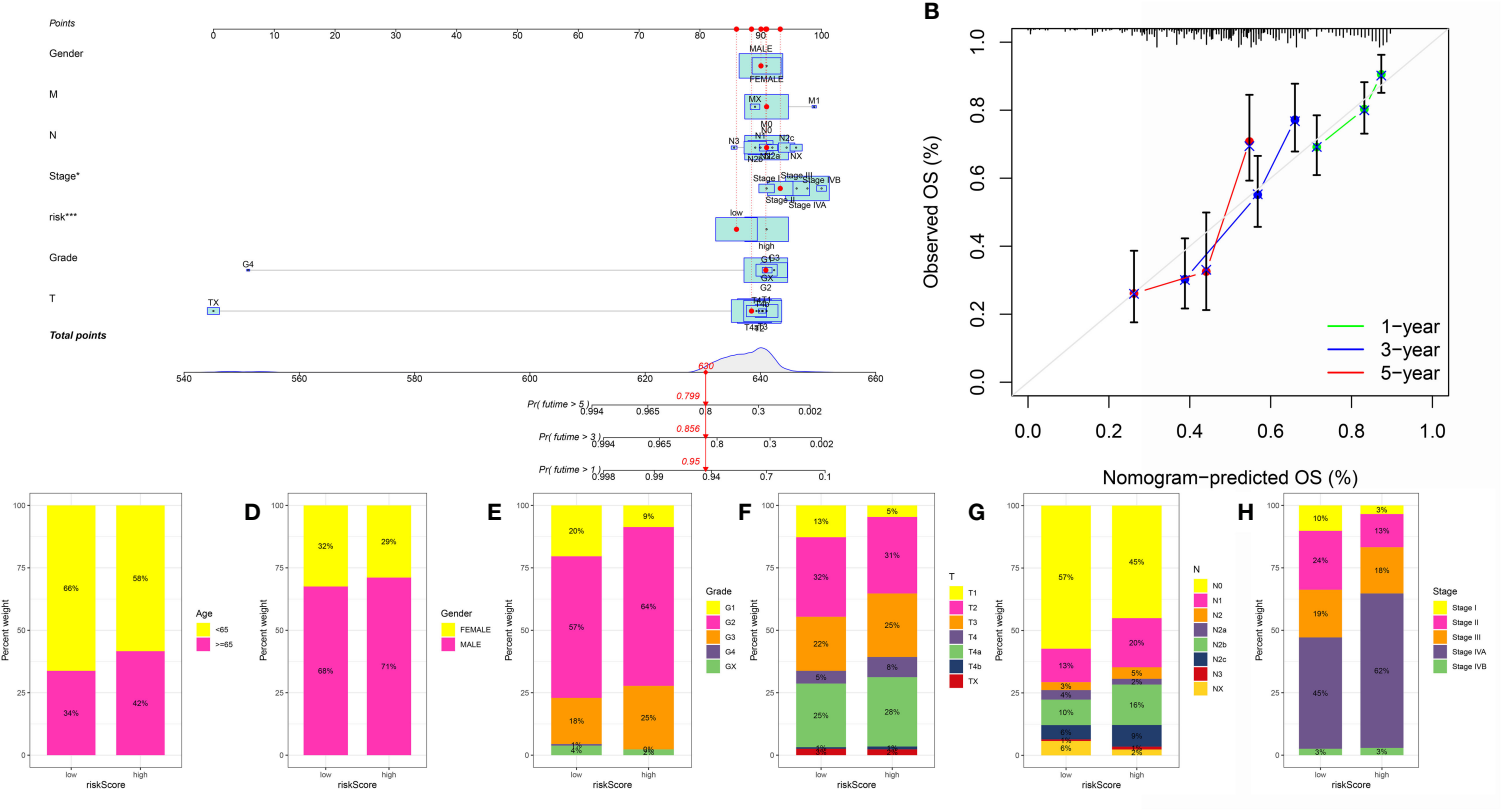
**(A)** The nomogram was constructed based on the clinical features and risk score; **(B)** The calibration curve was applied to evaluate the predictive value in OSCC cohort; The correlation between risk score and age **(C)**, gender **(D)**, grade **(E)**, T stage **(F)**, N stage **(G)** and stage **(H)**.

In order to obtain a better model in the OSCC cohort, we then constructed the nomogram based on the risk score and the clinical features (**Figure 7A**). The calibration curve demonstrated that the nomogram can be regarded as a good model for the prediction of OSCC patients (**Figure 7B**). Subsequently, we performed the correlation analysis. The results demonstrated that the high-risk score was correlated with the higher age, grade, stage, T stage, and N stage. In addition, the patients involved in the high-risk group were more likely to be associated with male OSCC patients (**Figures 7C–H**).

### Exploration of the pathways, expression level, and OS of genes involved in the risk model

**Figure 8:**
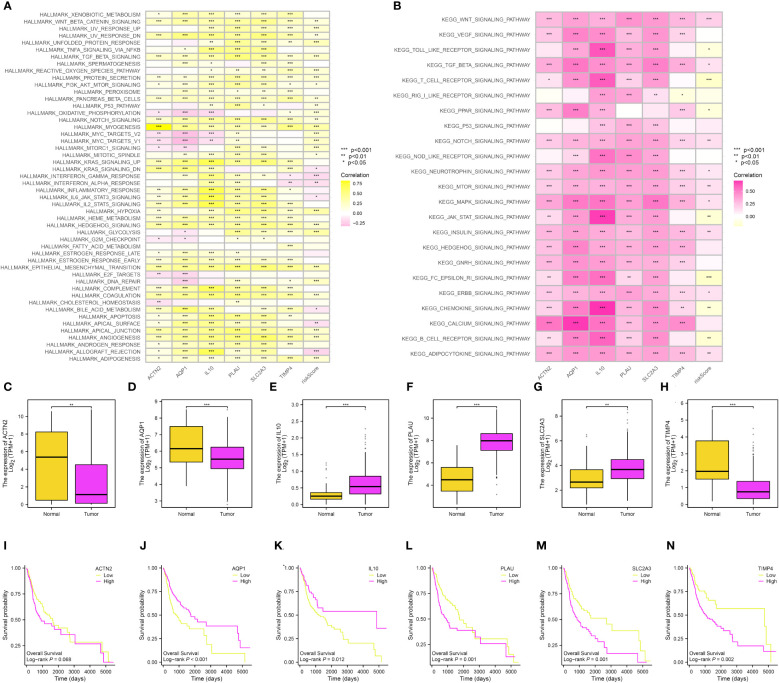
**(A)** The GSVA based on the Hallmark term; **(B)** The GSVA based on the KEGG term; The expression level of ACTN2 **(C)**, AQP1 **(D)**, IL10 **(E)**, PLAU **(F)**, SLC2A3 **(G)** and TIMP4 **(H)**; The survival analysis between low- and high-ACTN2 **(I)**, AQP1 **(J)**, IL10 **(K)**, PLAU **(L)**, SLC2A3 **(M)** and TIMP4 **(N)** groups.

Next, in order to further explore the role of genes involved in the risk model, we first performed the GSVA by the terms of KEGG and Hallmark. For the Hallmark term, the results demonstrated that wnt beta-catenin signaling, unfolded protein response, TGF beta signaling, reactive oxygen species pathway, protein secretion, and coagulation were the most associated pathways (**Figure 8A**). In terms of the KEGG, the results demonstrated that the wnt signaling pathway, toll-like receptor signaling pathway, T cell receptor signaling pathway, and notch signaling pathway were closely associated with the risk score (**Figure 8B**). The expression of IL10, PLAU, and SLC2A3 was high in OSCC samples, while the expression of ACTN2, AQP1, and TIMP4 was low in OSCC samples (**Figures 8C–H**). In addition, we also performed the survival analysis of 6 key genes in the OSCC cohort. For ACTN2, PLAU, SLC2A3, and TIMP4, the high expression level of ACTN2, PLAU, SLC2A3, and TIMP4 were correlated with the poorer OS. However, the high expression level of AQP1 and IL10 were correlated with better OS (**Figures 8I–N**).

### Evaluation of the expression level of IL10 in HOEC and SCC-4 cells

**Figure 9:**
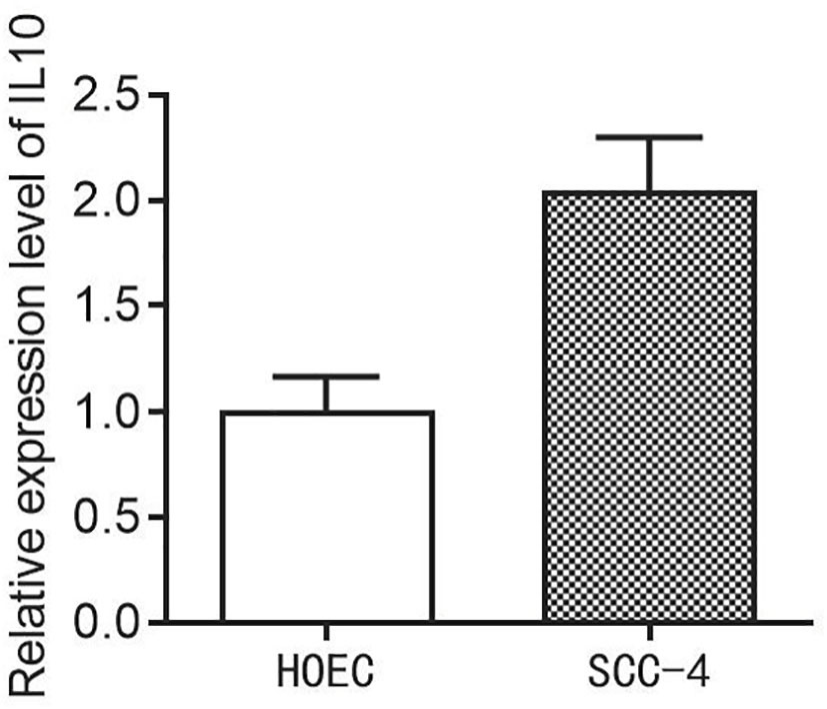
The expression level of IL10 in HOEC and SCC-4 cells.

Finally, in order to evaluate the expression level of the IL10 in HOEC and SCC-4 cells, we then performed the PCR assay. The results demonstrated that the expression of IL10 is higher in SCC-4 cells compared to the HOEC cells (**Figure 9**).

## Discussion

Genetic factors associated with periodontitis are currently being investigated in many studies. In addition to genetic risk factors, behavioral factors also play a role in determining an individual’s propensity to develop periodontitis in a complex multifactorial disease such as periodontitis (17). In addition, many studies have discovered that periodontal disease may be closely correlated with multiple cancers (18). In this work, we aim to explore the potential correlations between OSCC and periodontal disease. Firstly, we obtained the interactive genes of periodontal disease from the CTD database and the expression data of OSCC from the TCGA database. In addition, the function of fibroblasts is essential for disease progression, tissue homeostasis, cancer progression, inflammatory and fibrotic conditions, as well as wound healing. In addition to secreting growth factors and extracellular matrix proteins, CAFs promote tumor proliferation, resistance to therapies, and rejection of the immune system (19). Therefore, we then explored the role of CAFs in the OSCC cohort. The ssGSEA was applied to evaluate the score of CAFs in OSCC patients. Based on the ssGSEA, the OSCC cohort was successfully divided into different cohorts. Further analysis suggested that the immune-related scores were closely correlated with the expression level of HLA-related genes and immune checkpoint-related genes, which may be the potential biomarkers for immune-related therapy. In recent years, CAFs have been discovered to be closely associated with multiple cancers (12). In a previous study, IL32 secreted by CAF promoted breast cancer cell invasion and metastasis through integrin β3-p38 MAPK signaling (13). In addition, CAF-based risk signatures are found to be able to accurately predict hepatocellular carcinoma prognosis, and can also be used to interpret the response of hepatocellular carcinoma to immunotherapy (14). According to another study, CAF-derived lipids facilitate cancer peritoneal metastasis through the enhancement of membrane fluidity. Our study successfully revealed the potential correlation between CAFs and the OSCC (15).

In order to further explore the role of CAFs in periodontal disease and OSCC, we then performed the differentially expressed analysis between low- and high-CAF groups. And then, we successfully obtained the CAFs-related genes. Based on the periodontal disease-related genes, we finally obtained the genes that were closely associated with the CAFs and periodontal disease. Further analysis revealed that 6 genes, which include ACTN2, AQP1, IL10, PLAU, SLC2A3, and TIMP4, were the key prognosis-related genes in the OSCC cohort. In recent years, multiple analyses focused on the role of bioinformatics analysis in the diagnosis and treatment of cancer patients. In a previous study, pharmacological techniques and omics databases were used to predict the potentially harmful effects of hazardous substances The effects of dibutyl phthalate on prostate cancer are possibly androgen-independent at low concentrations, so androgen-deprivation therapy patients may be exposed to this chemical (20). In another study, an online database provided a rationale for mRNA vaccine development and suggested that KLHL14 might serve as an antigen for the development of mRNA vaccines in MALT lymphoma patients (21).

**Figure 10:**
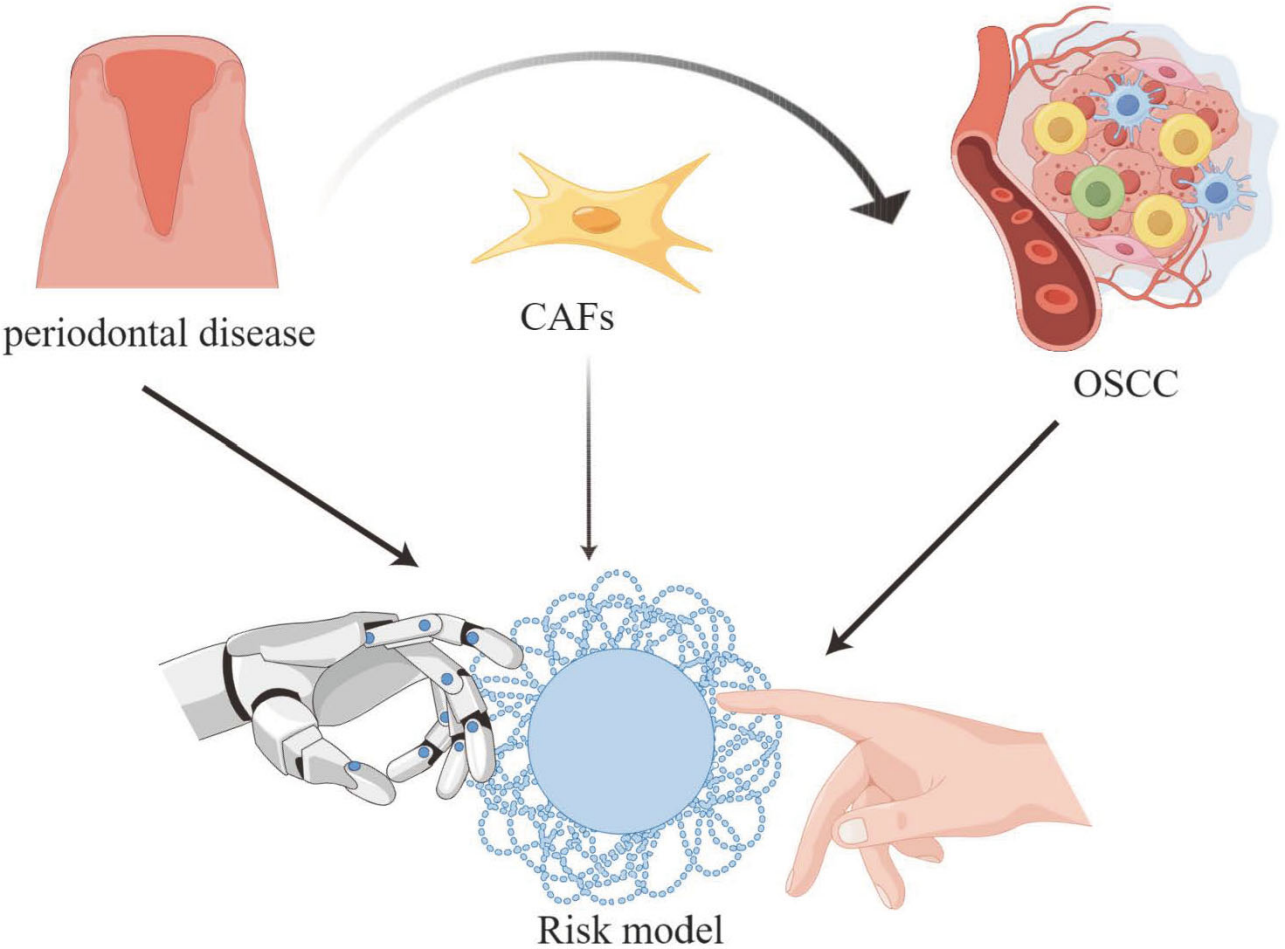
The flow chart in this work.

Next, we also evaluate the potential pathways that were closely correlated with the risk model. The results demonstrated that the wnt signaling pathway, toll-like receptor signaling pathway, T cell receptor signaling pathway, and notch signaling pathway were closely associated with the risk score. Multiple studies have discovered that these pathways may play a key role in cancers. Wnt signaling plays a critical role in developing and growing organisms. Its complexity and functional roles make it one of the most important signaling pathways (22). The enzyme plays a key role in developing new organisms during embryogenesis by promoting the differentiation, polarization, and migration of cells. Regulatory signals from Toll-like receptors are crucial for activating innate and adaptive immune responses and play an important role in colorectal cancer development (23). The benefit of targeting this pathway in cancer treatment is now well established, and several drugs have been approved, including BCG, monophosphoryl lipid A, and imiquimod (24) (**Figure 10**).

Finally, we also performed the differentially expressed analysis and the survival analysis. The results demonstrated that 6 genes involved in the risk model were closely correlated with the OS of the OSCC patients. Multiple studies have discovered that these genes were closely associated with cancers. In a previous study, ERCC1, PARP1, and AQP1 were identified as poor prognostic biomarkers for colon cancer at stages II-III (25). In addition, another study revealed that TIMP/KLF2 is a target of tumor-derived exosomal miR-3157-3p that promotes angiogenesis, vascular permeability, and metastasis in non-small cell lung cancer (26).

However, some limitations were also within in this work. Firstly, the different methods used in the bioinformatics analysis may lead to the bias (27). In addition, the application of the biomarkers in the clinical diagnosis, treatment and diagnosis requires the further validation (20, 21). Also, multiple studies are also very important to further validate the role of risk model in the OCSS cohort (28, 29).

## Data availability statement

The original contributions presented in the study are included in the article/**Supplementary Material**. Further inquiries can be directed to the corresponding authors.

## Author contributions

SW writes the manuscript. LW analyze the data. All authors contributed to the article and approved the submitted version.

## References

[1] Niemiec BA. Periodontal disease. Top Companion Anim Med (2008) 23(2):72–80. doi: 10.1053/j.tcam.2008.02.003 18482707

[2] Genco RJ Borgnakke WS. Risk factors for periodontal disease. Periodontol 2000 (2013) 62(1):59–94. doi: 10.1111/j.1600-0757.2012.00457.x 23574464

[3] Gasner NS Schure RS. Periodontal disease. In: StatPearls. Treasure Island (FL: StatPearls Publishing (2023).

[4] Passanezi E Sant’Ana ACP. Role of occlusion in periodontal disease. Periodontol 2000. (2019) 79(1):129–50. doi: 10.1111/prd.12251 30892765

[5] Sordi MB Magini RS Panahipour L Gruber R. Pyroptosis-mediated periodontal disease. Int J Mol Sci (2021) 23(1):372. doi: 10.3390/ijms23010372 35008798 PMC8745163

[6] Hajishengallis G Chavakis T. Local and systemic mechanisms linking periodontal disease and inflammatory comorbidities. Nat Rev Immunol (2021) 21(7):426–40. doi: 10.1038/s41577-020-00488-6 PMC7841384 33510490

[7] Michaud DS Fu Z Shi J Chung M. Periodontal disease, tooth loss, and cancer risk. Epidemiol Rev (2017) 39(1):49–58. doi: 10.1093/epirev/mxx006 28449041 PMC5868279

[8] Sroussi HY Epstein JB Bensadoun RJ Saunders DP Lalla RV Migliorati CA. Common oral complications of head and neck cancer radiation therapy: mucositis, infections, saliva change, fibrosis, sensory dysfunctions, dental caries, periodontal disease, and osteoradionecrosis. Cancer Med (2017) 6(12):2918–31. doi: 10.1002/cam4.1221 PMC5727249 29071801

[9] Sedghi LM Bacino M Kapila YL. Periodontal disease: the good, the bad, and the unknown. Front Cell Infect Microbiol (2021) 11:766944. doi: 10.3389/fcimb.2021.766944 34950607 PMC8688827

[10] Nwizu N Wactawski-Wende J Genco RJ. Periodontal disease and cancer: epidemiologic studies and possible mechanisms. Periodontol 2000. (2020) 83(1):213–33. doi: 10.1111/prd.12329 PMC7328760 32385885

[11] Zhang Y He J He B Huang R Li M. Effect of tobacco on periodontal disease and oral cancer. Tob Induc Dis (2019) 17:40. doi: 10.18332/tid/106187 31516483 PMC6662776

[12] Biffi G Tuveson DA. Diversity and biology of cancer-associated fibroblasts. Physiol Rev (2021) 101(1):147–76. doi: 10.1152/physrev.00048.2019 PMC7864232 32466724

[13] Chen Y McAndrews KM Kalluri R. Clinical and therapeutic relevance of cancer-associated fibroblasts. Nat Rev Clin Oncol (2021) 18(12):792–804. doi: 10.1038/s41571-021-00546-5 34489603 PMC8791784

[14] Sahai E Astsaturov I Cukierman E DeNardo DG Egeblad M Evans RM. A framework for advancing our understanding of cancer-associated fibroblasts. Nat Rev Cancer. (2020) 20(3):174–86. doi: 10.1038/s41568-019-0238-1 PMC7046529 31980749

[15] Lavie D Ben-Shmuel A Erez N Scherz-Shouval R. Cancer-associated fibroblasts in the single-cell era. Nat Cancer. (2022) 3(7):793–807. doi: 10.1038/s43018-022-00411-z 35883004 PMC7613625

[16] Ding L Ren J Zhang D Li Y Huang X Hu Q. A novel stromal lncRNA signature reprograms fibroblasts to promote the growth of oral squamous cell carcinoma via LncRNA-CAF/interleukin-33. Carcinogenesis (2018) 39(3):397–406. doi: 10.1093/carcin/bgy006 29346528

[17] Johnson TM. Smoking and periodontal disease. US Army Med Dep J (2017) 3(17):67–70. 29214622

[18] Ebersole JL Graves CL Gonzalez OA LA M PE H Hartsfield JK Jr. Aging, inflammation, immunity and periodontal disease. Periodontol 2000. (2016) 72(1):54–75. doi: 10.1111/prd.12135 27501491

[19] Wu F Yang J Liu J Wang Y Mu J Zeng Q. Signaling pathways in cancer-associated fibroblasts and targeted therapy for cancer. Signal Transduct Target Ther (2021) 6(1):218. doi: 10.1038/s41392-021-00641-0 34108441 PMC8190181

[20] Zhang T Wu J Zhang X Zhou X Wang S Wang Z. Pharmacophore based in silico study with laboratory verification-environmental explanation of prostate cancer recurrence. Environ Sci pollut Res Int (2021) 28(43):61581–91. doi: 10.1007/s11356-021-14970-8 34184217

[21] Jiang X Zhang H Ni J Zhang X Ding K. Identifying tumor antigens and immune subtypes of gastrointestinal MALT lymphoma for immunotherapy development. Front Oncol (2022) 12:1060496. doi: 10.3389/fonc.2022.1060496 36568181 PMC9772875

[22] Nusse R Clevers H. Wnt/β-catenin signaling, disease, and emerging therapeutic modalities. Cel (2017) 169(6):985–99. doi: 10.1016/j.cell.2017.05.016 28575679

[23] Keshavarz A Pourbagheri-Sigaroodi A Zafari P Bagheri N Ghaffari SH Bashash D. Toll-like receptors (TLRs) in cancer; with an extensive focus on TLR agonists and antagonists. IUBMB Life (2021) 73(1):10–25. doi: 10.1002/iub.2412 33217774

[24] Mishra V Pathak C. Human toll-like receptor 4 (hTLR4): structural and functional dynamics in cancer. Int J Biol Macromol. (2019) 122:425–51. doi: 10.1016/j.ijbiomac.2018.10.142 30365988

[25] Abdelrahman AE Ibrahim DA El-Azony A Alnagar AA Ibrahim A. ERCC1, PARP-1, and AQP1 as predictive biomarkers in colon cancer patients receiving adjuvant chemotherapy. Cancer biomark (2020) 27(2):251–64. doi: 10.3233/CBM-190994 PMC12662279 31903985

[26] Kim YS Kim SH Kang JG Ko JH. Expression level and glycan dynamics determine the net effects of TIMP-1 on cancer progression. BMB Rep (2012) 45(11):623–8. doi: 10.5483/bmbrep.2012.45.11.233 PMC4133808 23187000

[27] Ye S Liu Q Huang K Jiang X Zhang X. The comprehensive analysis based study of perfluorinated compounds-environmental explanation of bladder cancer progression. Ecotoxicol Environ Saf. (2022) 229:113059. doi: 10.1016/j.ecoenv.2021.113059 34894427

[28] Jiang X Zhang H Wang X Zhang X Ding K. Comprehensive analysis of the association between human diseases and water pollutants. Int J Environ Res Public Health (2022) 19(24):16475. doi: 10.3390/ijerph192416475 36554354 PMC9779172

[29] Hong Z Li Y Deng X Chen M Pan J Chen Z. Comprehensive analysis of triphenyl phosphate: an environmental explanation of colorectal cancer progression. Ecotoxicol Environ Saf. (2022) 241:113778. doi: 10.1016/j.ecoenv.2022.113778 36068737

