# Supplementary figures and images for: Single-cell RNA sequencing and traditional RNA sequencing reveals the role of cancer-associated fibroblasts in oral squamous cell carcinoma cohort (part 1 of 2)
Source: Front Oncol. 2023 May 10;13:1195520. doi: 10.3389/fonc.2023.1195520 (PMC10206127; doi:10.3389/fonc.2023.1195520)

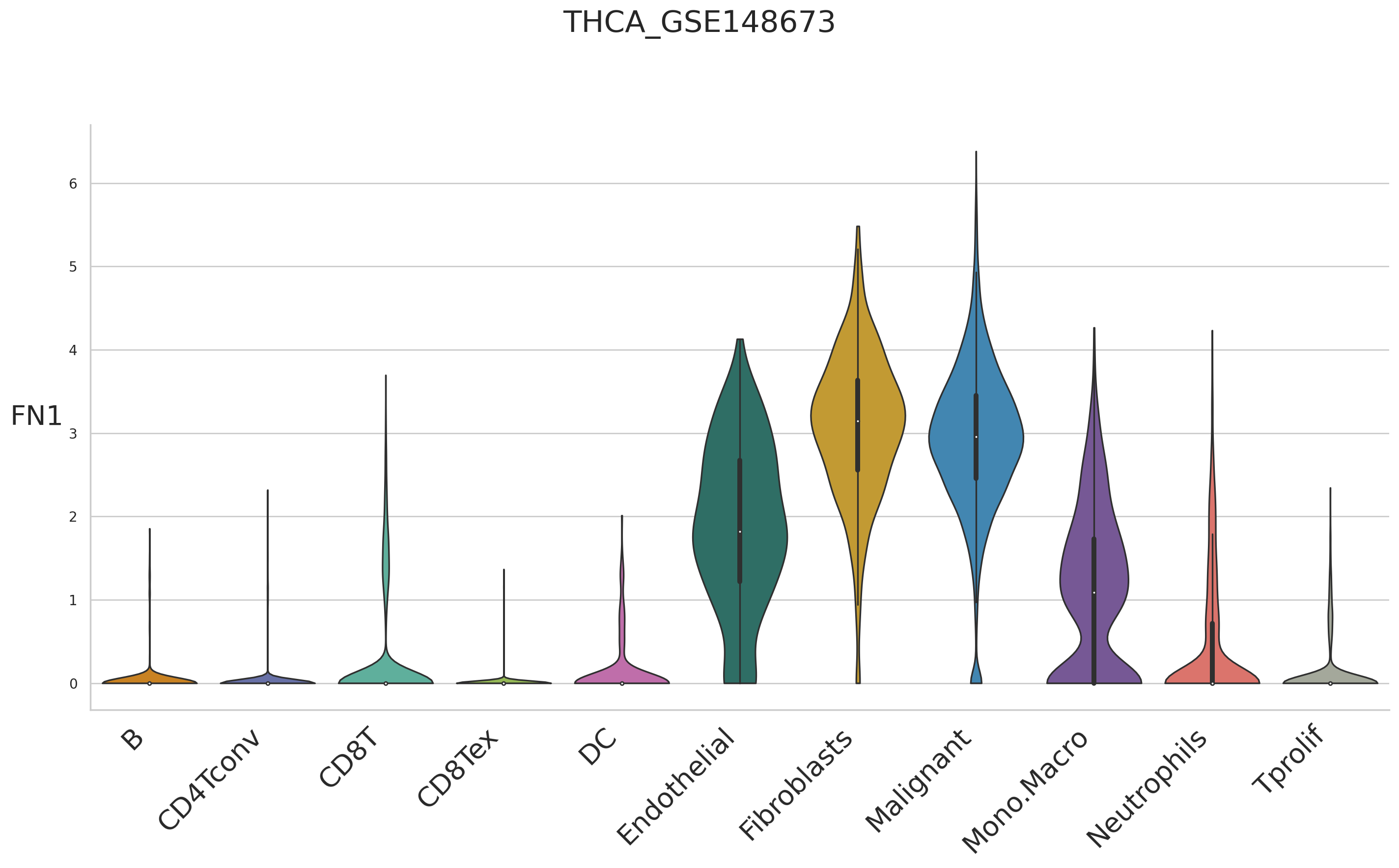

Supplement: Supplementary file 1 [file DataSheet_1.zip › bjbhzmusvx_THCA_GSE148673_violin_multiple_Celltype_curated_None.png]

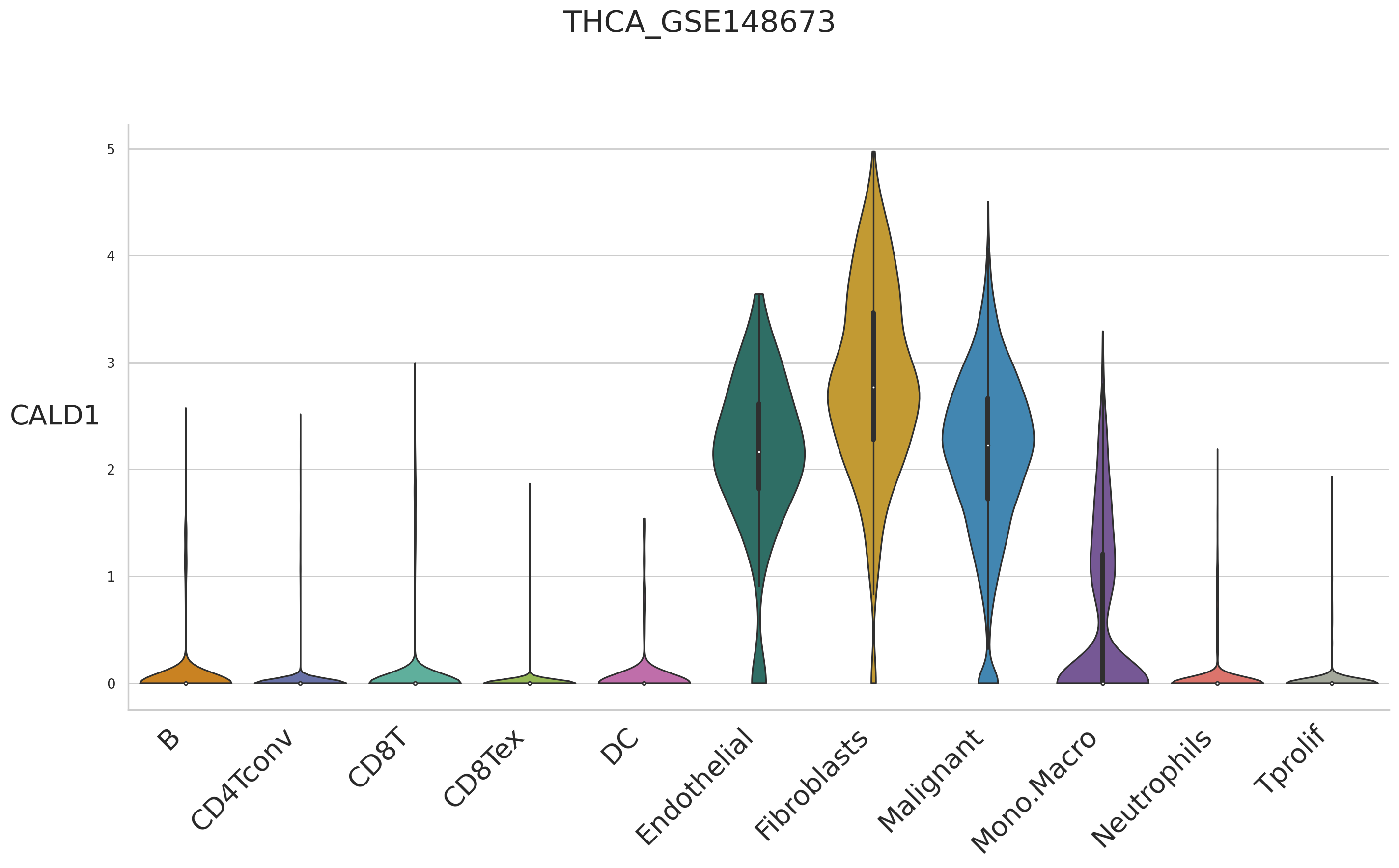

Supplement: Supplementary file 1 [file DataSheet_1.zip › dmuyoujpiw_THCA_GSE148673_violin_multiple_Celltype_curated_None.png]

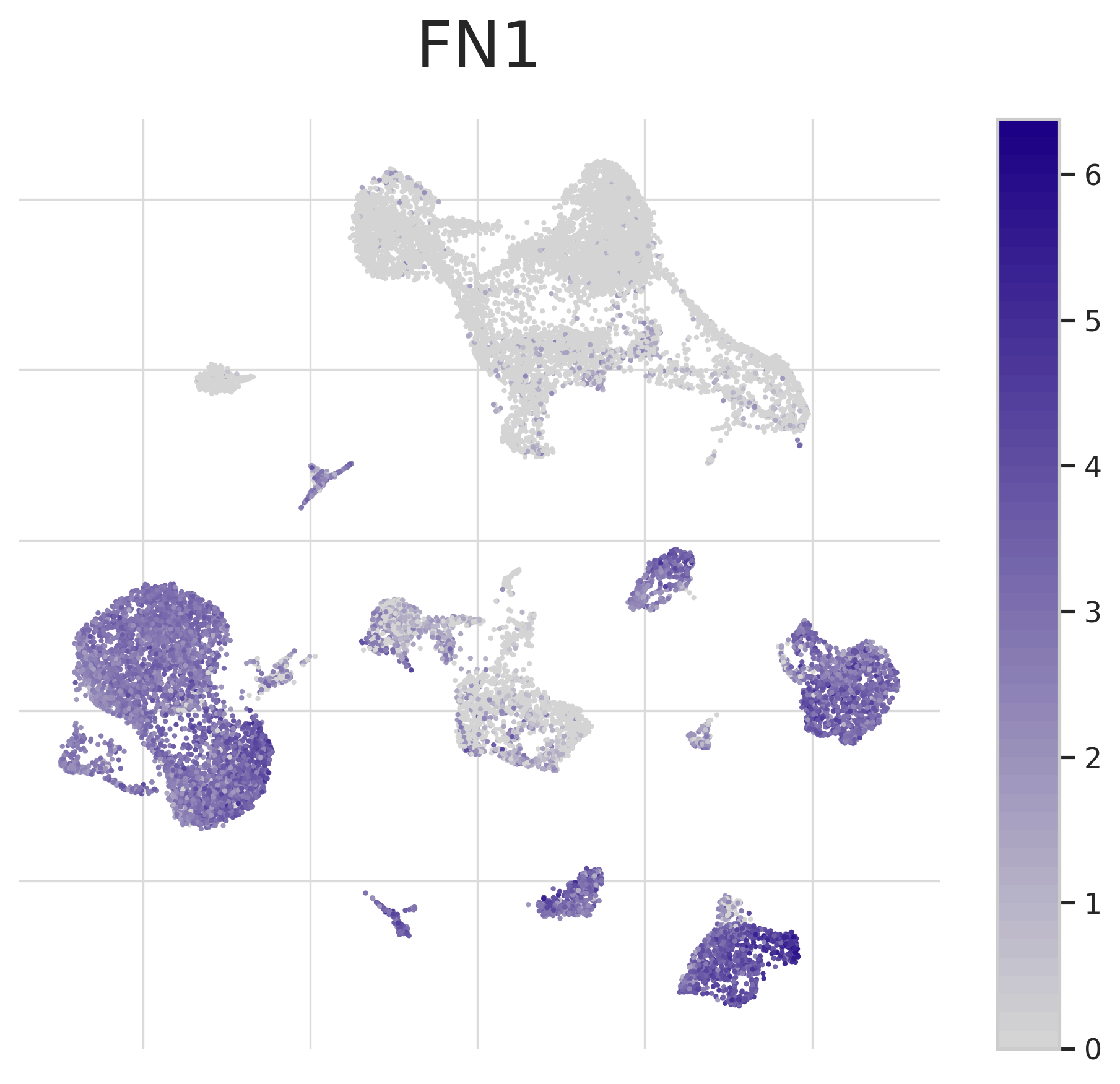

Supplement: Supplementary file 1 [file DataSheet_1.zip › epwjnqmakk_THCA_GSE148673_FN1_umap.png]

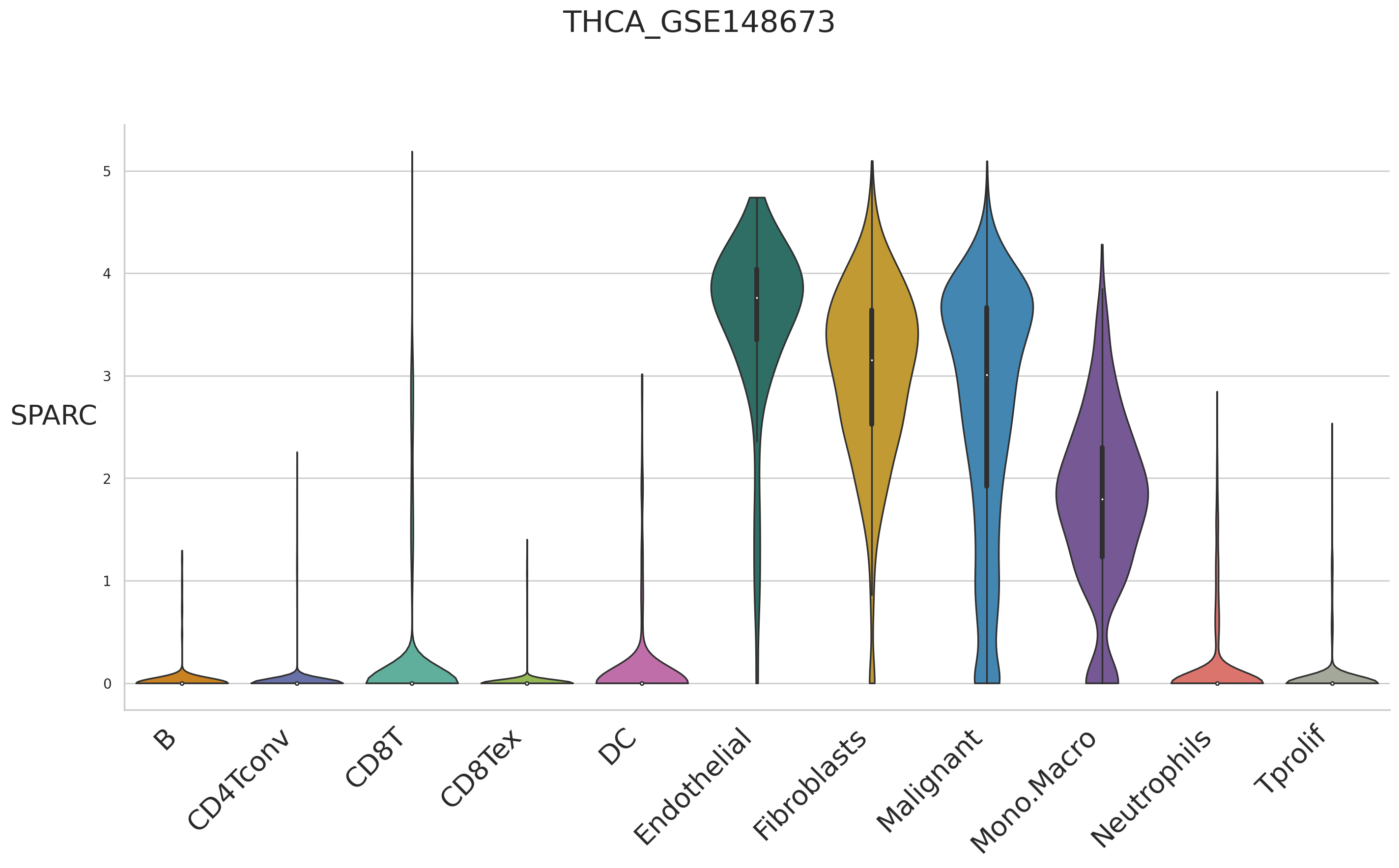

Supplement: Supplementary file 1 [file DataSheet_1.zip › evrxwlcibs_THCA_GSE148673_violin_multiple_Celltype_curated_None.png]

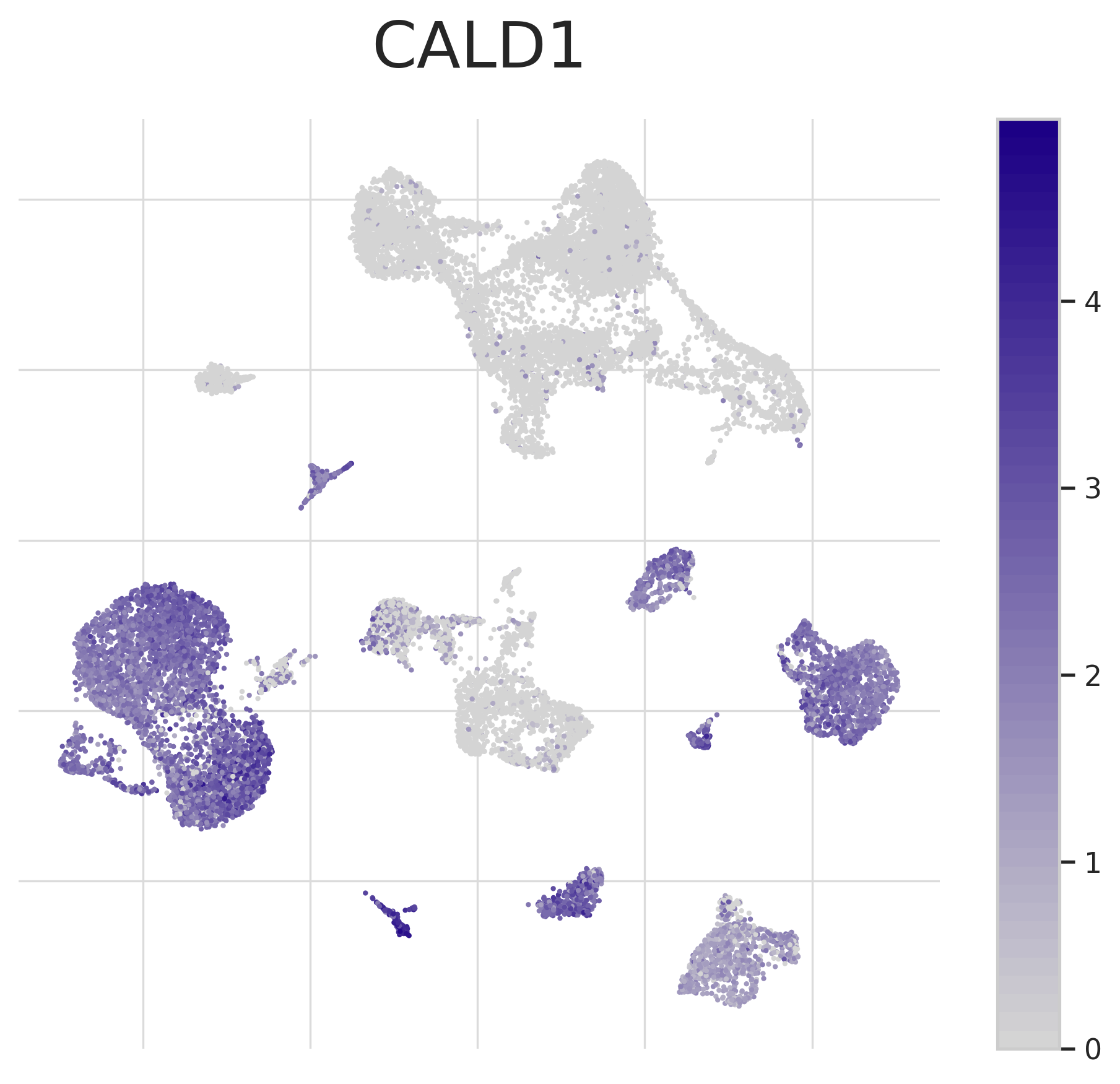

Supplement: Supplementary file 1 [file DataSheet_1.zip › flpbzphawn_THCA_GSE148673_CALD1_umap.png]

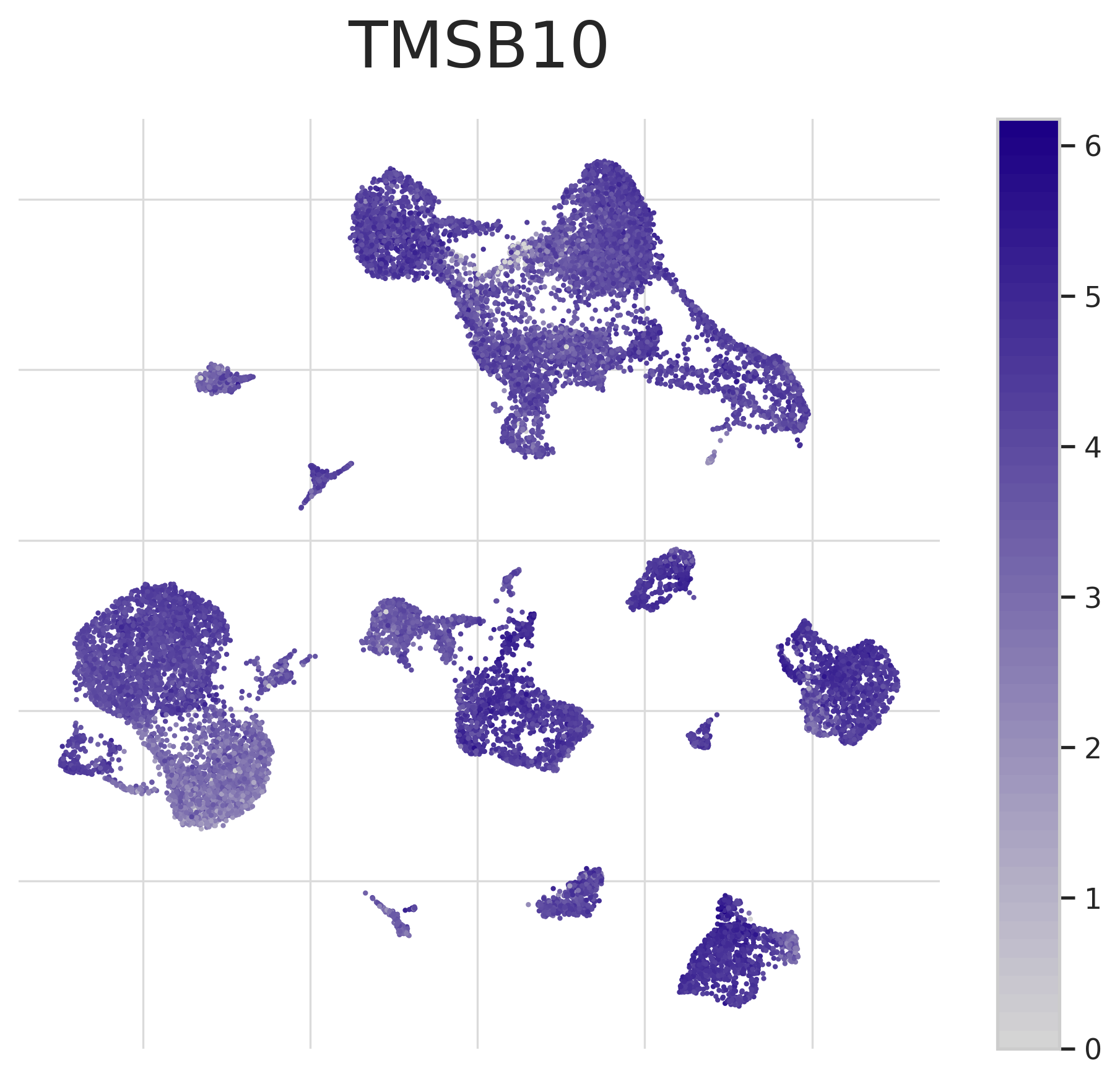

Supplement: Supplementary file 1 [file DataSheet_1.zip › gocrldjoks_THCA_GSE148673_TMSB10_umap.png]

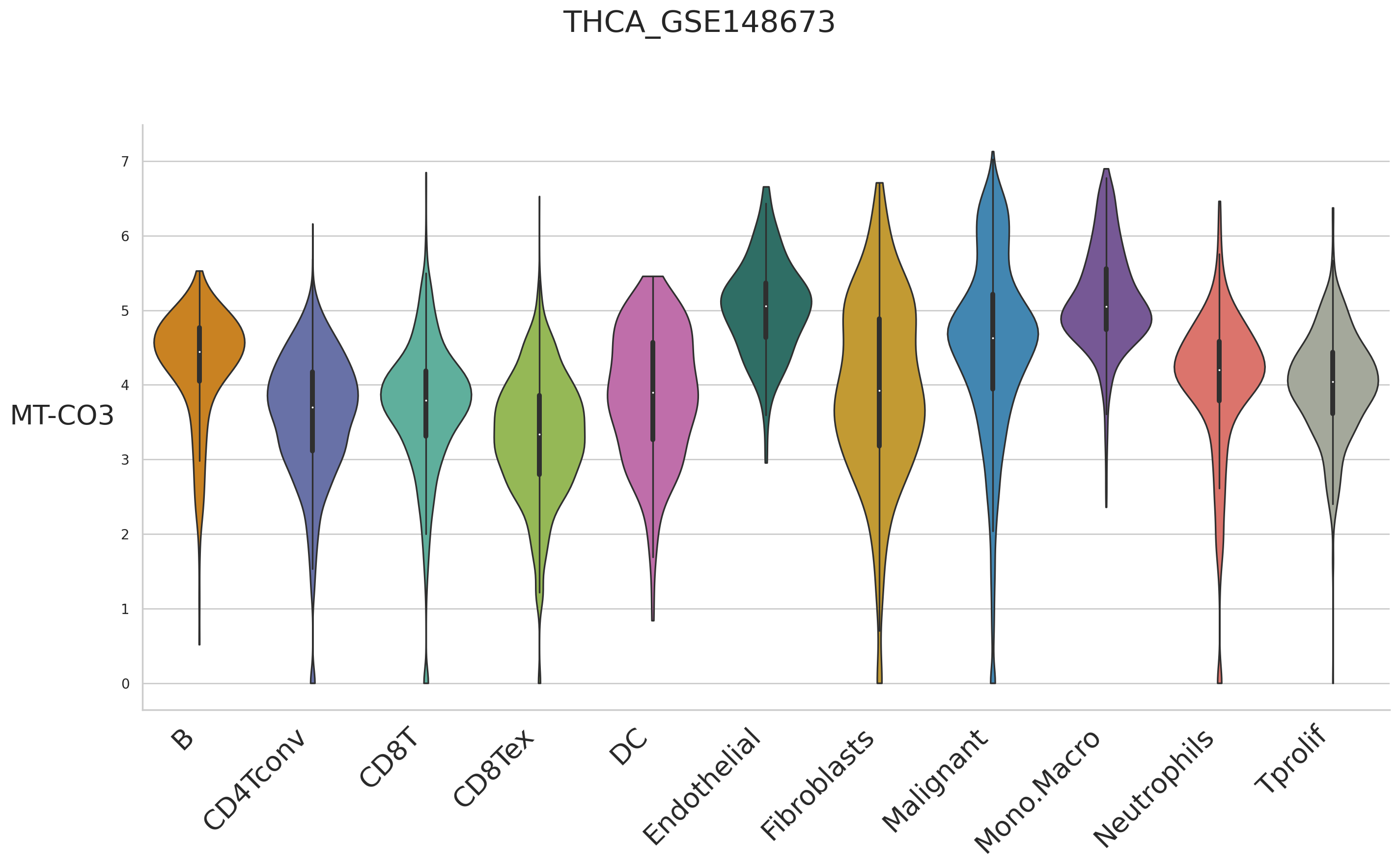

Supplement: Supplementary file 1 [file DataSheet_1.zip › huwpchraqw_THCA_GSE148673_violin_multiple_Celltype_curated_None.png]

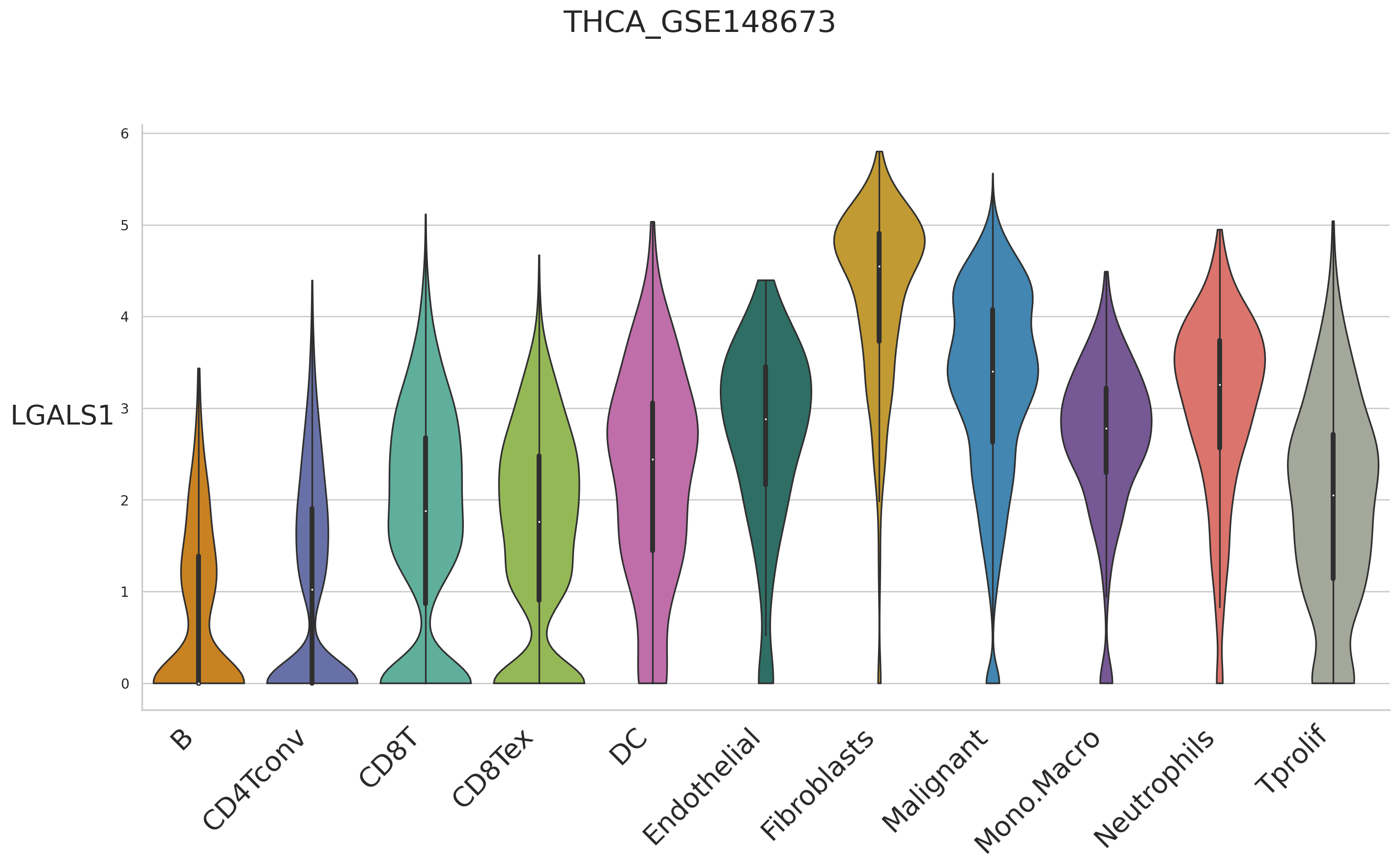

Supplement: Supplementary file 1 [file DataSheet_1.zip › ihbgtogztu_THCA_GSE148673_violin_multiple_Celltype_curated_None.png]

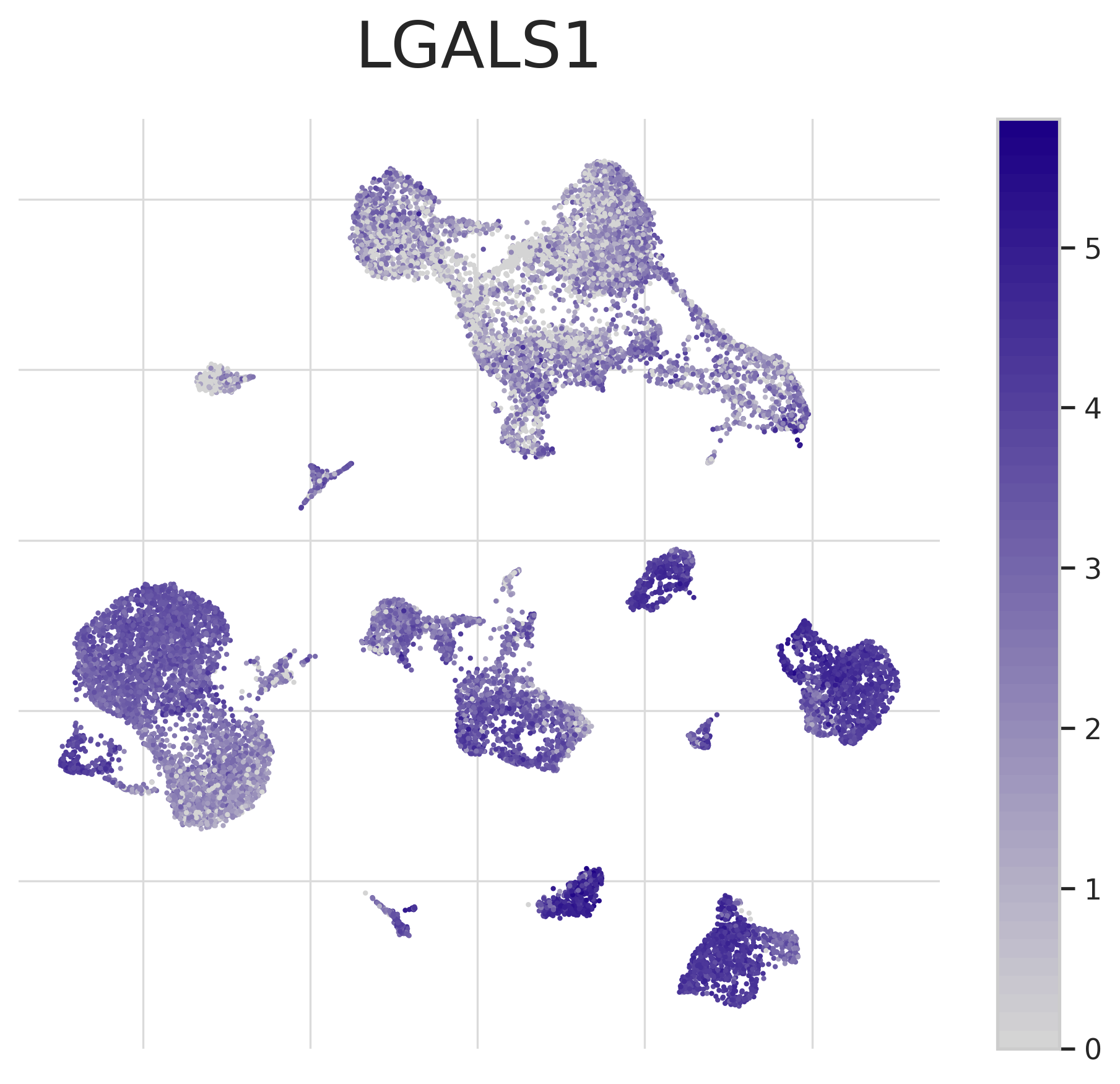

Supplement: Supplementary file 1 [file DataSheet_1.zip › ijflnbsqkn_THCA_GSE148673_LGALS1_umap.png]

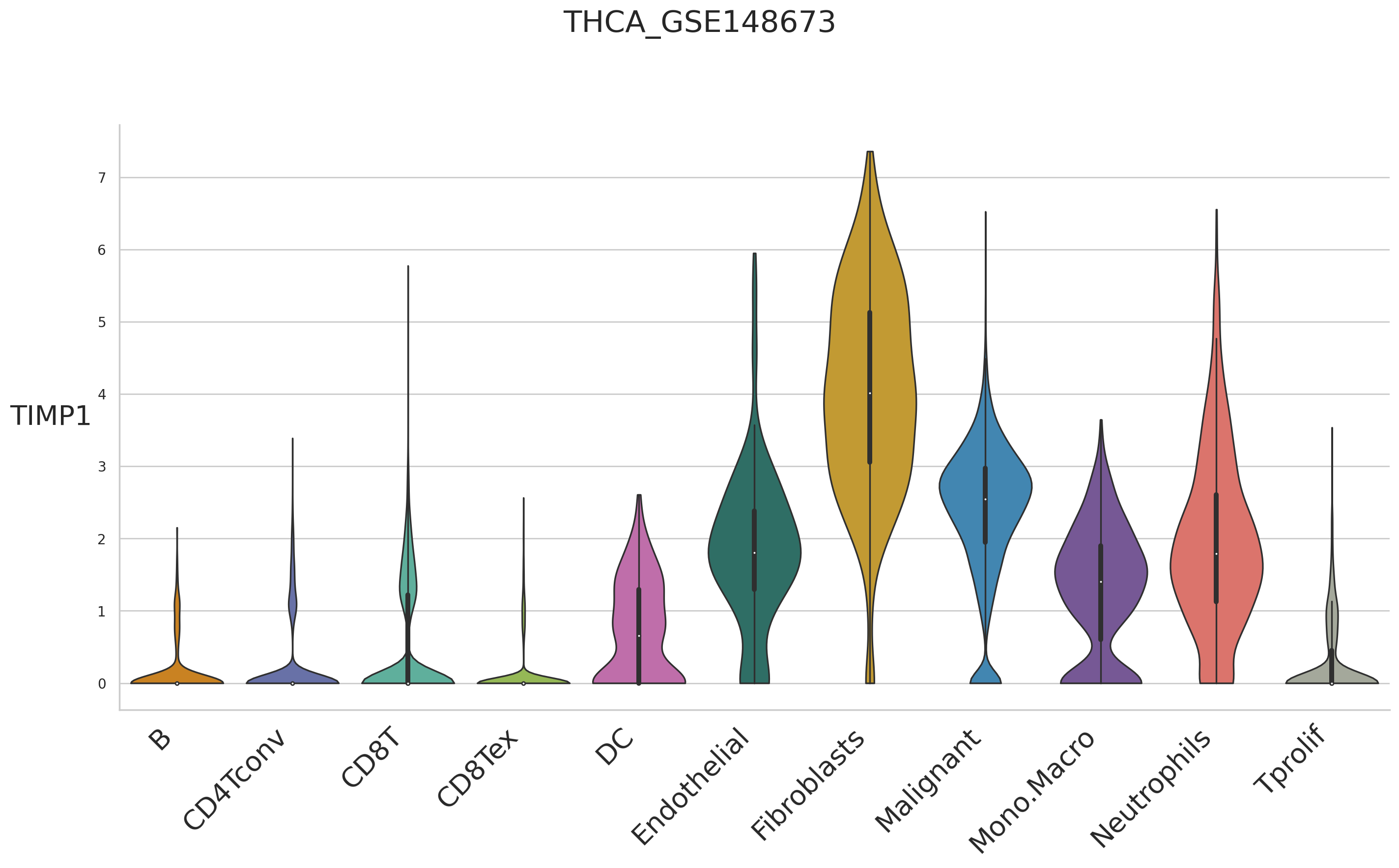

Supplement: Supplementary file 1 [file DataSheet_1.zip › ofsjwmrvft_THCA_GSE148673_violin_multiple_Celltype_curated_None.png]

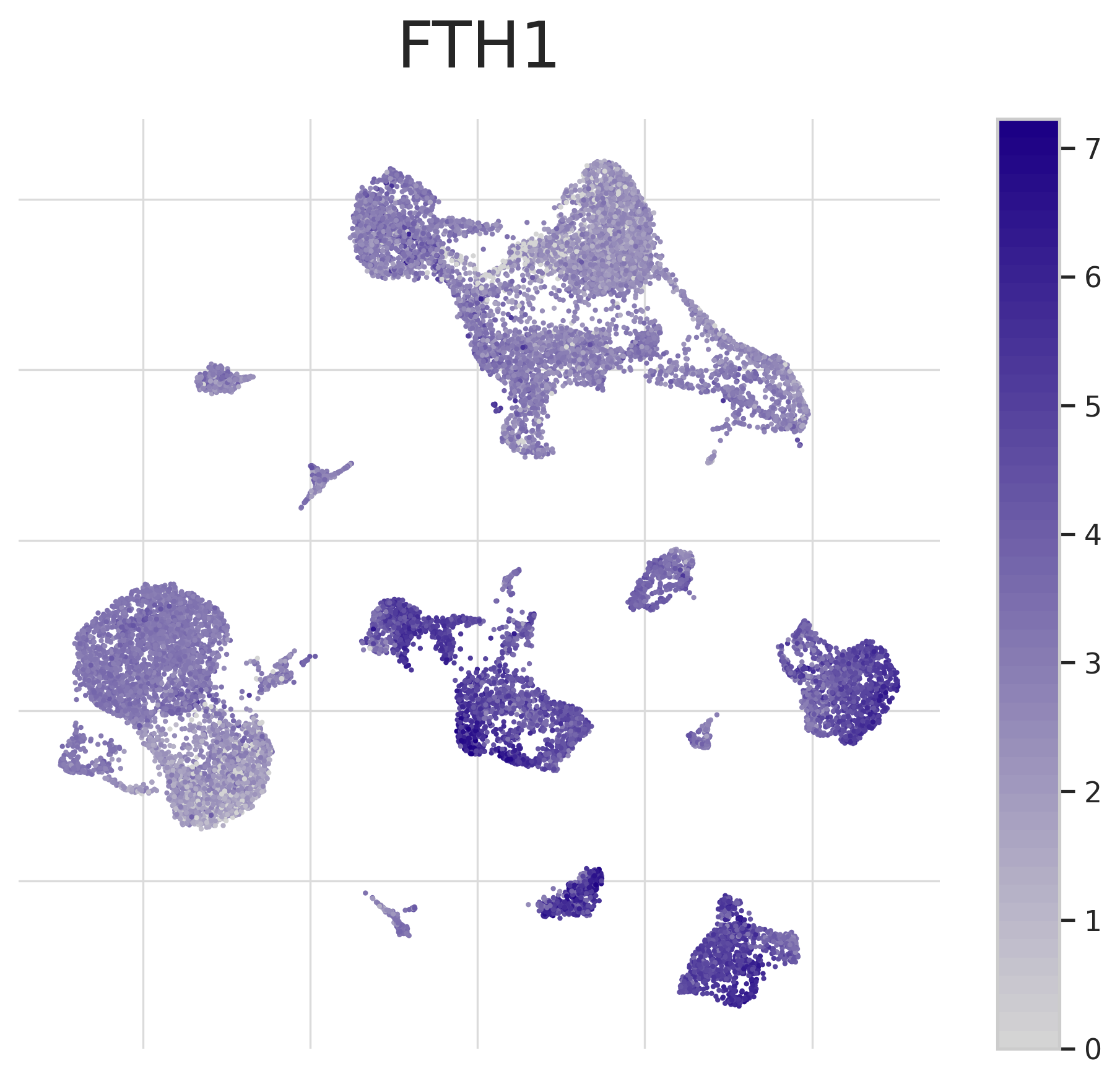

Supplement: Supplementary file 1 [file DataSheet_1.zip › pdifzqahyh_THCA_GSE148673_FTH1_umap.png]

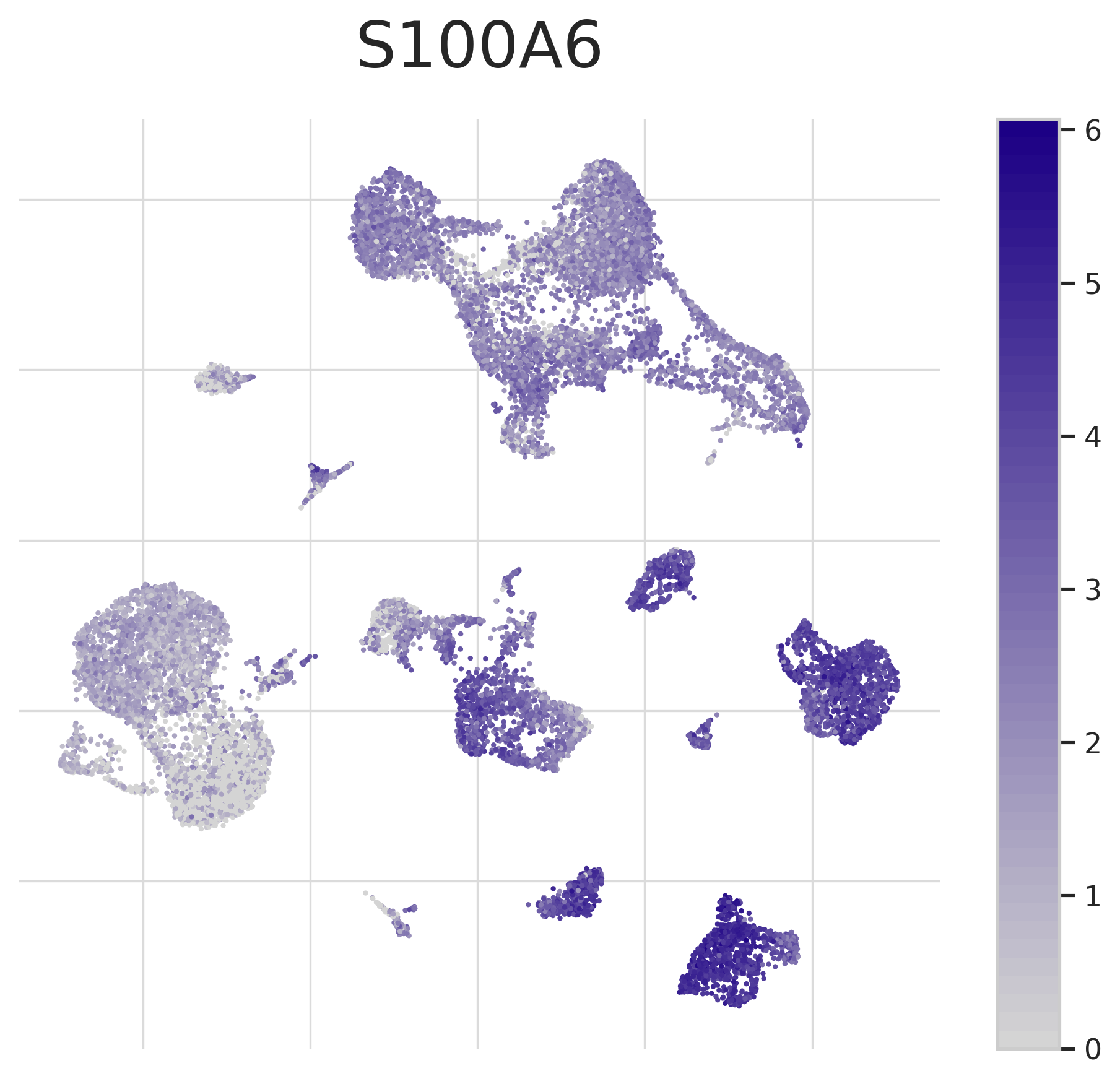

Supplement: Supplementary file 1 [file DataSheet_1.zip › qalgkoiuaq_THCA_GSE148673_S100A6_umap.png]

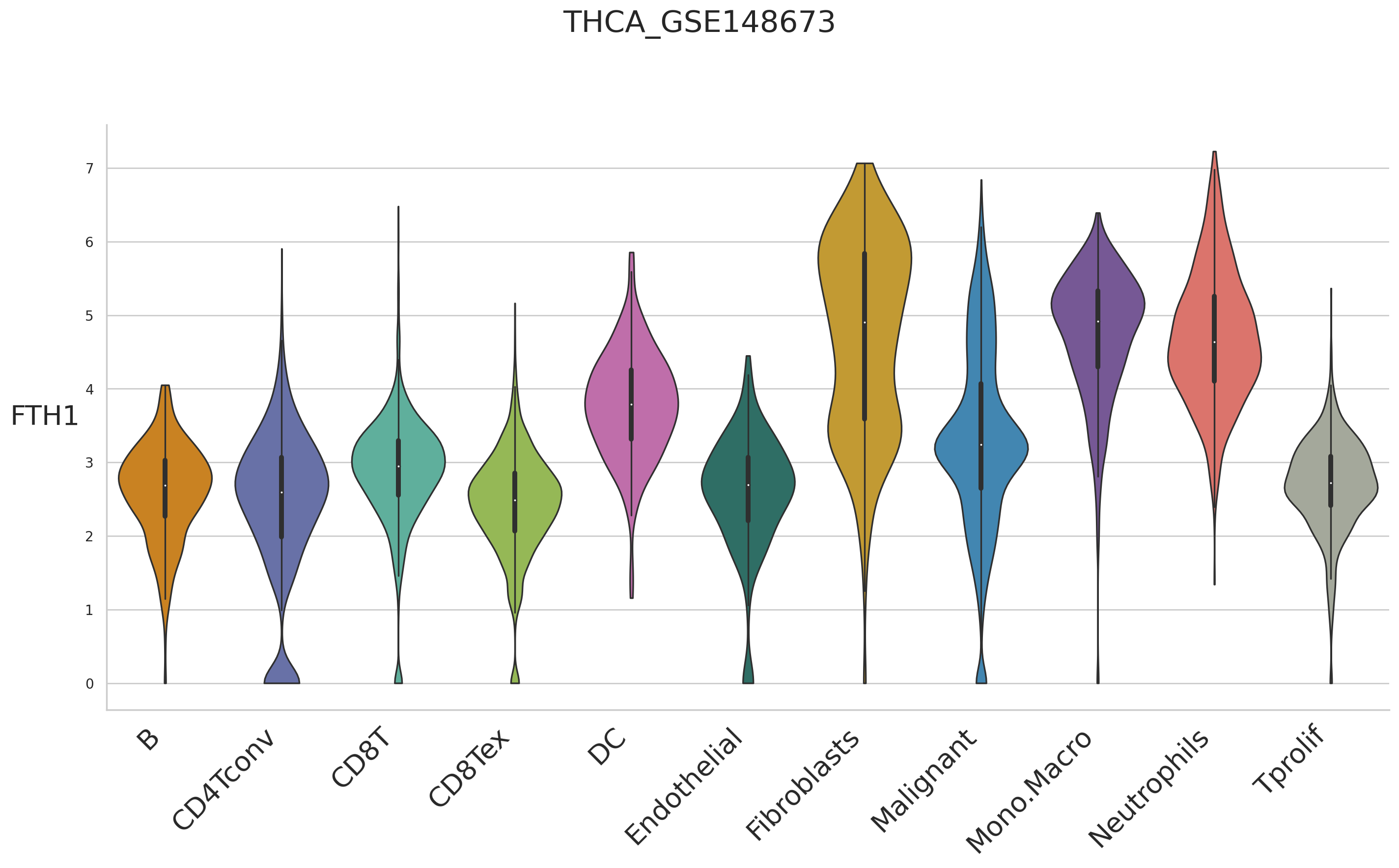

Supplement: Supplementary file 1 [file DataSheet_1.zip › qoevuospbh_THCA_GSE148673_violin_multiple_Celltype_curated_None.png]

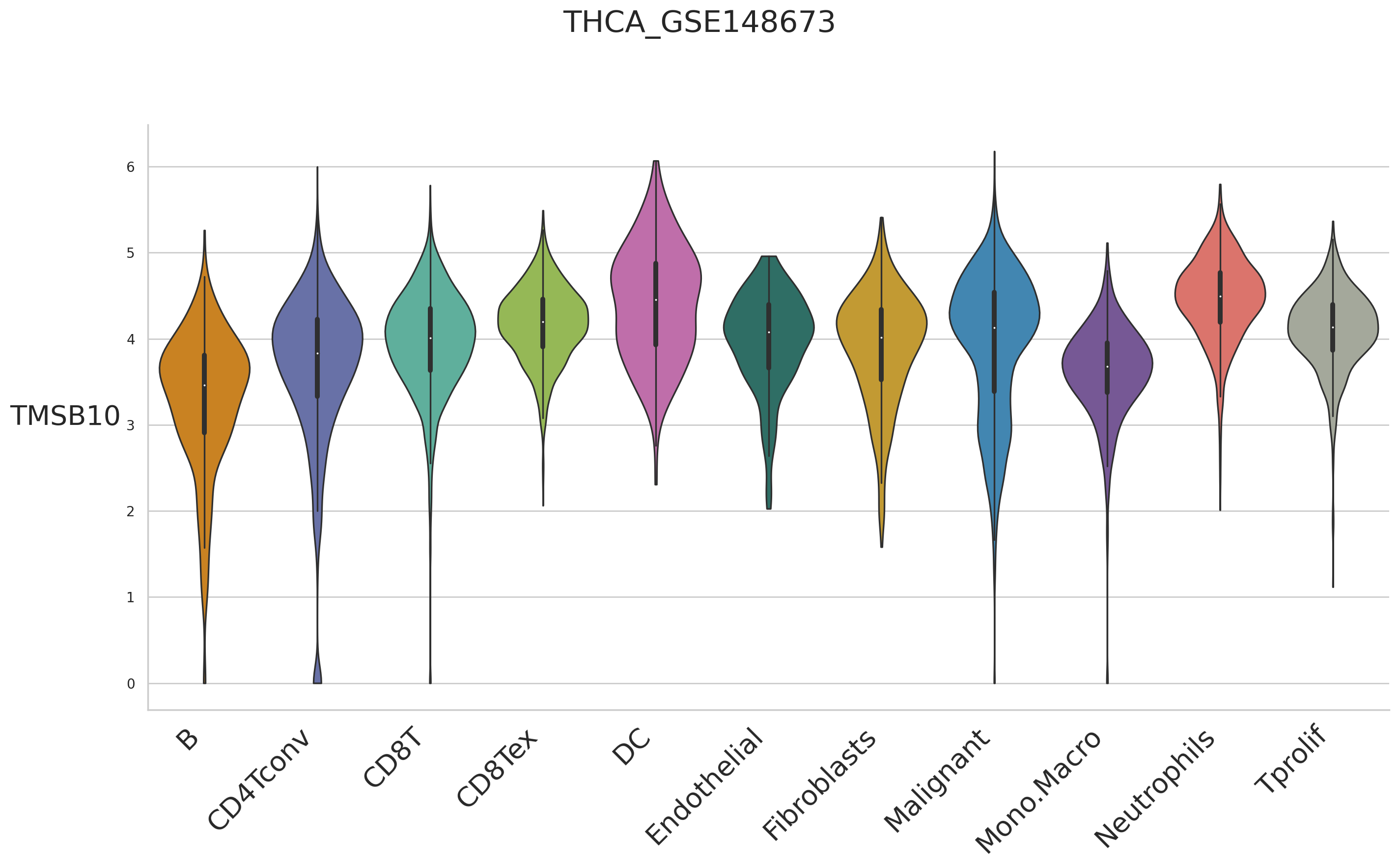

Supplement: Supplementary file 1 [file DataSheet_1.zip › qwsisyiwwl_THCA_GSE148673_violin_multiple_Celltype_curated_None.png]

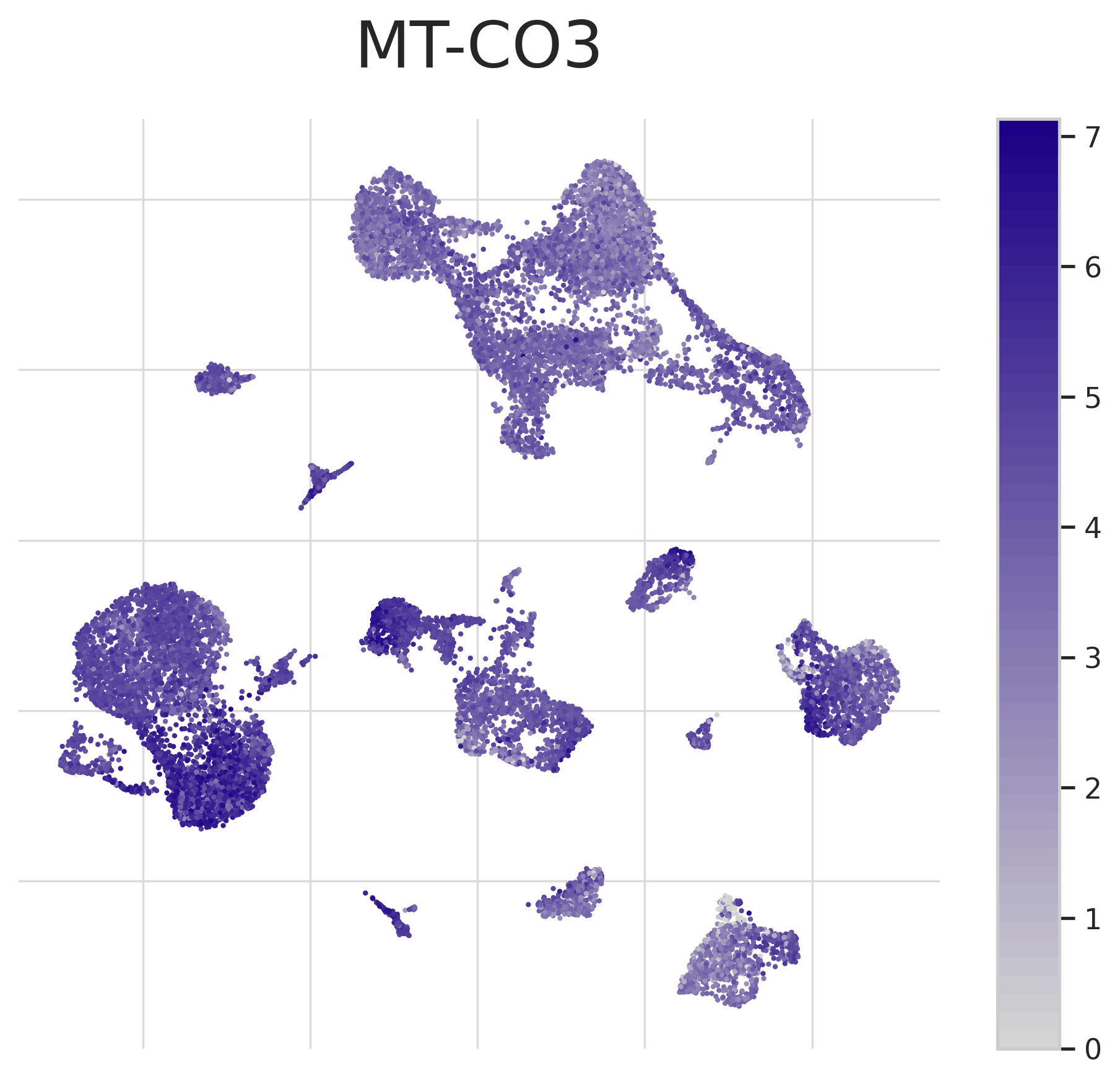

Supplement: Supplementary file 1 [file DataSheet_1.zip › sjxwrsqvqq_THCA_GSE148673_MT-CO3_umap.png]

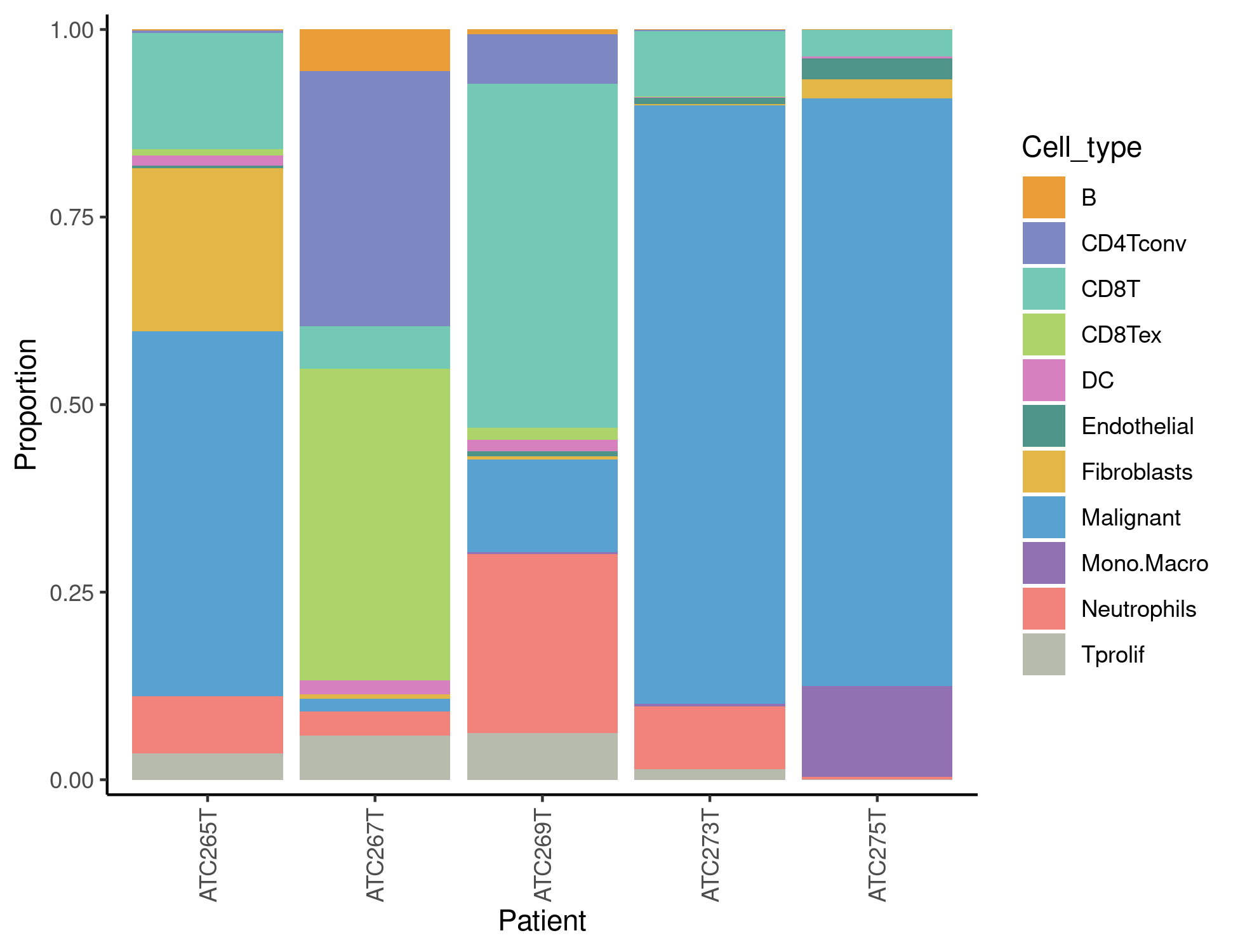

Supplement: Supplementary file 1 [file DataSheet_1.zip › THCA_GSE148673_barplot.png]

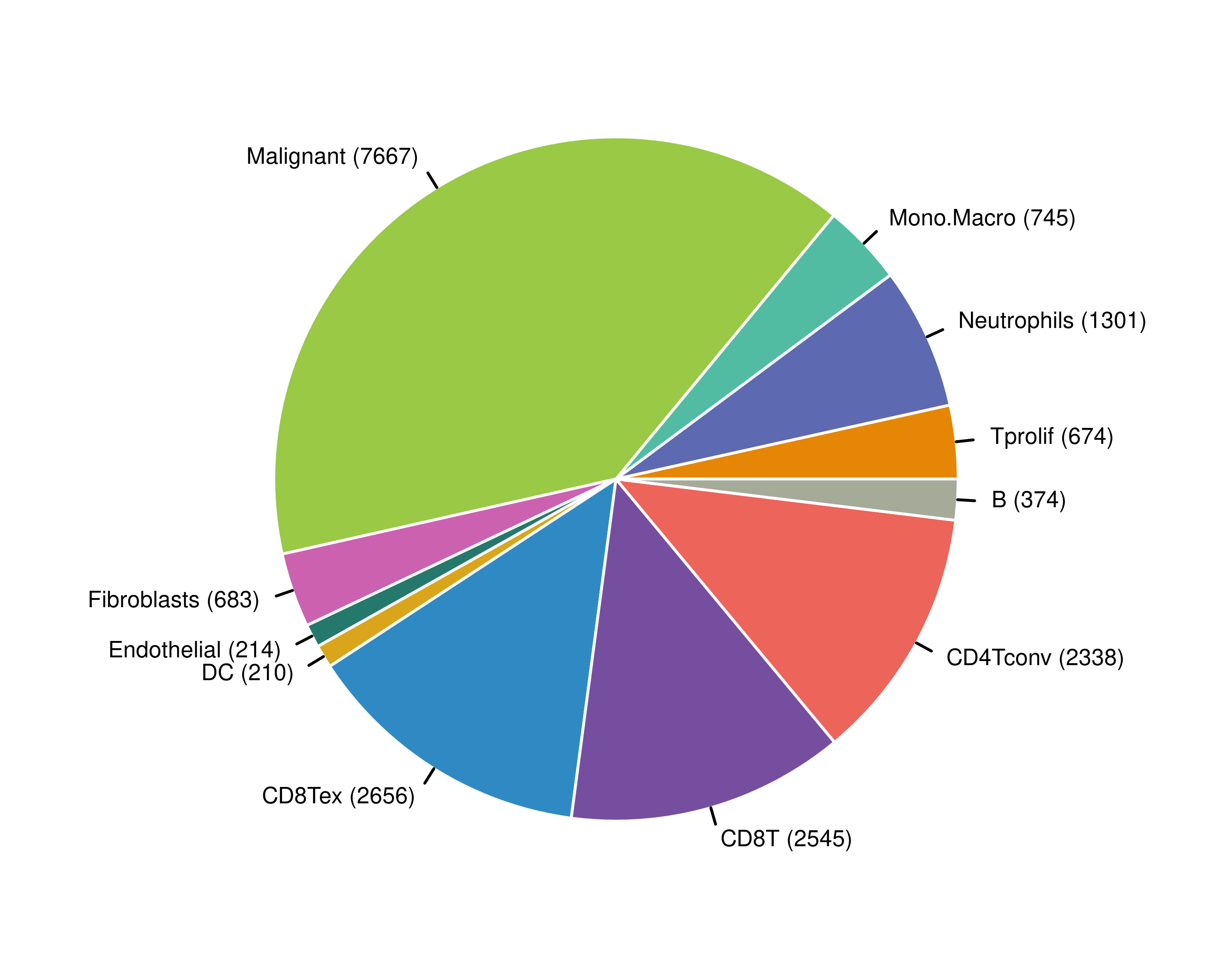

Supplement: Supplementary file 1 [file DataSheet_1.zip › THCA_GSE148673_pie.png]

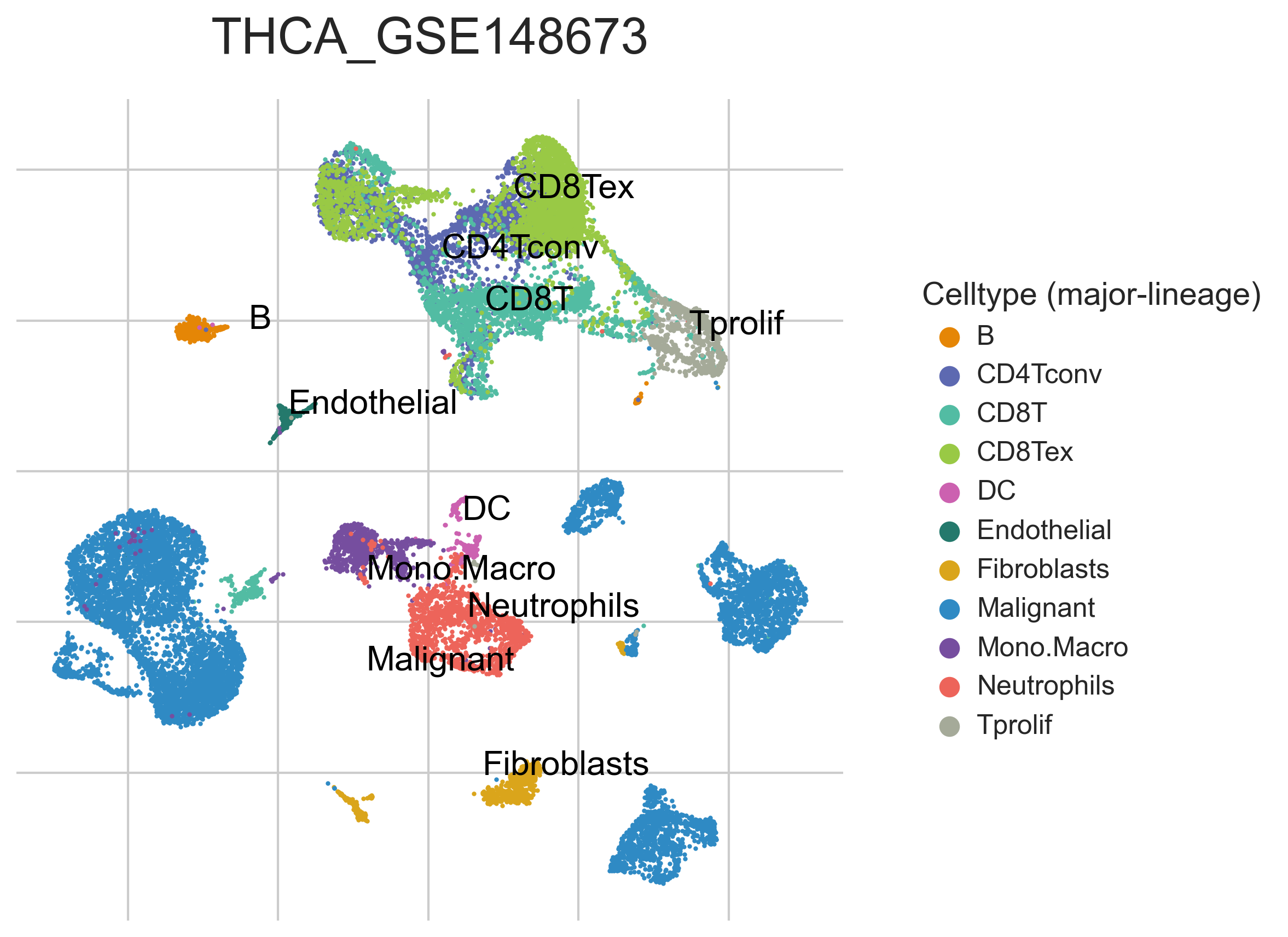

Supplement: Supplementary file 1 [file DataSheet_1.zip › THCA_GSE148673_umap_Celltype_curated.png]

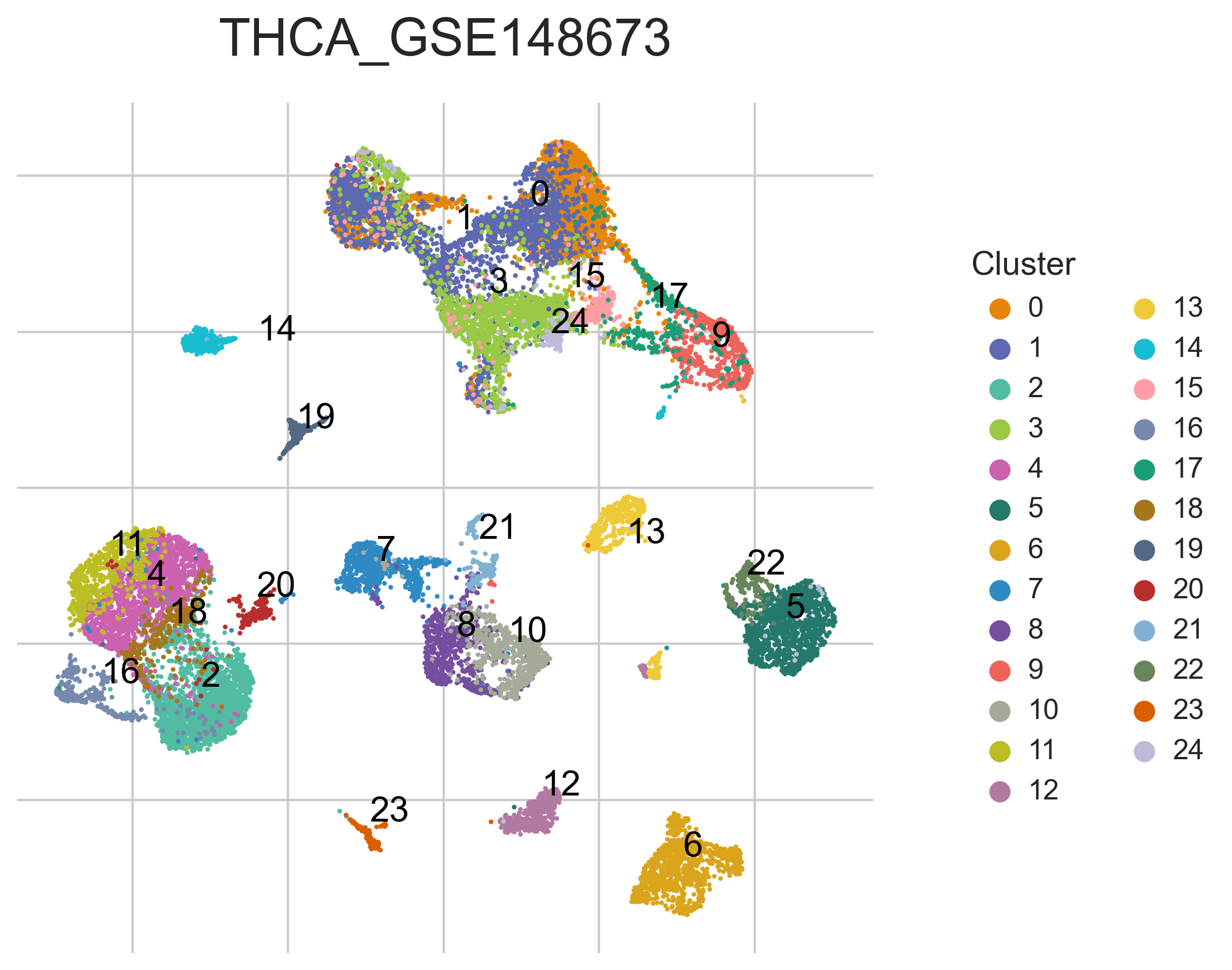

Supplement: Supplementary file 1 [file DataSheet_1.zip › THCA_GSE148673_umap_Cluster.png]

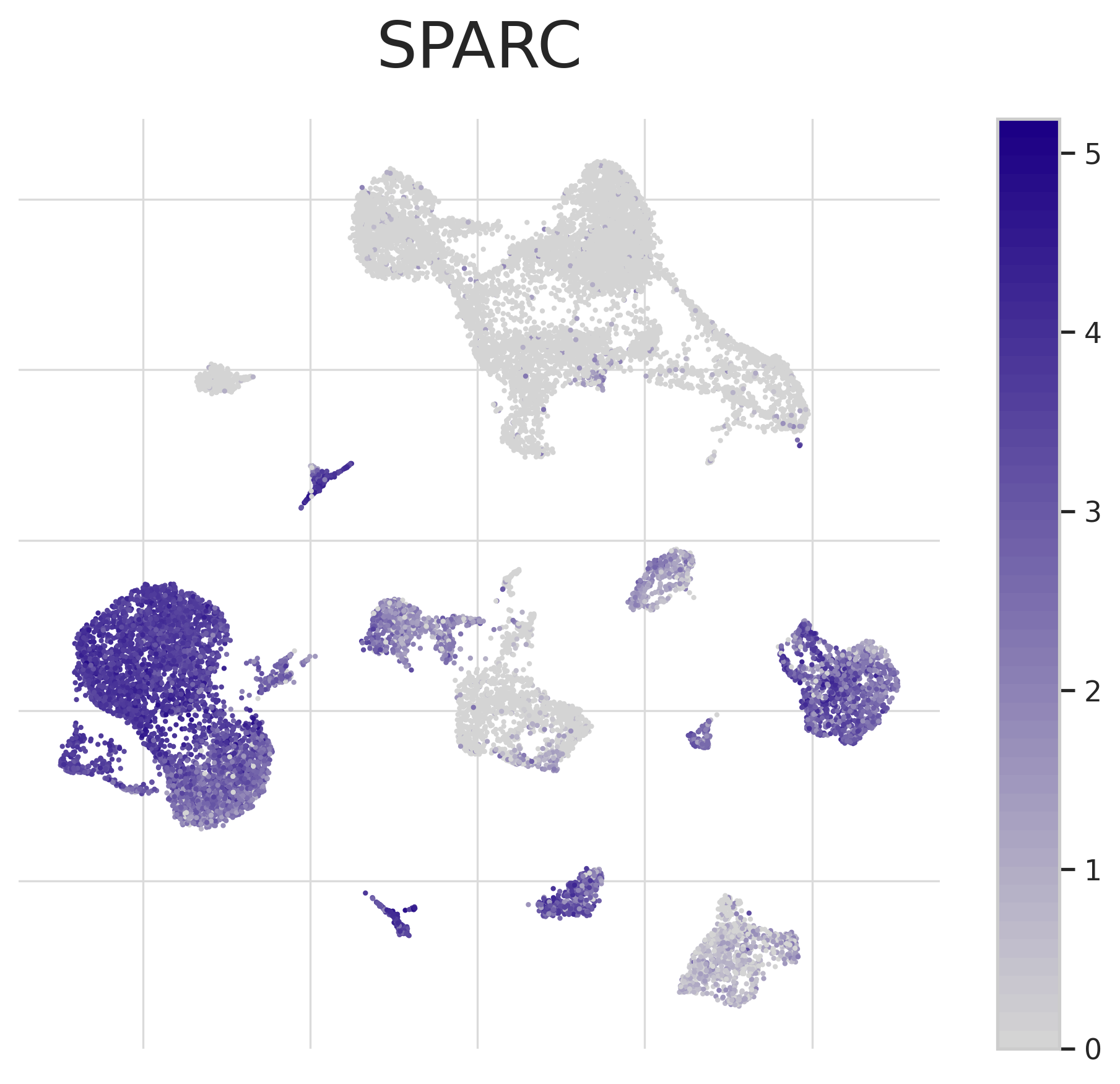

Supplement: Supplementary file 1 [file DataSheet_1.zip › xtdanbrcuv_THCA_GSE148673_SPARC_umap.png]

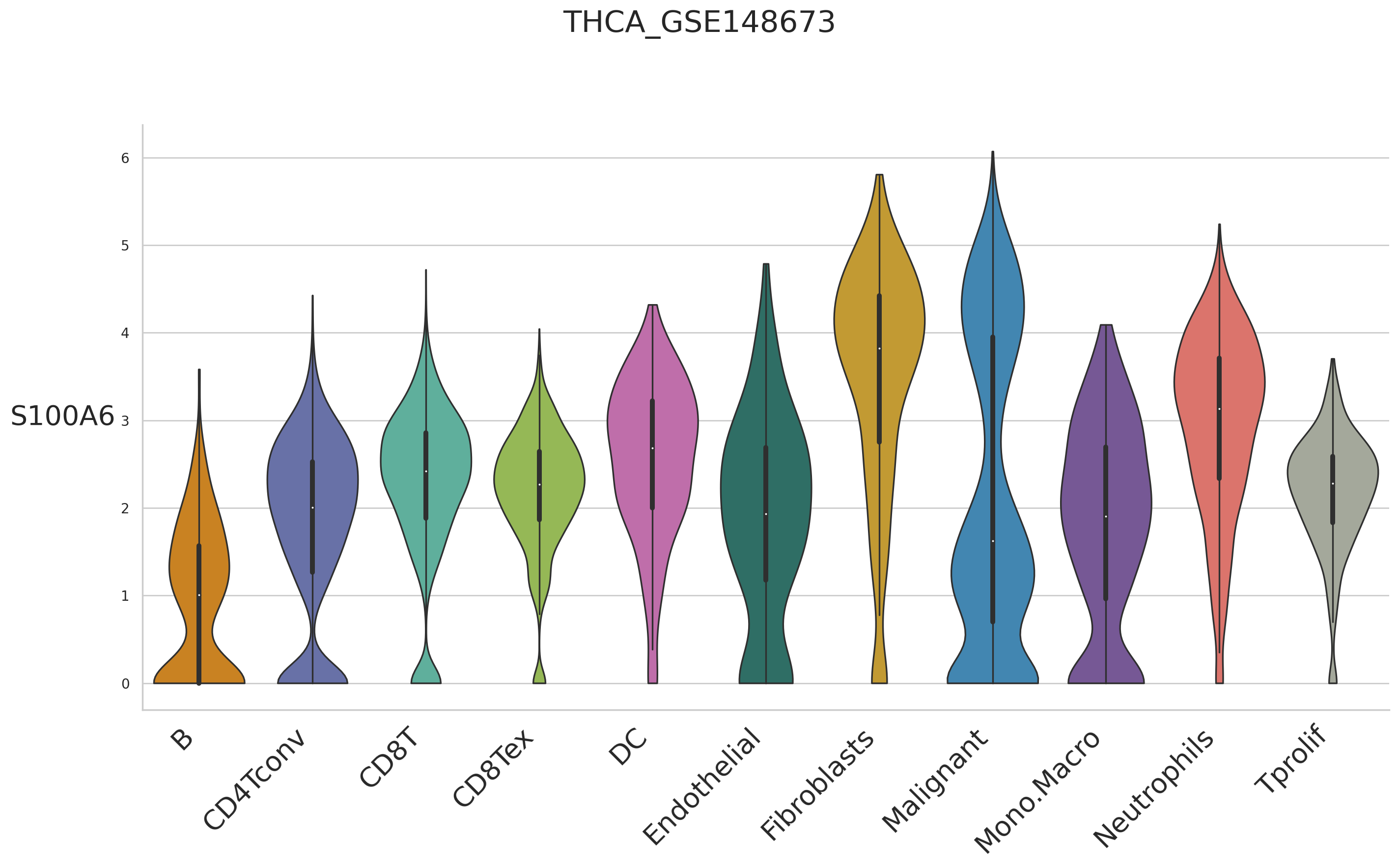

Supplement: Supplementary file 1 [file DataSheet_1.zip › ydrujfsoyf_THCA_GSE148673_violin_multiple_Celltype_curated_None.png]

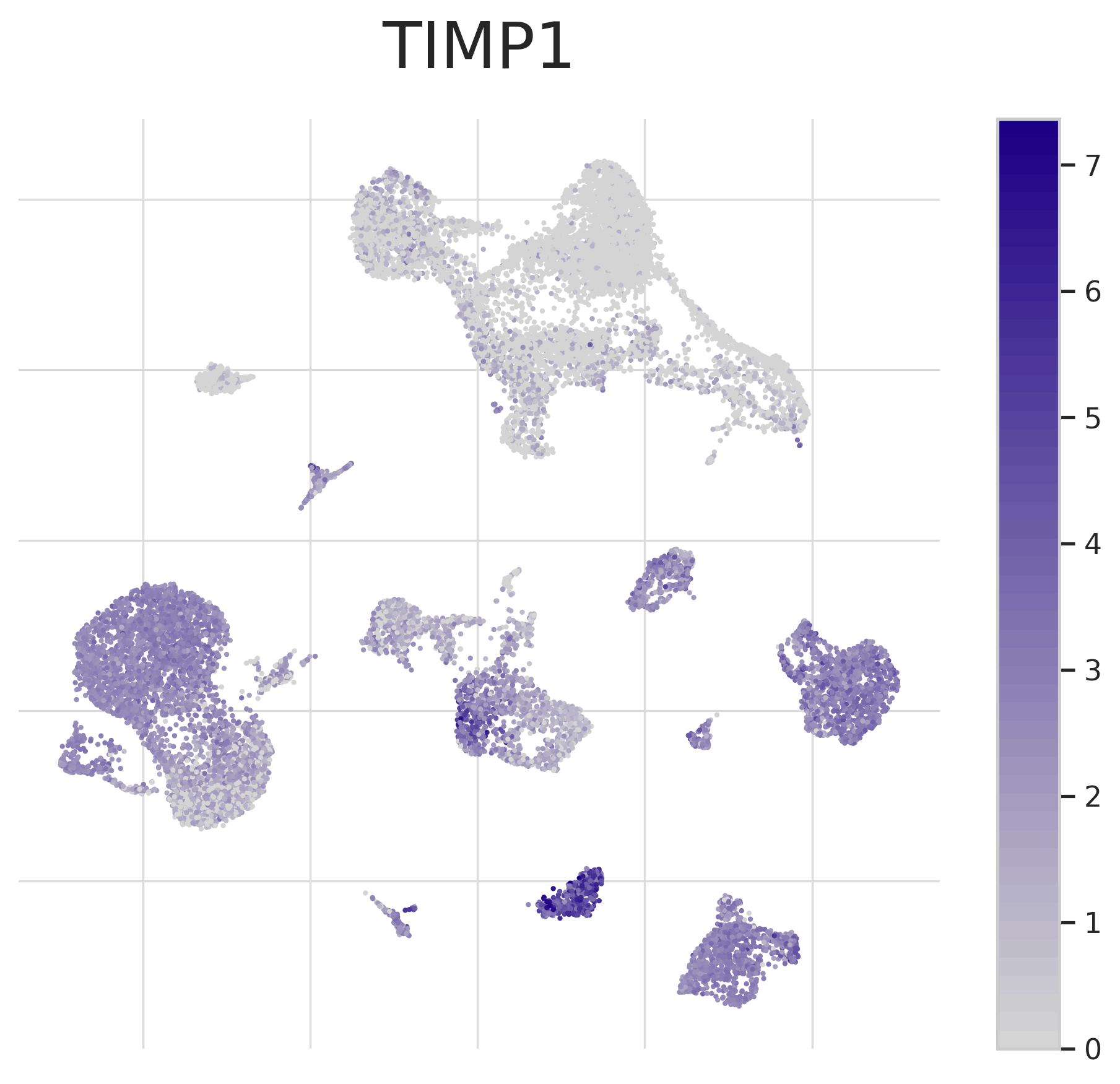

Supplement: Supplementary file 1 [file DataSheet_1.zip › ygtxlfqaly_THCA_GSE148673_TIMP1_umap.png]

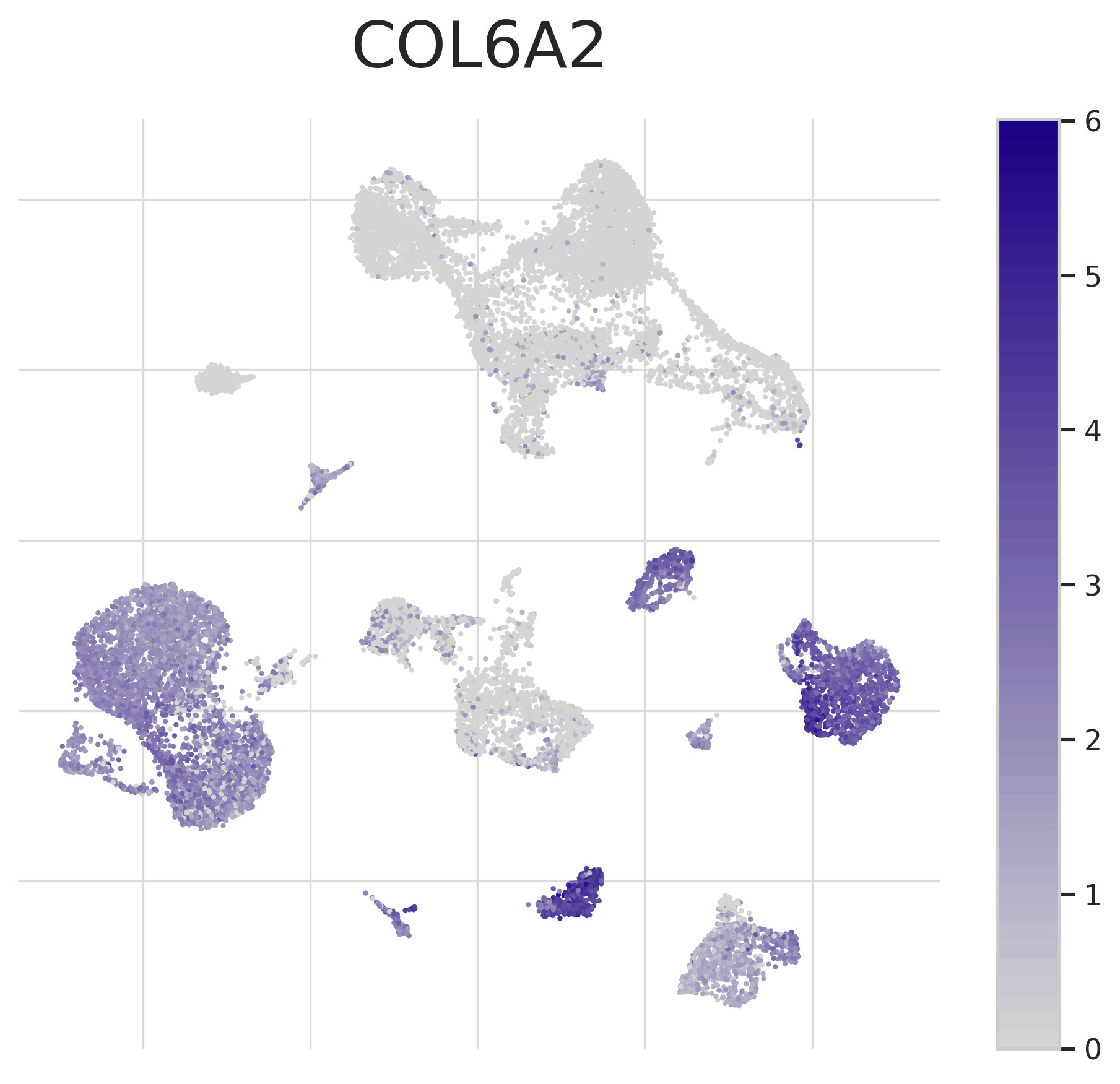

Supplement: Supplementary file 1 [file DataSheet_1.zip › zbdmejbedk_THCA_GSE148673_COL6A2_umap.png]

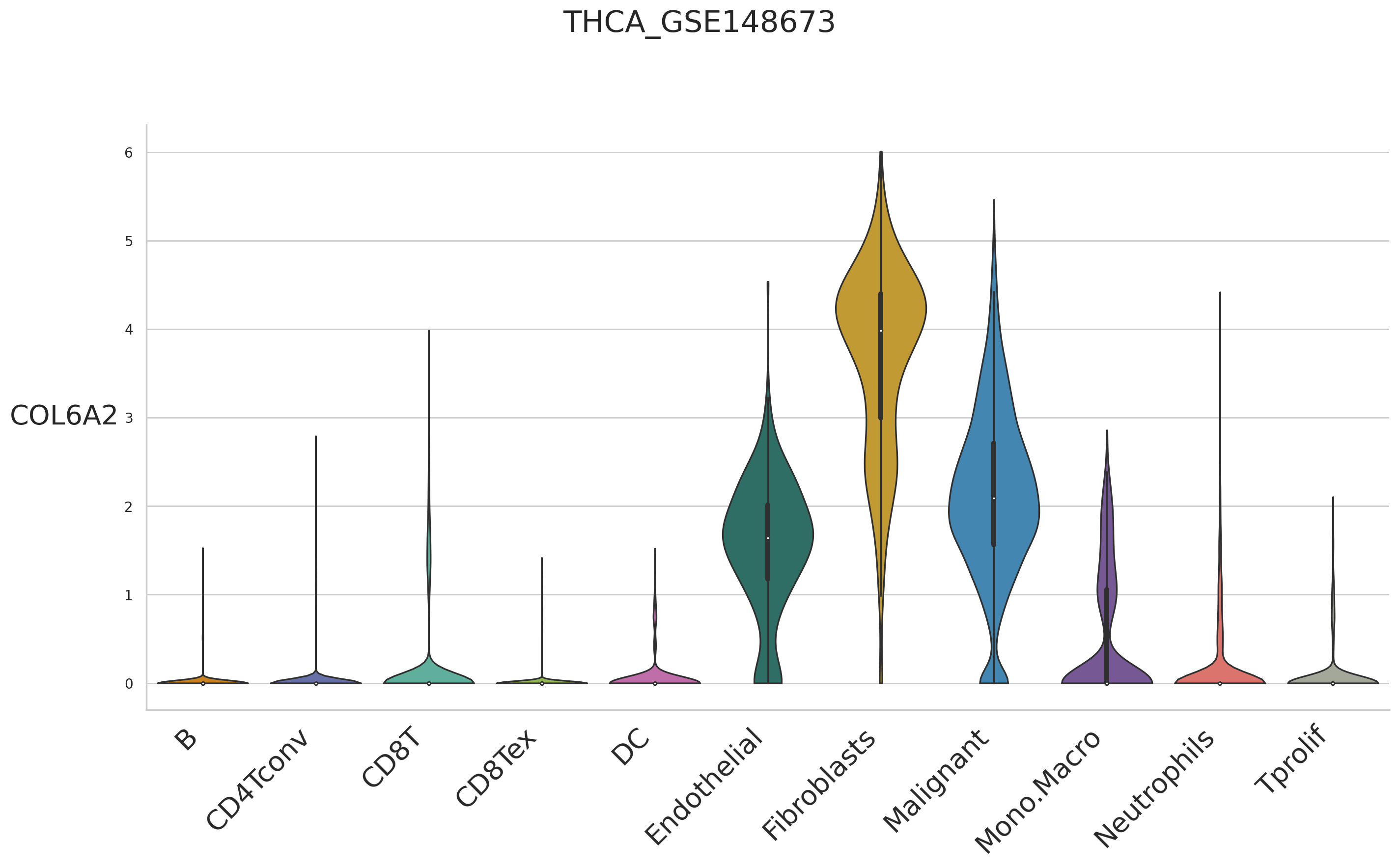

Supplement: Supplementary file 1 [file DataSheet_1.zip › zzsugrdkqd_THCA_GSE148673_violin_multiple_Celltype_curated_None.png]

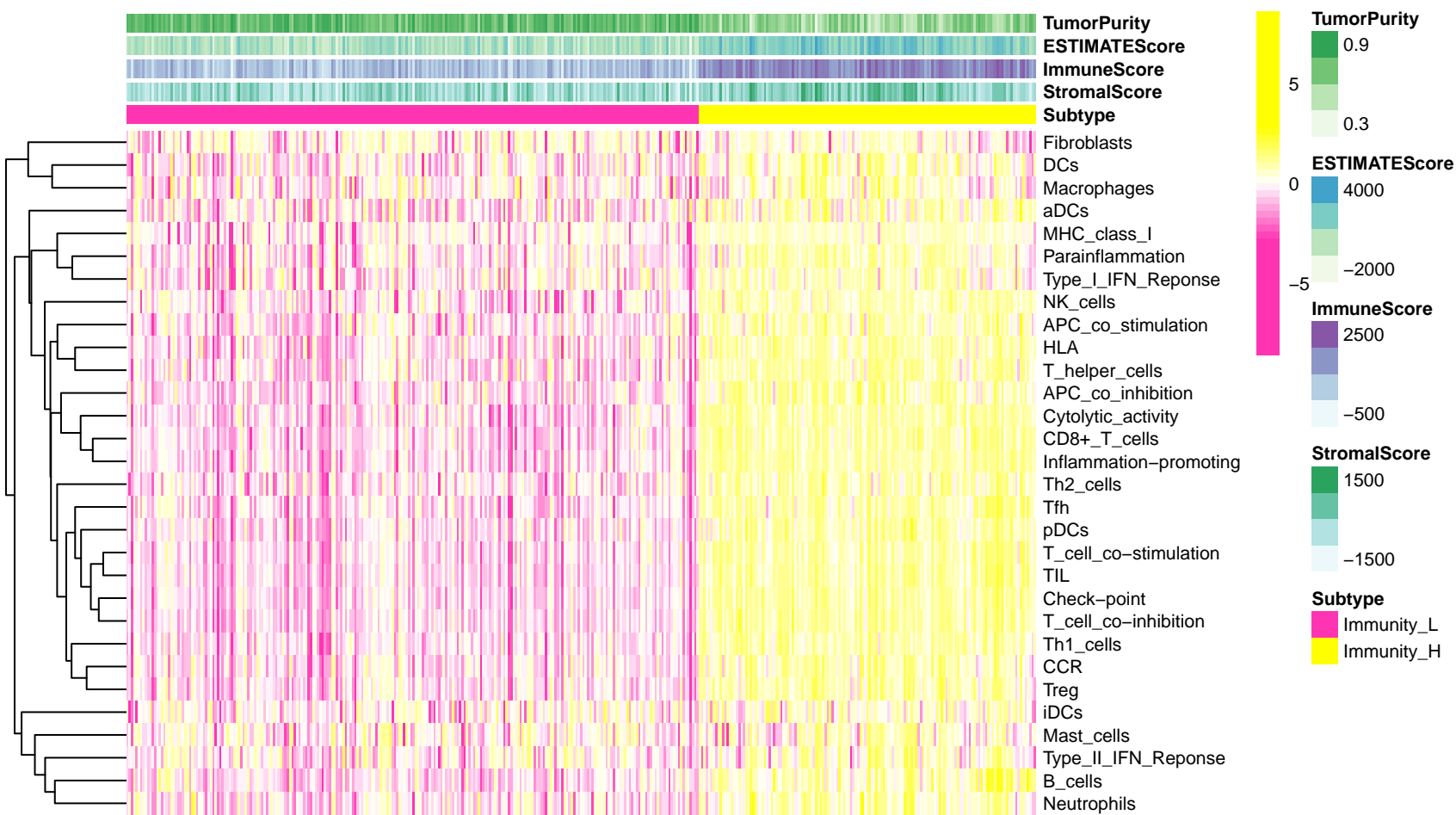

Supplement: Supplementary file 2 [file DataSheet_2.zip › estimateHM.pdf]

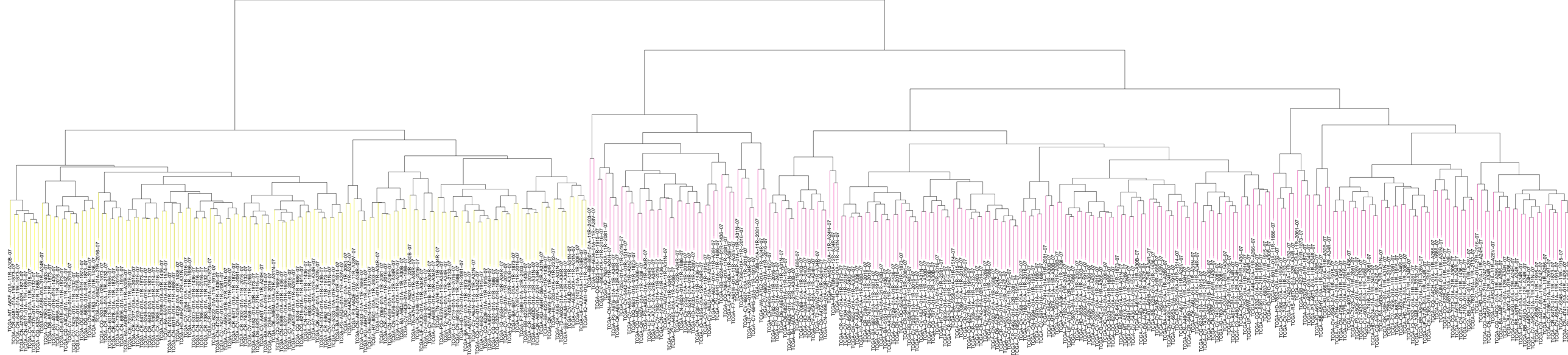

Supplement: Supplementary file 2 [file DataSheet_2.zip › hclust.pdf]

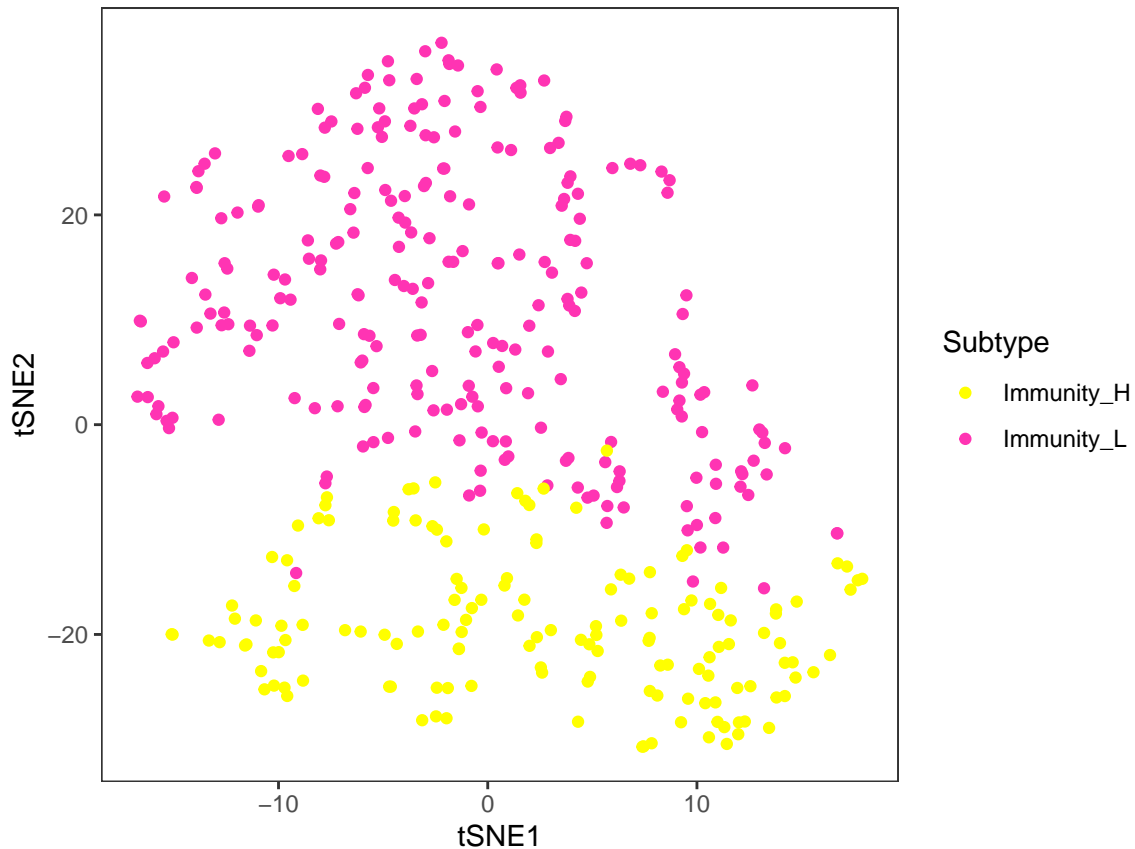

Supplement: Supplementary file 2 [file DataSheet_2.zip › tSNE.pdf]

Subtype

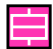

Immunity\_L

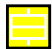

Immunity\_H

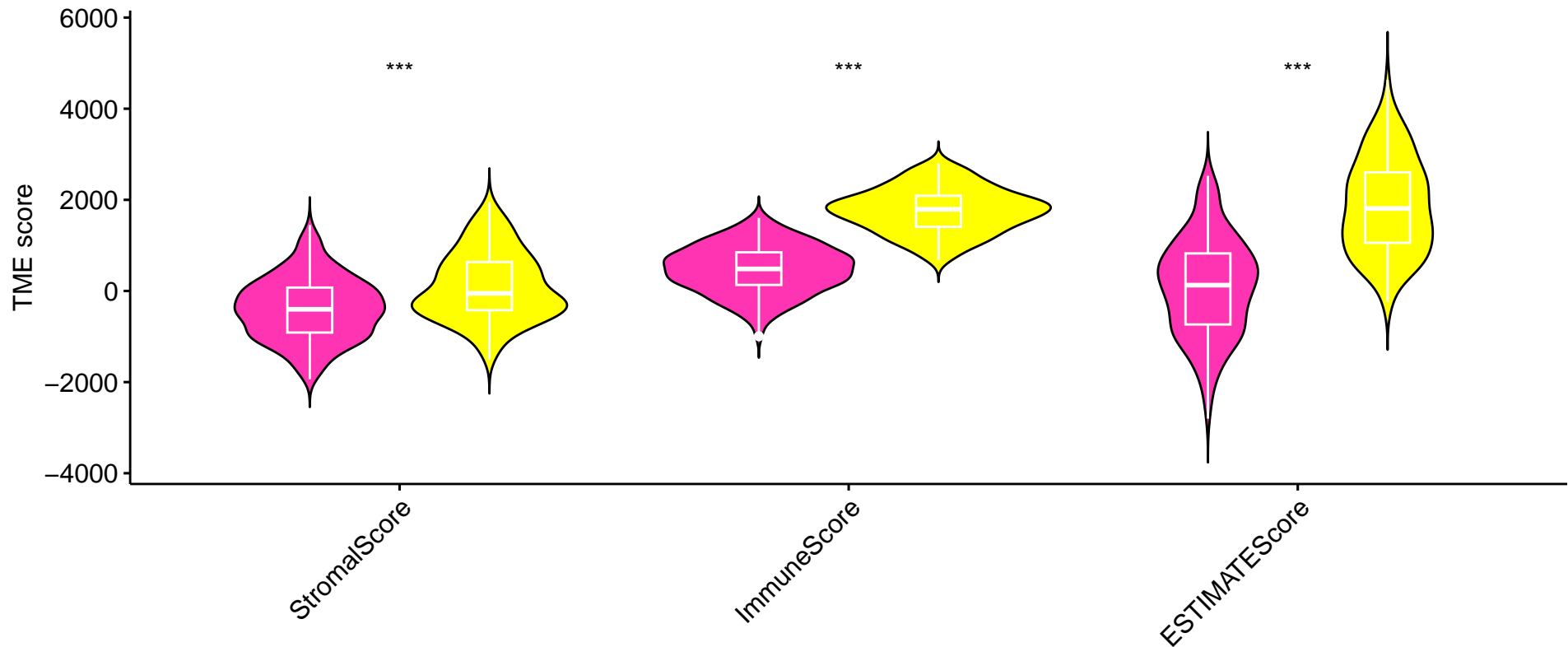

Supplement: Supplementary file 2 [file DataSheet_2.zip › vioplot.pdf]

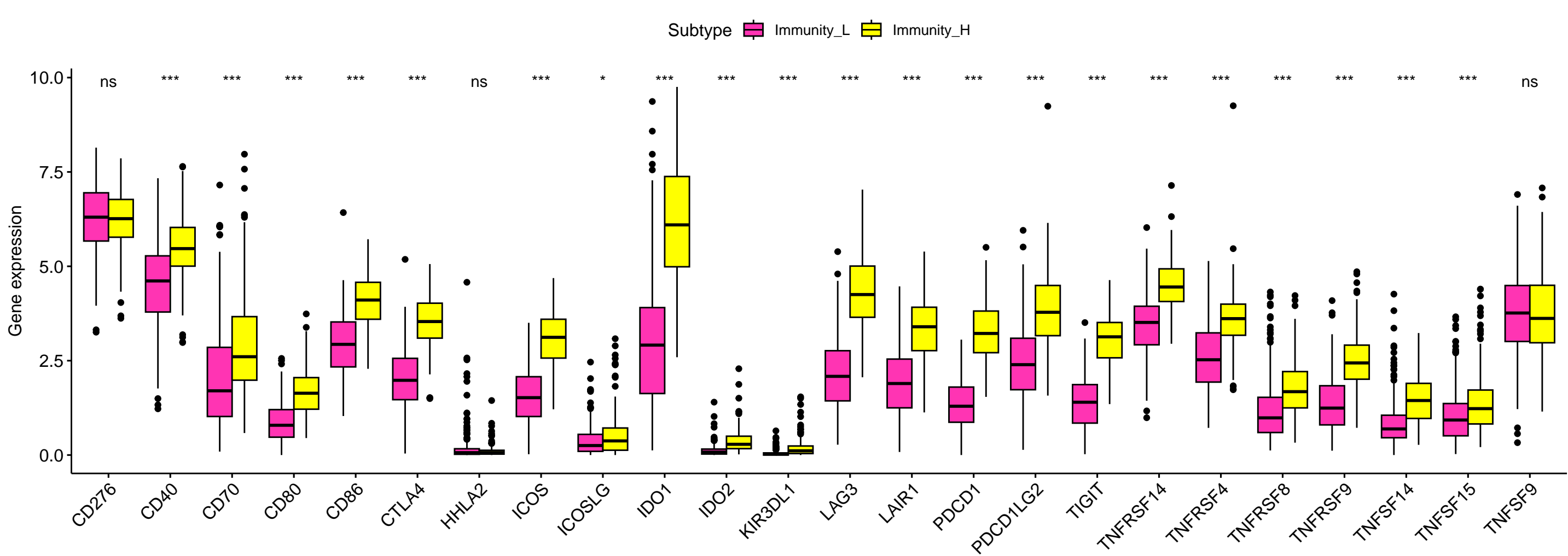

Supplement: Supplementary file 3 [file DataSheet_3.zip › CP1.pdf]

Subtype Immunity\_L Immunity\_H

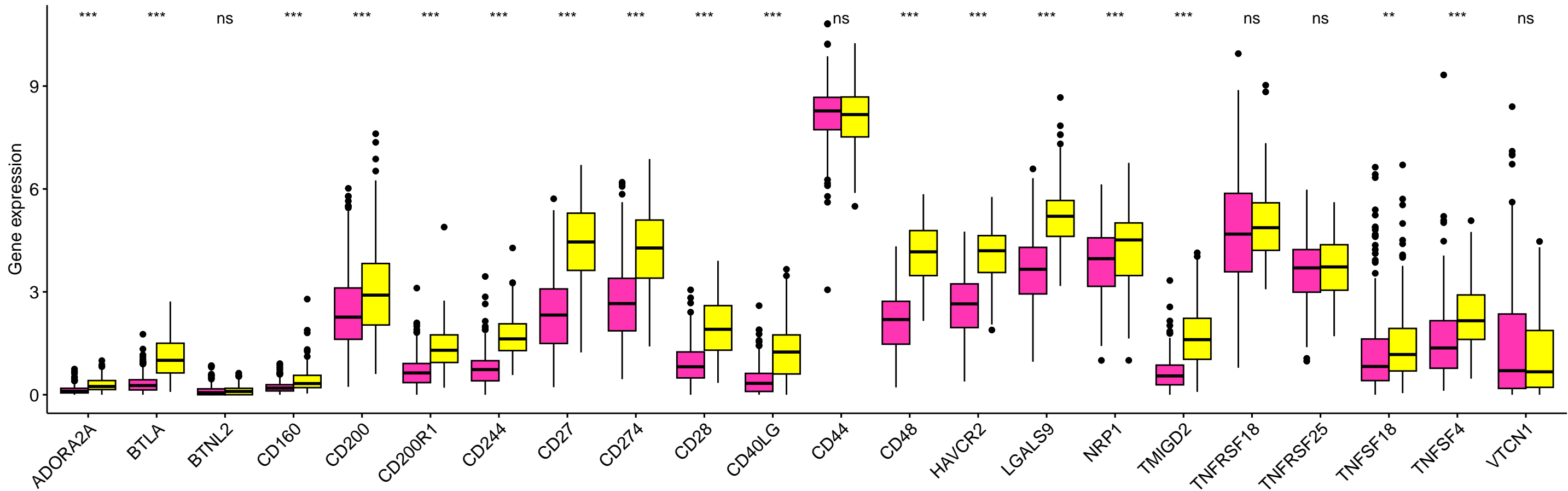

Supplement: Supplementary file 3 [file DataSheet_3.zip › CP2.pdf]

Subtype Immunity\_L Immunity\_H

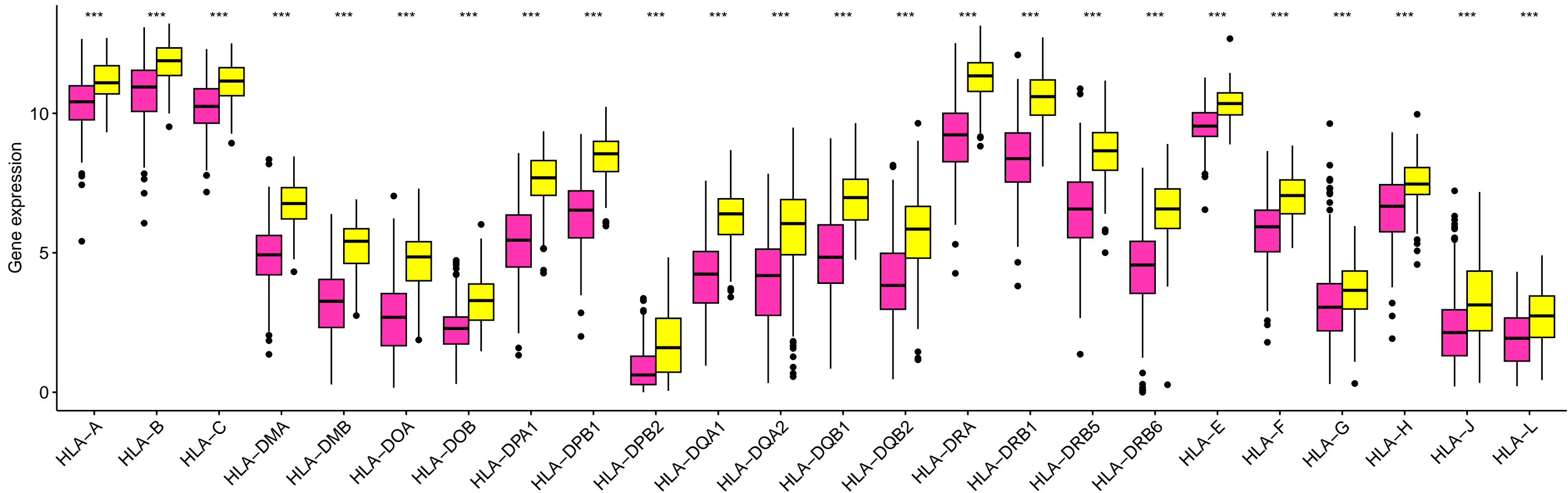

Supplement: Supplementary file 3 [file DataSheet_3.zip › HLA.pdf]

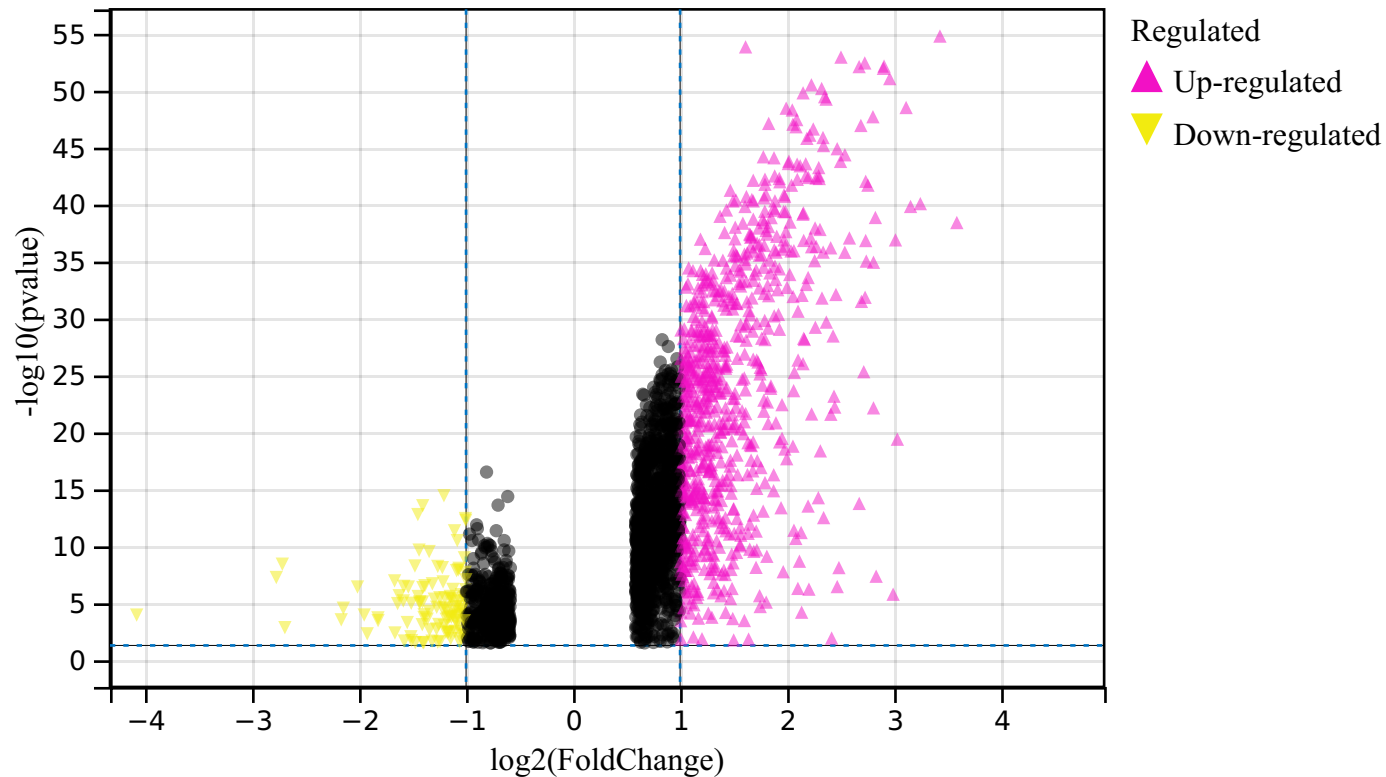

Supplement: Supplementary file 4 [file DataSheet_4.zip › 9f630b3a1c6d7810d7c2810b9a859af2.pdf]

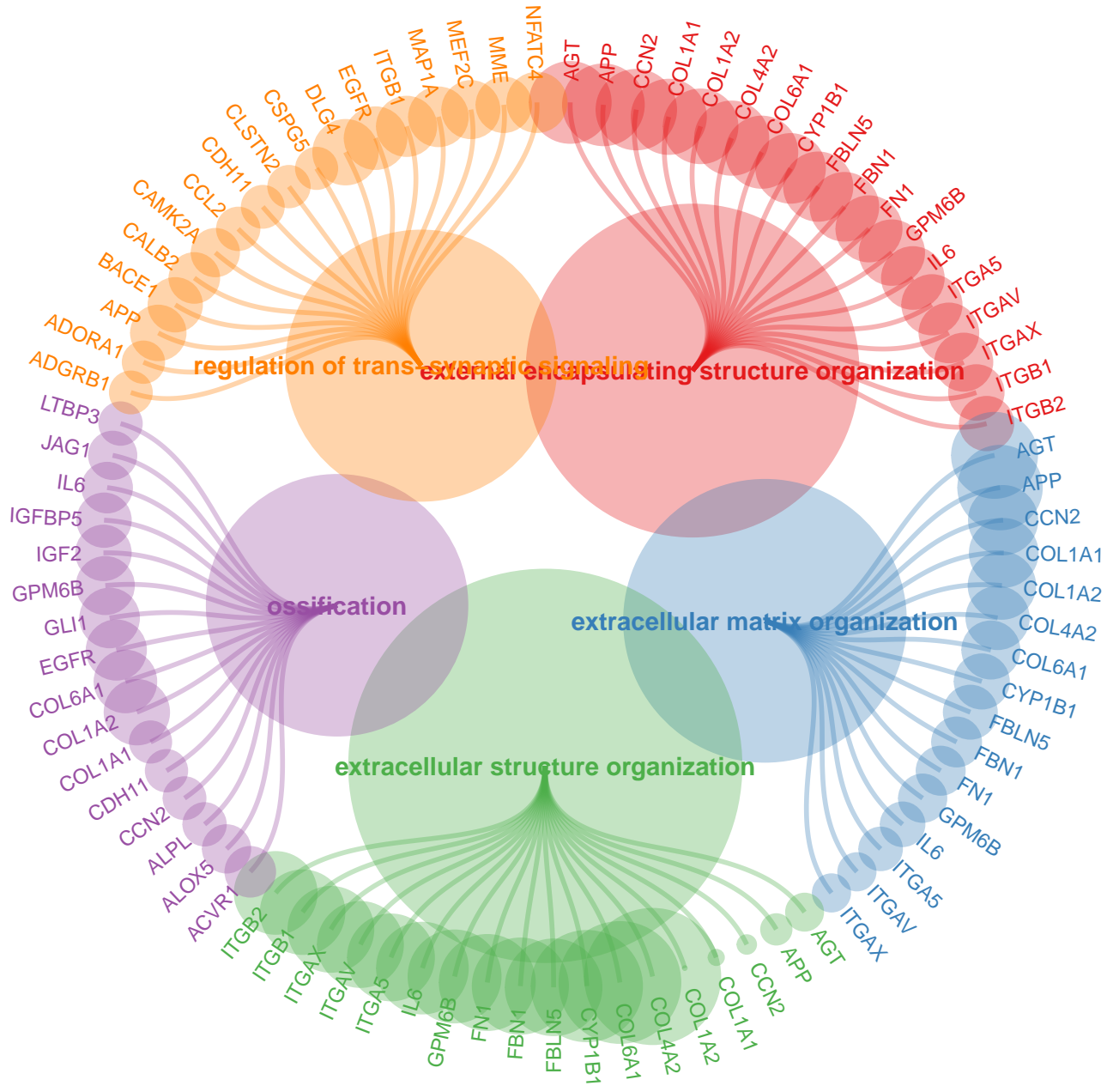

Supplement: Supplementary file 4 [file DataSheet_4.zip › BP.pdf]

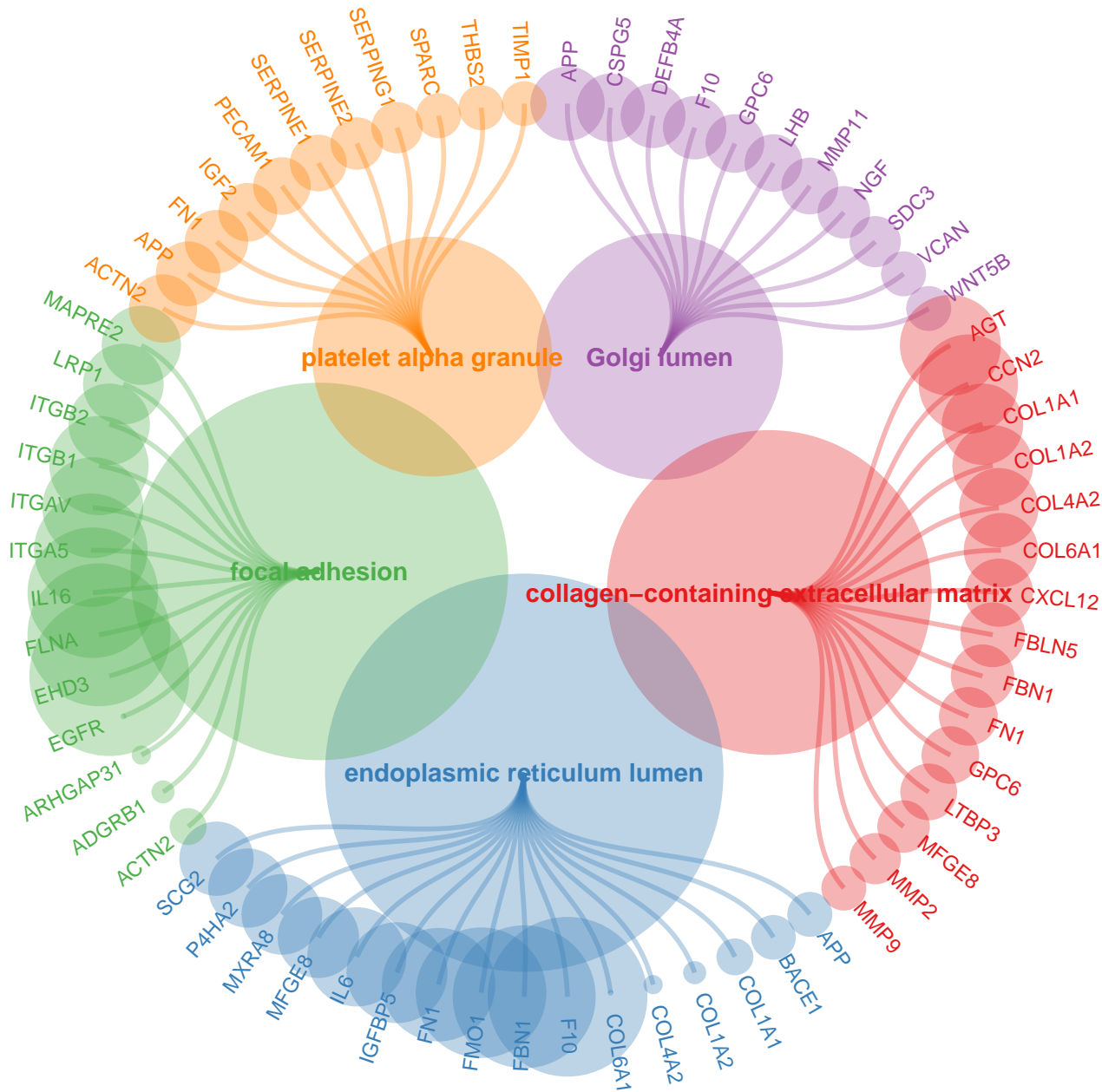

Supplement: Supplementary file 4 [file DataSheet_4.zip › CC.pdf]

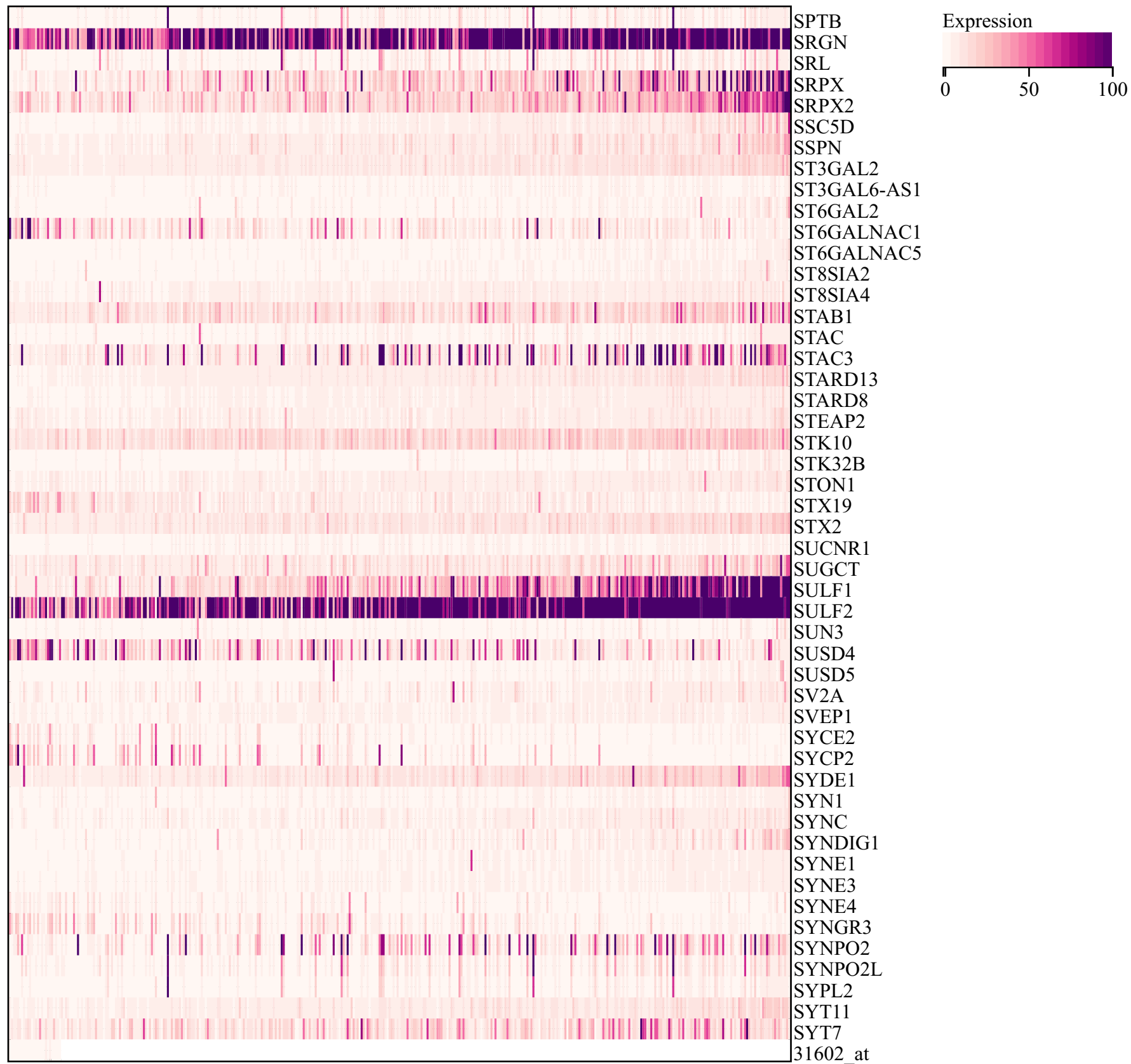

Supplement: Supplementary file 4 [file DataSheet_4.zip › ddee3b587bcbbfe2b8cb0cbb1cabd8db.pdf]

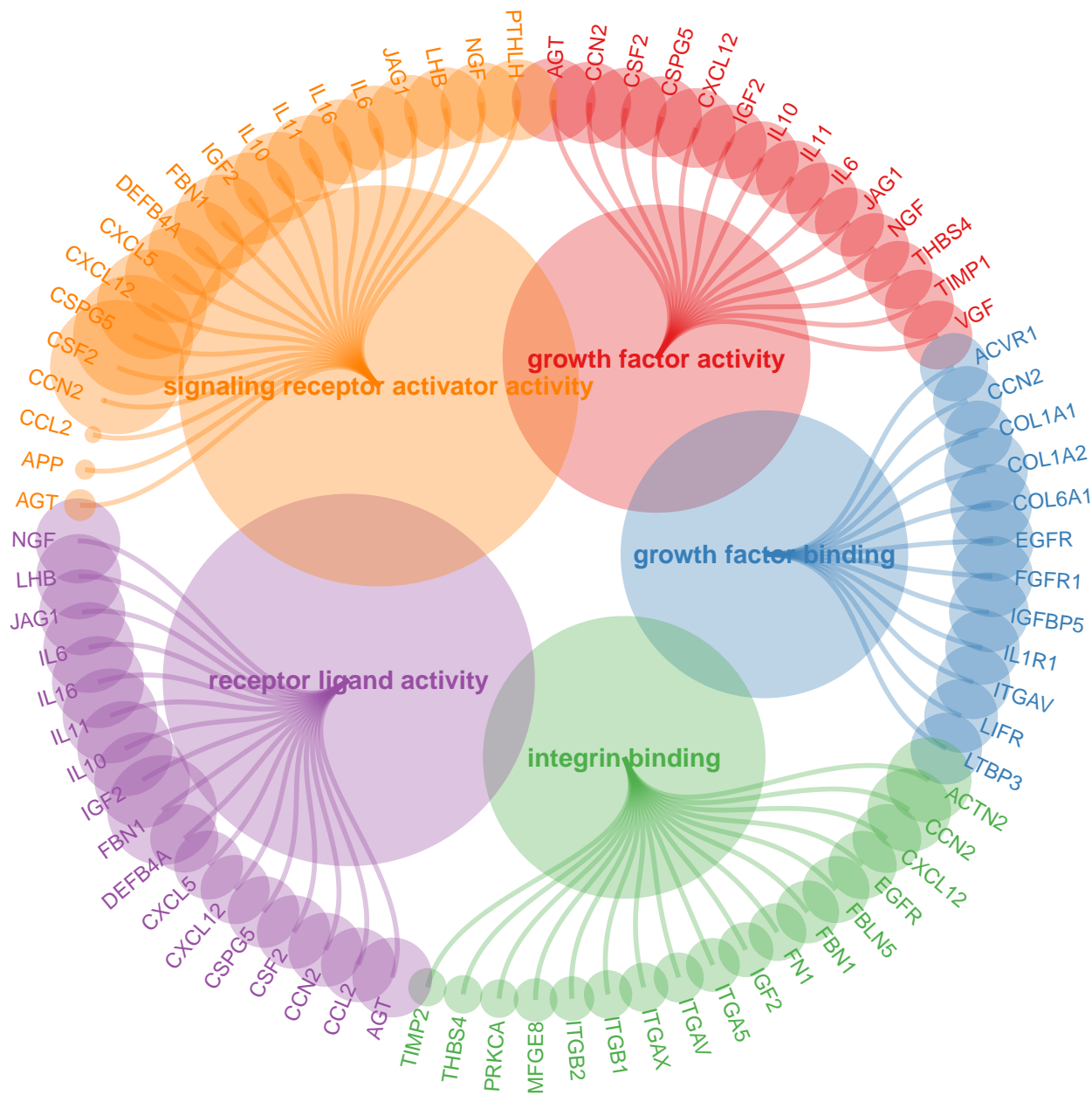

Supplement: Supplementary file 4 [file DataSheet_4.zip › MF.pdf]

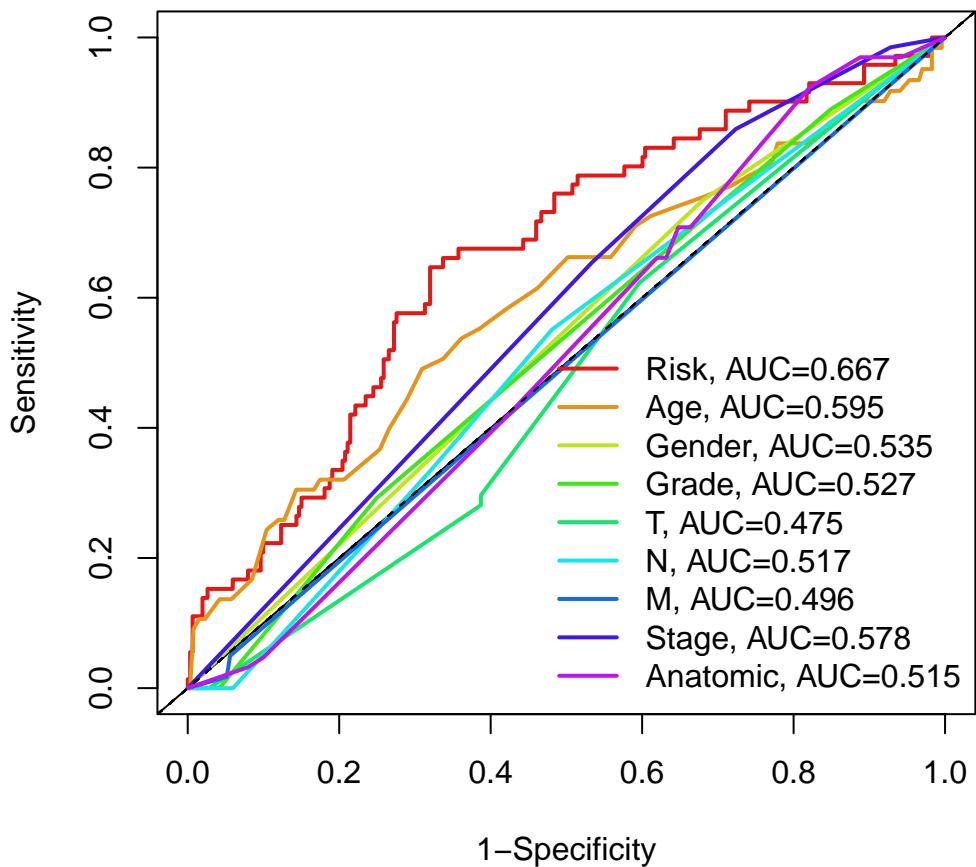

Supplement: Supplementary file 5 [file DataSheet_5.zip › cliROC.pdf]

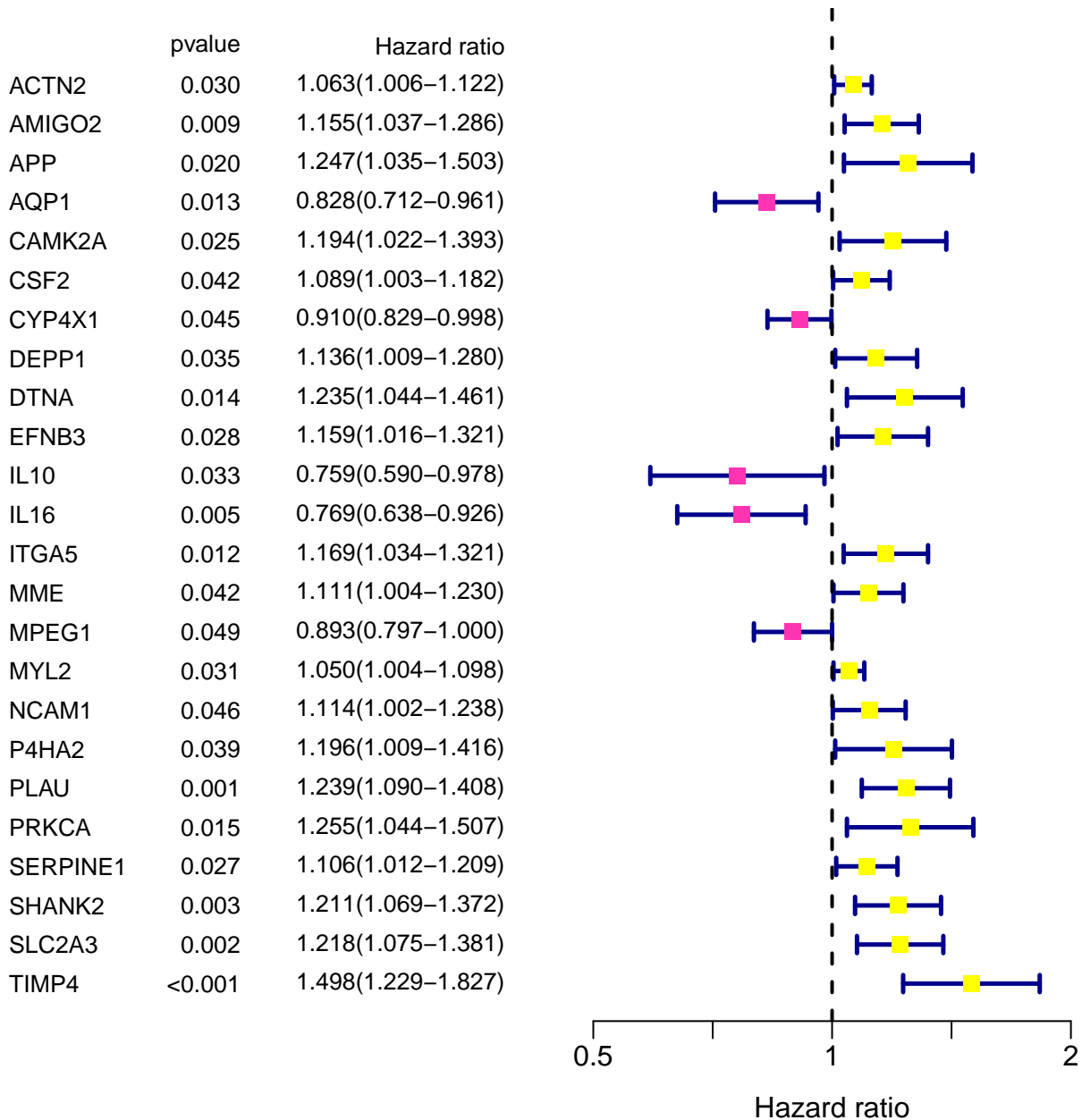

Supplement: Supplementary file 5 [file DataSheet_5.zip › forest.pdf]

Partial Likelihood Deviance

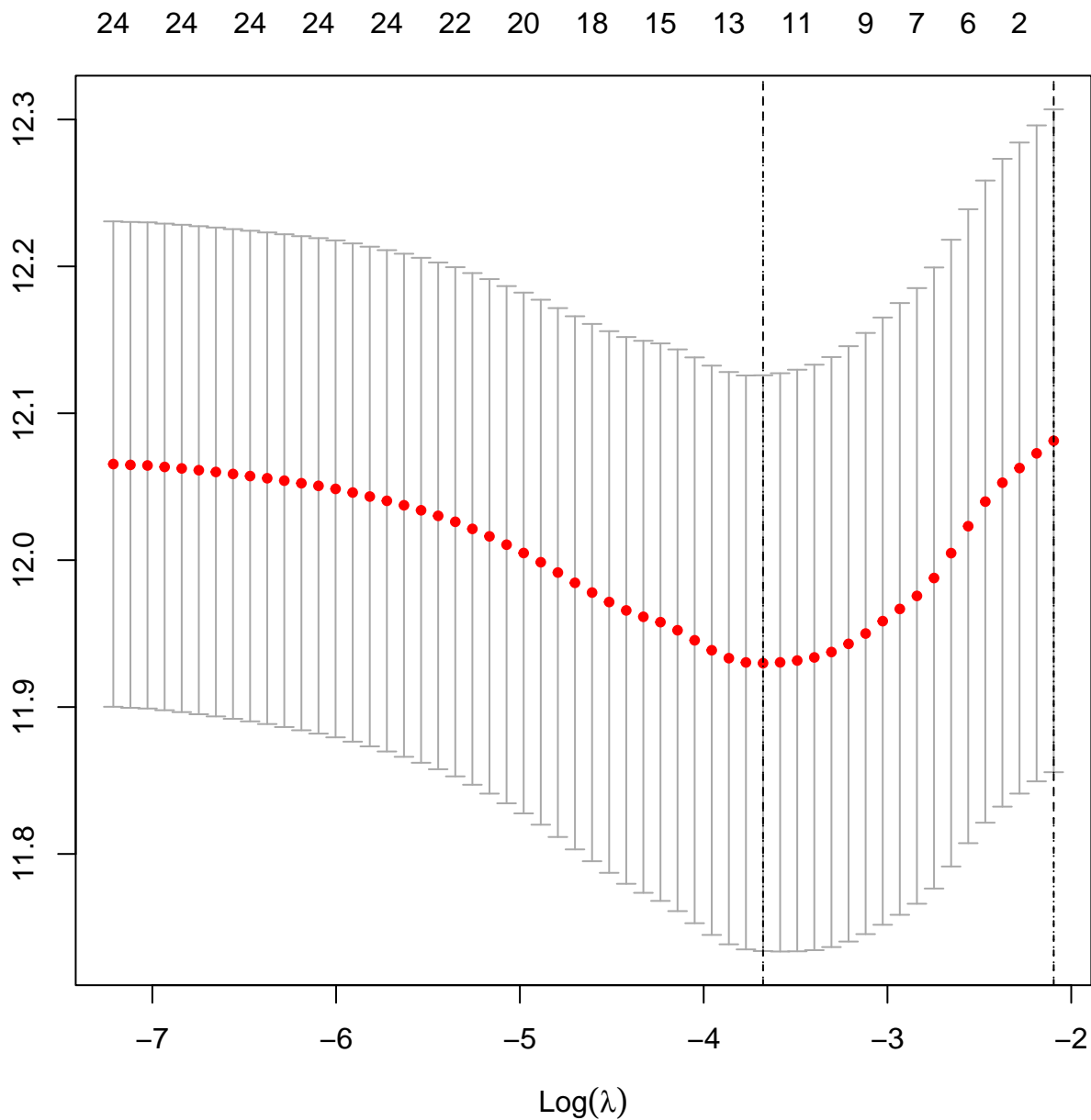

Supplement: Supplementary file 5 [file DataSheet_5.zip › lasso.cvfit.pdf]

Coefficients

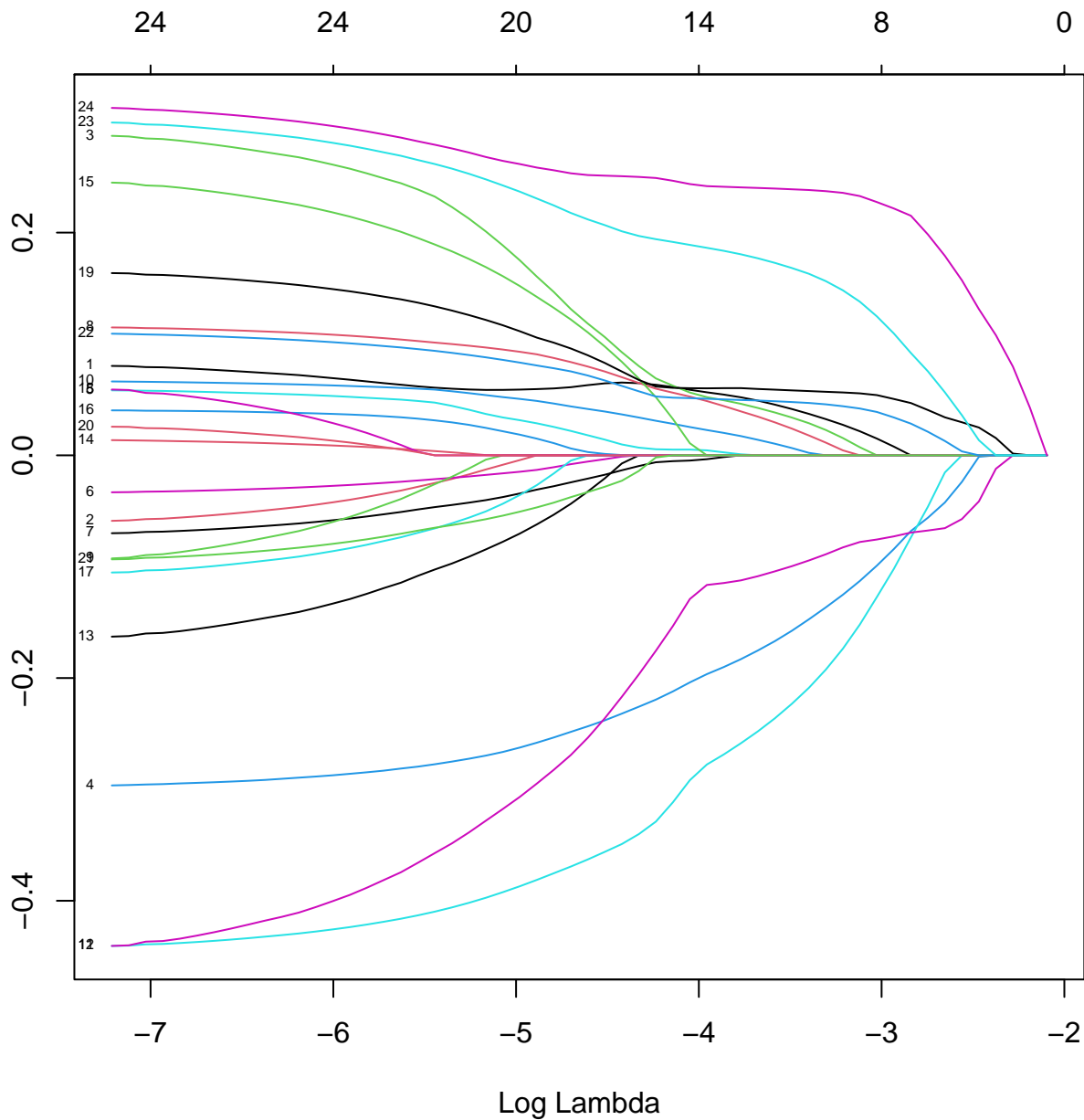

Supplement: Supplementary file 5 [file DataSheet_5.zip › lasso.lambda.pdf]

|           | <b>pvalue</b> | <b>Hazard ratio</b> |
|-----------|---------------|---------------------|
| Age       | <0.001        | 1.031(1.015–1.048)  |
| Gender    | 0.781         | 1.053(0.730–1.520)  |
| Grade     | 0.433         | 1.093(0.875–1.365)  |
| T         | 0.059         | 0.877(0.765–1.005)  |
| N         | 0.810         | 0.977(0.810–1.179)  |
| M         | 0.749         | 0.907(0.497–1.653)  |
| Stage     | <0.001        | 1.700(1.322–2.185)  |
| Anatomic  | 0.801         | 1.008(0.948–1.071)  |
| riskScore | <0.001        | 1.424(1.262–1.607)  |

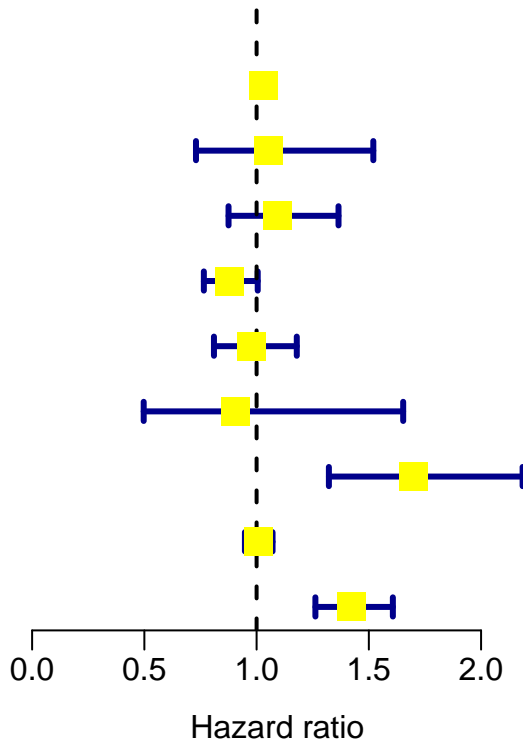

Supplement: Supplementary file 5 [file DataSheet_5.zip › multiForest.pdf]

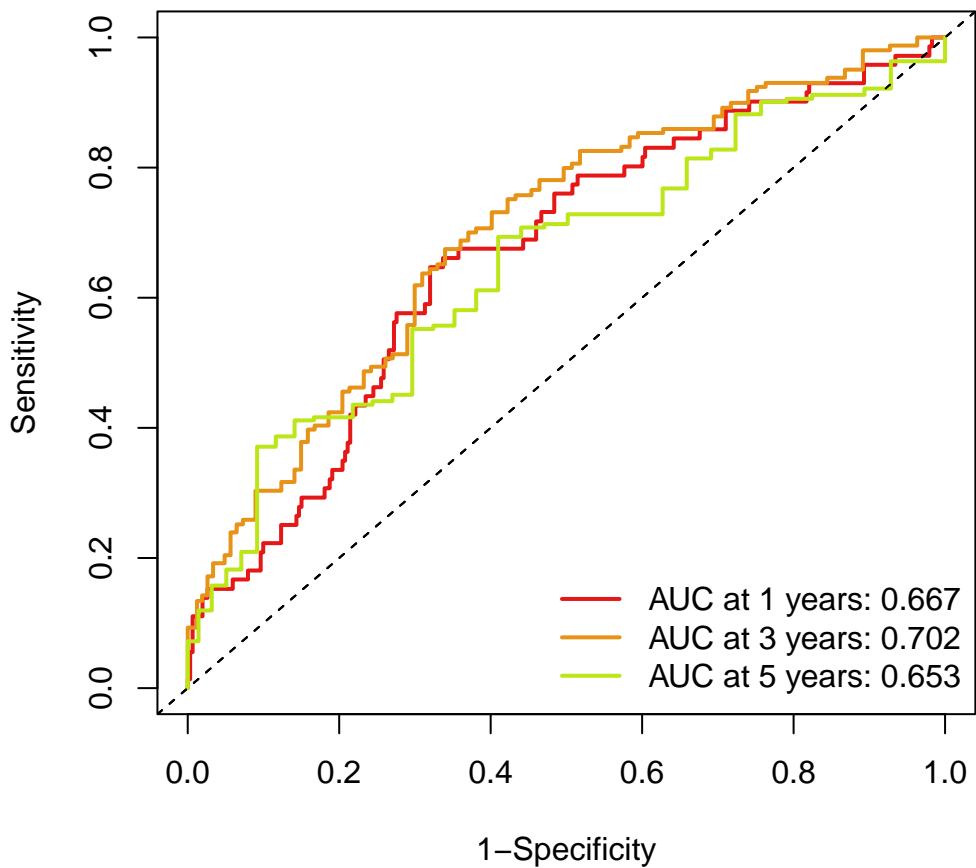

Supplement: Supplementary file 5 [file DataSheet_5.zip › ROC.pdf]

Risk + High risk + Low risk

Survival probability

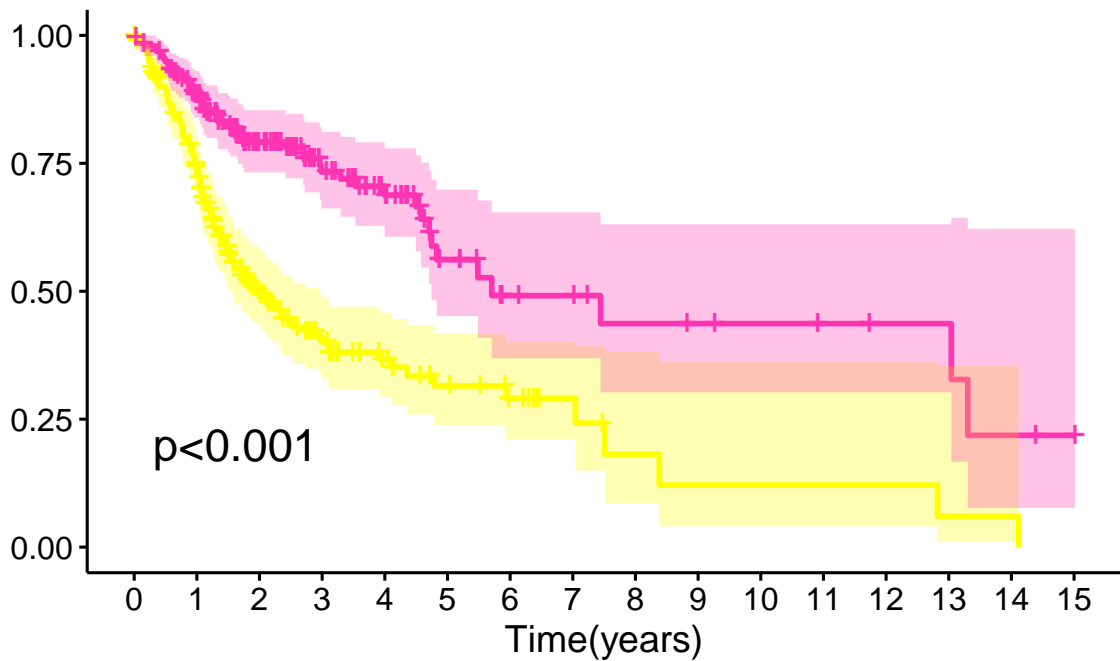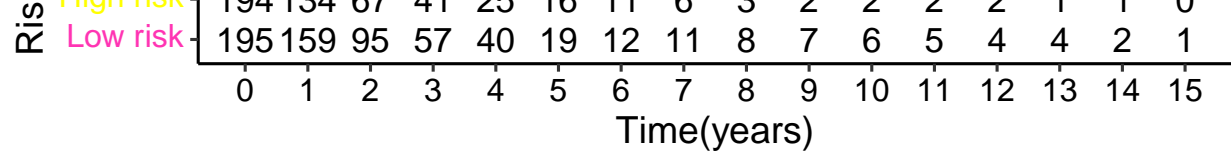

Supplement: Supplementary file 5 [file DataSheet_5.zip › survival.TCGA.pdf]

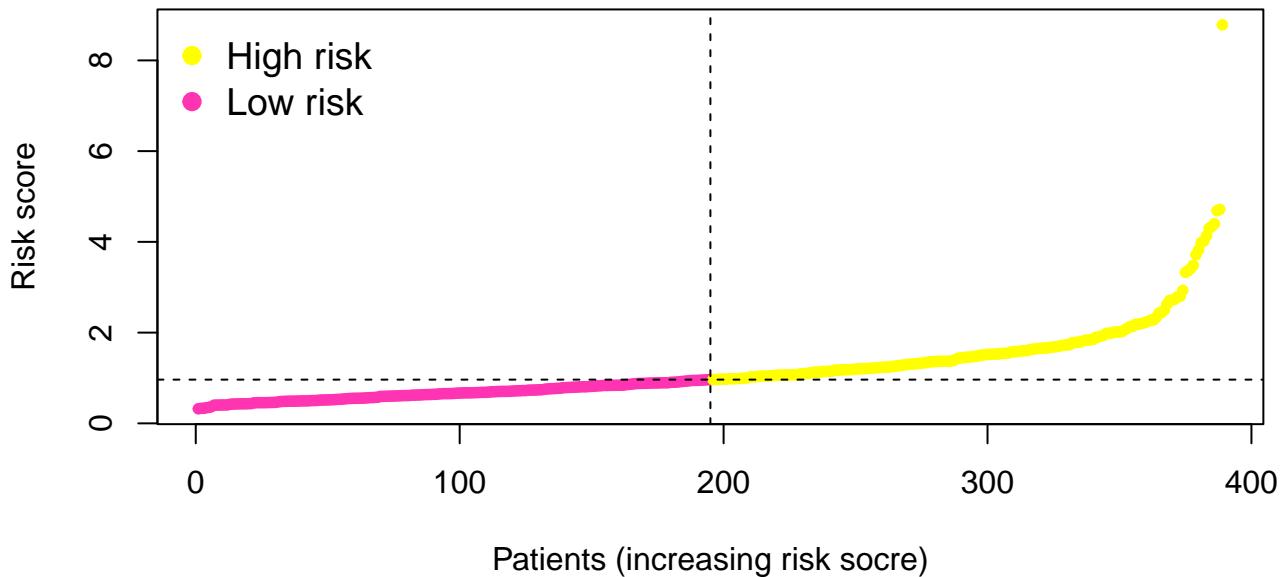

Supplement: Supplementary file 5 [file DataSheet_5.zip › TCGA.riskScore.pdf]

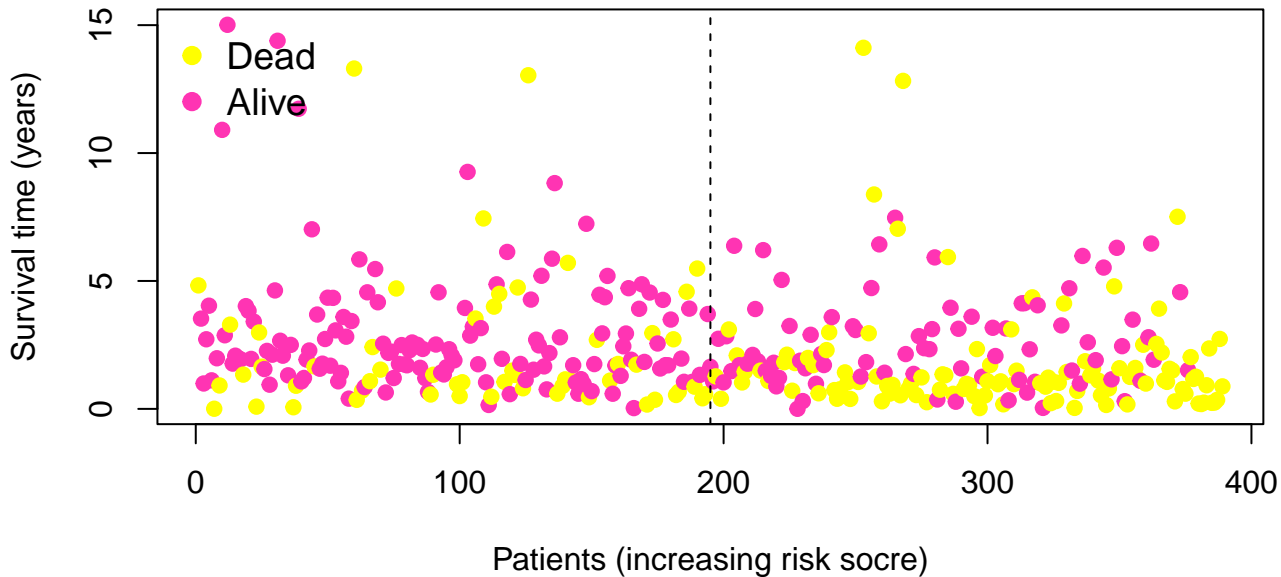

Supplement: Supplementary file 5 [file DataSheet_5.zip › TCGA.survStat.pdf]

|           | <b>pvalue</b> | <b>Hazard ratio</b> |
|-----------|---------------|---------------------|
| Age       | 0.001         | 1.023(1.009–1.037)  |
| Gender    | 0.527         | 0.896(0.636–1.261)  |
| Grade     | 0.288         | 1.109(0.917–1.341)  |
| T         | 0.296         | 1.060(0.950–1.183)  |
| N         | 0.351         | 1.076(0.923–1.254)  |
| M         | 0.318         | 0.743(0.414–1.331)  |
| Stage     | <0.001        | 1.529(1.253–1.867)  |
| Anatomic  | 0.493         | 0.981(0.928–1.037)  |
| riskScore | <0.001        | 1.506(1.343–1.689)  |

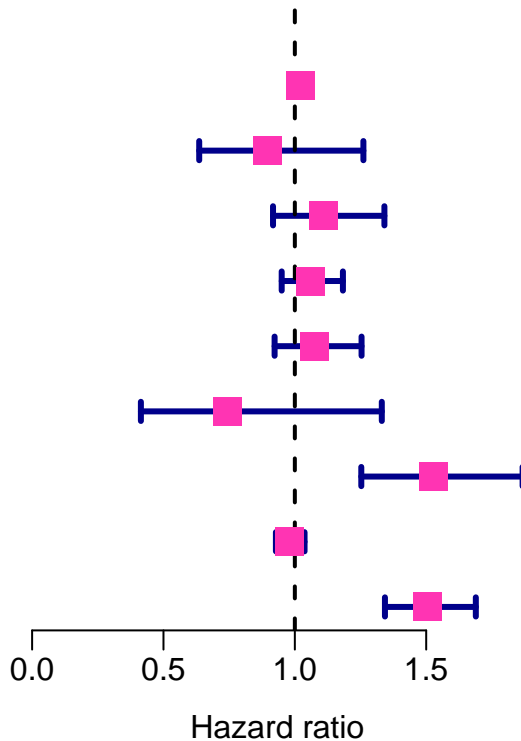

Supplement: Supplementary file 5 [file DataSheet_5.zip › uniForest.pdf]

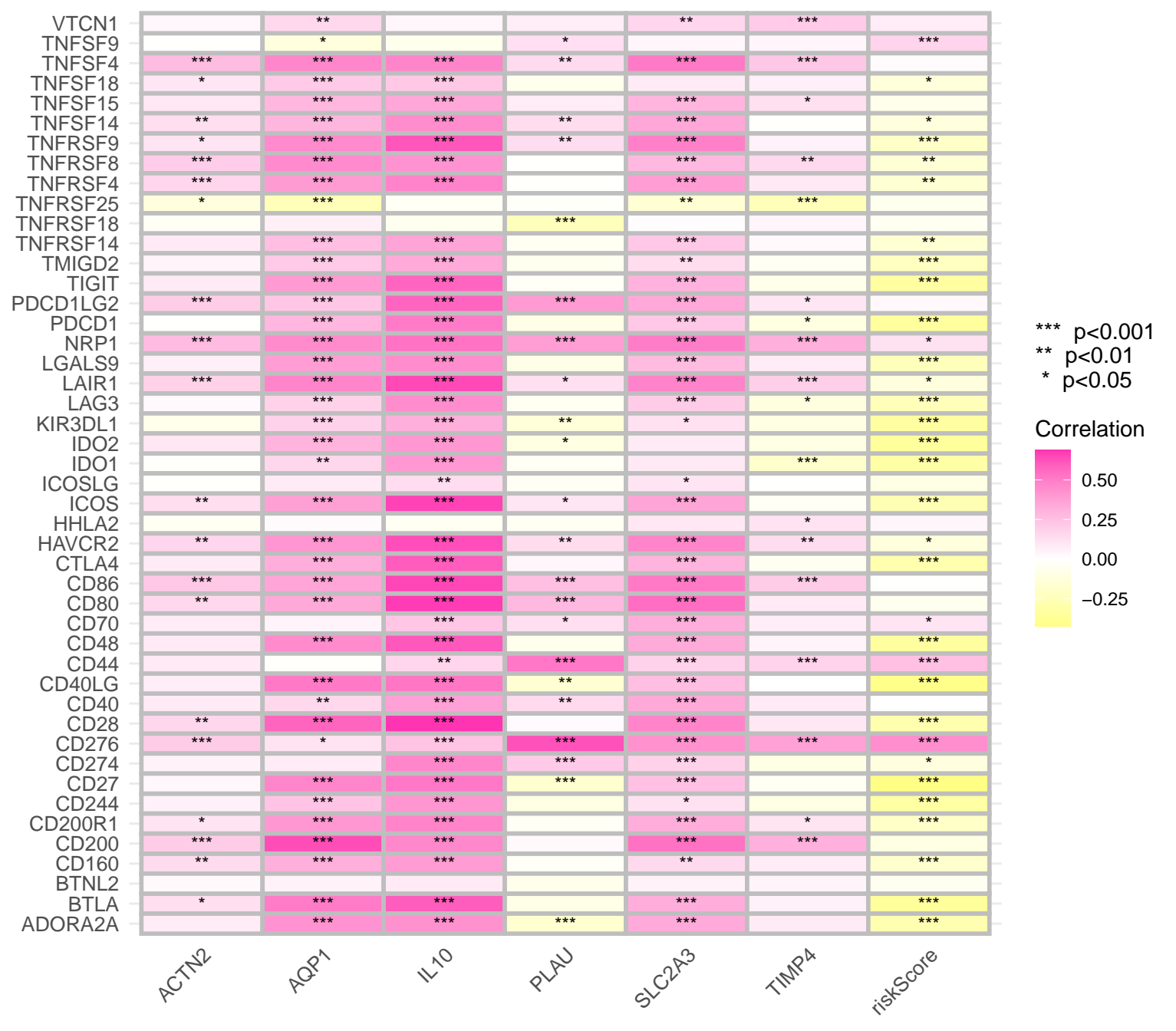

Supplement: Supplementary file 6 [file DataSheet_6.zip › checkpointCor.pdf]

B cell memory\_CIBERSORT

$R = -0.1, p = 0.04$

0.15

0.10

0.05

0.00

1

3

4

Risk score

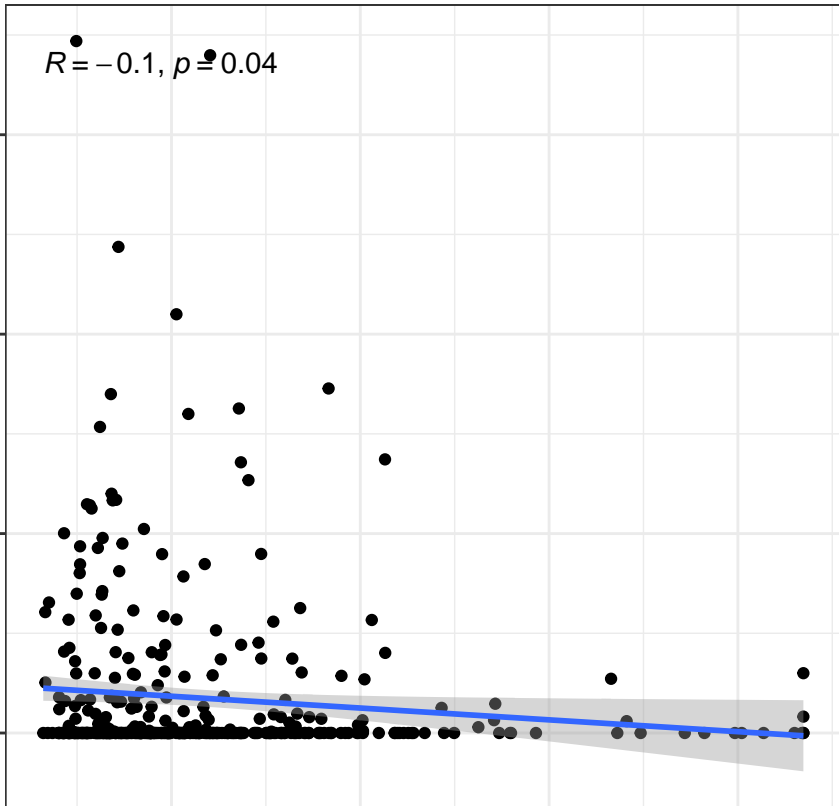

Supplement: Supplementary file 6 [file DataSheet_6.zip › cor.B cell memory_CIBERSORT.pdf]

B cell memory\_CIBERSORT-ABS

$R = -0.12, p = 0.023$

0.2

0.1

0.0

1

Risk score

2

3

4

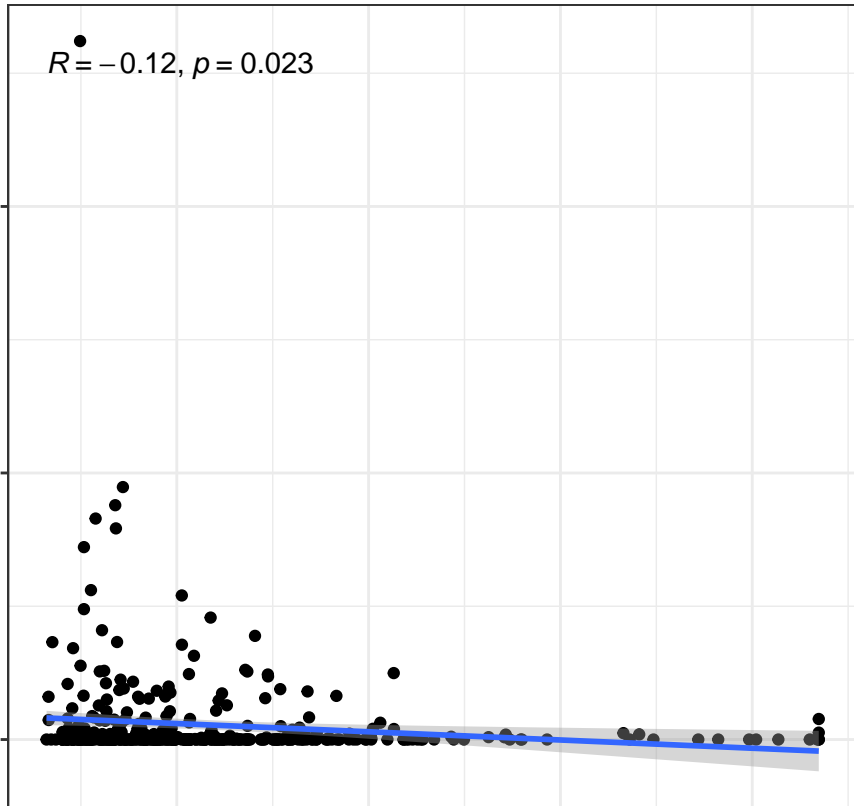

Supplement: Supplementary file 6 [file DataSheet_6.zip › cor.B cell memory_CIBERSORT-ABS.pdf]

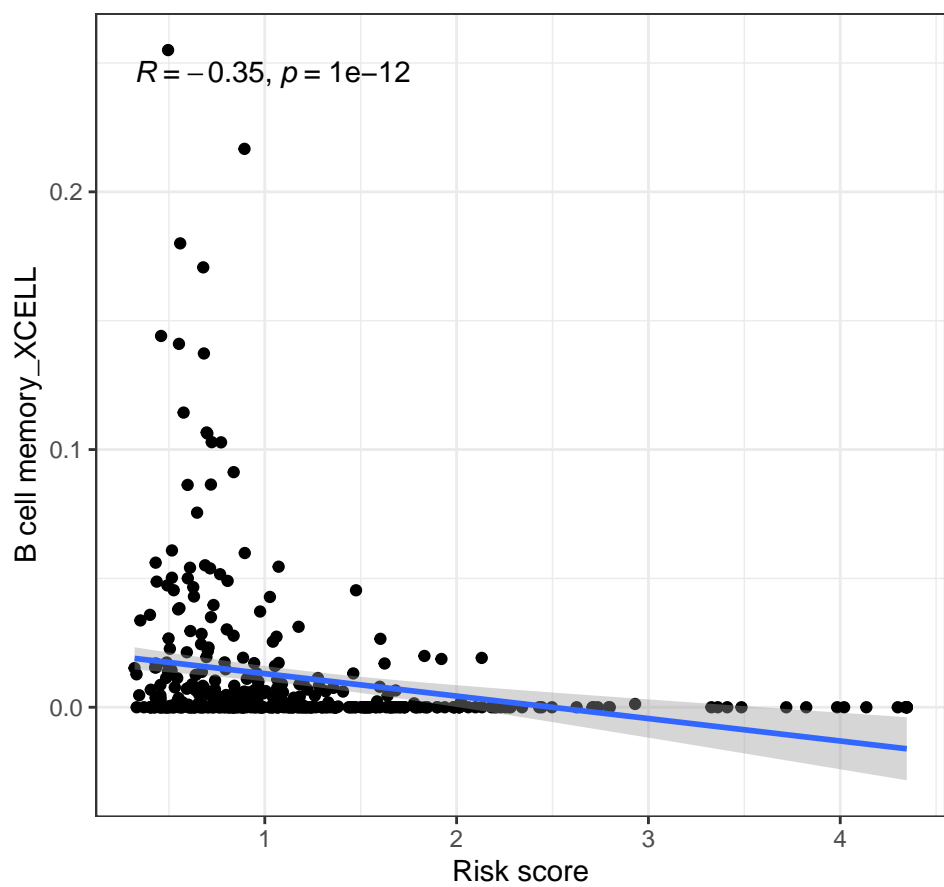

Supplement: Supplementary file 6 [file DataSheet_6.zip › cor.B cell memory_XCELL.pdf]

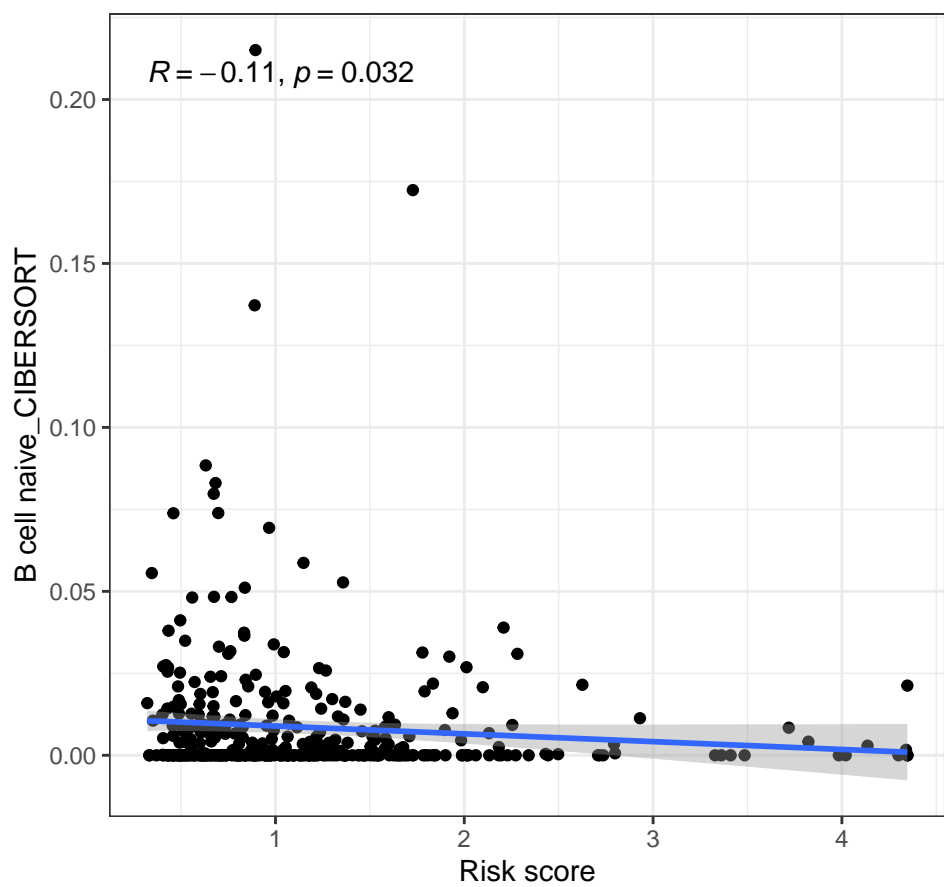

Supplement: Supplementary file 6 [file DataSheet_6.zip › cor.B cell naive_CIBERSORT.pdf]

B cell naive\_CIBERSORT-ABS

$R = -0.13, p = 0.011$

0.20

0.15

0.10

0.05

0.00

1

Risk score

3

4

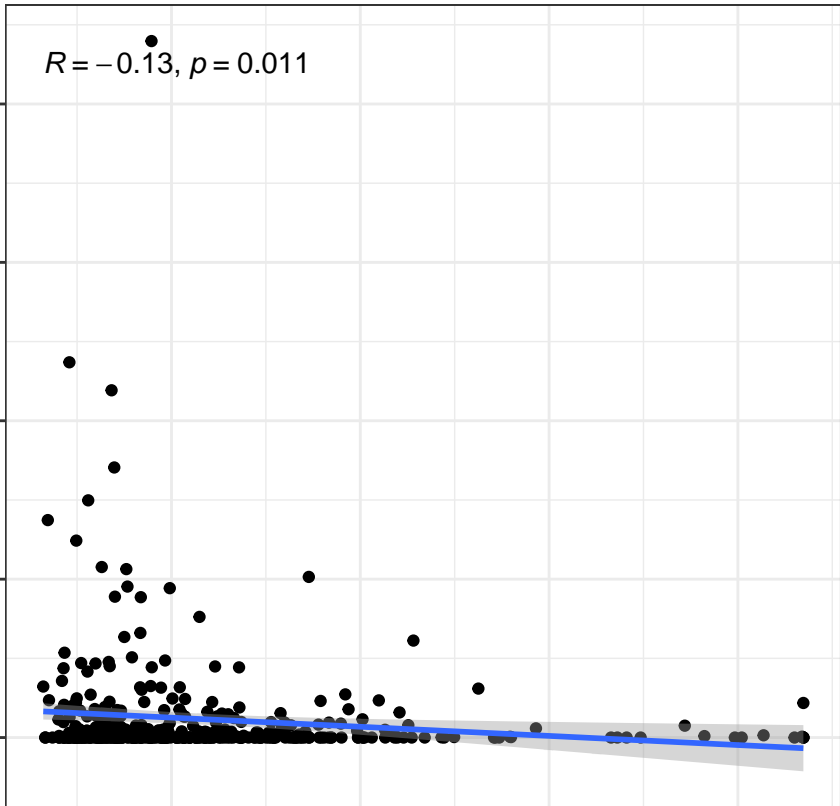

Supplement: Supplementary file 6 [file DataSheet_6.zip › cor.B cell naive_CIBERSORT-ABS.pdf]

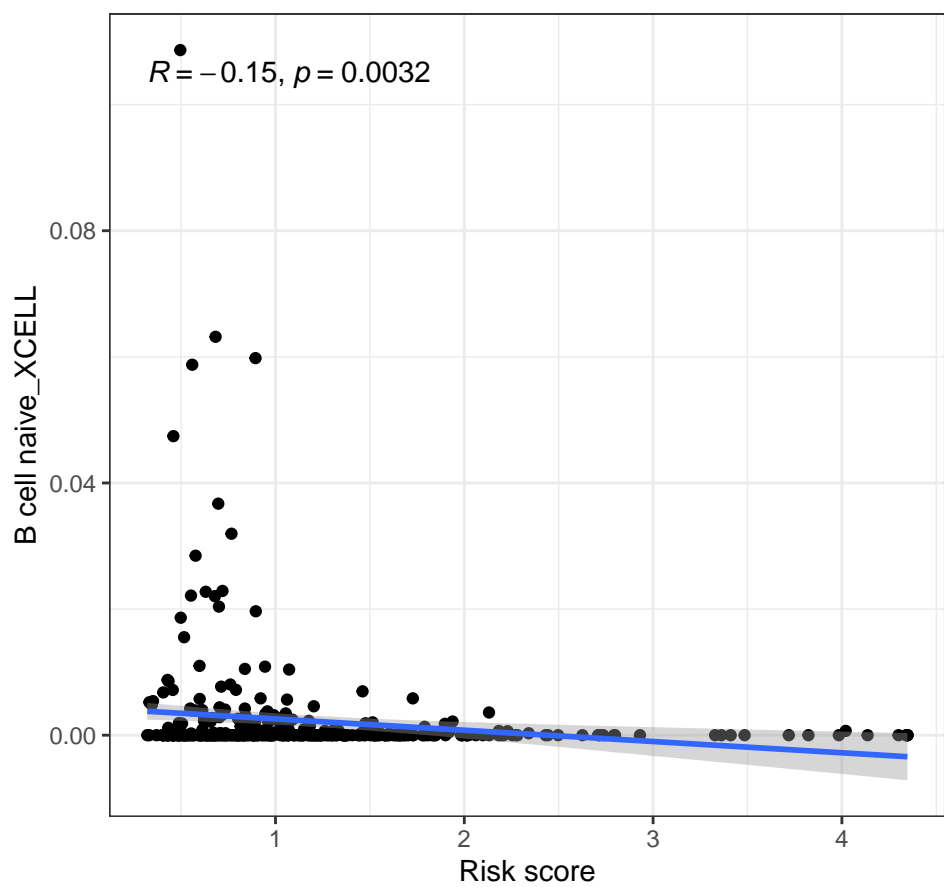

Supplement: Supplementary file 6 [file DataSheet_6.zip › cor.B cell naive_XCELL.pdf]

B cell plasma\_CIBERSORT-ABS

$R = -0.15, p = 0.0029$

0.20  
0.15  
0.10  
0.05  
0.00

Risk score

3

4

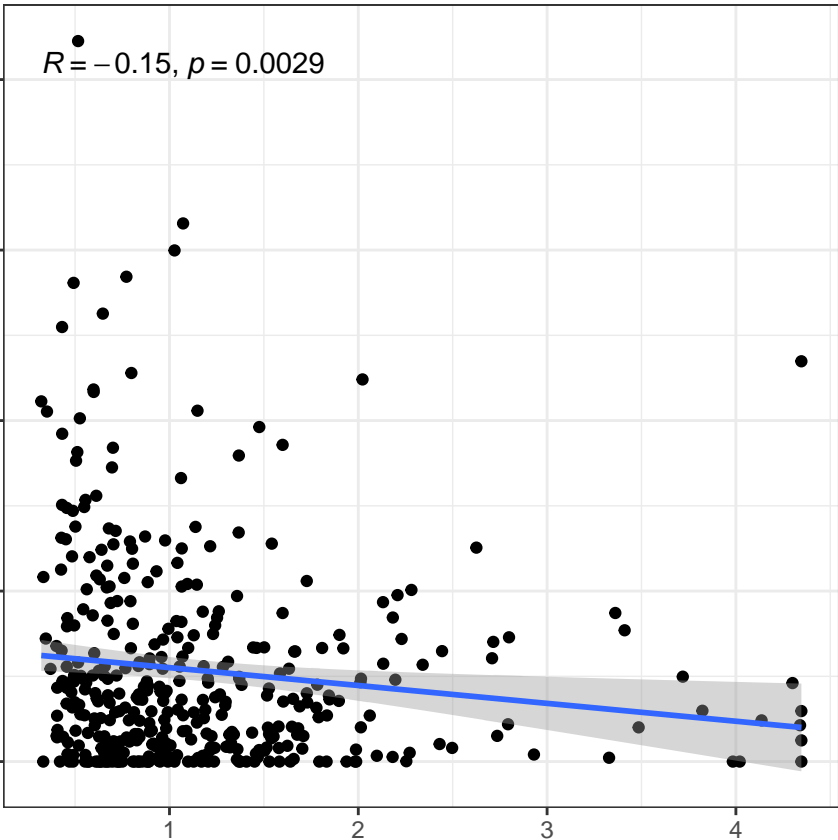

Supplement: Supplementary file 6 [file DataSheet_6.zip › cor.B cell plasma_CIBERSORT-ABS.pdf]

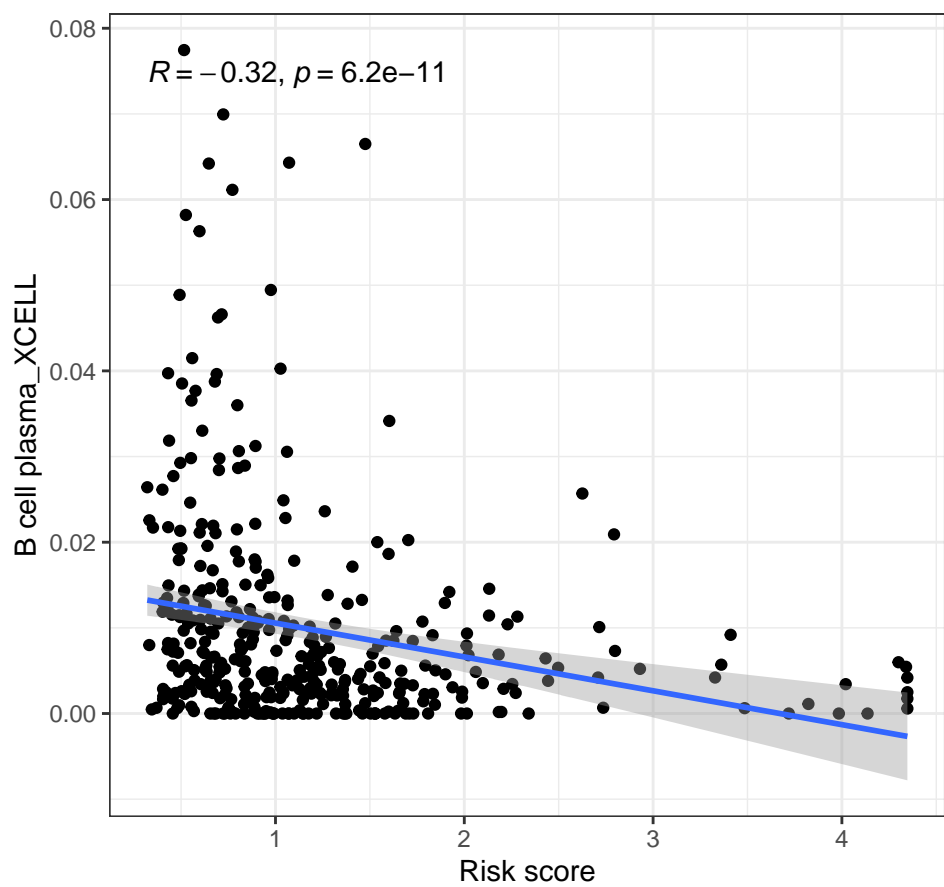

Supplement: Supplementary file 6 [file DataSheet_6.zip › cor.B cell plasma_XCELL.pdf]

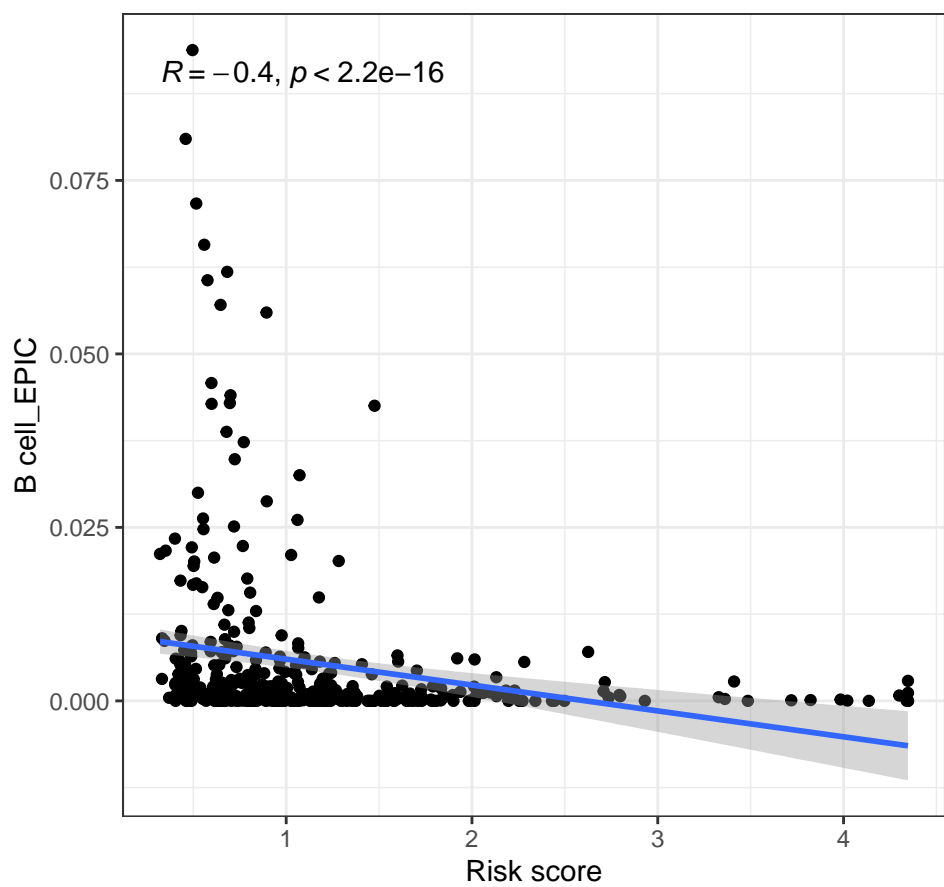

Supplement: Supplementary file 6 [file DataSheet_6.zip › cor.B cell_EPIC.pdf]

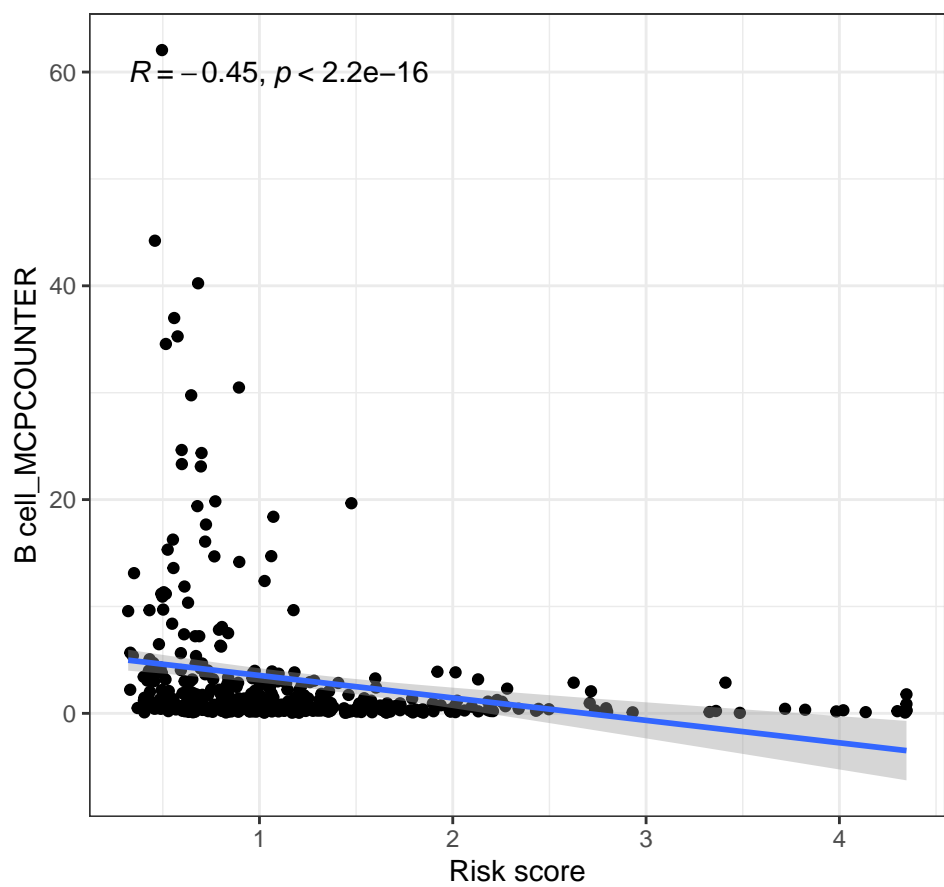

Supplement: Supplementary file 6 [file DataSheet_6.zip › cor.B cell_MCPCOUNTER.pdf]

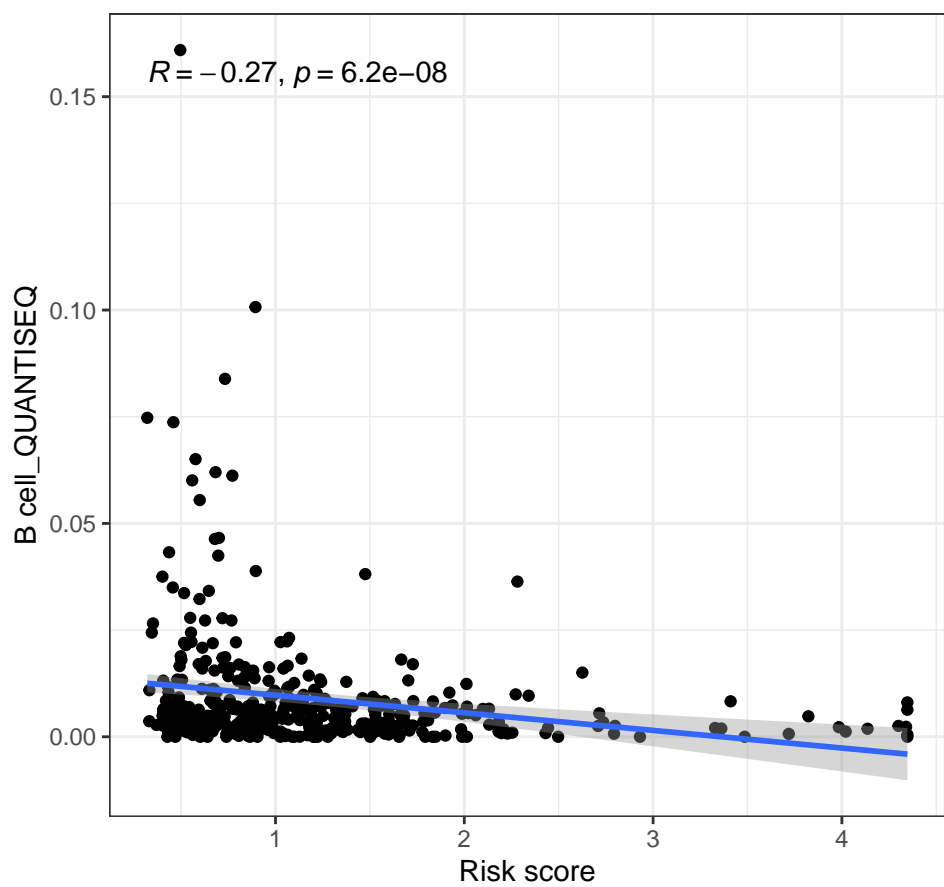

Supplement: Supplementary file 6 [file DataSheet_6.zip › cor.B cell_QUANTISEQ.pdf]

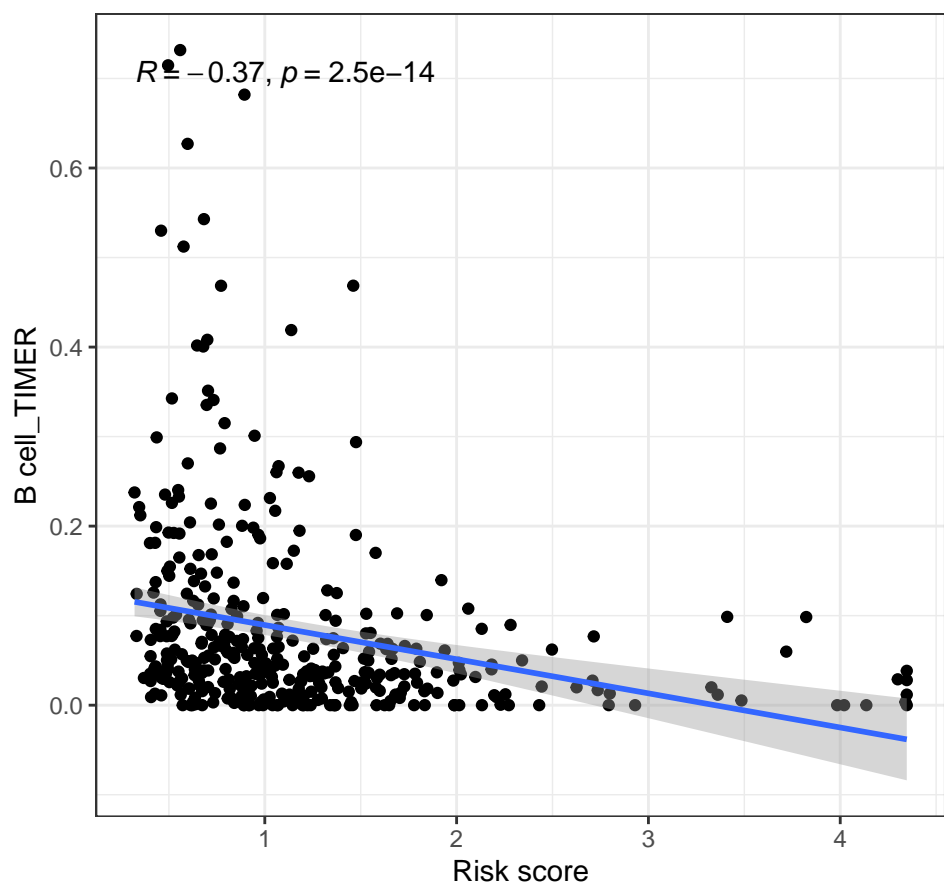

Supplement: Supplementary file 6 [file DataSheet_6.zip › cor.B cell_TIMER.pdf]

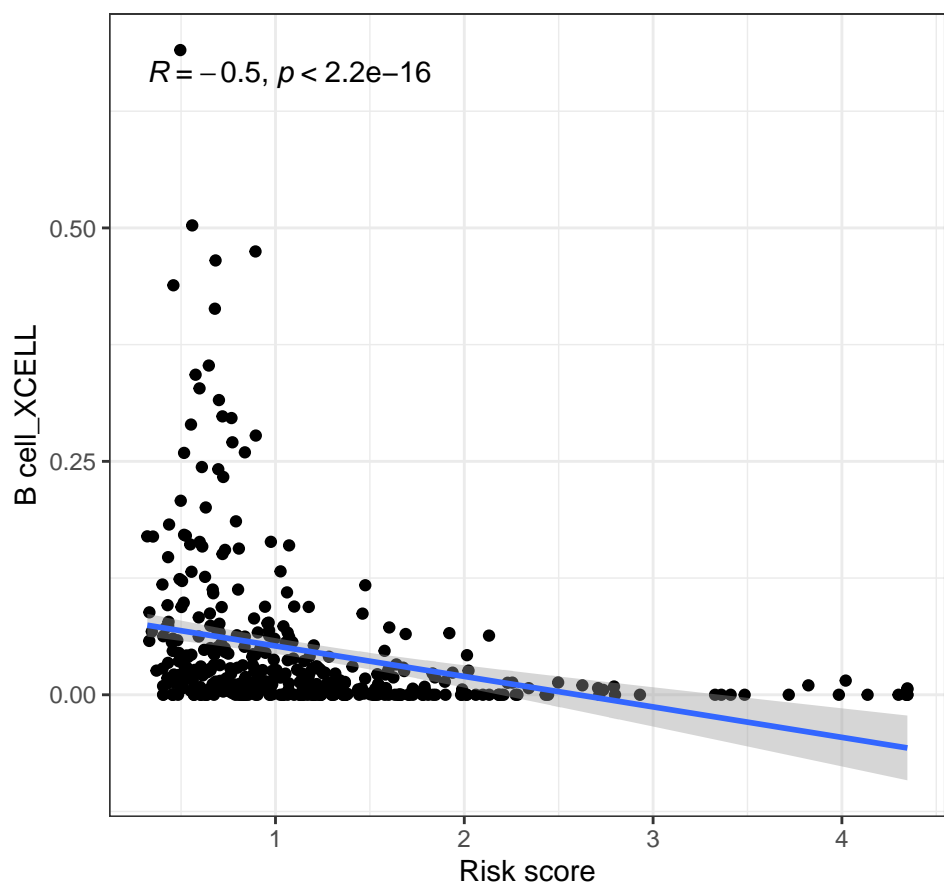

Supplement: Supplementary file 6 [file DataSheet_6.zip › cor.B cell_XCELL.pdf]

Cancer associated fibroblast\_EPIC

$R = 0.15, p = 0.004$

1.00  
0.75  
0.50  
0.25  
0.00

Risk score

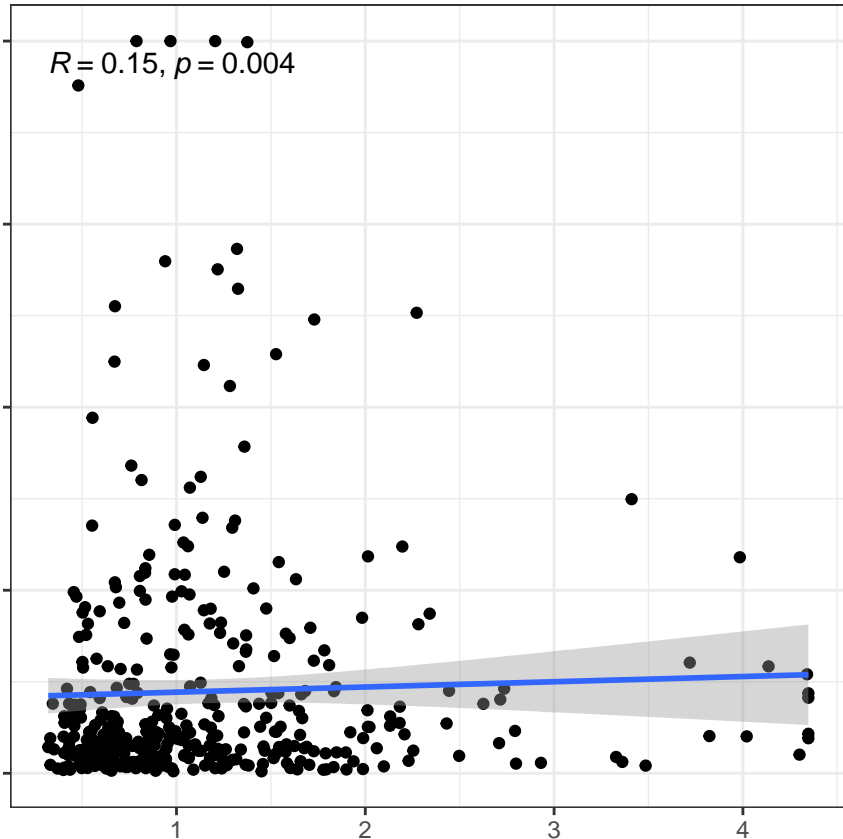

Supplement: Supplementary file 6 [file DataSheet_6.zip › cor.Cancer associated fibroblast_EPIC.pdf]

Cancer associated fibroblast\_MCP\_COUNTER

$R = 0.17, p = 0.00053$

6000

4000

2000

0

1

Risk score

3

4

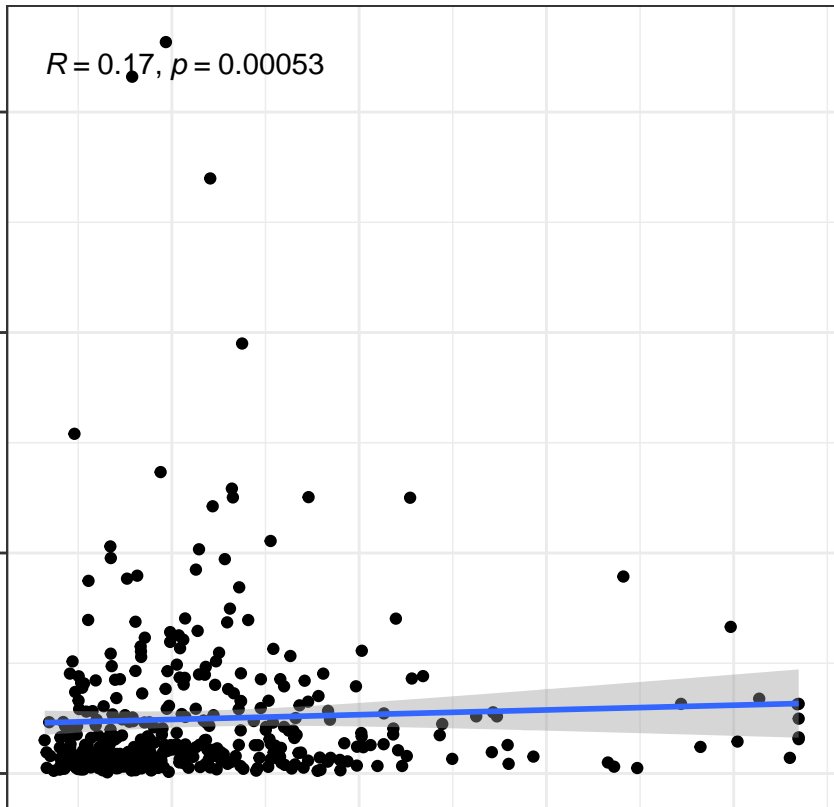

Supplement: Supplementary file 6 [file DataSheet_6.zip › cor.Cancer associated fibroblast_MCPCOUNTER.pdf]

Class-switched memory B cell\_XCELL

$R = -0.43, p < 2.2e-16$

0.00

0.05

0.10

0.15

1

Risk score

3

4

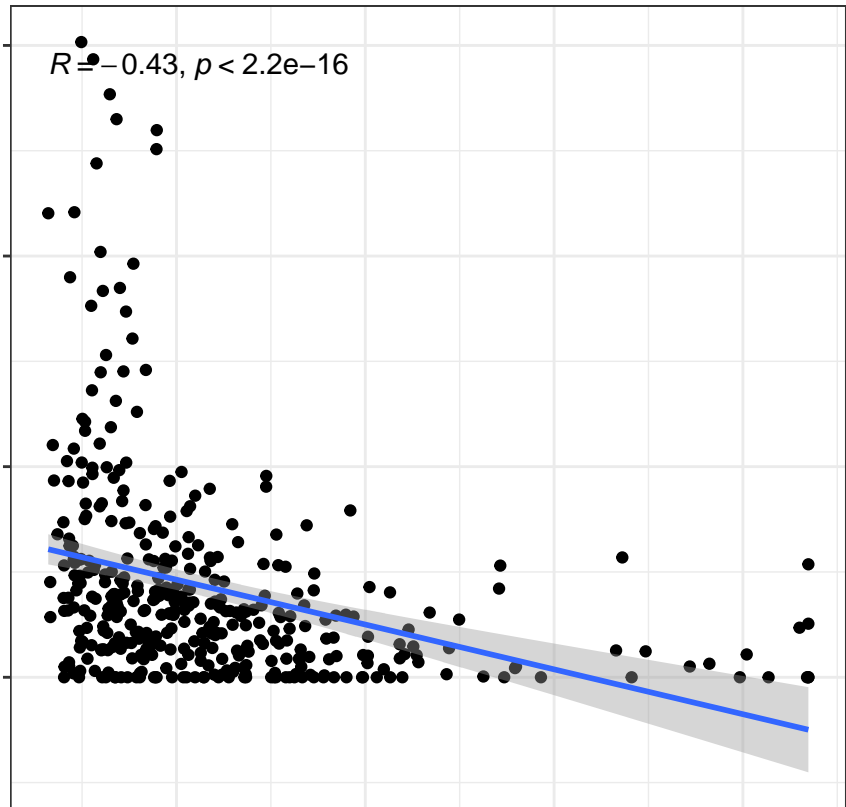

Supplement: Supplementary file 6 [file DataSheet_6.zip › cor.Class-switched memory B cell_XCELL.pdf]

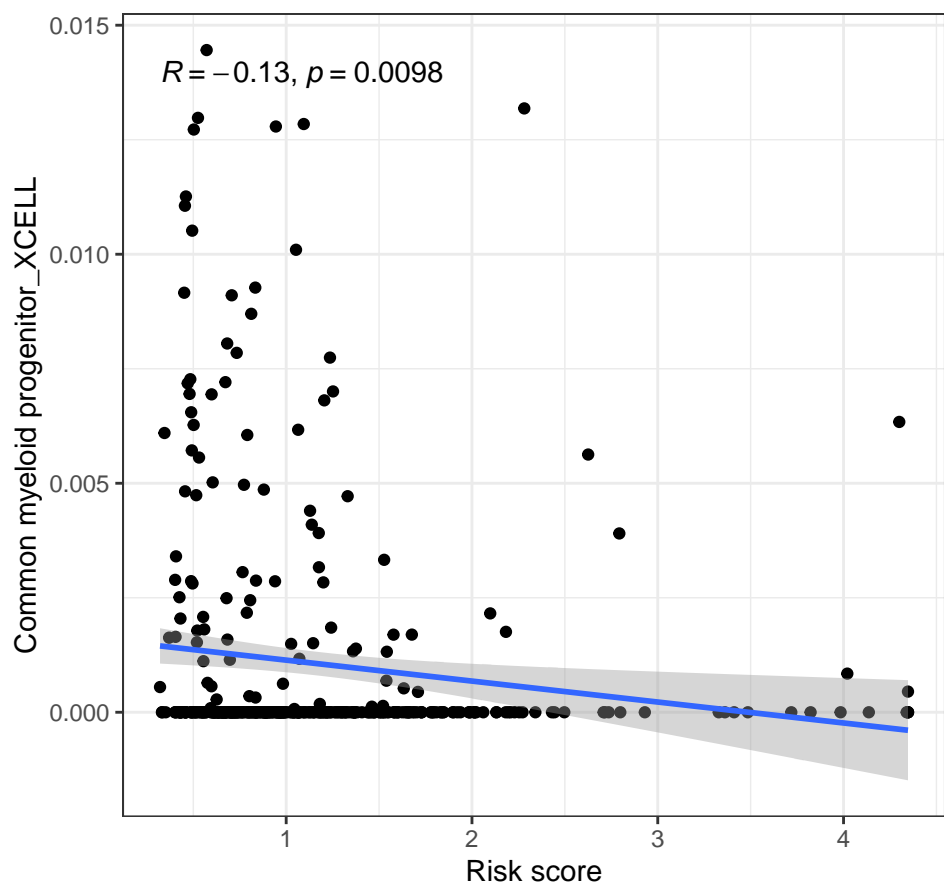

Supplement: Supplementary file 6 [file DataSheet_6.zip › cor.Common myeloid progenitor_XCELL.pdf]

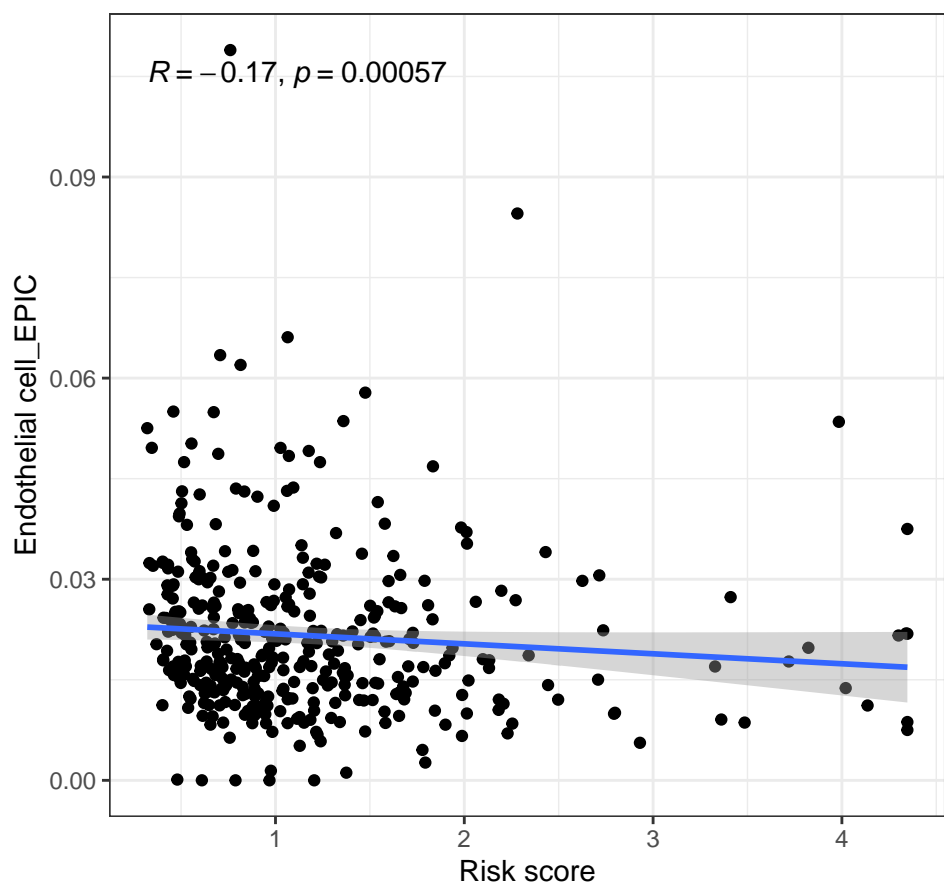

Supplement: Supplementary file 6 [file DataSheet_6.zip › cor.Endothelial cell_EPIC.pdf]

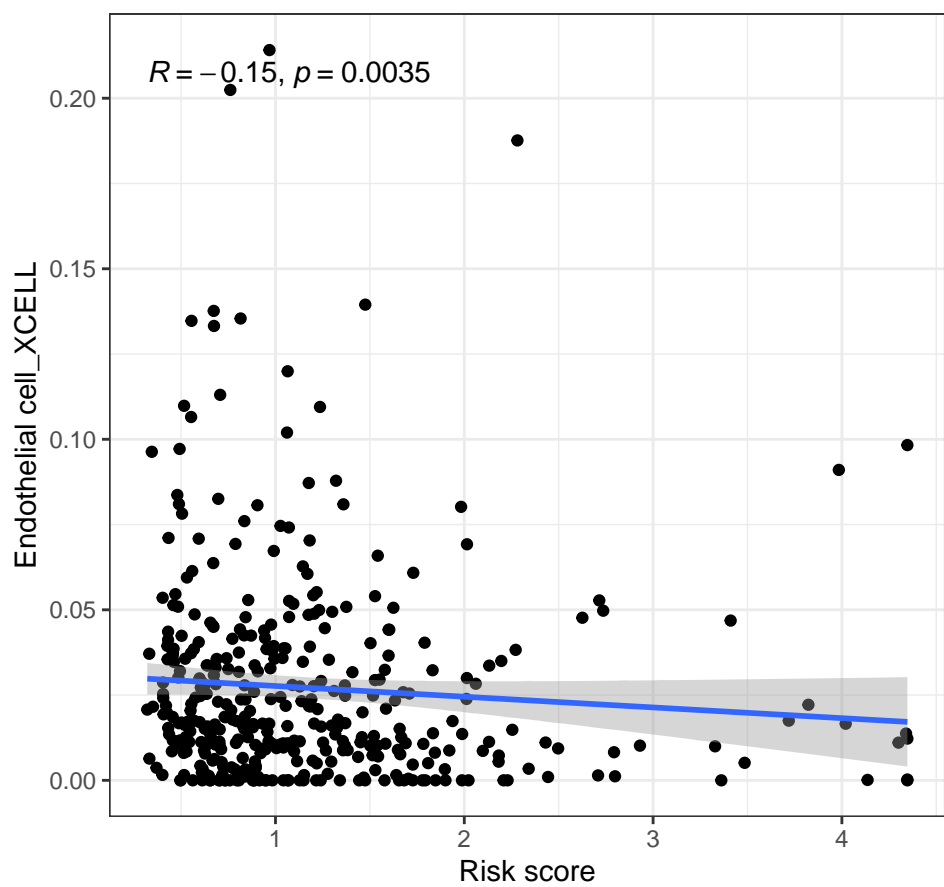

Supplement: Supplementary file 6 [file DataSheet_6.zip › cor.Endothelial cell_XCELL.pdf]

Hematopoietic stem cell\_XCELL

$R = -0.18, p = 0.00035$

0.3  
0.2  
0.1  
0.0

Risk score

1

3

4

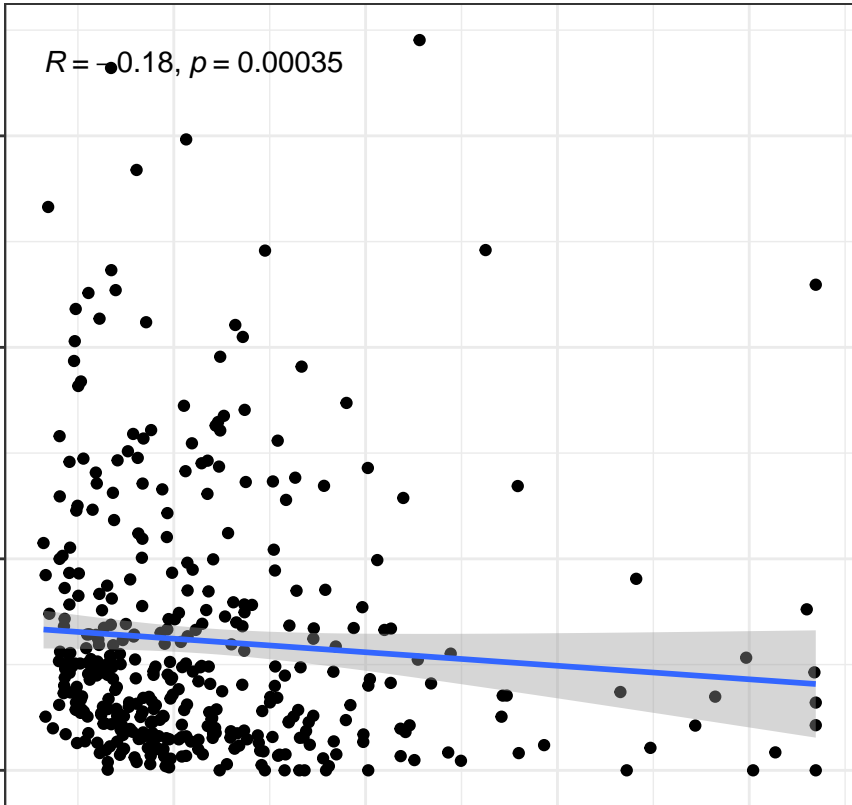

Supplement: Supplementary file 6 [file DataSheet_6.zip › cor.Hematopoietic stem cell_XCELL.pdf]

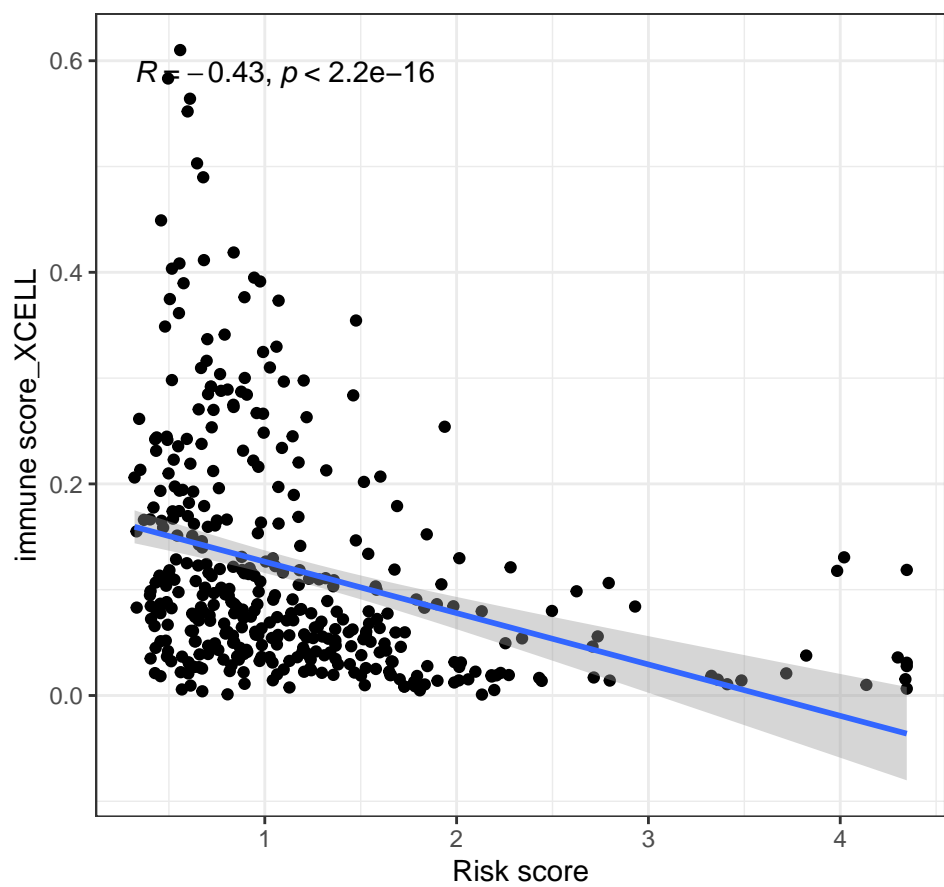

Supplement: Supplementary file 6 [file DataSheet_6.zip › cor.immune score_XCELL.pdf]

Macrophage M0\_CIBERSORT

$R = 0.34, p = 1.1\text{e-}11$

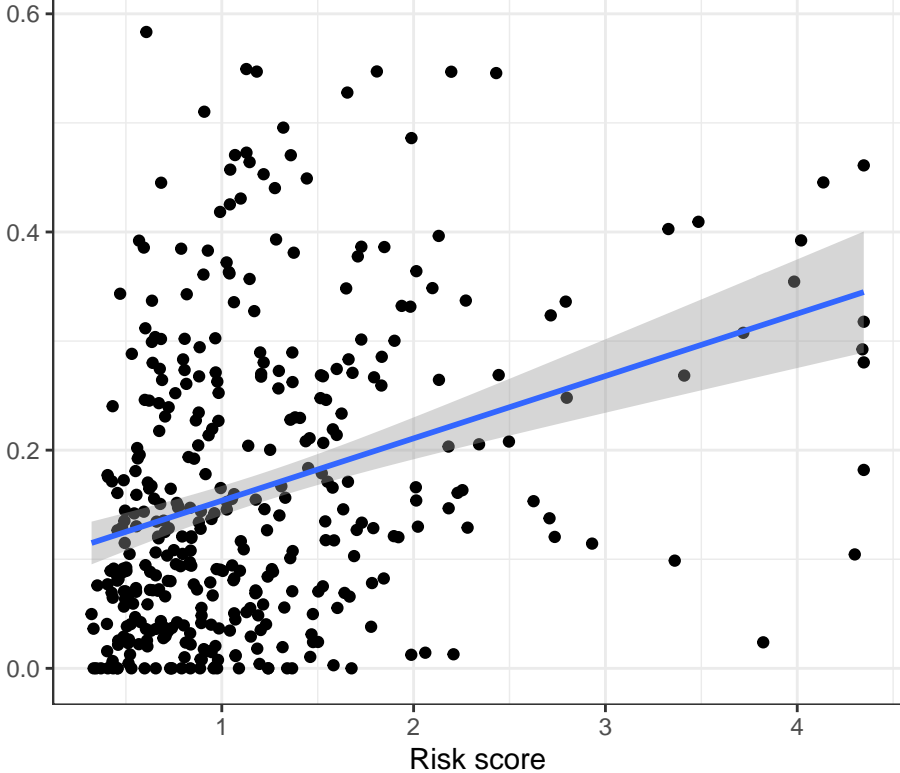

Supplement: Supplementary file 6 [file DataSheet_6.zip › cor.Macrophage M0_CIBERSORT.pdf]

Macrophage M0\_CIBERSORT-ABS

$R = 0.16, p = 0.0014$

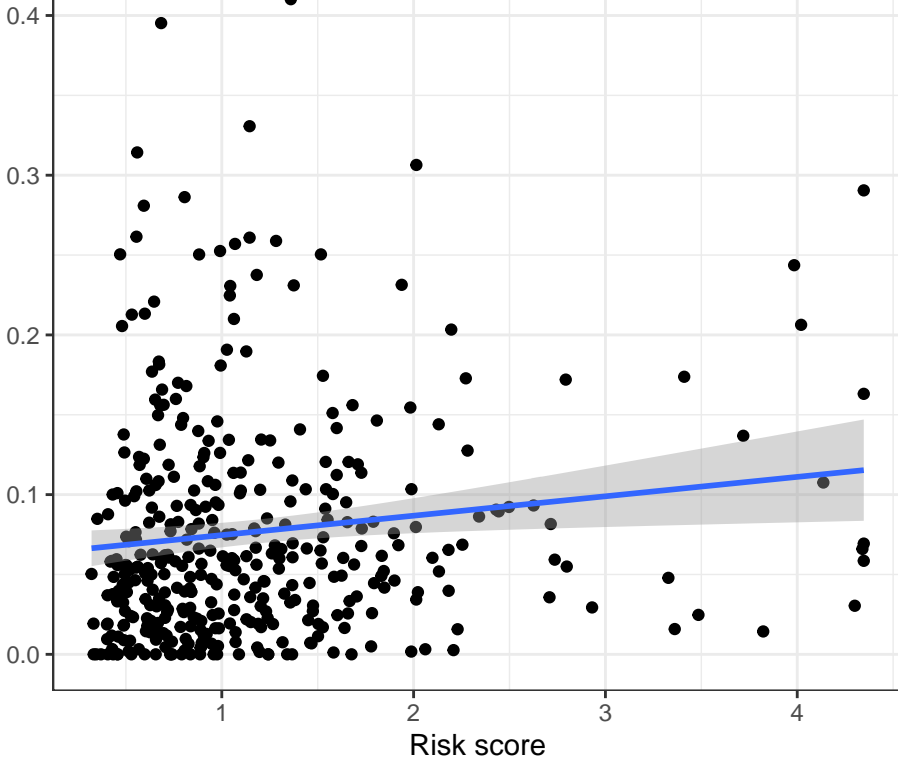

Supplement: Supplementary file 6 [file DataSheet_6.zip › cor.Macrophage M0_CIBERSORT-ABS.pdf]

Macrophage M1\_CIBERSORT

$R = -0.23$ ,  $p = 3.3e-06$

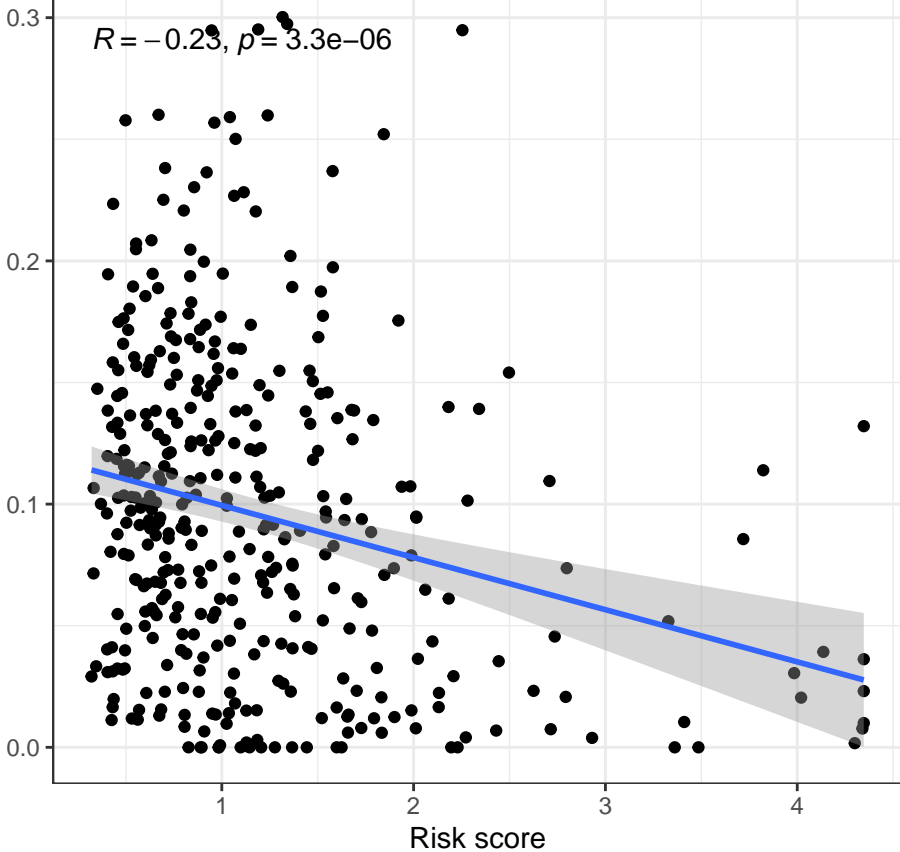

Supplement: Supplementary file 6 [file DataSheet_6.zip › cor.Macrophage M1_CIBERSORT.pdf]

Macrophage M1\_CIBERSORT-ABS

$R = -0.3, p = 8.8e-10$

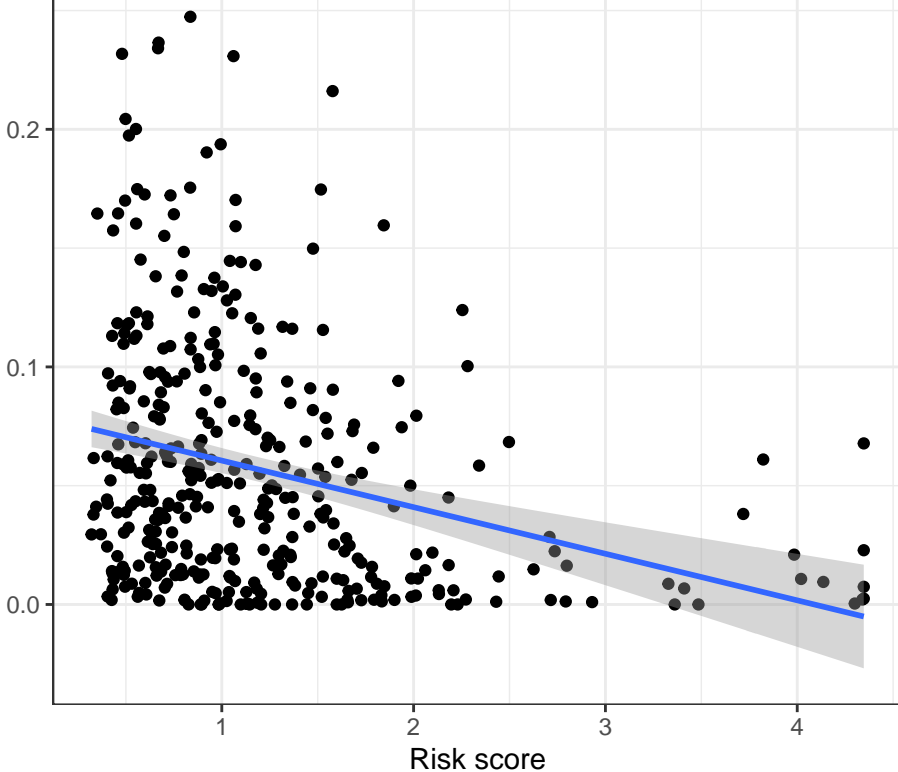

Supplement: Supplementary file 6 [file DataSheet_6.zip › cor.Macrophage M1_CIBERSORT-ABS.pdf]

Macrophage M1\_QUANTISEQ

$R = 0.16, p = 0.0014$

0.20  
0.15  
0.10  
0.05  
0.00

Risk score

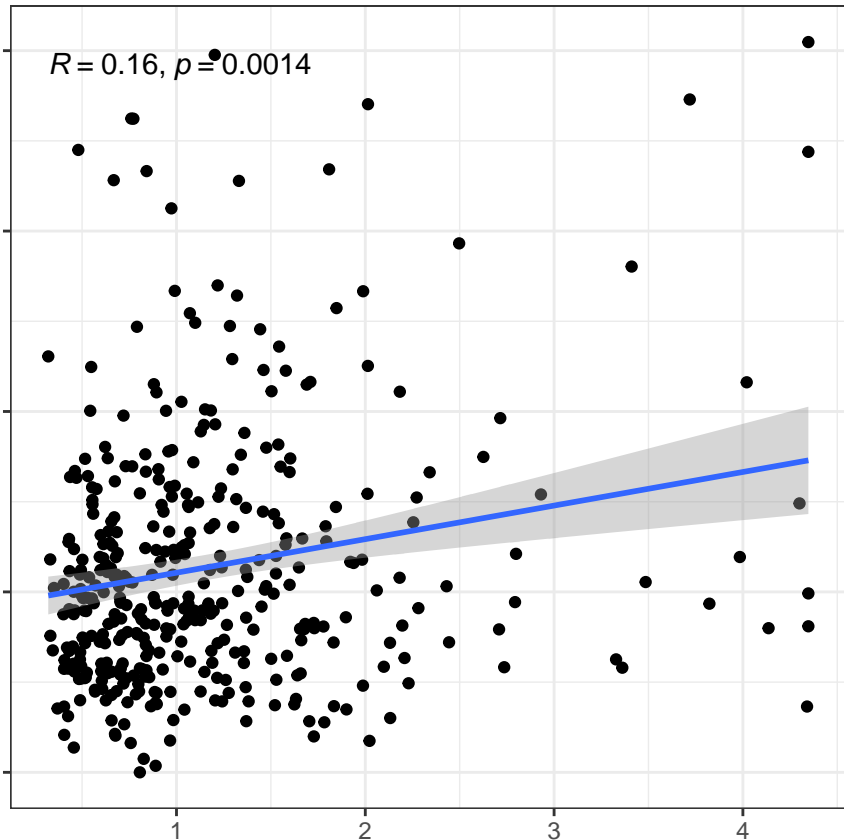

Supplement: Supplementary file 6 [file DataSheet_6.zip › cor.Macrophage M1_QUANTISEQ.pdf]

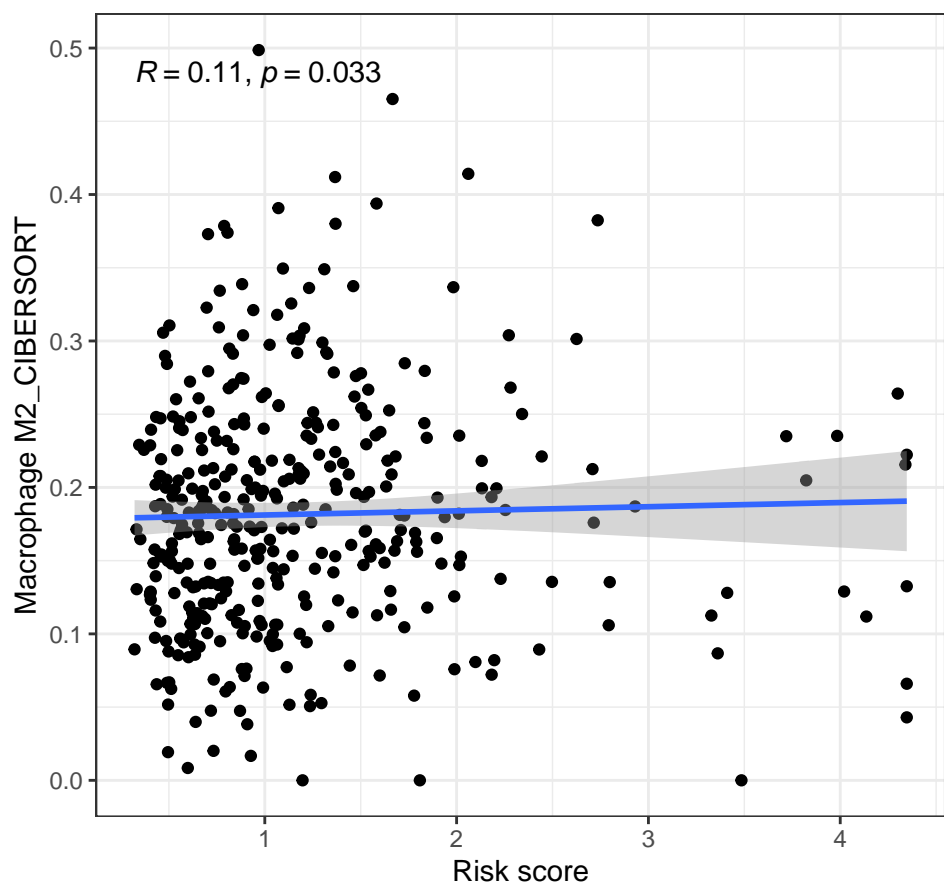

Supplement: Supplementary file 6 [file DataSheet_6.zip › cor.Macrophage M2_CIBERSORT.pdf]

Macrophage M2\_CIBERSORT-ABS

$R = -0.14, p = 0.0075$

0.0

0.1

0.2

0.3

0.4

0.5

Risk score

1

2

3

4

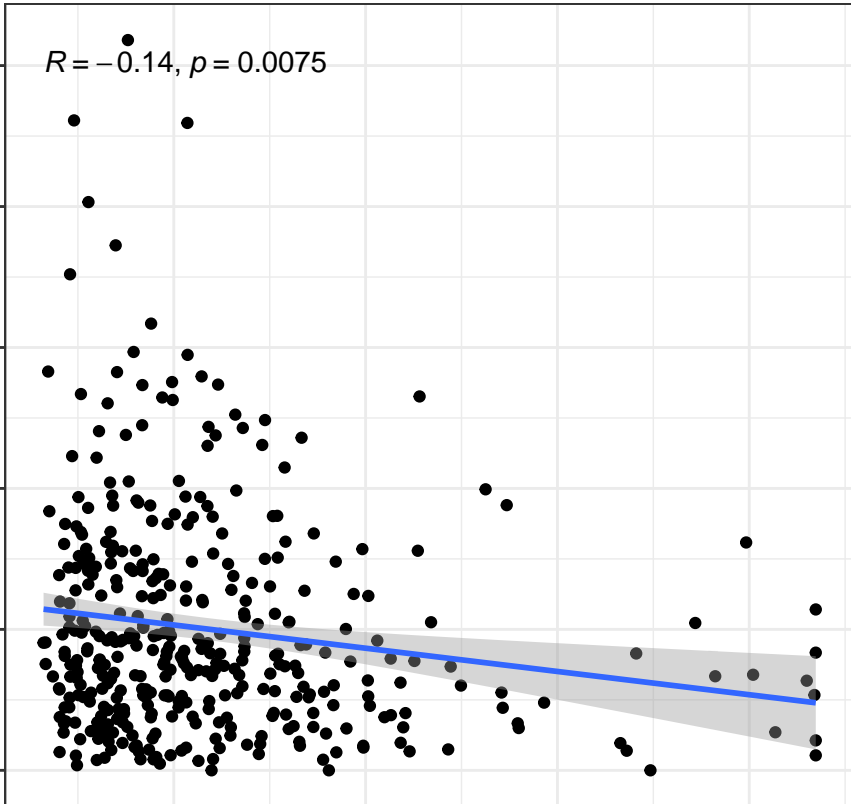

Supplement: Supplementary file 6 [file DataSheet_6.zip › cor.Macrophage M2_CIBERSORT-ABS.pdf]

Macrophage M2\_QUANTISEQ

0.100  
0.075  
0.050  
0.025  
0.000

$R = -0.17, p = 0.00078$

Risk score

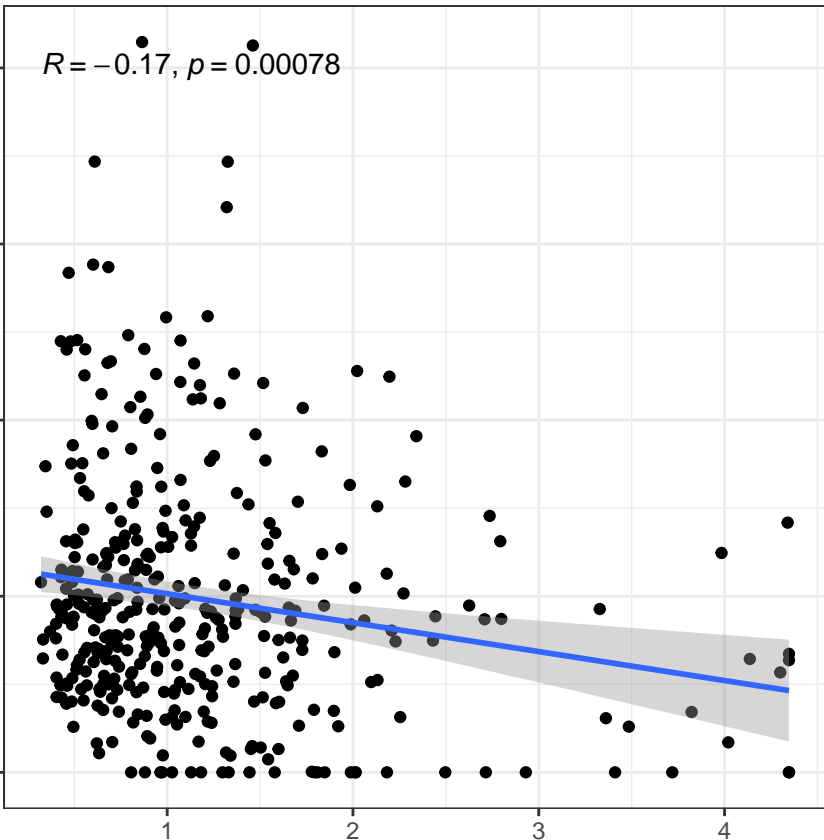

Supplement: Supplementary file 6 [file DataSheet_6.zip › cor.Macrophage M2_QUANTISEQ.pdf]

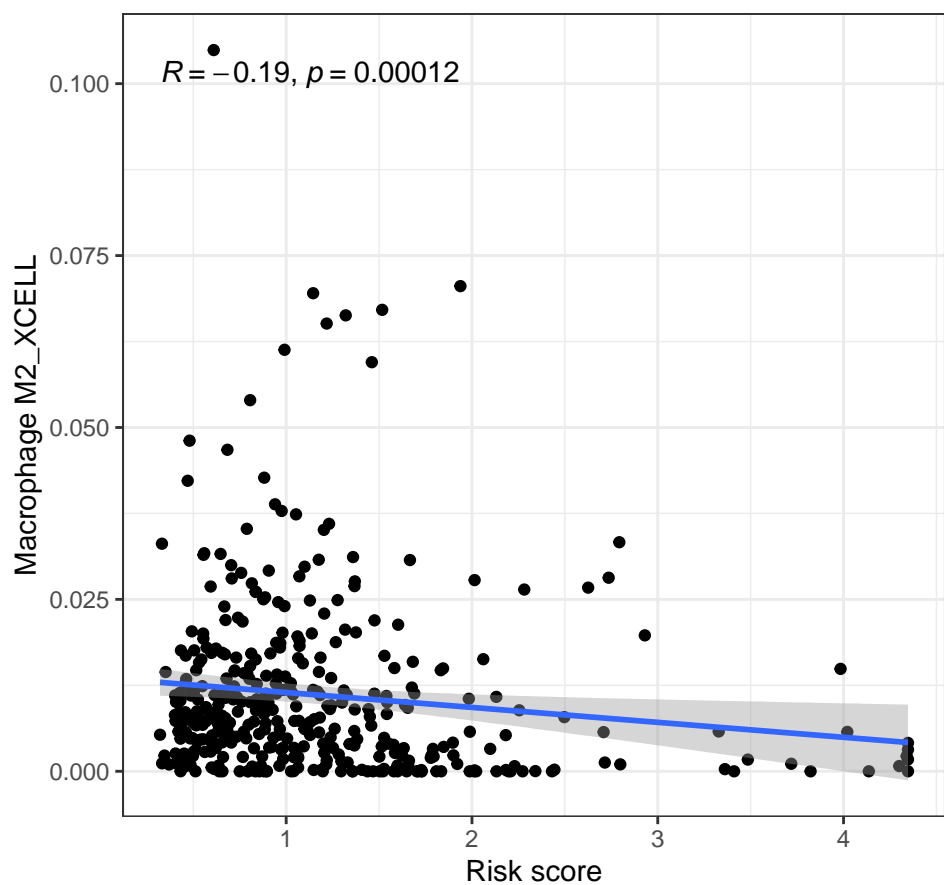

Supplement: Supplementary file 6 [file DataSheet_6.zip › cor.Macrophage M2_XCELL.pdf]

Macrophage/Monocyte\_MCPCOUNTER

$R = -0.15, p = 0.0042$

Risk score

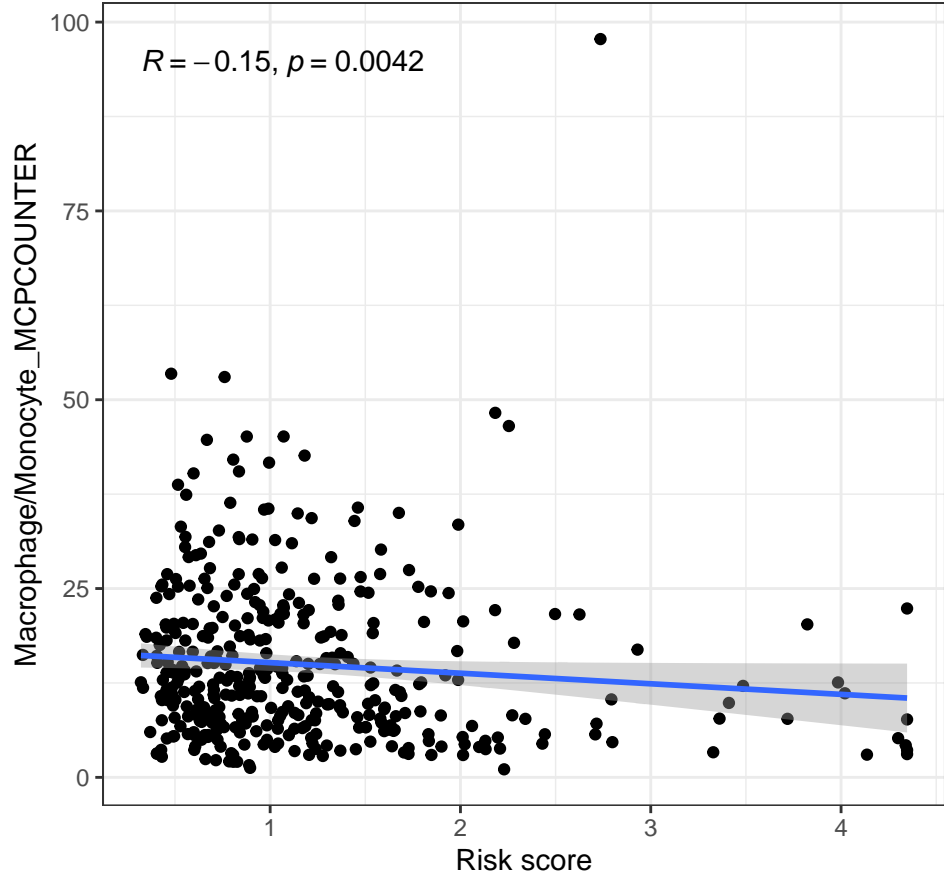

Supplement: Supplementary file 6 [file DataSheet_6.zip › cor.Macrophage_Monocyte_MCPCOUNTER.pdf]

Mast cell activated\_CIBERSORT

$R = -0.15$ ,  $p = 0.0038$

0.10

0.05

0.00

1

Risk score

3

4

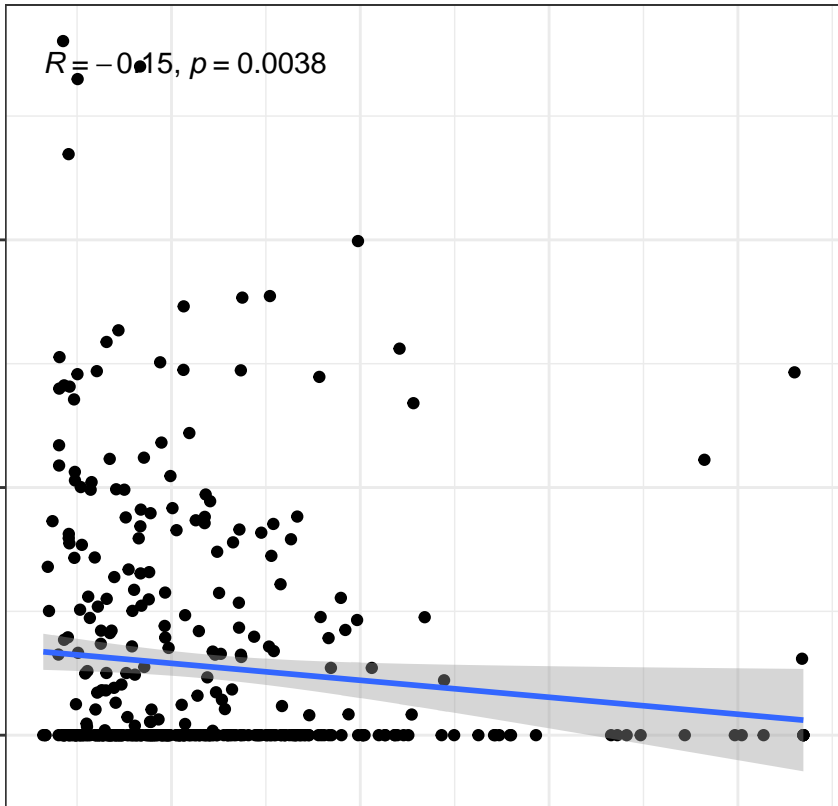

Supplement: Supplementary file 6 [file DataSheet_6.zip › cor.Mast cell activated_CIBERSORT.pdf]

Mast cell activated\_CIBERSORT-ABS

0.08

0.06

0.04

0.02

0.00

$R = -0.16, p = 0.0017$

1

Risk score

3

4

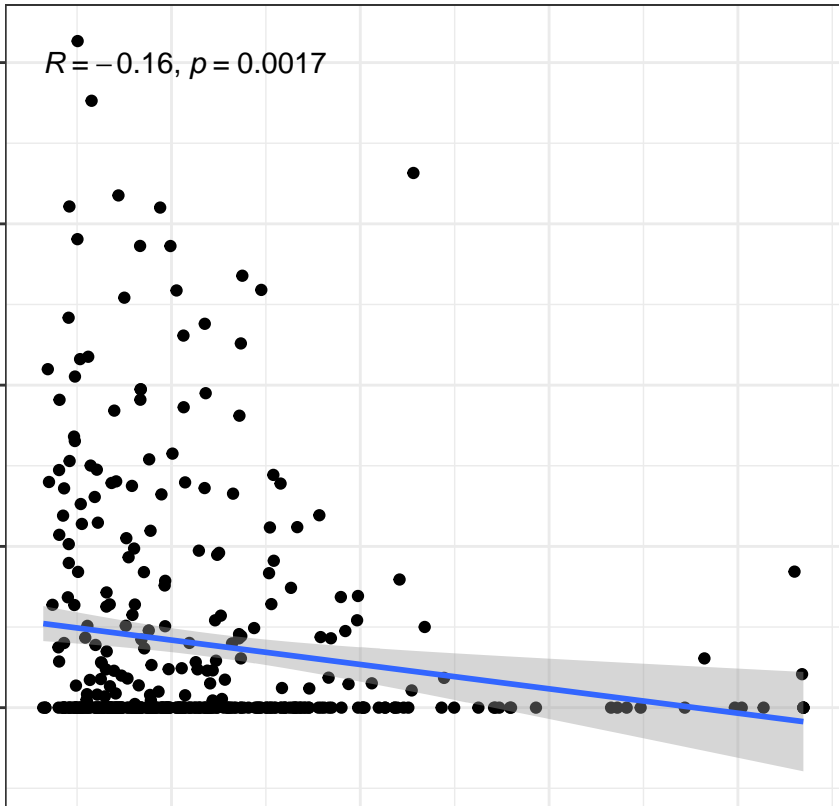

Supplement: Supplementary file 6 [file DataSheet_6.zip › cor.Mast cell activated_CIBERSORT-ABS.pdf]

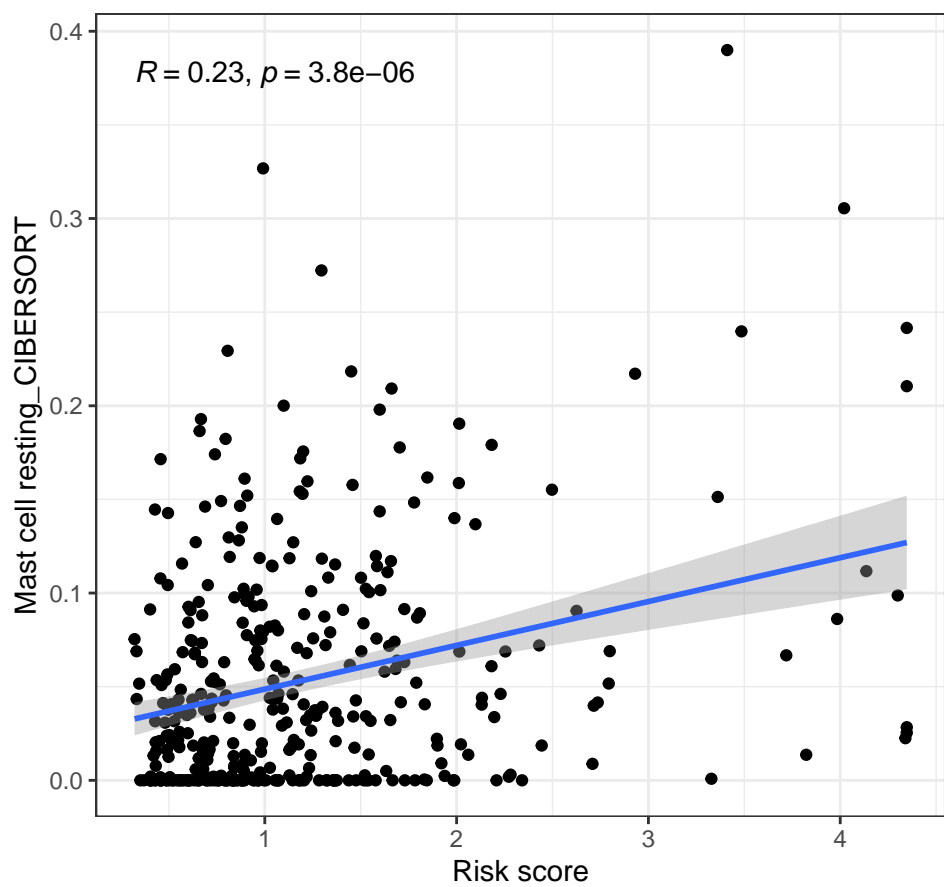

Supplement: Supplementary file 6 [file DataSheet_6.zip › cor.Mast cell resting_CIBERSORT.pdf]

Mast cell resting\_CIBERSORT-ABS

$R = 0.11, p = 0.023$

0.25  
0.20  
0.15  
0.10  
0.05  
0.00

Risk score

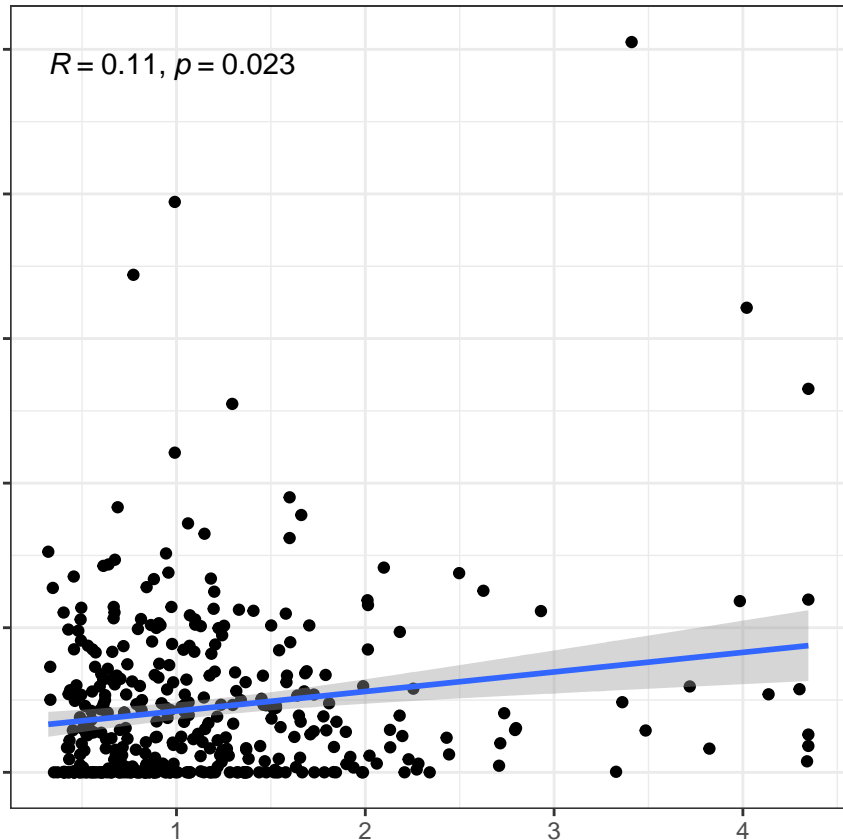

Supplement: Supplementary file 6 [file DataSheet_6.zip › cor.Mast cell resting_CIBERSORT-ABS.pdf]

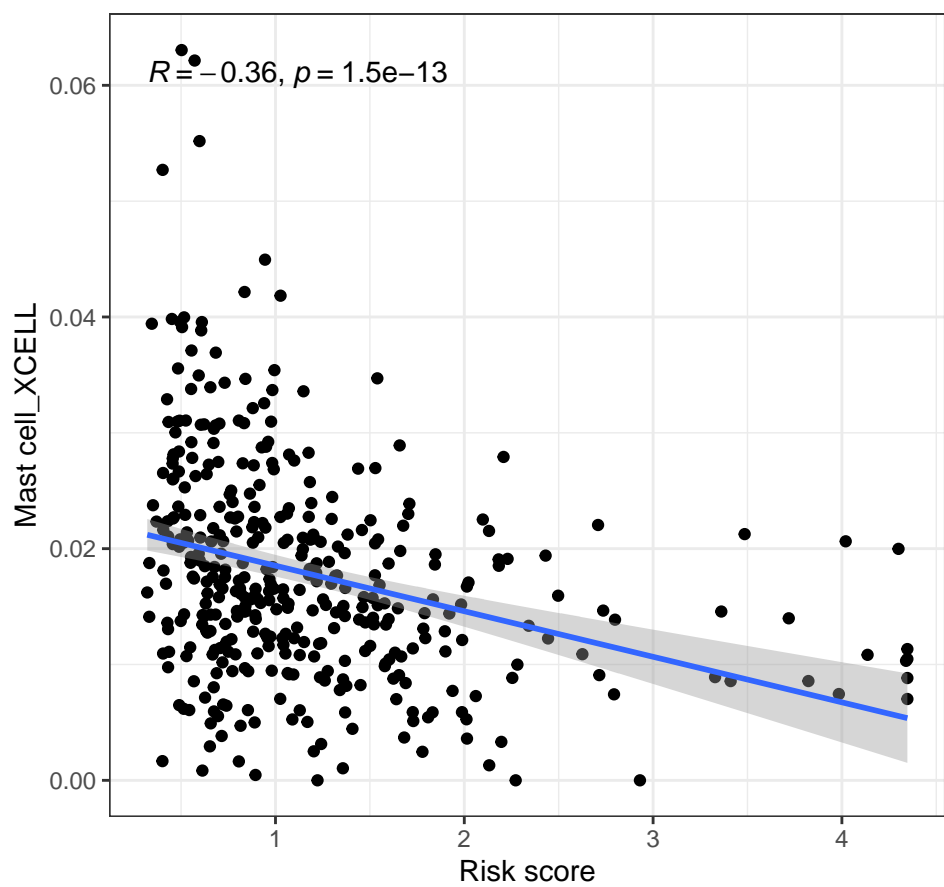

Supplement: Supplementary file 6 [file DataSheet_6.zip › cor.Mast cell_XCELL.pdf]

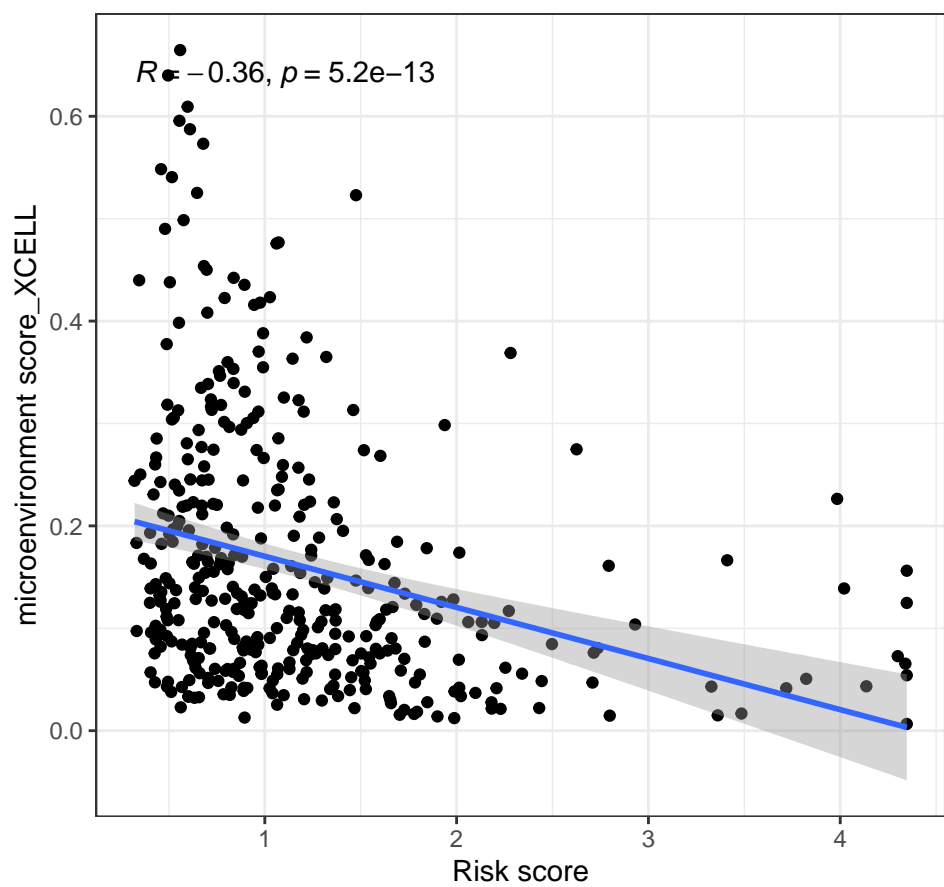

Supplement: Supplementary file 6 [file DataSheet_6.zip › cor.microenvironment score_XCELL.pdf]

Monocyte\_MCPOUNTER

$R = -0.15, p = 0.0042$

Risk score

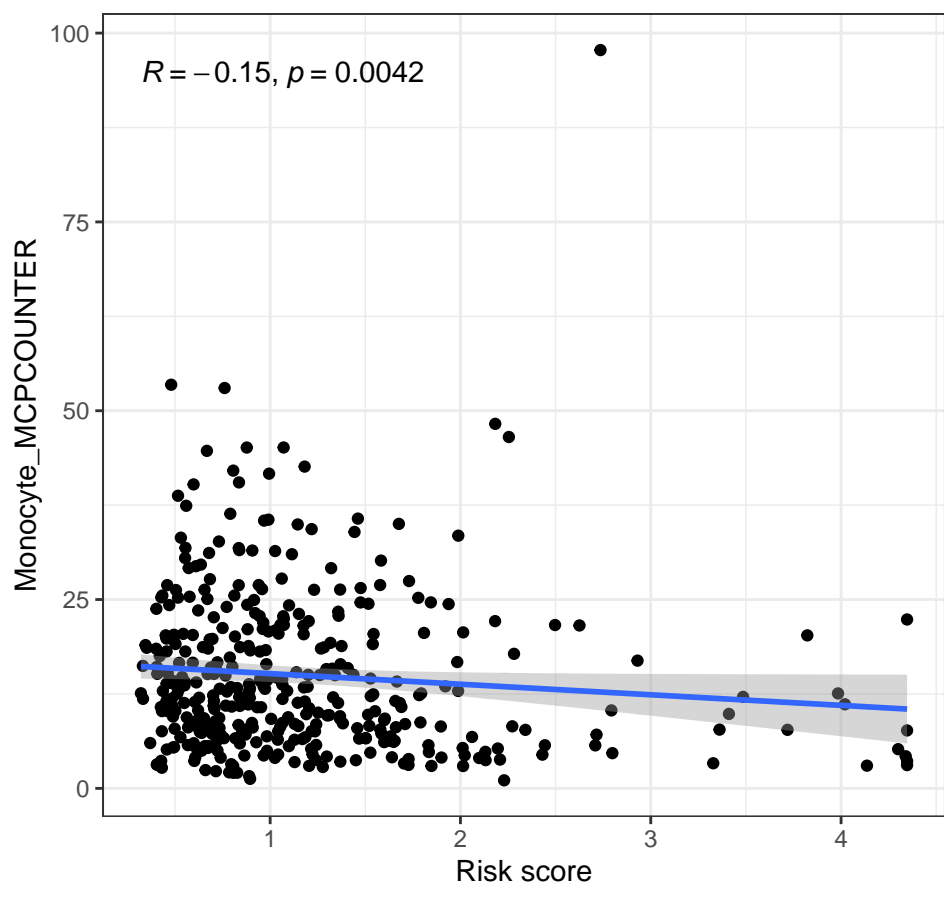

Supplement: Supplementary file 6 [file DataSheet_6.zip › cor.Monocyte_MCPCOUNTER.pdf]

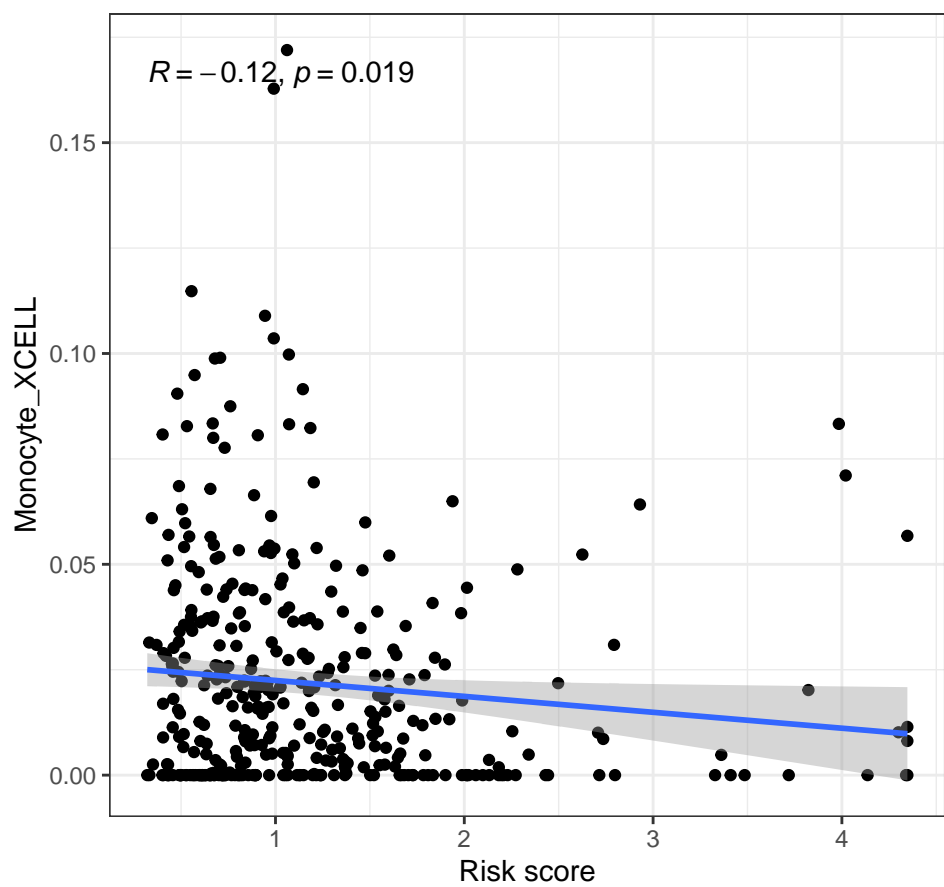

Supplement: Supplementary file 6 [file DataSheet_6.zip › cor.Monocyte_XCELL.pdf]

Myeloid dendritic cell activated\_XCELL

$R = -0.29$ ,  $p = 5.2e-09$

0.6  
0.4  
0.2  
0.0

Risk score

1

2

3

4

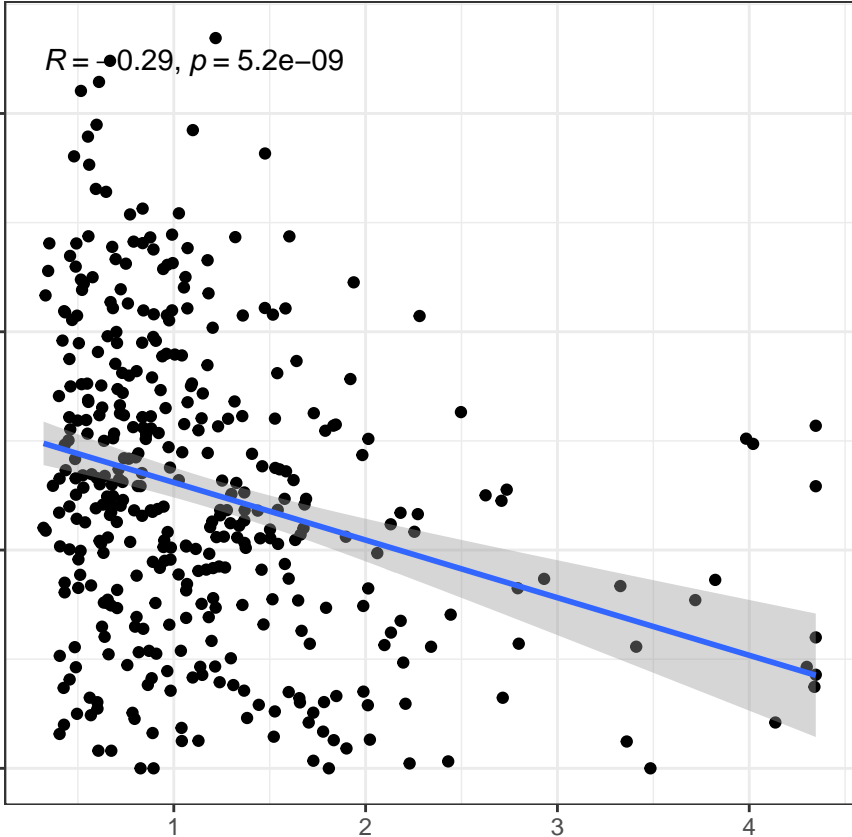

Supplement: Supplementary file 6 [file DataSheet_6.zip › cor.Myeloid dendritic cell activated_XCELL.pdf]

Myeloid dendritic cell resting\_CIBERSORT-ABS

$R = -0.15, p = 0.0034$

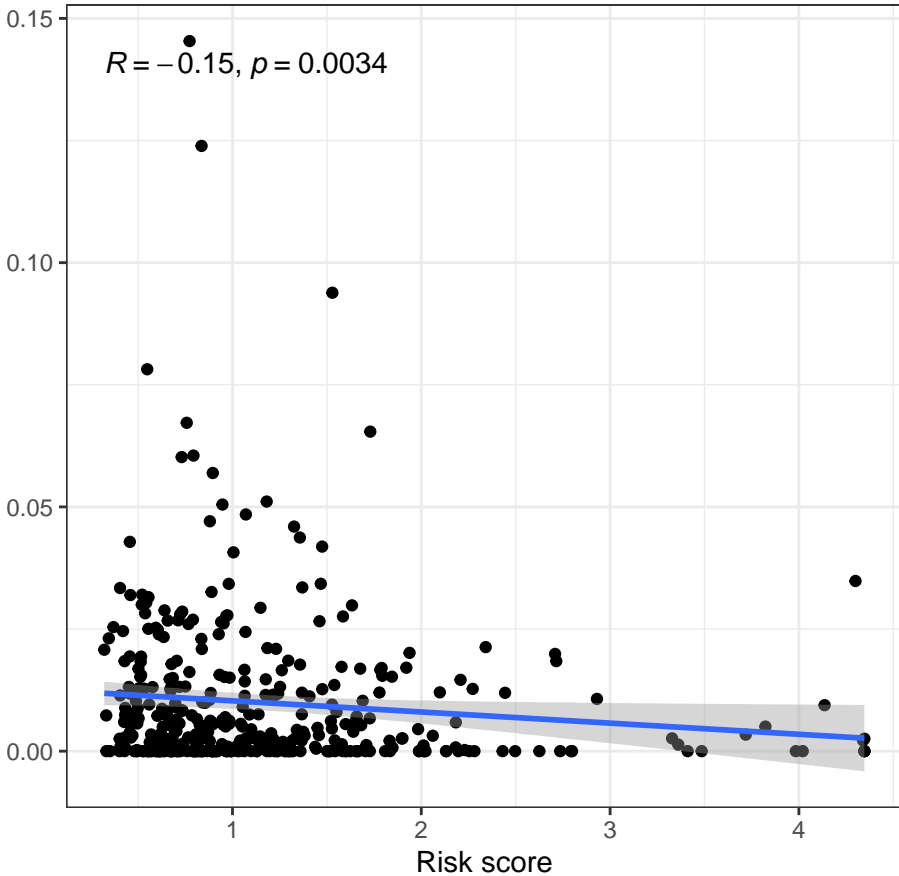

Supplement: Supplementary file 6 [file DataSheet_6.zip › cor.Myeloid dendritic cell resting_CIBERSORT-ABS.pdf]

Myeloid dendritic cell\_MCPOUNTER

$R = -0.26$ ,  $p = 3.3\text{e-}07$

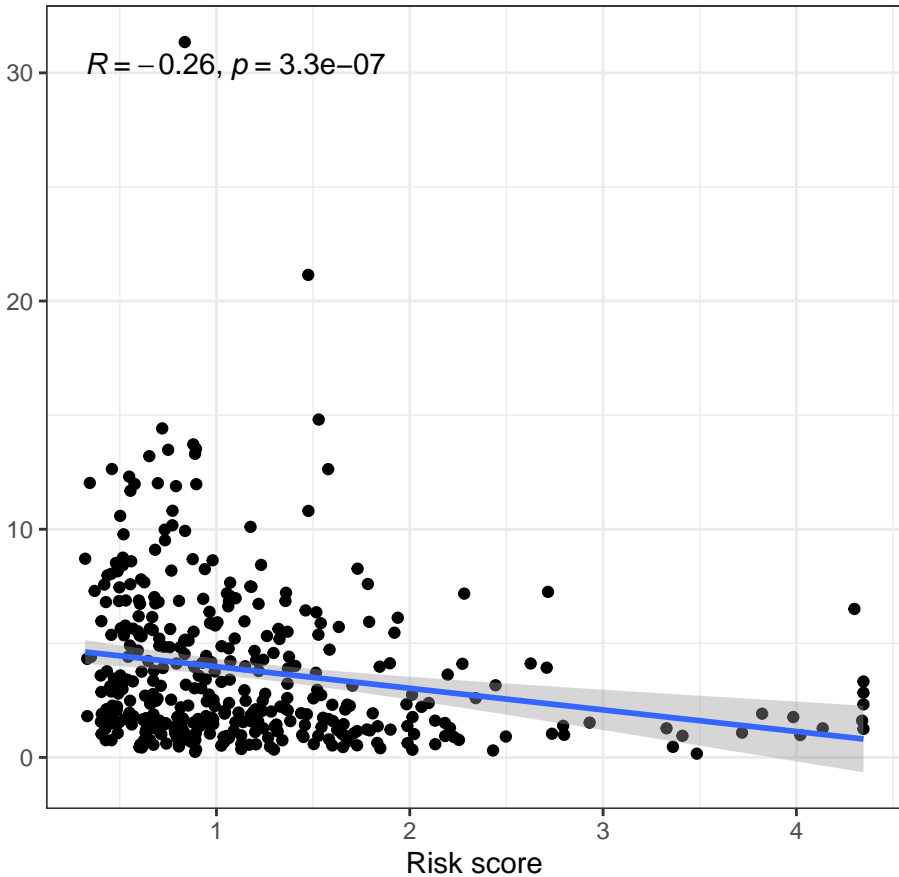

Supplement: Supplementary file 6 [file DataSheet_6.zip › cor.Myeloid dendritic cell_MCPCOUNTER.pdf]

Myeloid dendritic cell\_QUANTISEQ

$R = -0.11, p = 0.033$

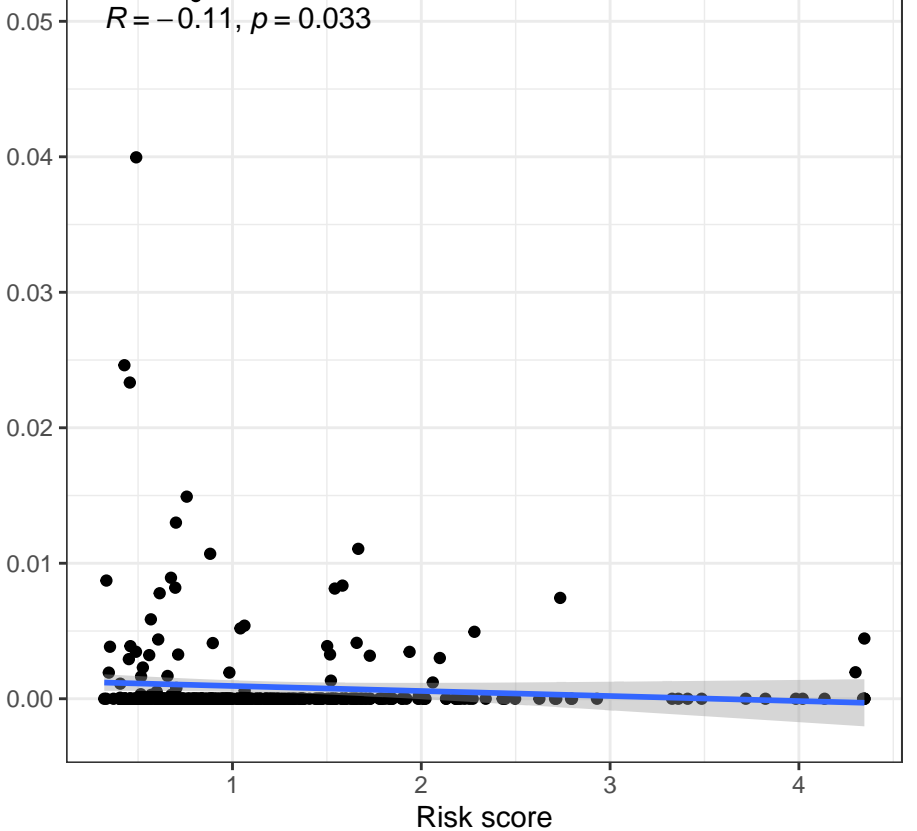

Supplement: Supplementary file 6 [file DataSheet_6.zip › cor.Myeloid dendritic cell_QUANTISEQ.pdf]

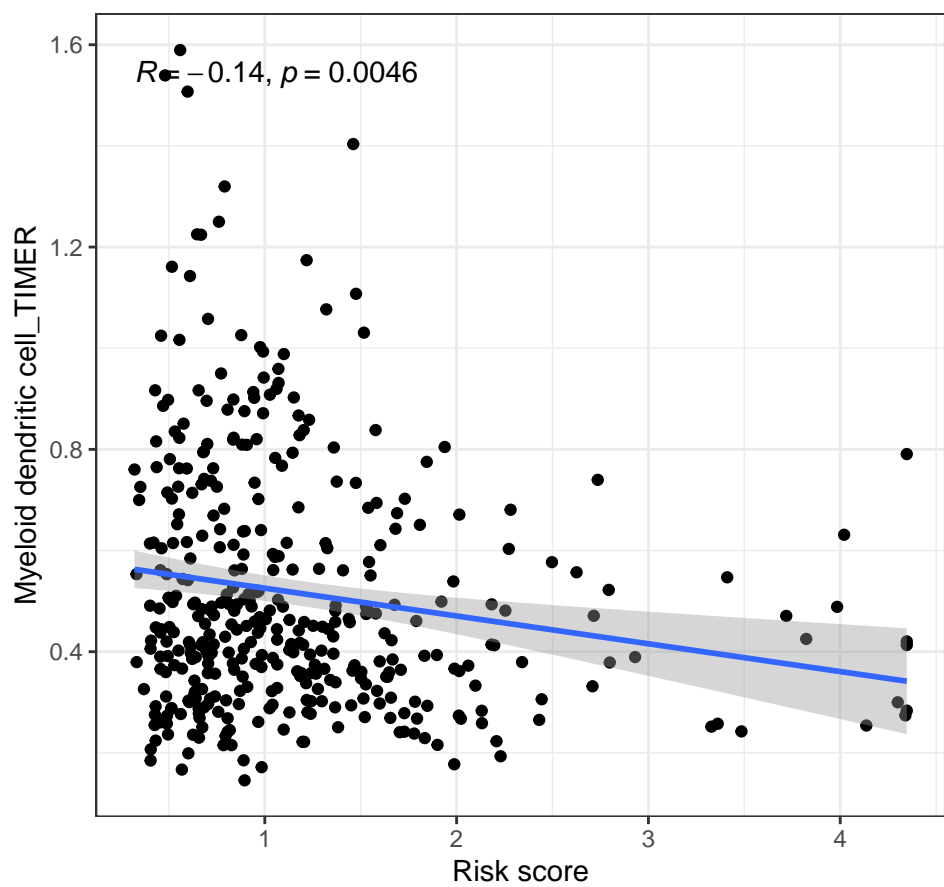

Supplement: Supplementary file 6 [file DataSheet_6.zip › cor.Myeloid dendritic cell_TIMER.pdf]

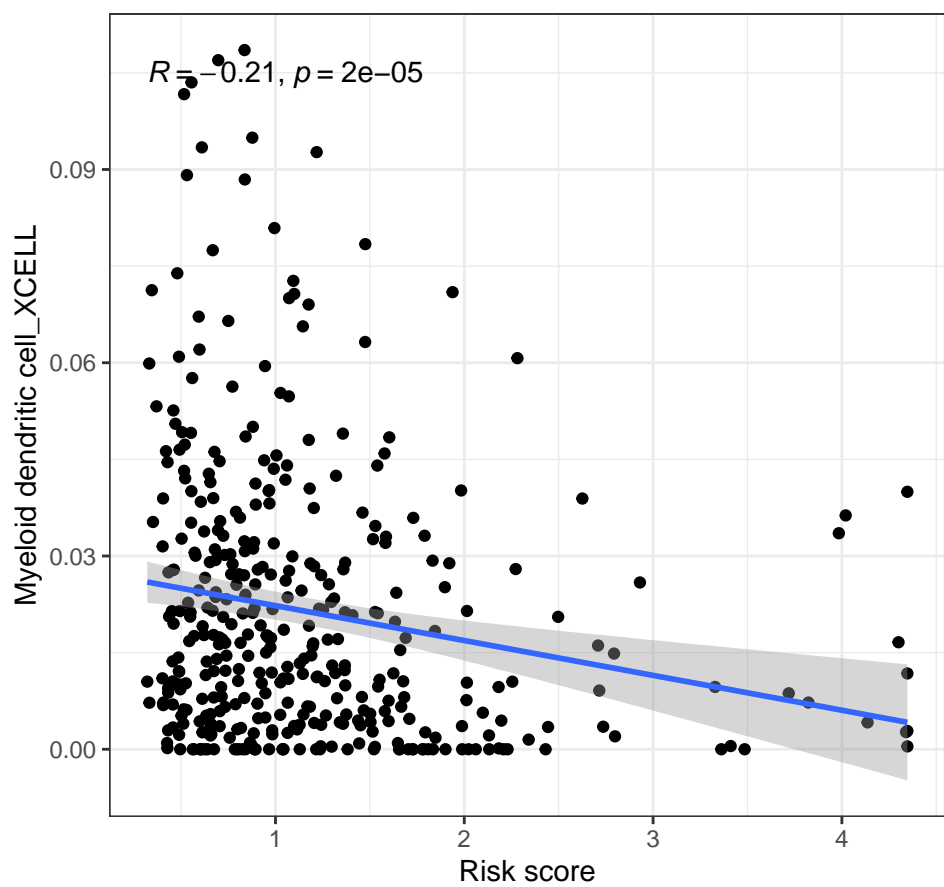

Supplement: Supplementary file 6 [file DataSheet_6.zip › cor.Myeloid dendritic cell_XCELL.pdf]

Neutrophil\_CIBERSORT

$R = -0.11, p = 0.03$

0.15

0.10

0.05

0.00

1

Risk score

3

4

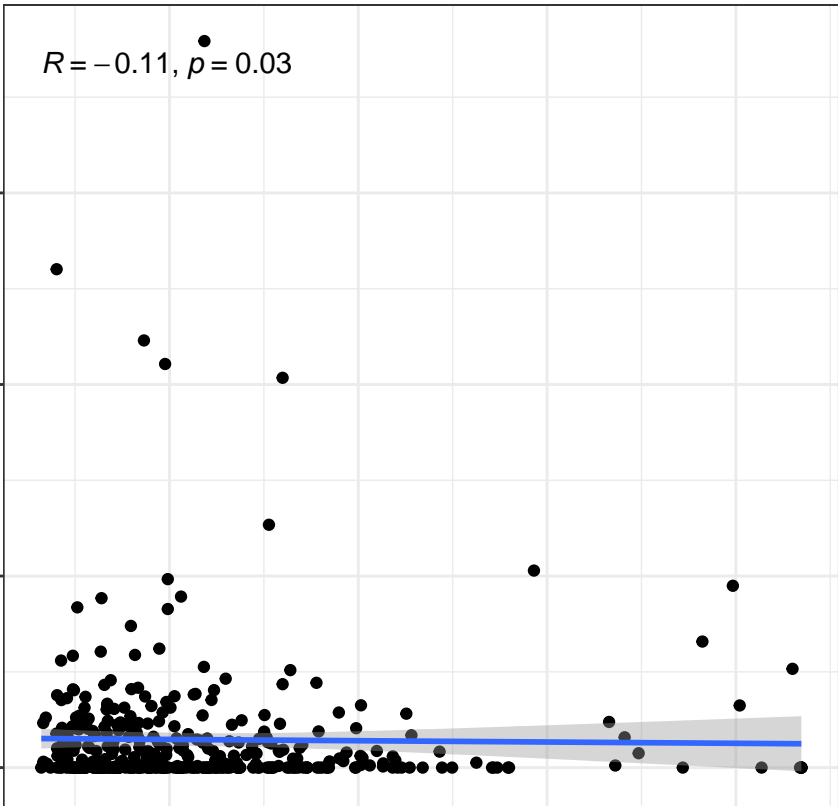

Supplement: Supplementary file 6 [file DataSheet_6.zip › cor.Neutrophil_CIBERSORT.pdf]

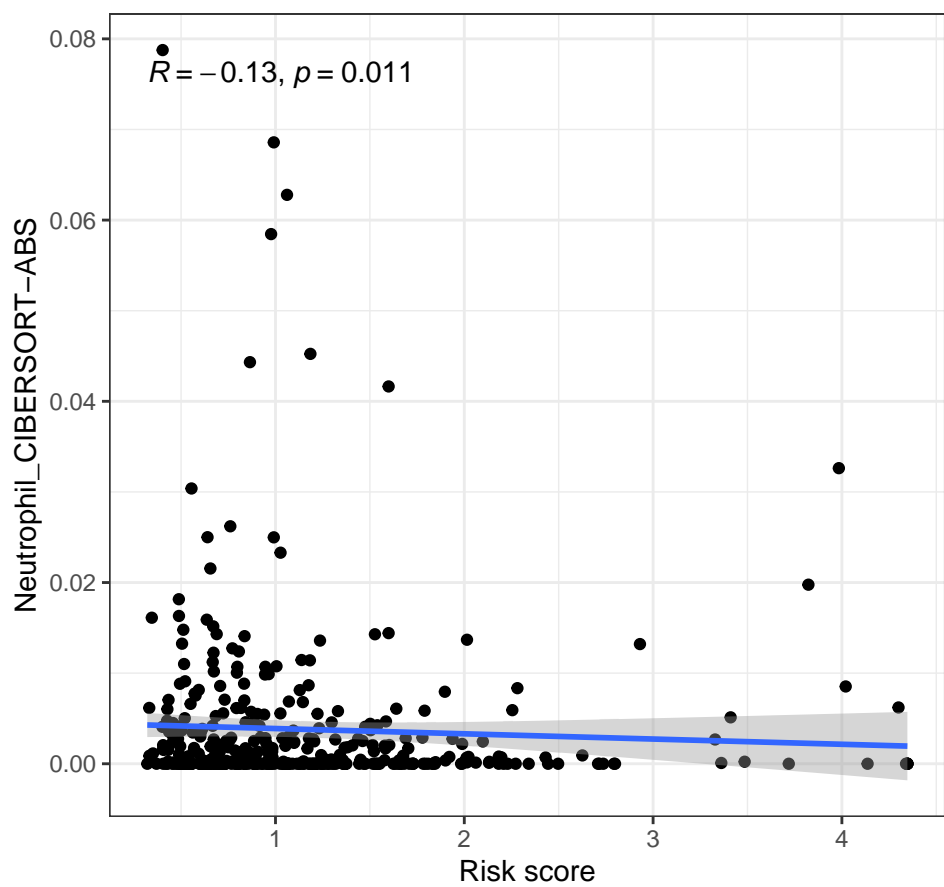

Supplement: Supplementary file 6 [file DataSheet_6.zip › cor.Neutrophil_CIBERSORT-ABS.pdf]

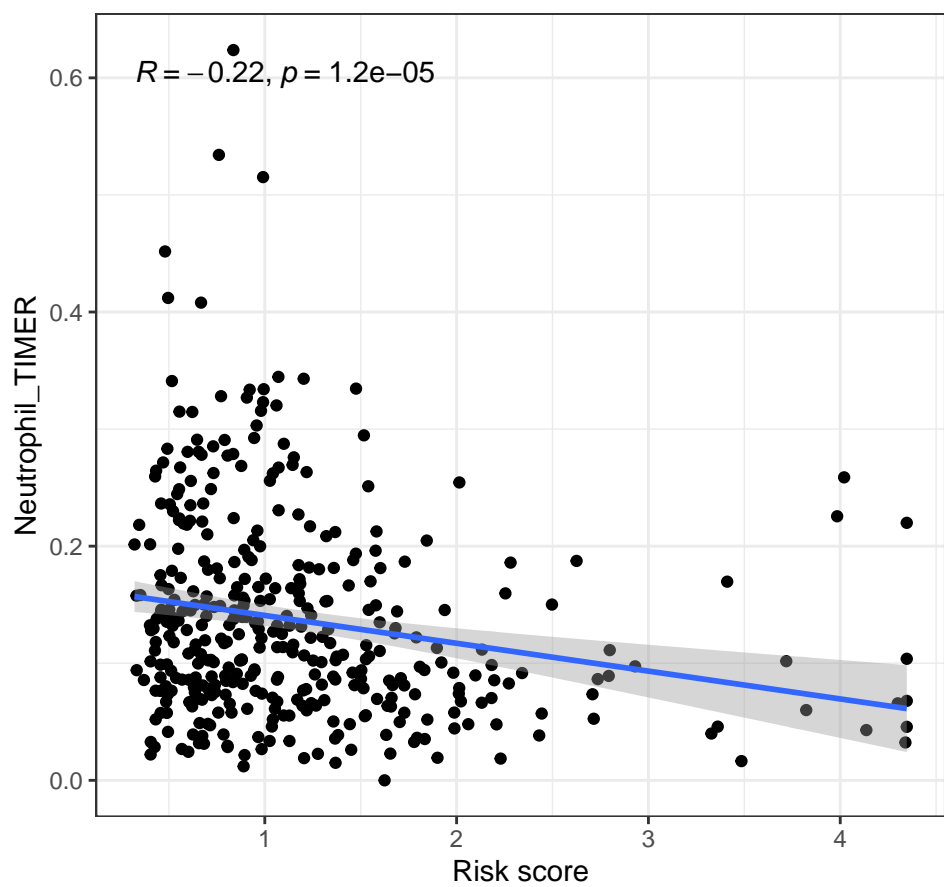

Supplement: Supplementary file 6 [file DataSheet_6.zip › cor.Neutrophil_TIMER.pdf]

NK cell activated\_CIBERSORT-ABS

$R = -0.16, p = 0.0014$

0.15

0.10

0.05

0.00

1

Risk score

3

4

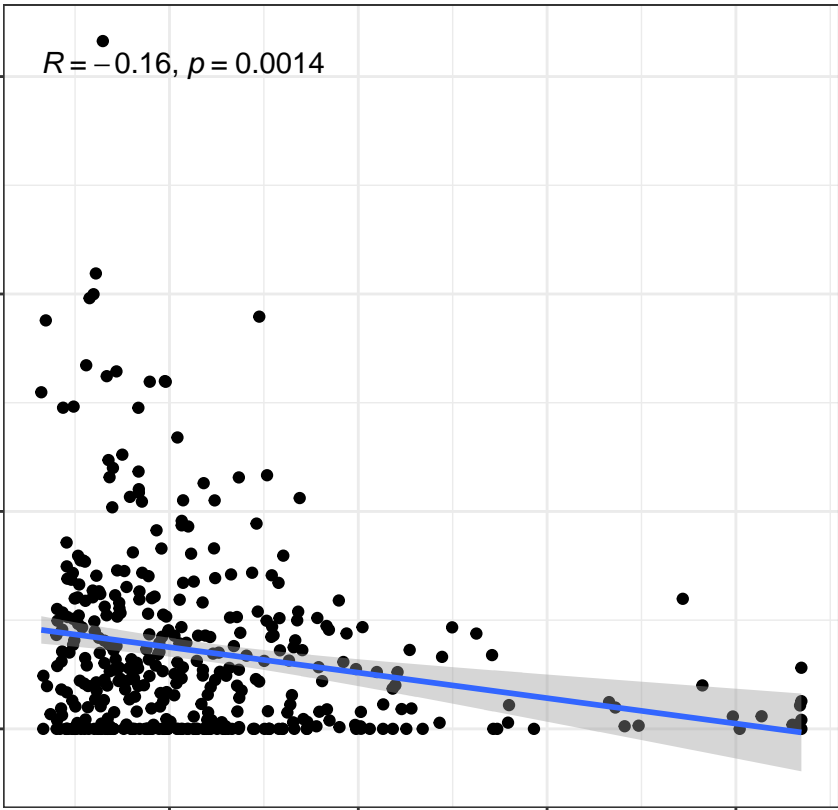

Supplement: Supplementary file 6 [file DataSheet_6.zip › cor.NK cell activated_CIBERSORT-ABS.pdf]

NK cell resting\_CIBERSORT

$R = 0.14, p = 0.0076$

0.09

0.06

0.03

0.00

1

Risk score

3

4

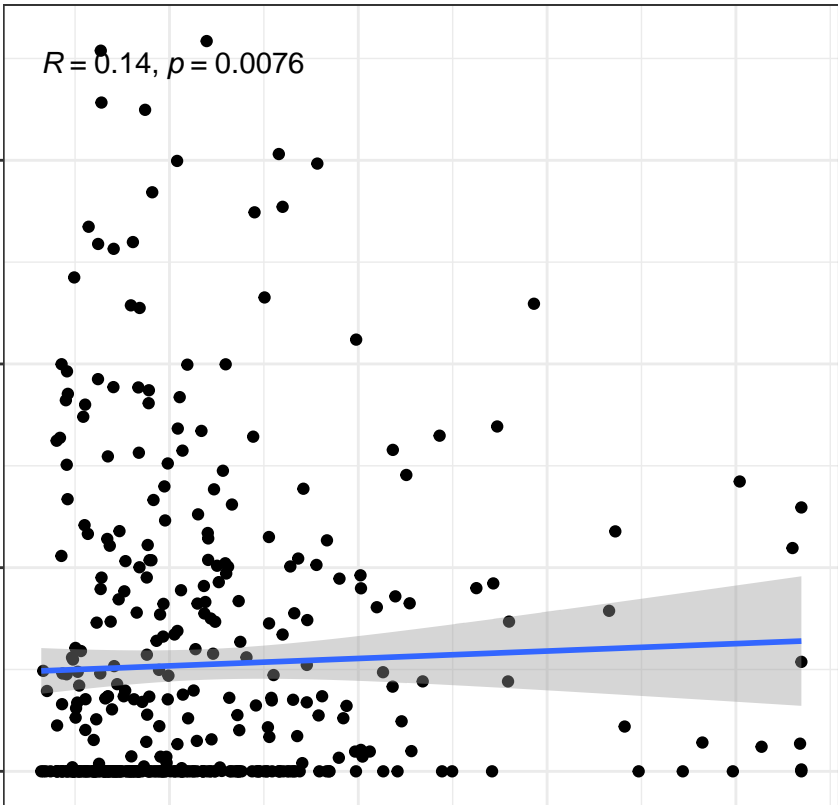

Supplement: Supplementary file 6 [file DataSheet_6.zip › cor.NK cell resting_CIBERSORT.pdf]

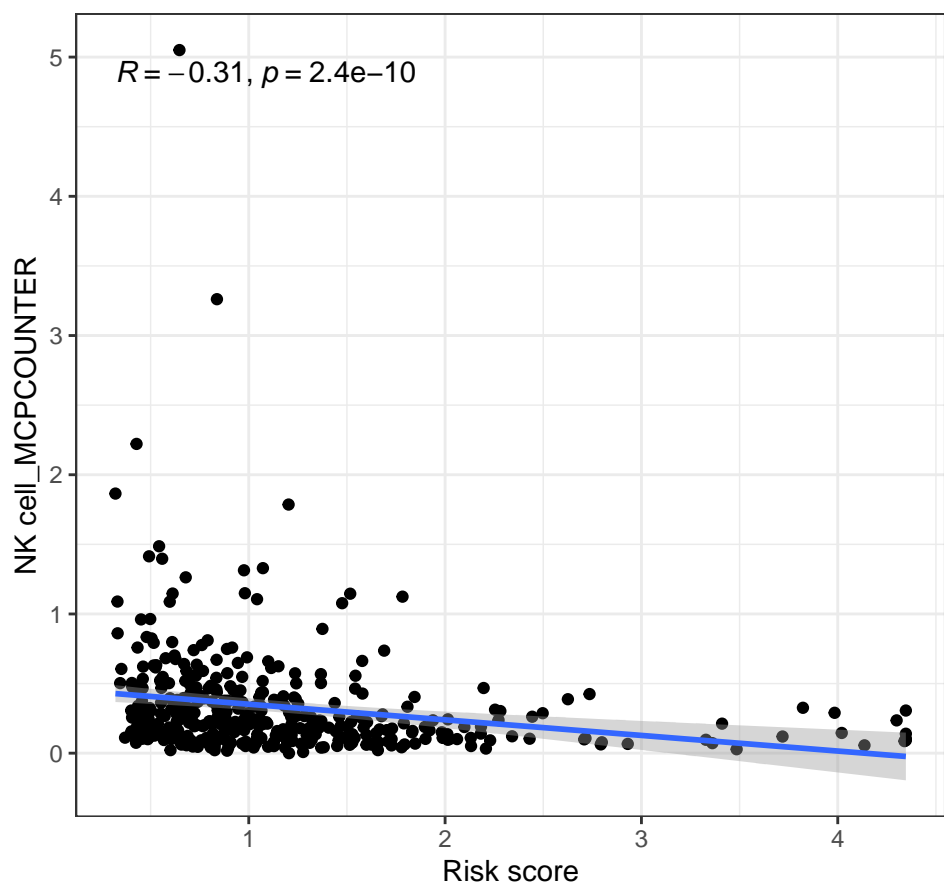

Supplement: Supplementary file 6 [file DataSheet_6.zip › cor.NK cell_MCPCOUNTER.pdf]

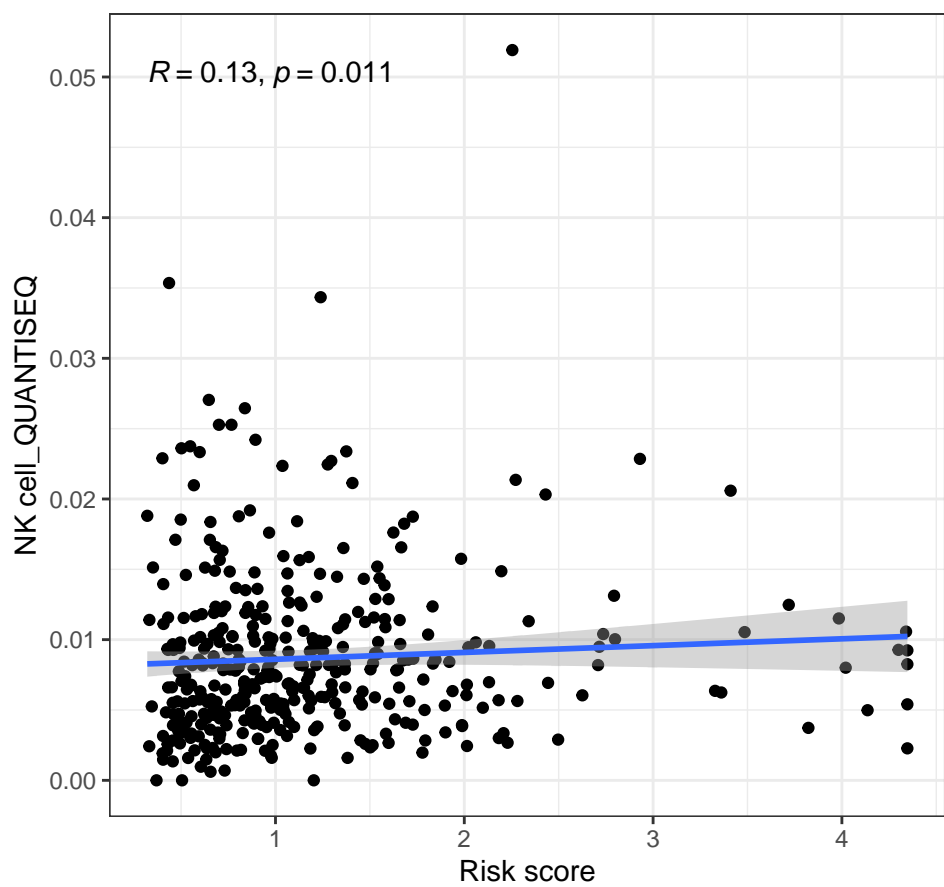

Supplement: Supplementary file 6 [file DataSheet_6.zip › cor.NK cell_QUANTISEQ.pdf]

Plasmacytoid dendritic cell\_XCELL

$R = -0.3, p = 2.9\text{e-}09$

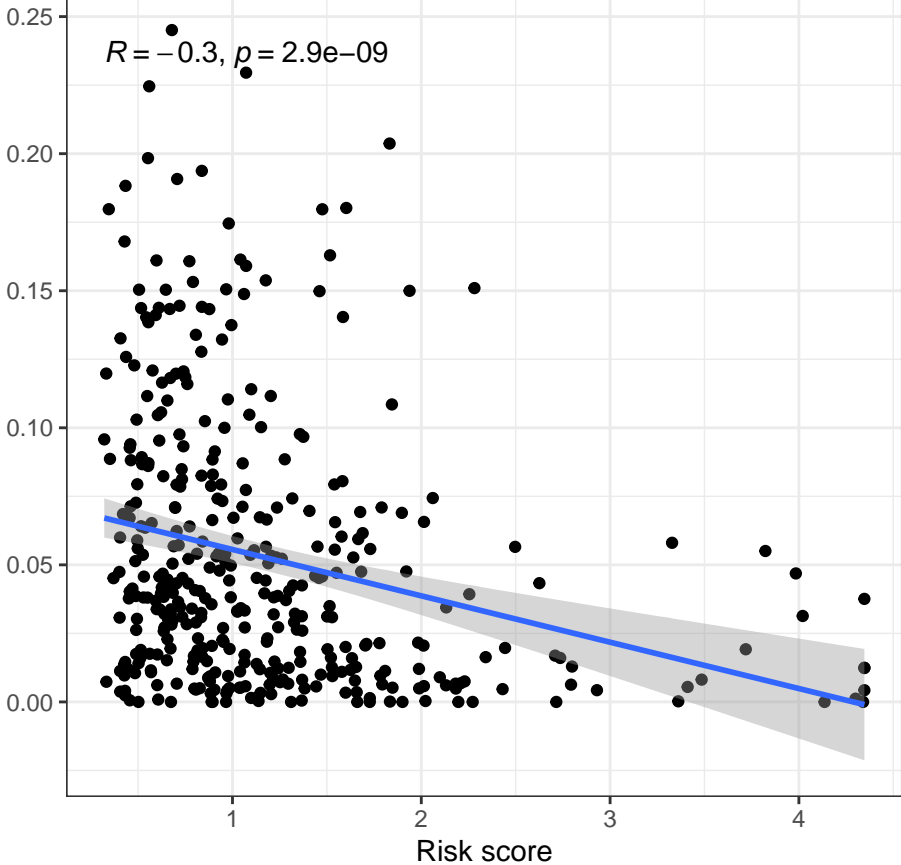

Supplement: Supplementary file 6 [file DataSheet_6.zip › cor.Plasmacytoid dendritic cell_XCELL.pdf]
