# Supplementary material for: Single-cell RNA sequencing and traditional RNA sequencing reveals the role of cancer-associated fibroblasts in oral squamous cell carcinoma cohort
Source: Front Oncol. 2023 May 10;13:1195520. doi: 10.3389/fonc.2023.1195520 (PMC10206127; doi:10.3389/fonc.2023.1195520)
Supplement: Supplementary file 6 [file DataSheet_6.zip › vioplot.pdf]

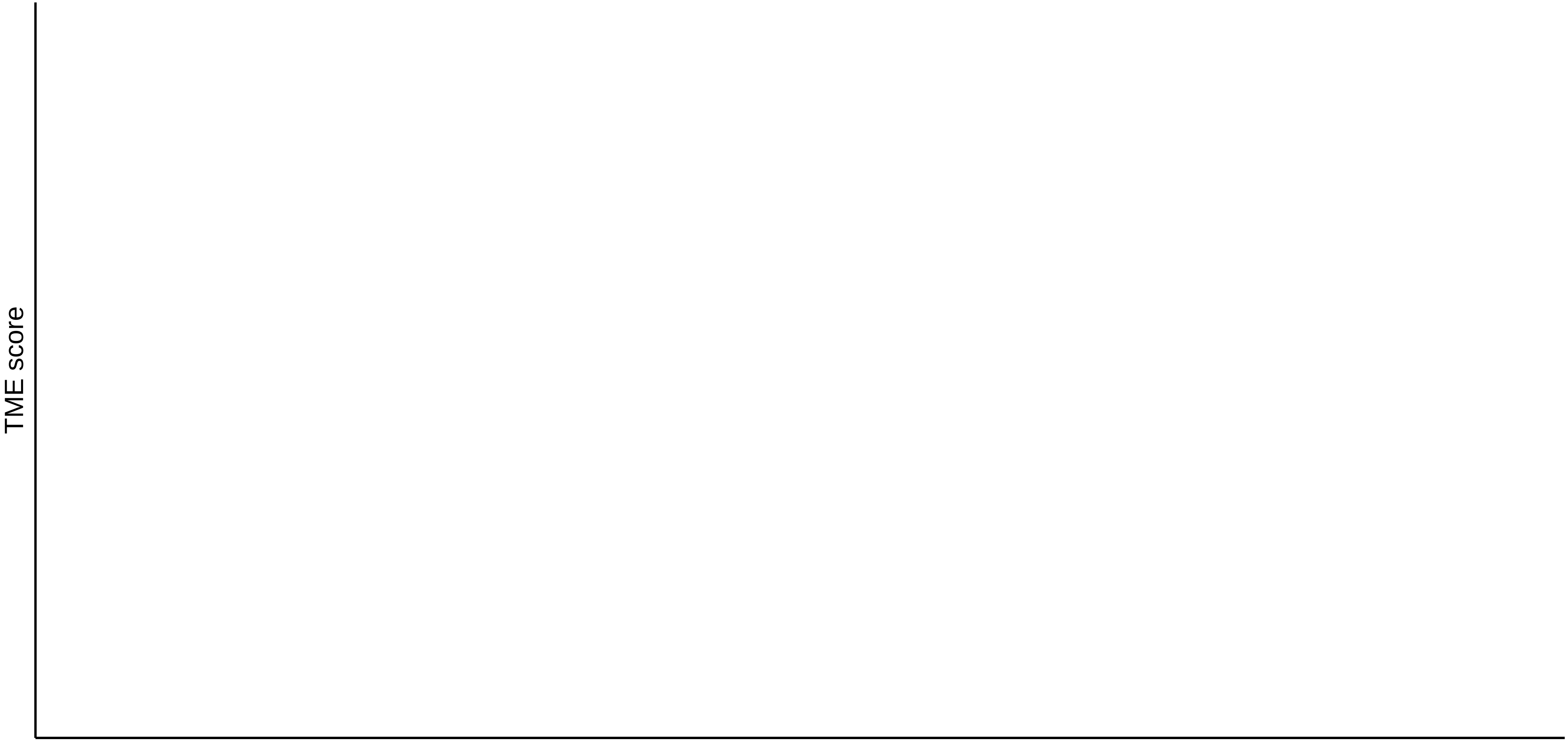
TME score
