# Supplementary figures and images for: Single-cell RNA sequencing and traditional RNA sequencing reveals the role of cancer-associated fibroblasts in oral squamous cell carcinoma cohort (part 2 of 2)
Source: Front Oncol. 2023 May 10;13:1195520. doi: 10.3389/fonc.2023.1195520 (PMC10206127; doi:10.3389/fonc.2023.1195520)

T cell CD4+ (non-regulatory)\_QUANTISEQ

$R = 0.17, p = 0.00092$

0.075  
0.050  
0.025  
0.000

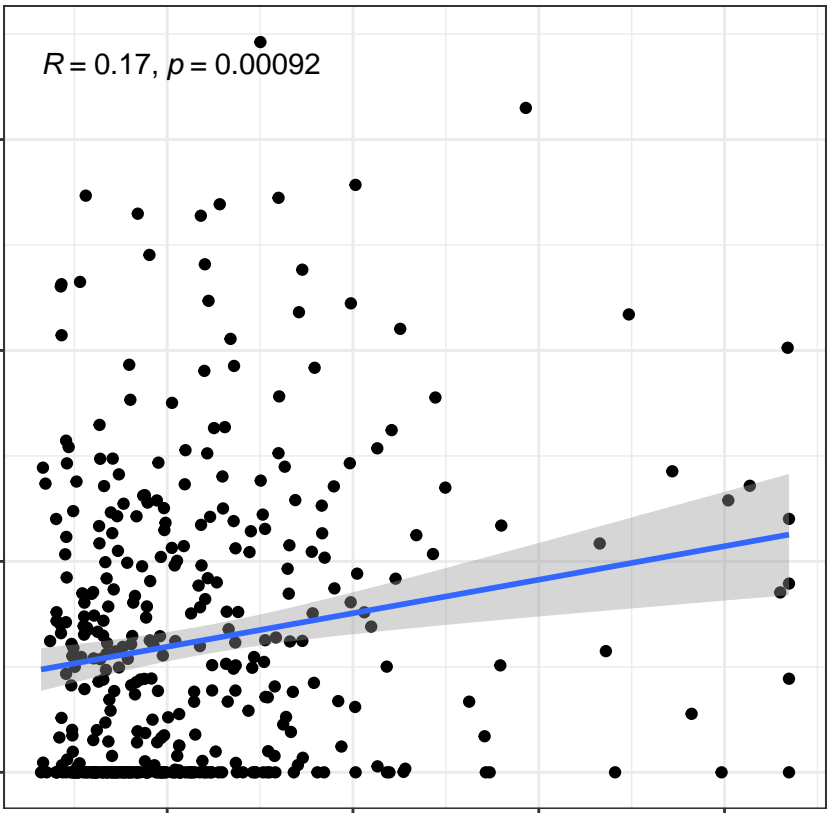

Risk score

Supplement: Supplementary file 6 [file DataSheet_6.zip › cor.T cell CD4+ (non-regulatory)_QUANTISEQ.pdf]

T cell CD4+ central memory\_XCELL

$R = -0.12, p = 0.021$

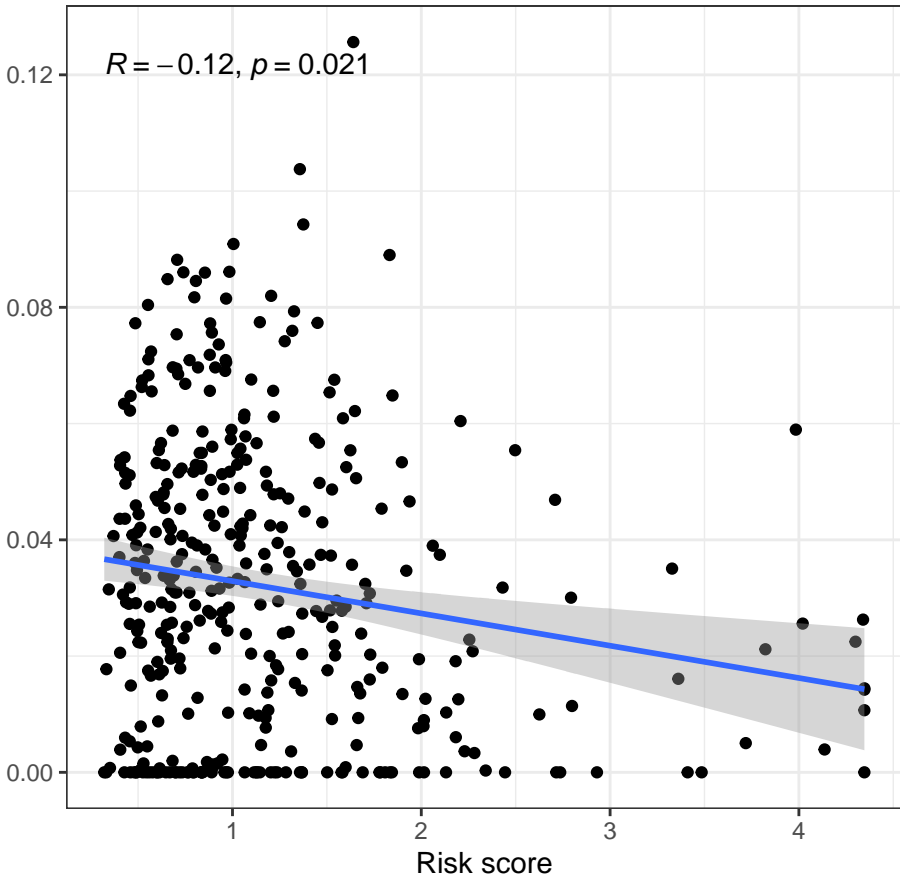

Supplement: Supplementary file 6 [file DataSheet_6.zip › cor.T cell CD4+ central memory_XCELL.pdf]

T cell CD4+ memory activated\_CIBERSORT

$R = -0.19, p = 0.00013$

0.20  
0.15  
0.10  
0.05  
0.00

Risk score

3

4

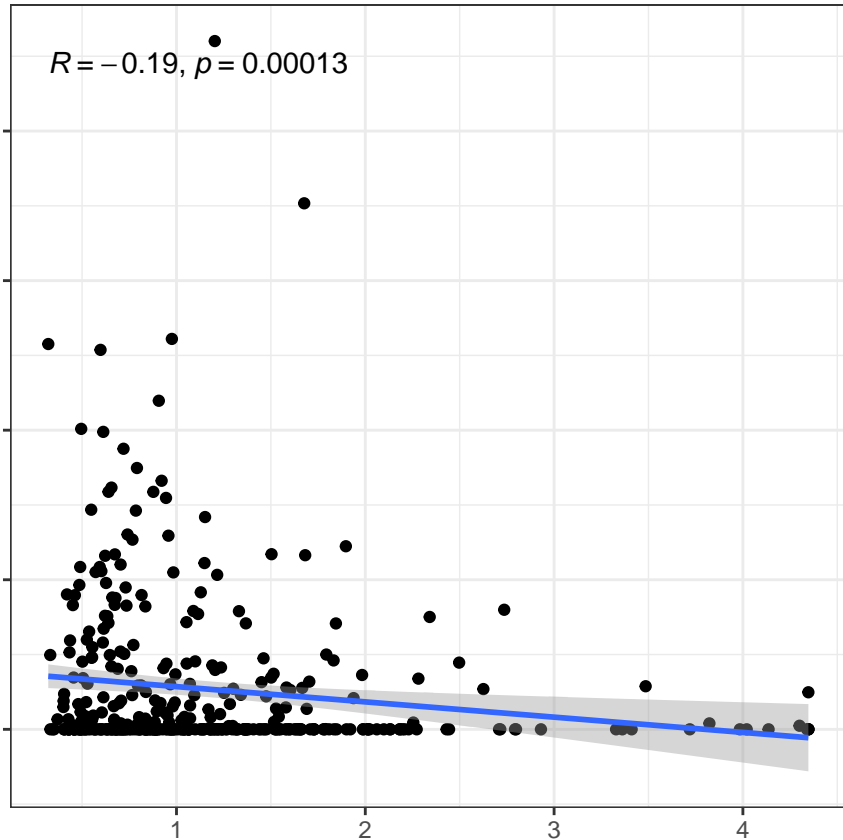

Supplement: Supplementary file 6 [file DataSheet_6.zip › cor.T cell CD4+ memory activated_CIBERSORT.pdf]

T cell CD4+ memory activated\_CIBERSORT-ABS

$R = -0.21$ ,  $p = 4.3e-05$

Risk score

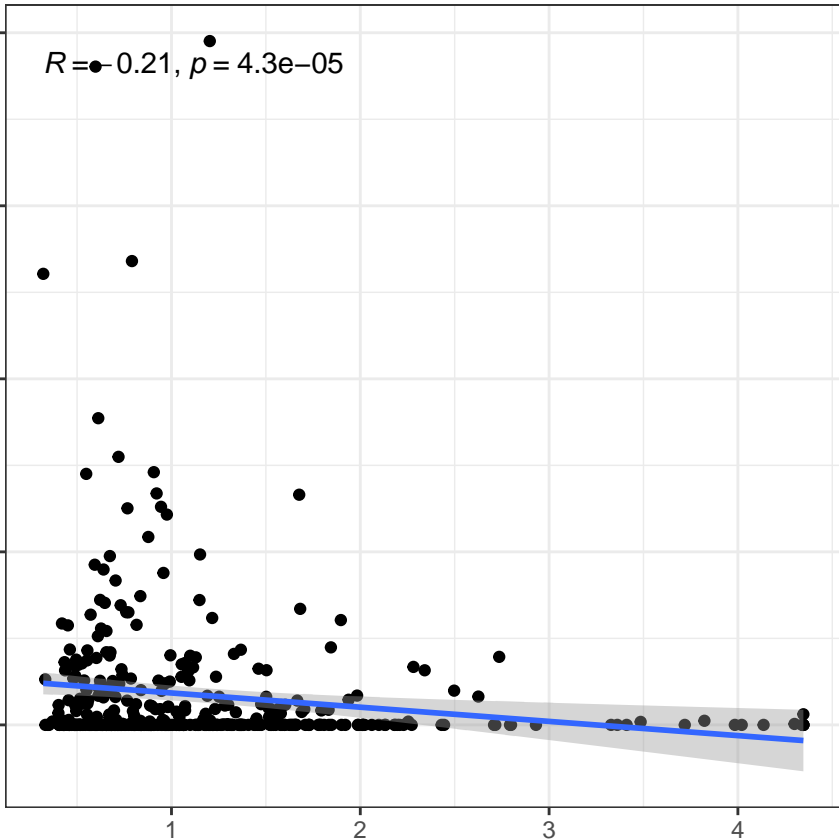

Supplement: Supplementary file 6 [file DataSheet_6.zip › cor.T cell CD4+ memory activated_CIBERSORT-ABS.pdf]

T cell CD4+ memory resting\_CIBERSORT\_ABS

$R = -0.13, p = 0.012$

0.3  
0.2  
0.1  
0.0

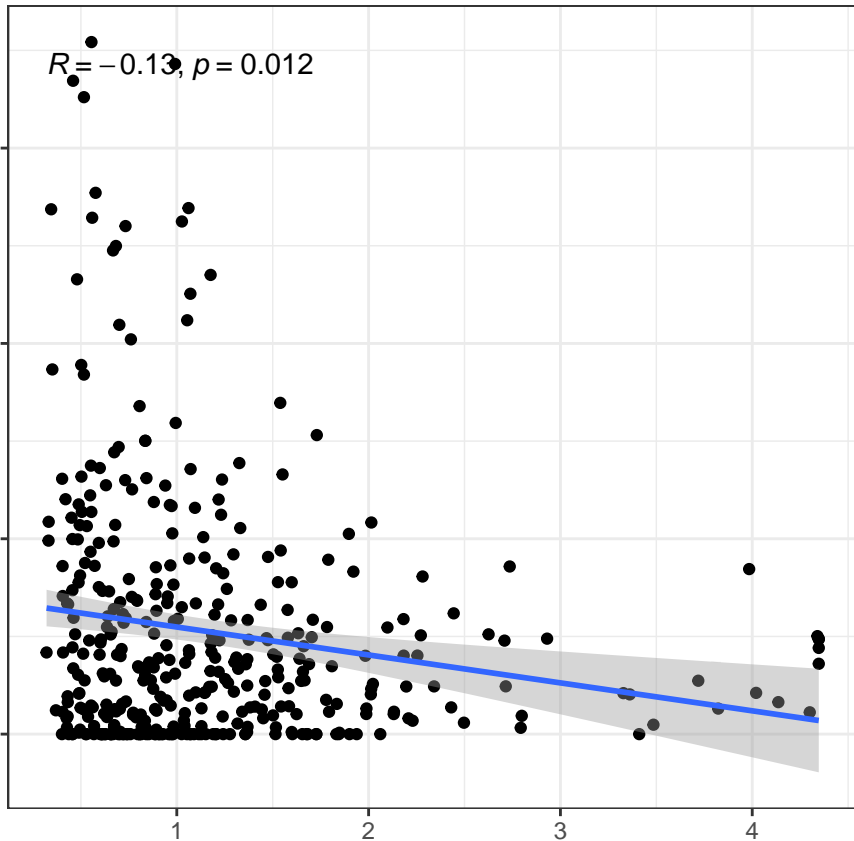

Risk score

Supplement: Supplementary file 6 [file DataSheet_6.zip › cor.T cell CD4+ memory resting_CIBERSORT-ABS.pdf]

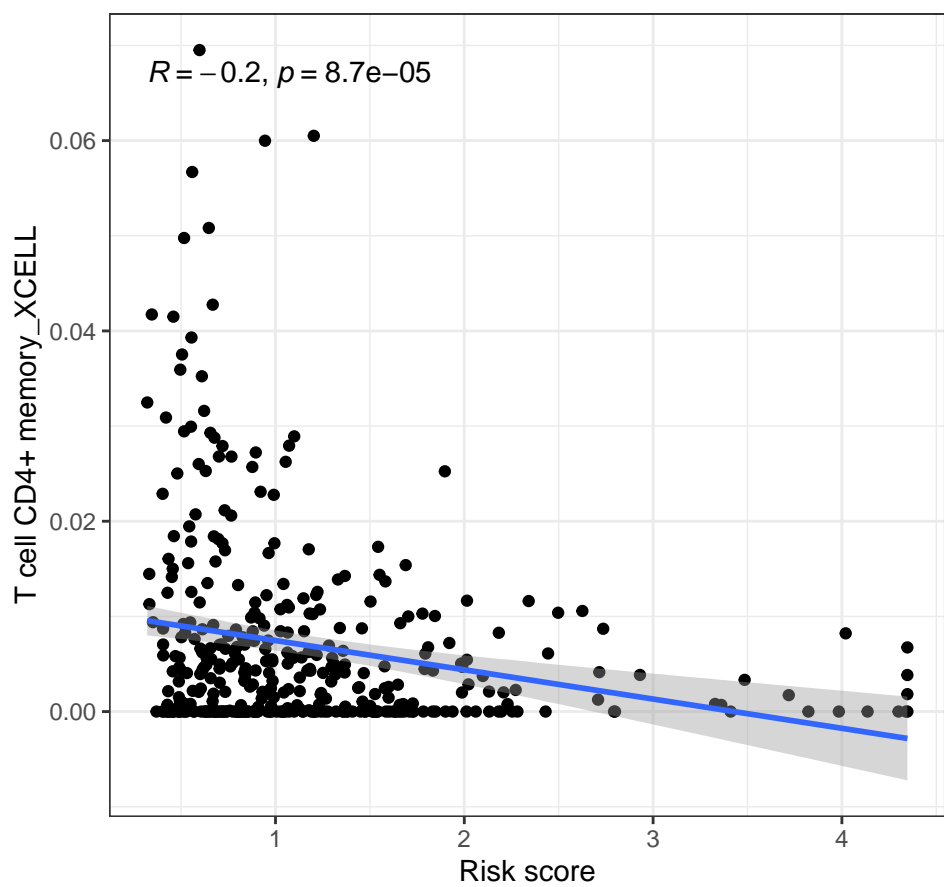

Supplement: Supplementary file 6 [file DataSheet_6.zip › cor.T cell CD4+ memory_XCELL.pdf]

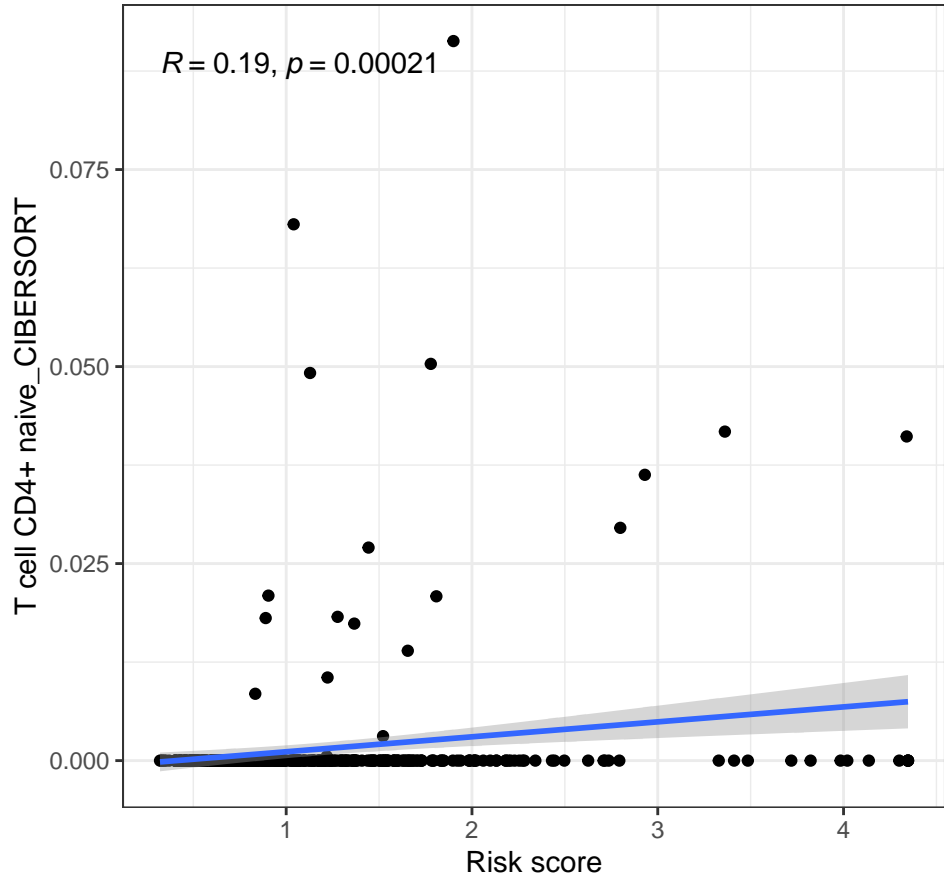

Supplement: Supplementary file 6 [file DataSheet_6.zip › cor.T cell CD4+ naive_CIBERSORT.pdf]

T cell CD4+ naive\_CIBERSORT-ABS

$R = 0.19, p = 0.00022$

0.010

0.005

0.000

1

2

3

4

Risk score

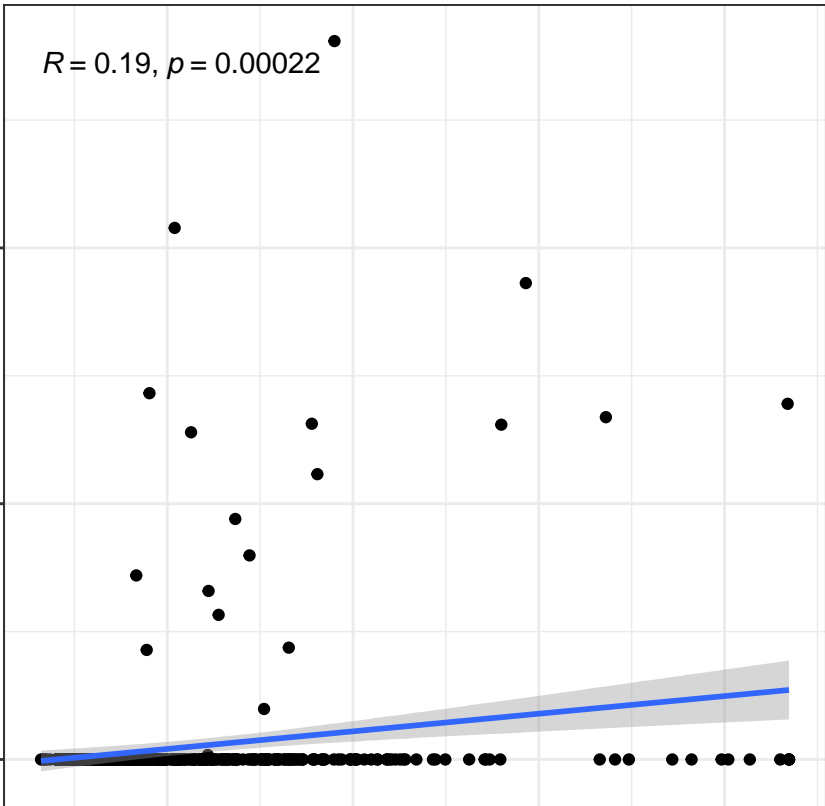

Supplement: Supplementary file 6 [file DataSheet_6.zip › cor.T cell CD4+ naive_CIBERSORT-ABS.pdf]

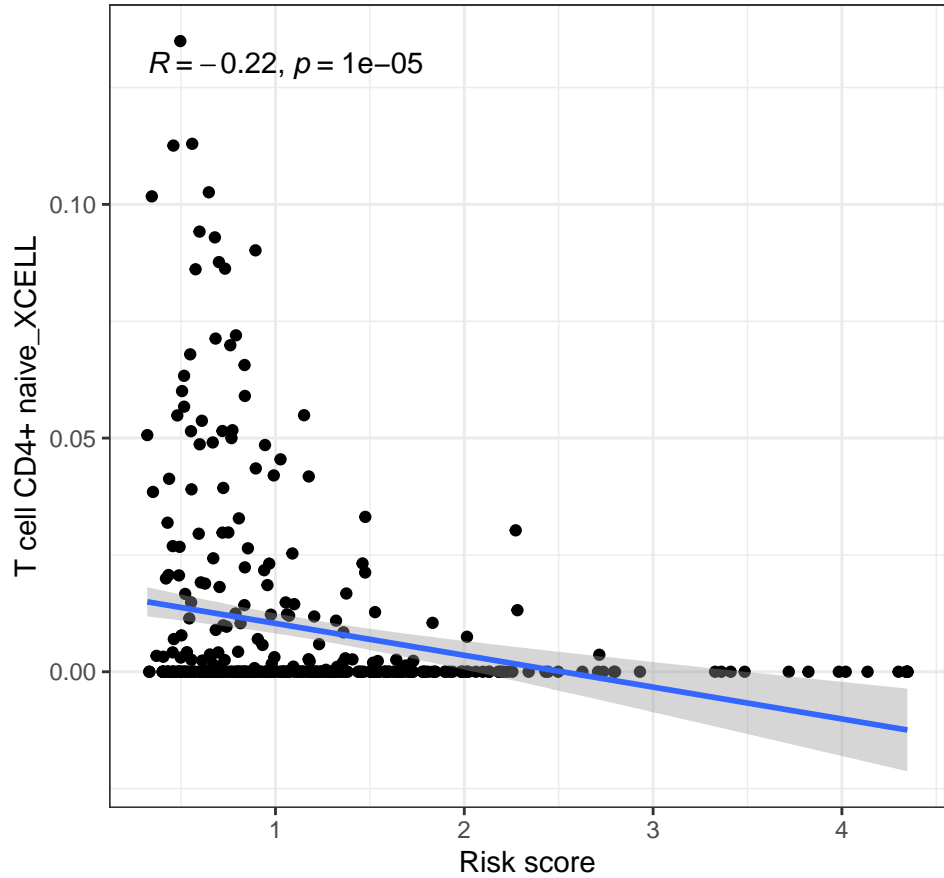

Supplement: Supplementary file 6 [file DataSheet_6.zip › cor.T cell CD4+ naive_XCELL.pdf]

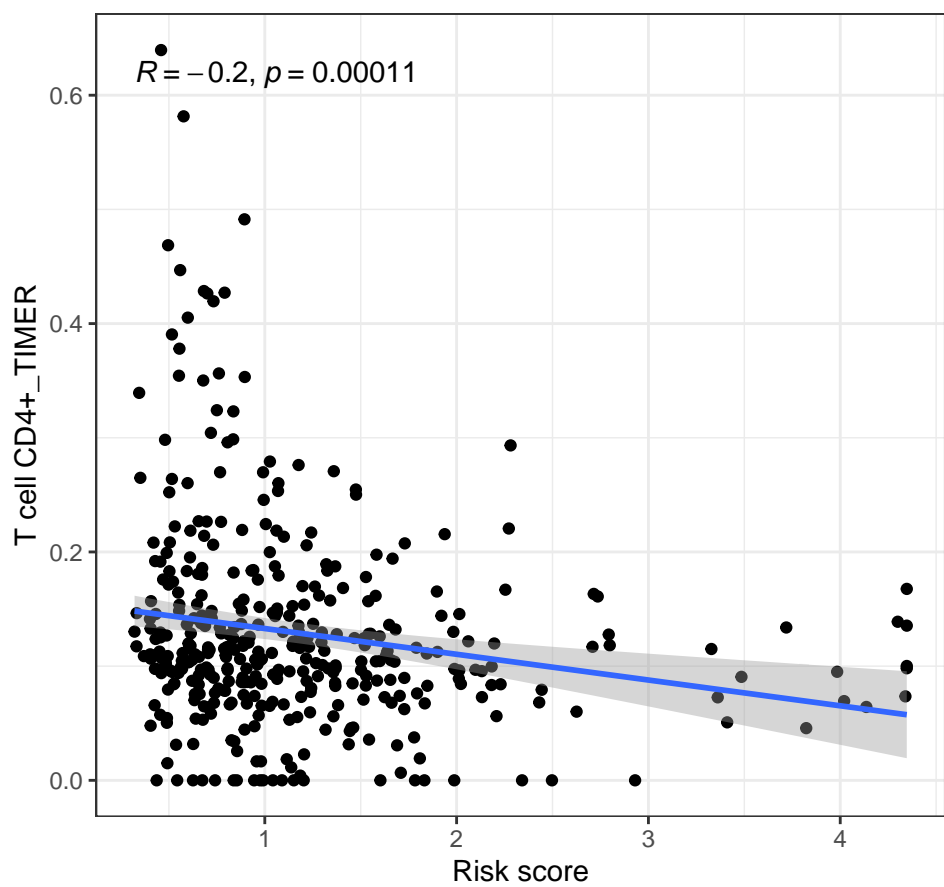

Supplement: Supplementary file 6 [file DataSheet_6.zip › cor.T cell CD4+_TIMER.pdf]

T cell CD8+ central memory\_XCELL

$R = -0.43, p < 2.2e-16$

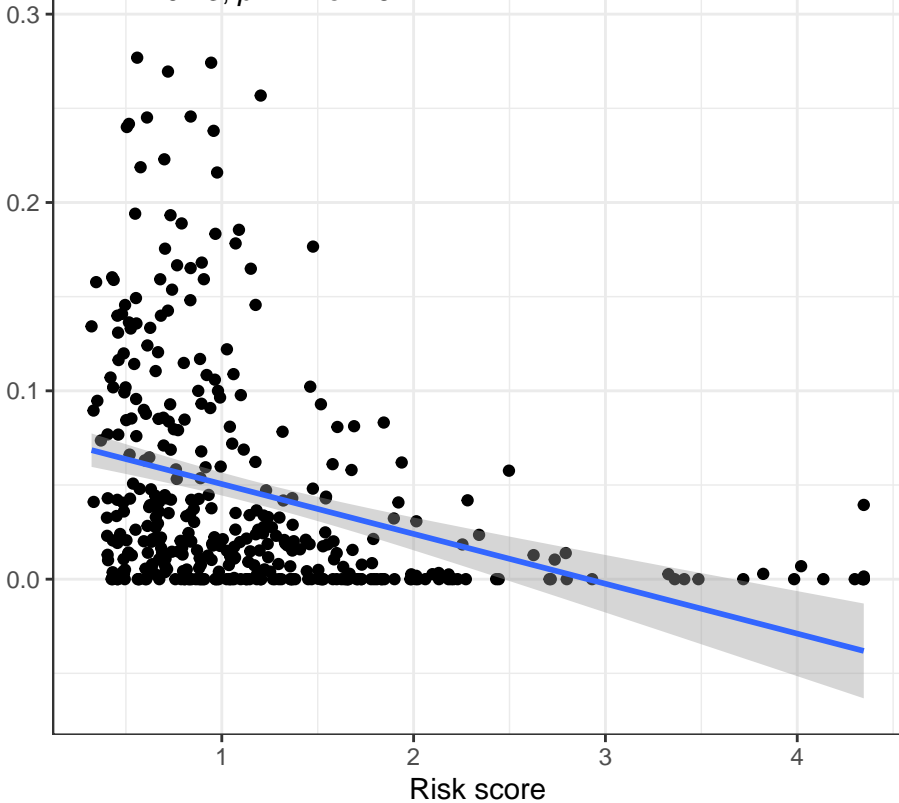

Supplement: Supplementary file 6 [file DataSheet_6.zip › cor.T cell CD8+ central memory_XCELL.pdf]

T cell CD8+ effector memory\_XCELL

$R = -0.25, p = 6.2e-07$

0.15

0.10

0.05

0.00

1

3

4

Risk score

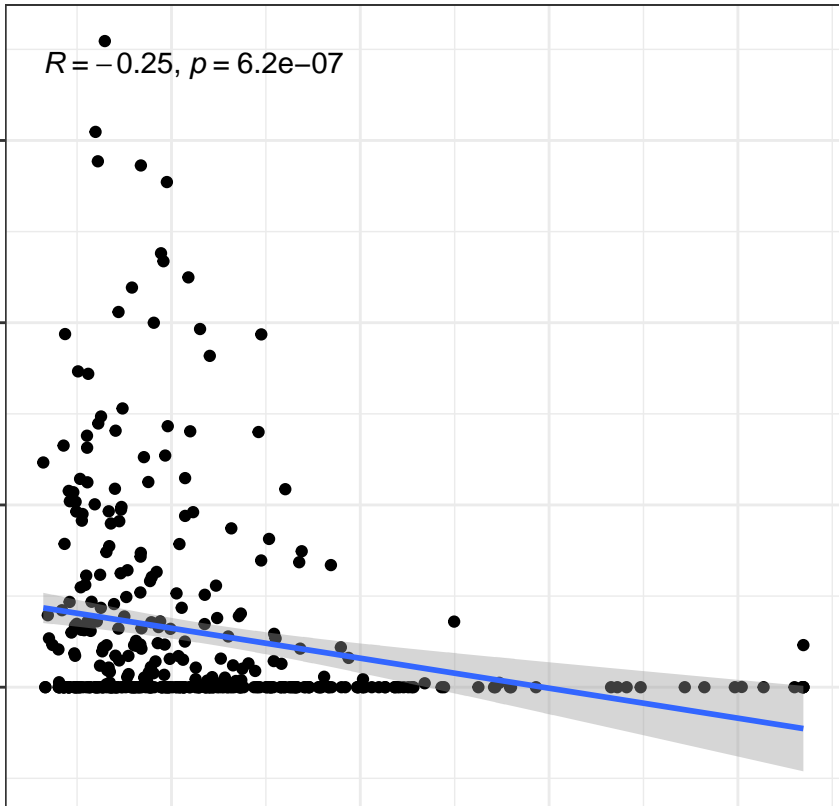

Supplement: Supplementary file 6 [file DataSheet_6.zip › cor.T cell CD8+ effector memory_XCELL.pdf]

T cell CD8+\_CIBERSORT

$R = -0.39, p = 1.7e-15$

0.4  
0.3  
0.2  
0.1  
0.0

Risk score

1

2

3

4

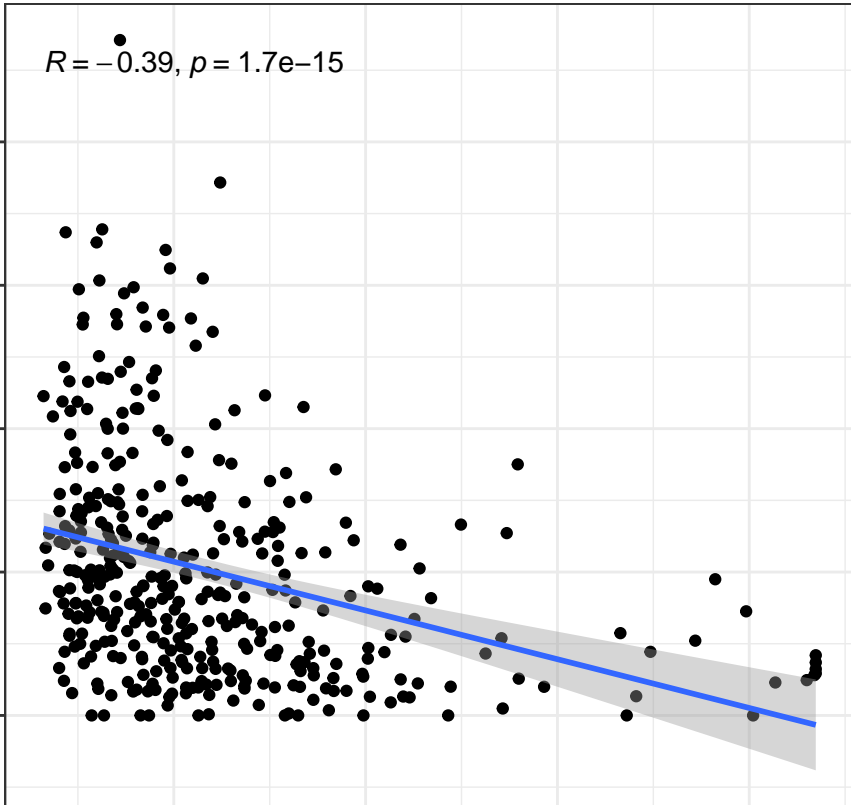

Supplement: Supplementary file 6 [file DataSheet_6.zip › cor.T cell CD8+_CIBERSORT.pdf]

T cell CD8+<sub>-</sub>CIBERSORT-ABS

$R = -0.41, p < 2.2e-16$

Risk score

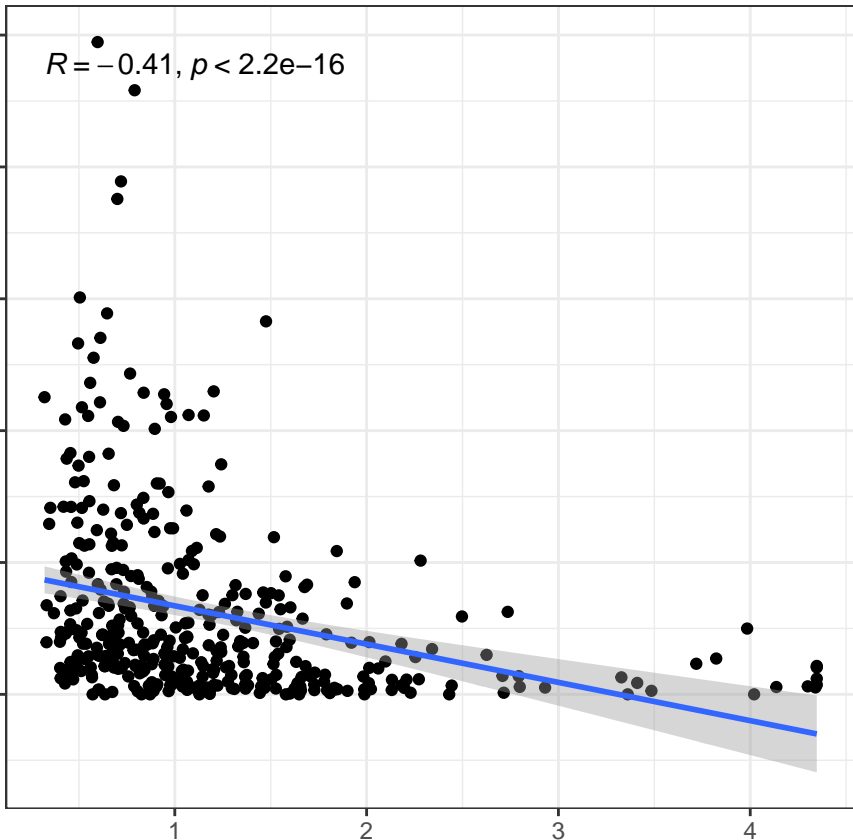

Supplement: Supplementary file 6 [file DataSheet_6.zip › cor.T cell CD8+_CIBERSORT-ABS.pdf]

T cell CD8+\_EPIC

$R = -0.35, p = 9.7\text{e-}13$

0.100  
0.075  
0.050  
0.025  
0.000

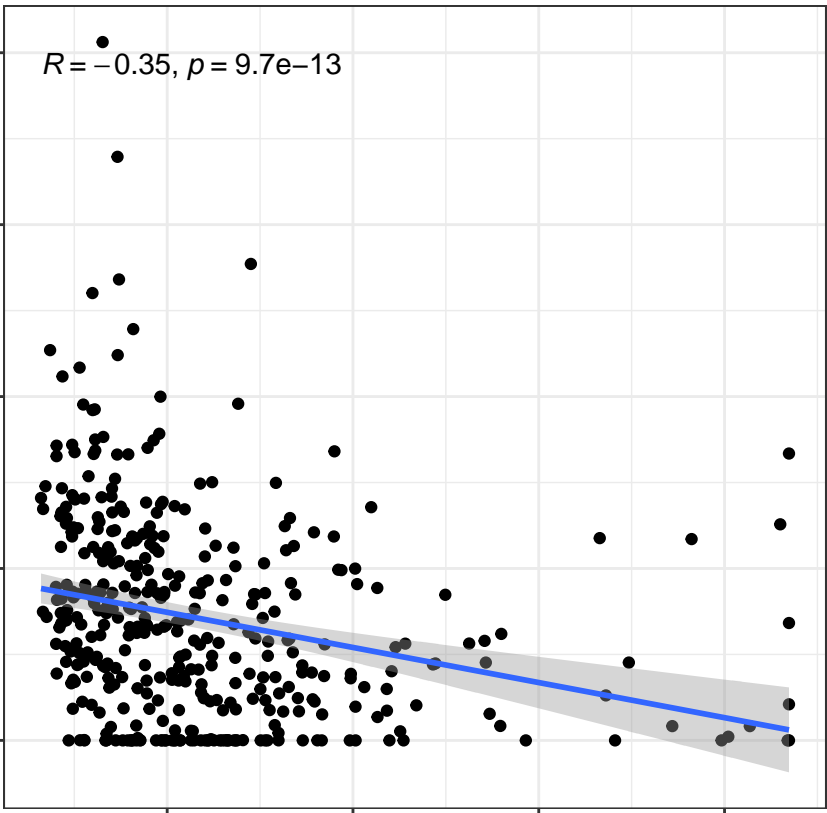

Risk score

Supplement: Supplementary file 6 [file DataSheet_6.zip › cor.T cell CD8+_EPIC.pdf]

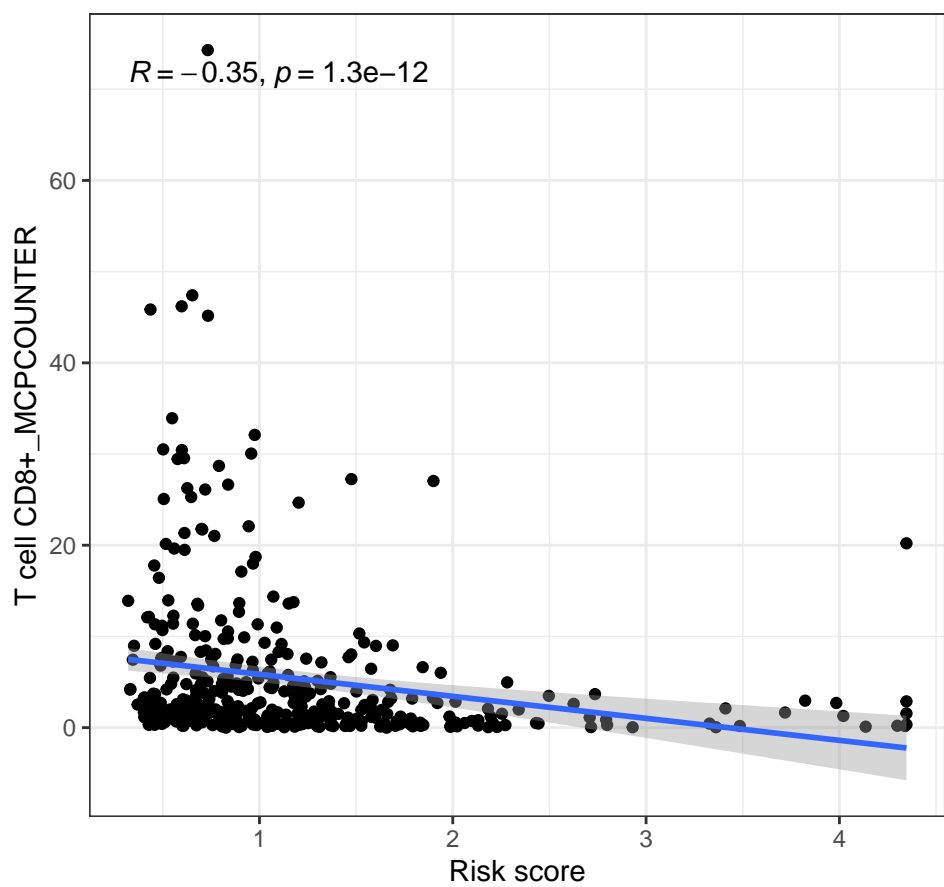

Supplement: Supplementary file 6 [file DataSheet_6.zip › cor.T cell CD8+_MCPCOUNTER.pdf]

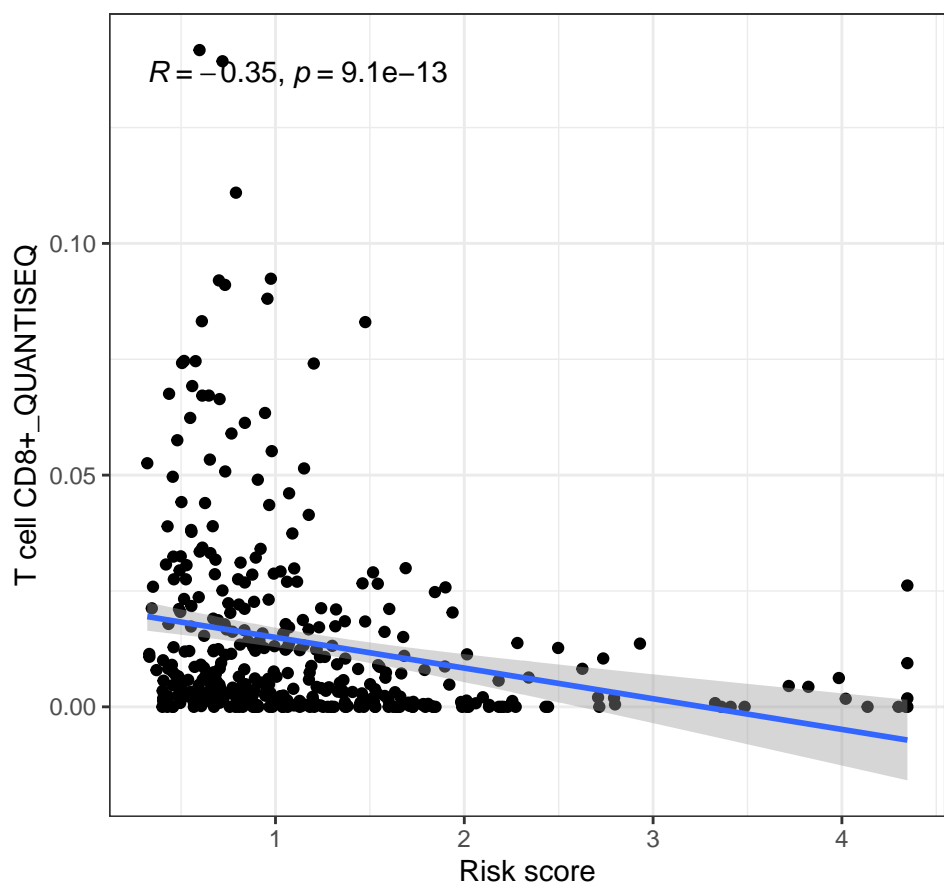

Supplement: Supplementary file 6 [file DataSheet_6.zip › cor.T cell CD8+_QUANTISEQ.pdf]

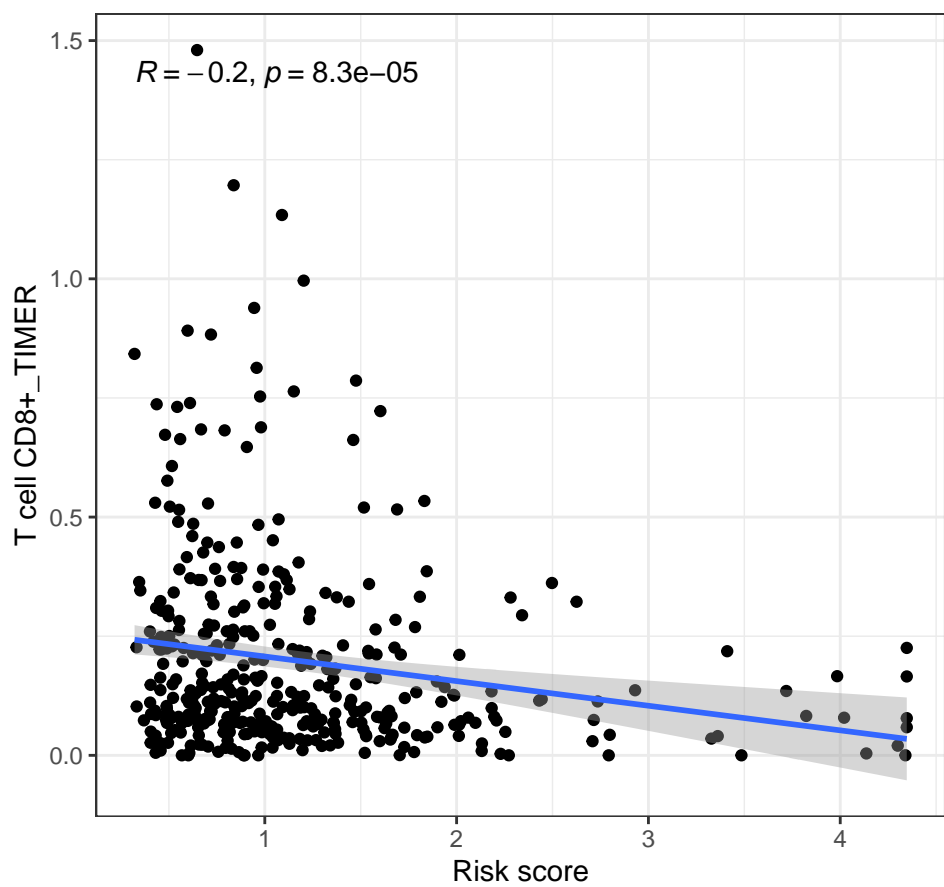

Supplement: Supplementary file 6 [file DataSheet_6.zip › cor.T cell CD8+_TIMER.pdf]

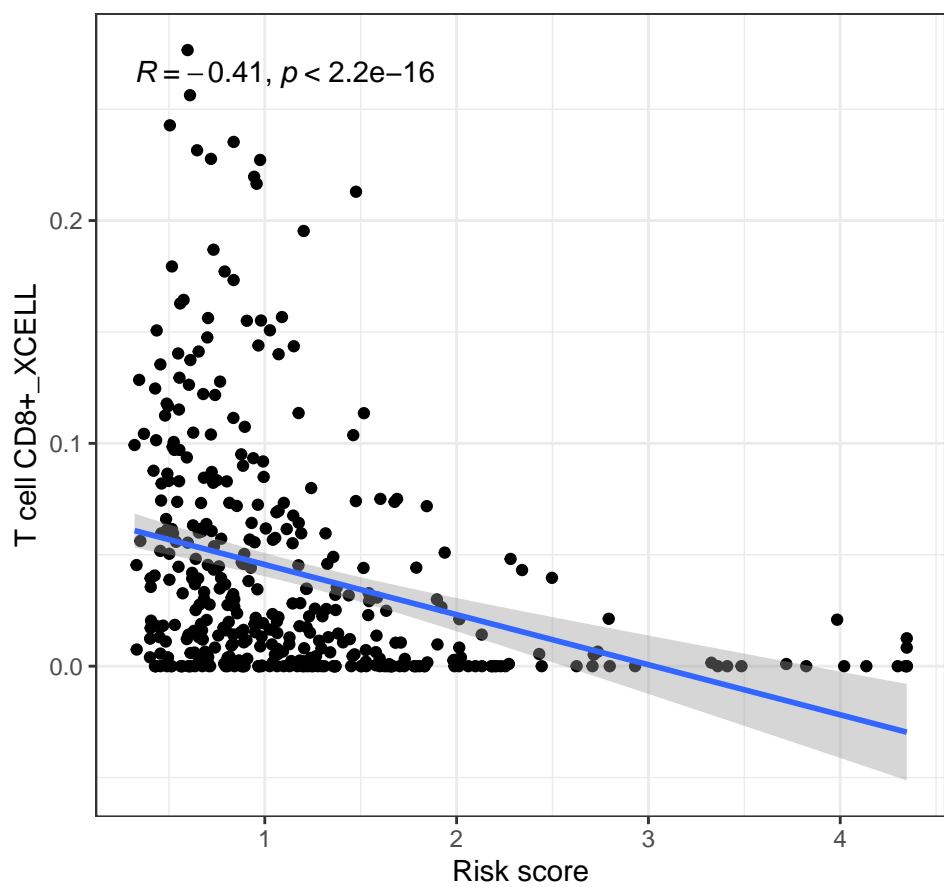

Supplement: Supplementary file 6 [file DataSheet_6.zip › cor.T cell CD8+_XCELL.pdf]

T cell follicular helper\_CIBERSORT

$R = -0.34, p = 1.1e-11$

0.20  
0.15  
0.10  
0.05  
0.00

Risk score

1

3

4

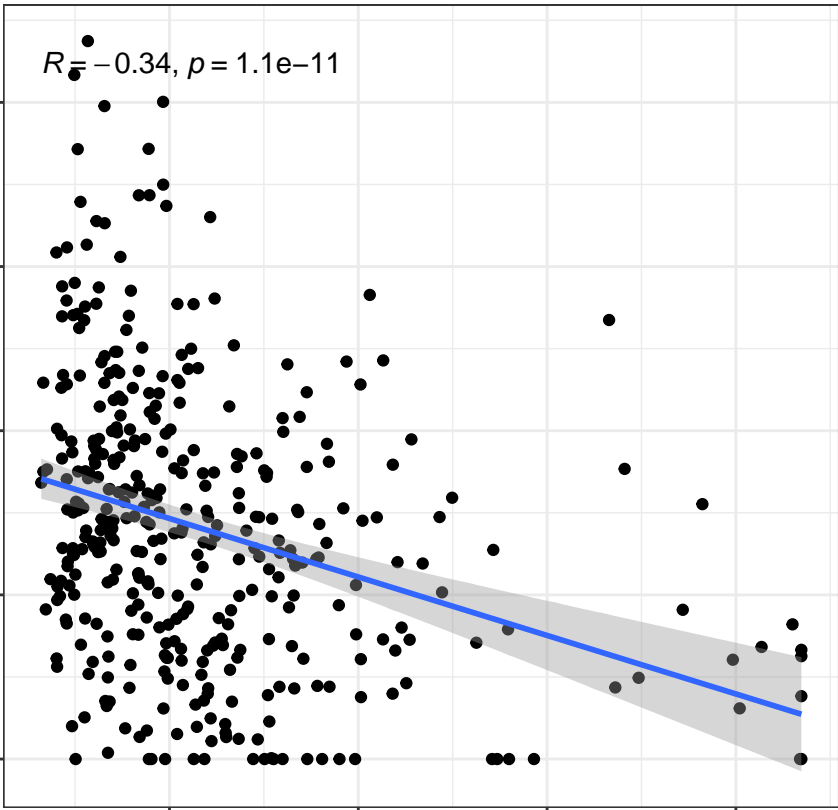

Supplement: Supplementary file 6 [file DataSheet_6.zip › cor.T cell follicular helper_CIBERSORT.pdf]

T cell follicular helper\_CIBERSORT-ABS

$R = -0.43, p < 2.2e-16$

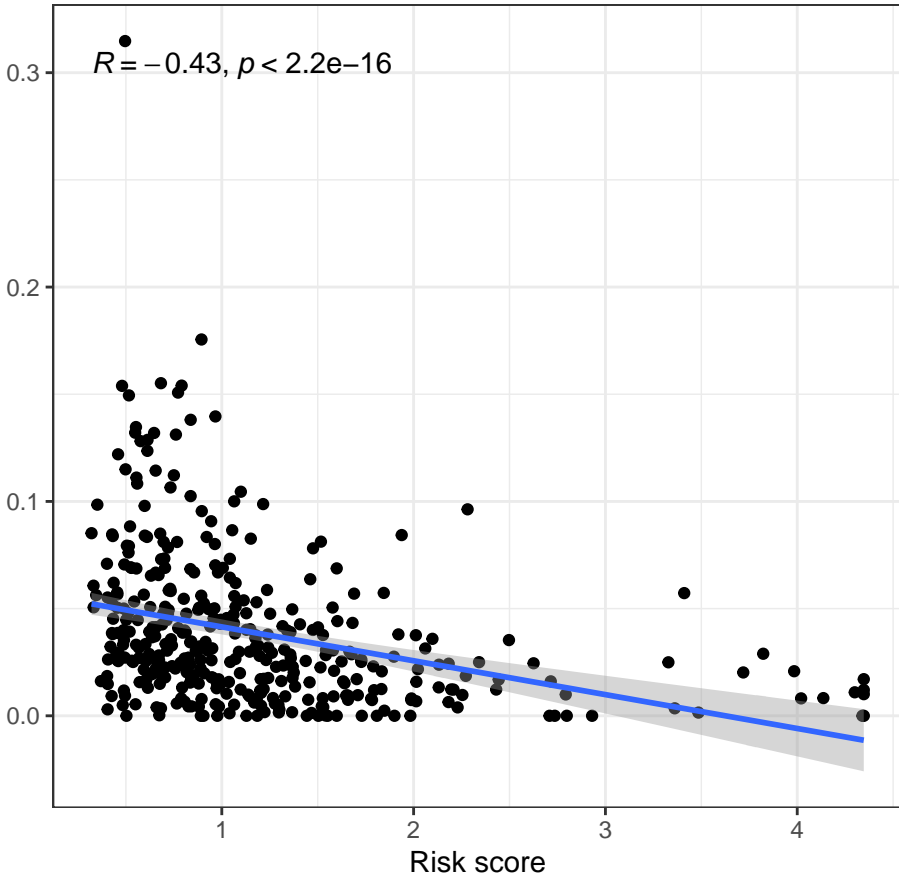

Supplement: Supplementary file 6 [file DataSheet_6.zip › cor.T cell follicular helper_CIBERSORT-ABS.pdf]

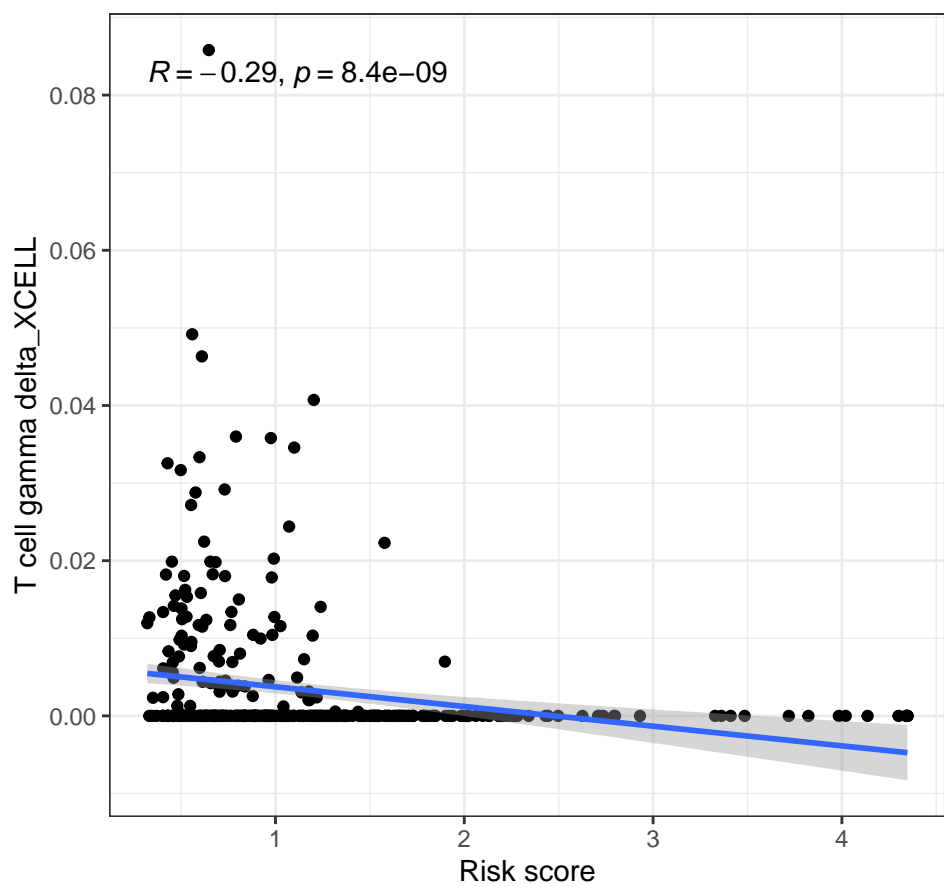

Supplement: Supplementary file 6 [file DataSheet_6.zip › cor.T cell gamma delta_XCELL.pdf]

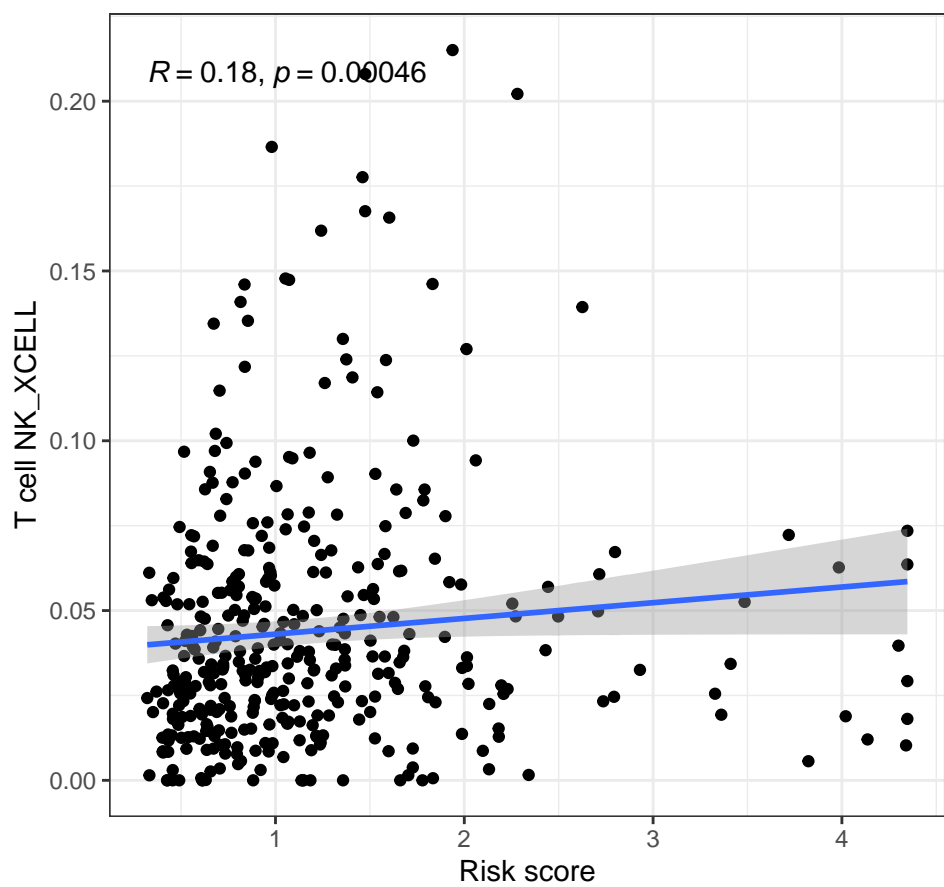

Supplement: Supplementary file 6 [file DataSheet_6.zip › cor.T cell NK_XCELL.pdf]

T cell regulatory (Tregs)\_CIBERSORT

$R = -0.13$ ,  $p = 0.012$

0.10

0.05

0.00

1

Risk score

3

4

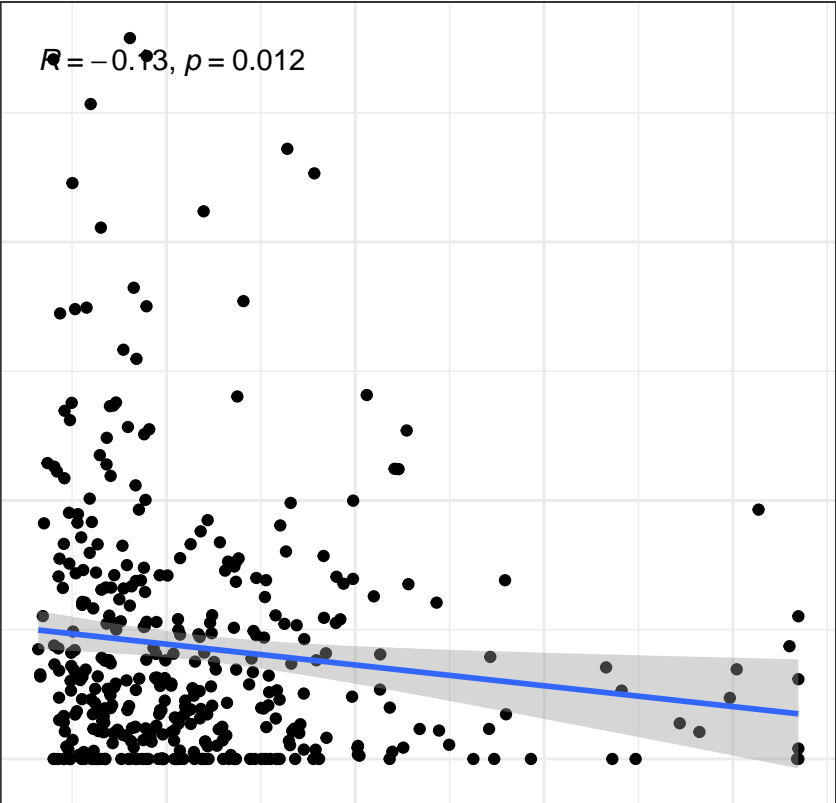

Supplement: Supplementary file 6 [file DataSheet_6.zip › cor.T cell regulatory (Tregs)_CIBERSORT.pdf]

T cell regulatory (Tregs)\_CIBERSORT-ABS

$R = -0.21$ ,  $p = 2.6e-05$

0.10

0.05

0.00

1

Risk score

3

4

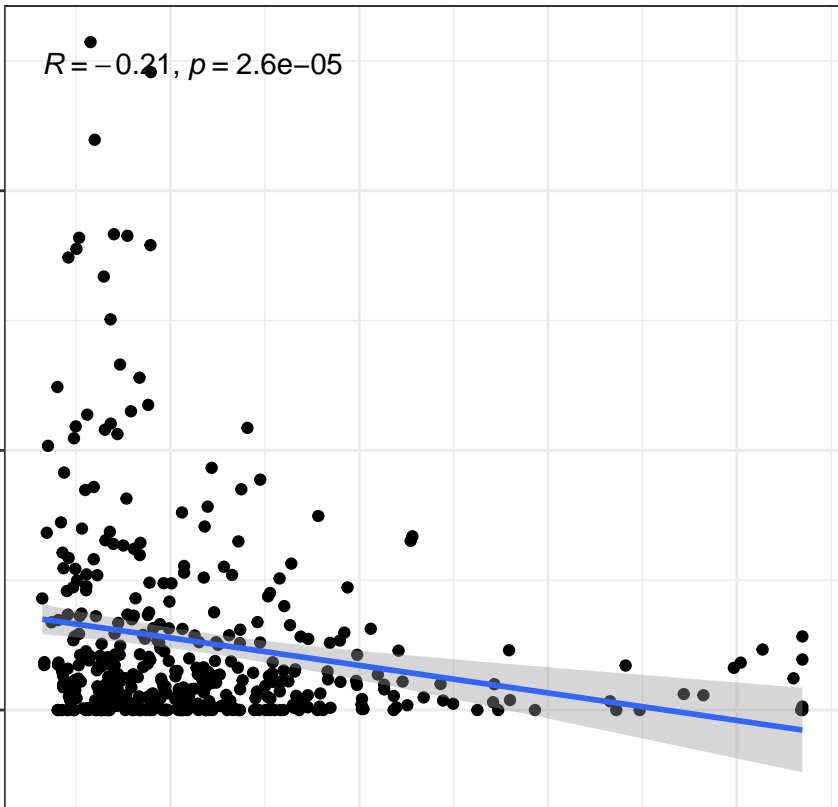

Supplement: Supplementary file 6 [file DataSheet_6.zip › cor.T cell regulatory (Tregs)_CIBERSORT-ABS.pdf]

T cell regulatory (Tregs)\_QUANTISEQ

$R = -0.26, p = 3.3e-07$

0.10

0.05

0.00

1

2

3

4

Risk score

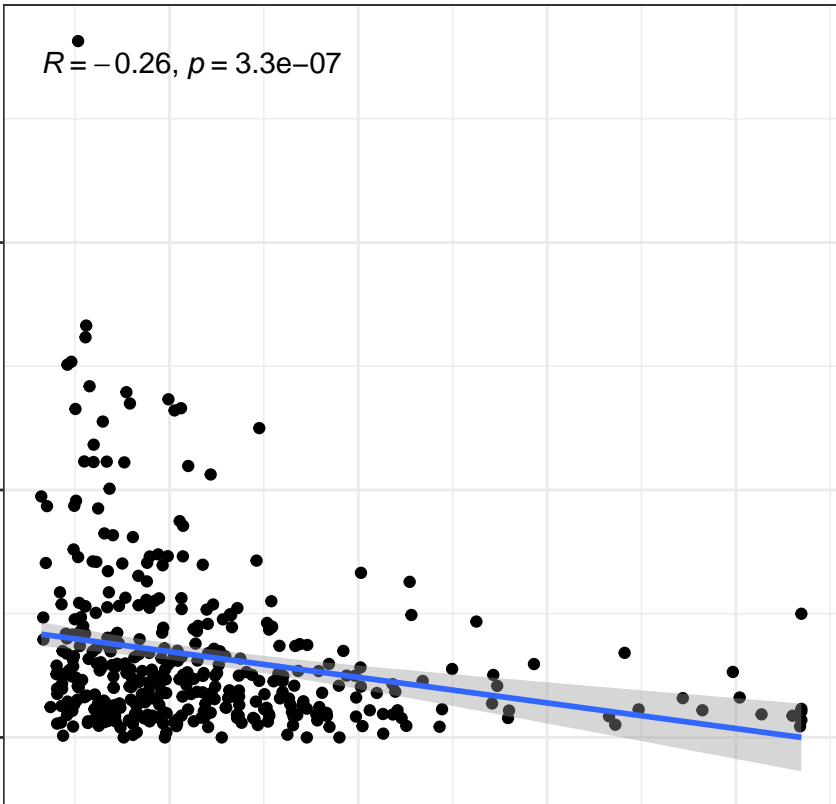

Supplement: Supplementary file 6 [file DataSheet_6.zip › cor.T cell regulatory (Tregs)_QUANTISEQ.pdf]

T cell\_MCPCOUNTER

$R = -0.36, p = 1.7e-13$

Risk score

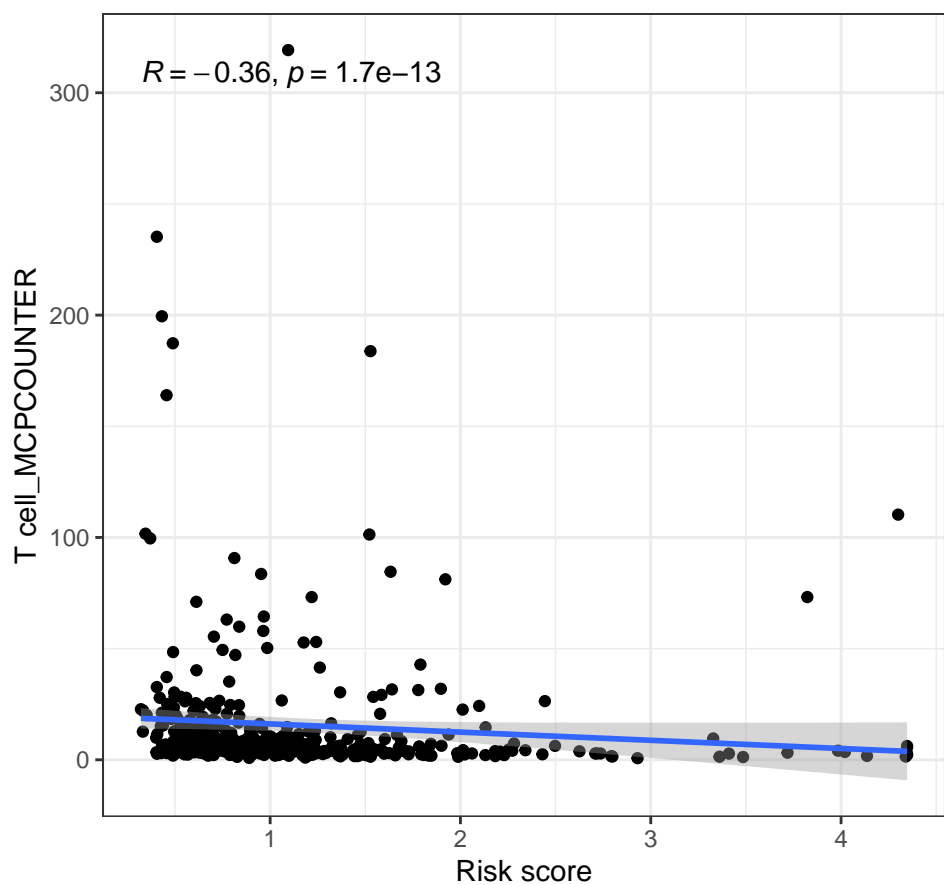

Supplement: Supplementary file 6 [file DataSheet_6.zip › cor.T cell_MCPCOUNTER.pdf]

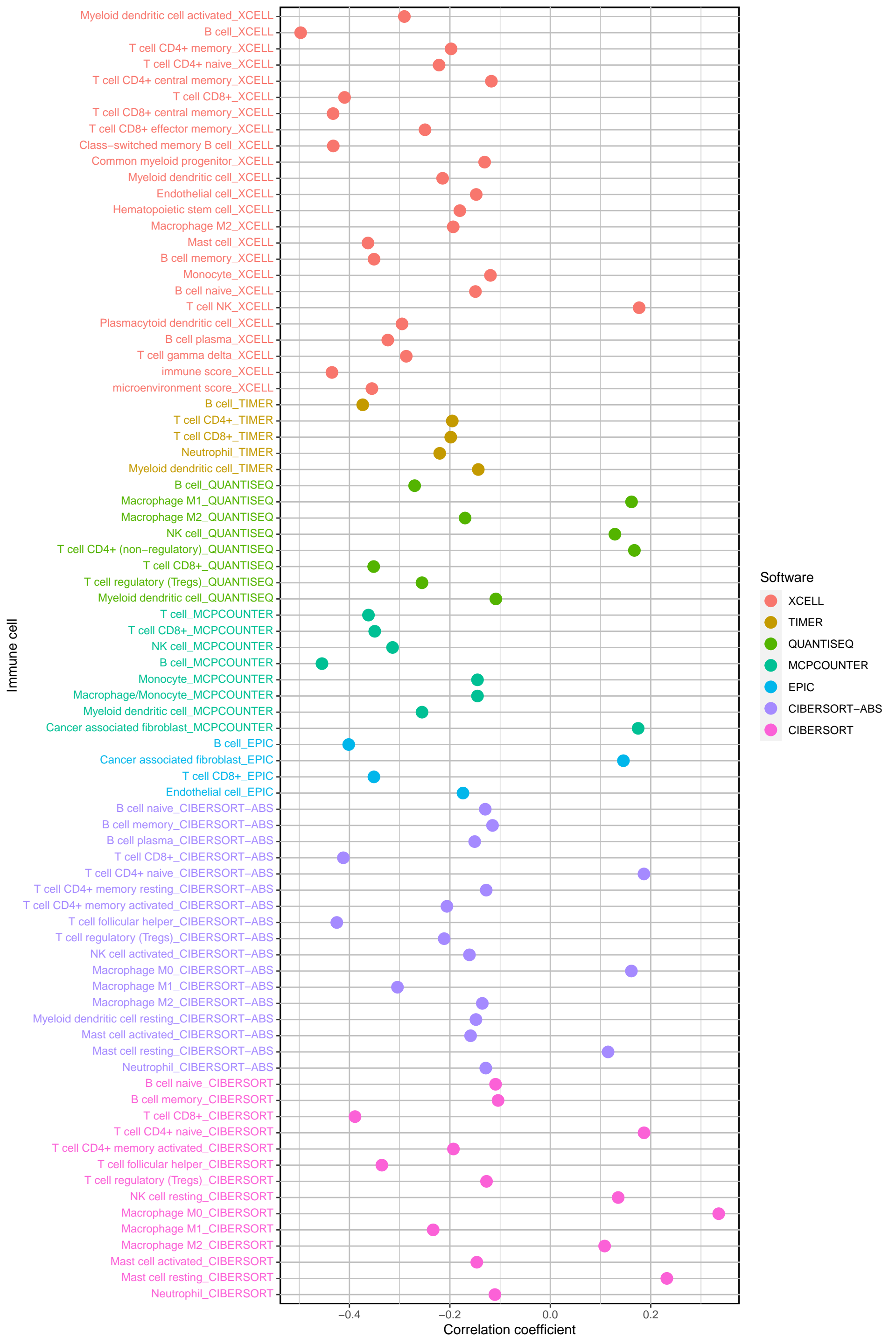

Supplement: Supplementary file 6 [file DataSheet_6.zip › correlation.pdf]

Percent weight

100  
75  
50  
25  
0

low

high

riskScore

66%

58%

34%

42%

Age

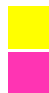

<65

>=65

Supplement: Supplementary file 7 [file DataSheet_7.zip › Age.barplot.pdf]

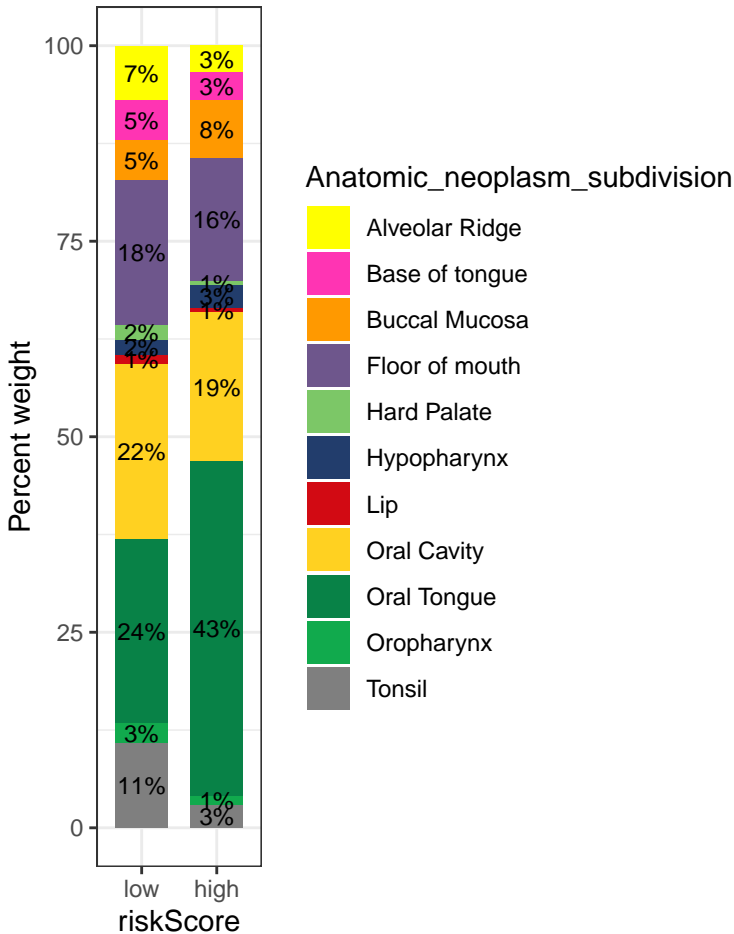

Supplement: Supplementary file 7 [file DataSheet_7.zip › Anatomic_neoplasm_subdivision.barplot.pdf]

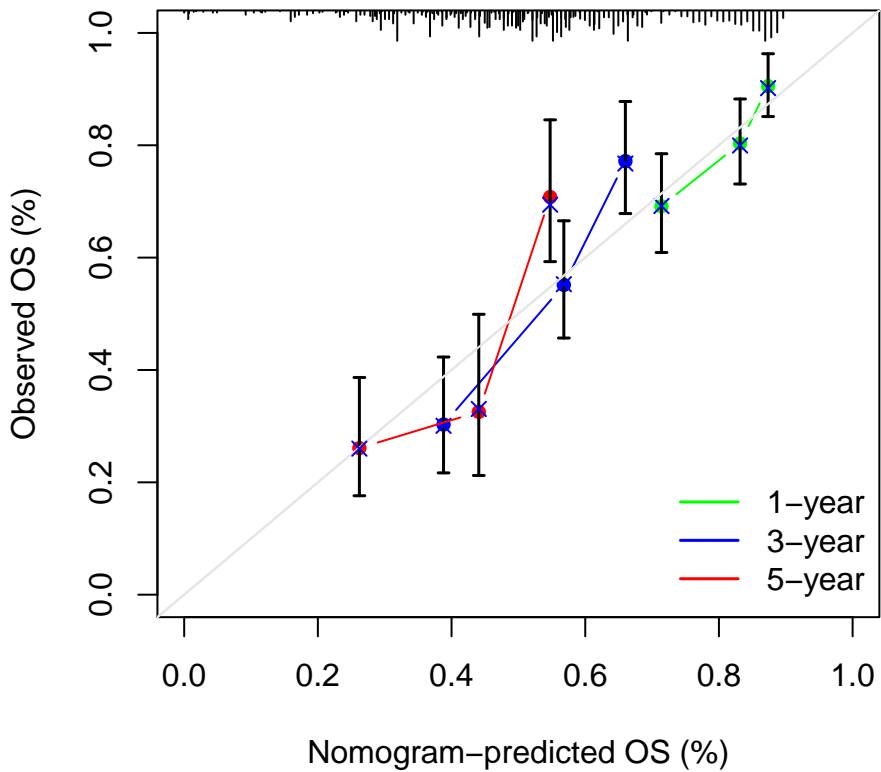

Supplement: Supplementary file 7 [file DataSheet_7.zip › calibration.pdf]

Percent weight

100  
75  
50  
25  
0

low

high

riskScore

32%

29%

68%

71%

Gender

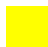

FEMALE

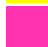

MALE

Supplement: Supplementary file 7 [file DataSheet_7.zip › Gender.barplot.pdf]

Percent weight

100  
75  
50  
25  
0

low

high

riskScore

Grade

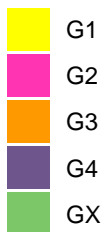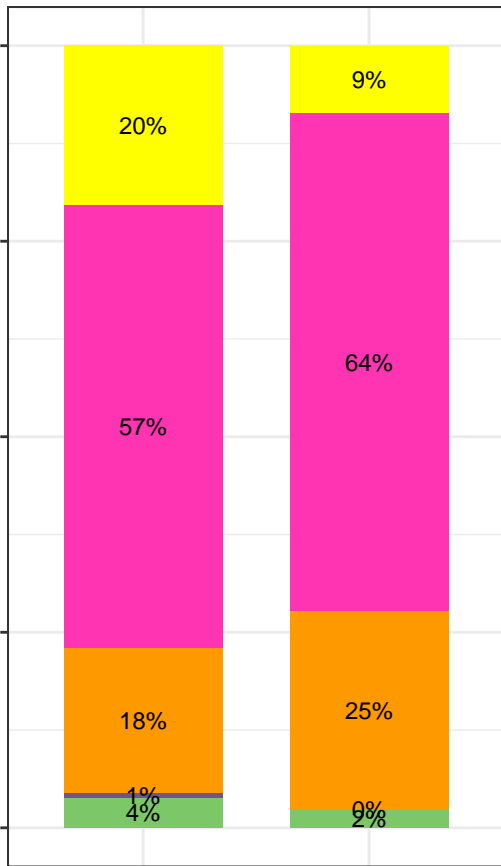

Supplement: Supplementary file 7 [file DataSheet_7.zip › Grade.barplot.pdf]

Percent weight

100  
75  
50  
25  
0

low

high

riskScore

94%

95%

1%  
4%

1%  
5%

M

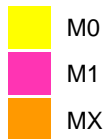

Supplement: Supplementary file 7 [file DataSheet_7.zip › M.barplot.pdf]

Percent weight

100  
75  
50  
25  
0

low

high

riskScore

N

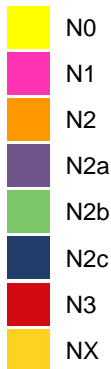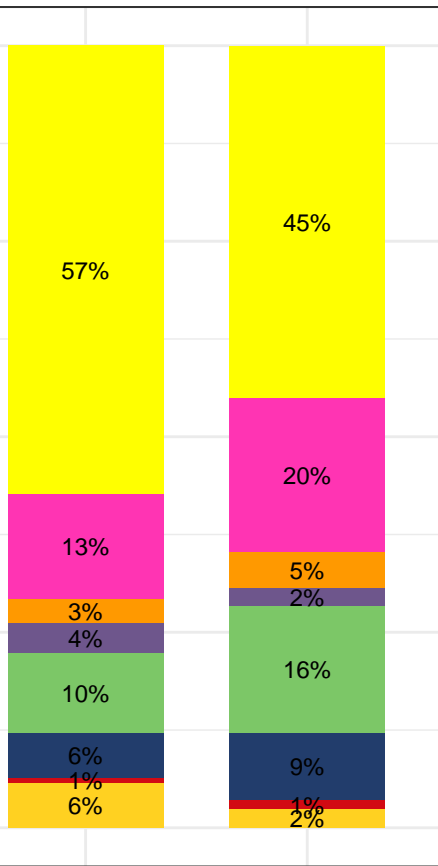

Supplement: Supplementary file 7 [file DataSheet_7.zip › N.barplot.pdf]

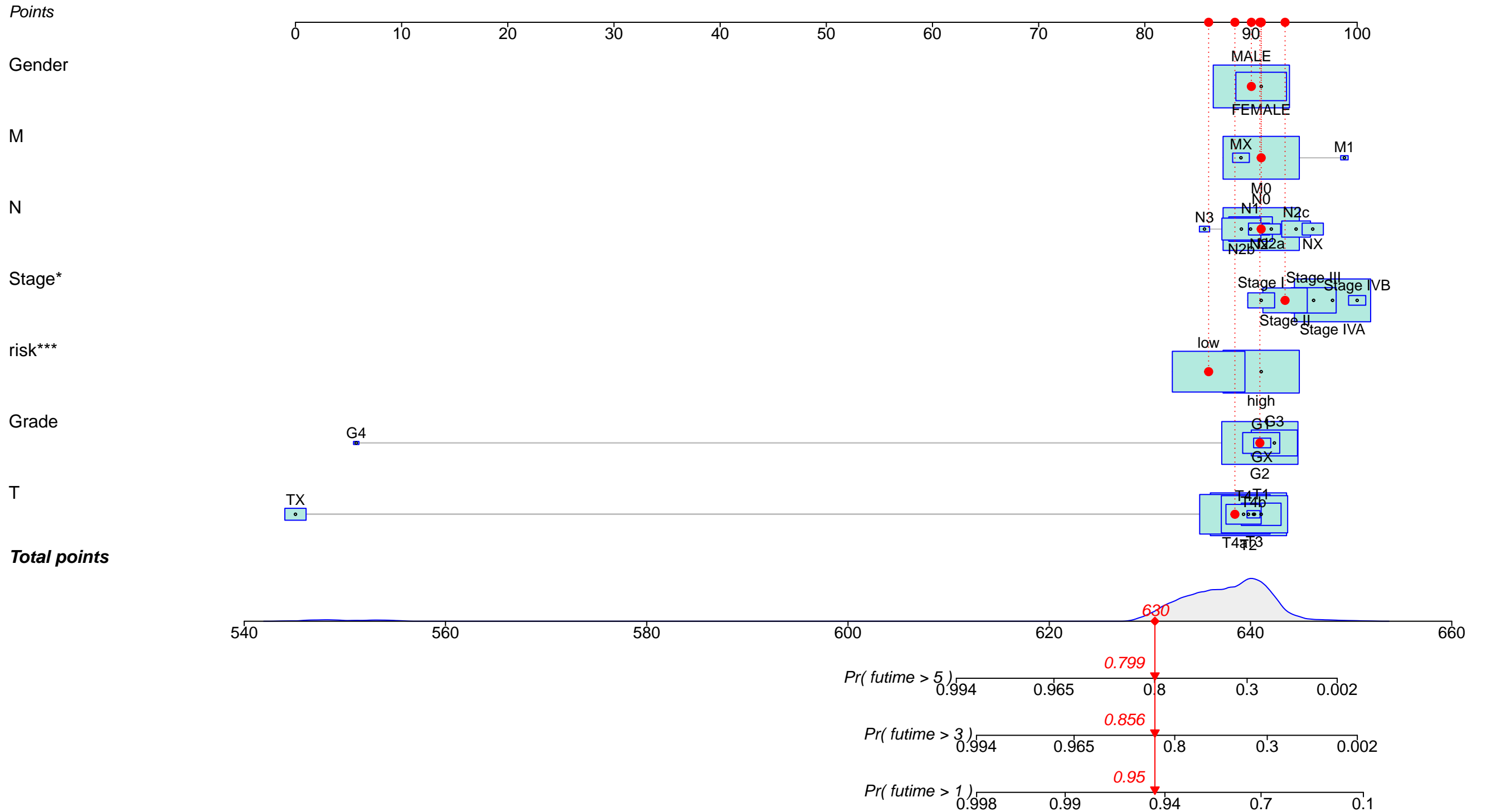

Supplement: Supplementary file 7 [file DataSheet_7.zip › Rplot01.pdf]

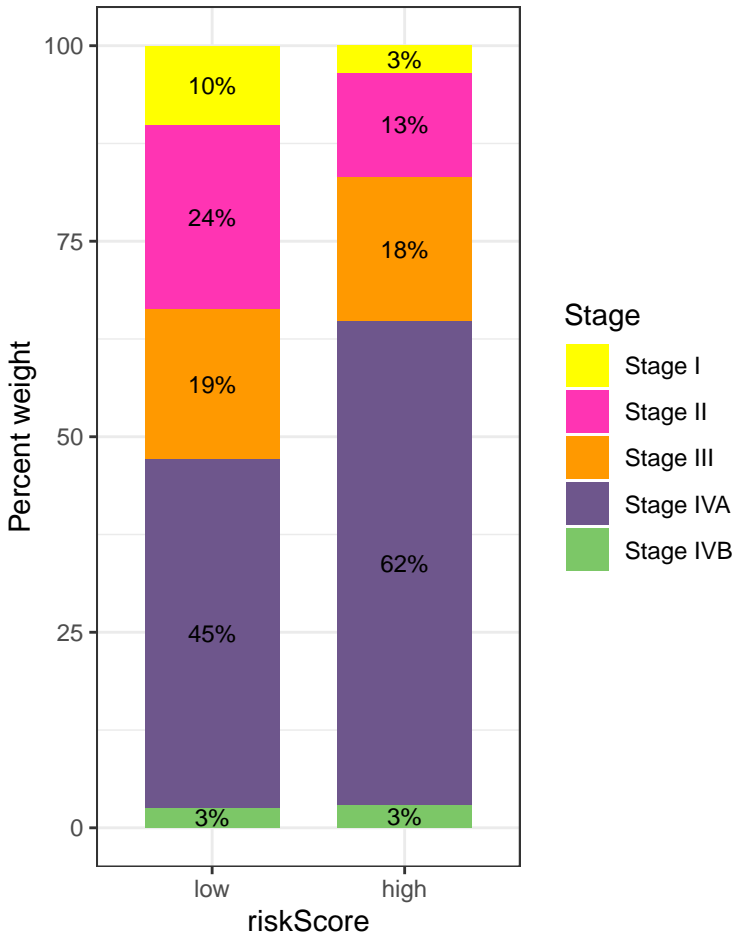

Supplement: Supplementary file 7 [file DataSheet_7.zip › Stage.barplot.pdf]

Percent weight

100  
75  
50  
25  
0

low

high

riskScore

T

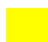

T1

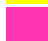

T2

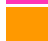

T3

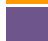

T4

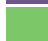

T4a

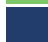

T4b

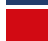

TX

13%

32%

22%

5%

25%

1%  
3%

5%

31%

25%

8%

28%

1%  
2%

Supplement: Supplementary file 7 [file DataSheet_7.zip › T.barplot.pdf]

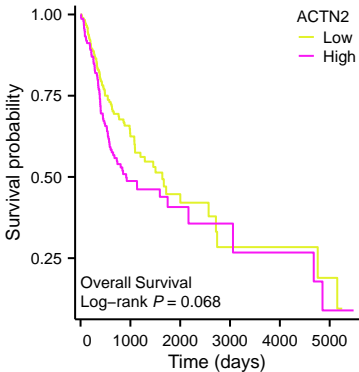

Supplement: Supplementary file 8 [file DataSheet_8.zip › ACTN2 1.pdf]

The expression of ACTN2  
 $\text{Log}_2(\text{TPM}+1)$

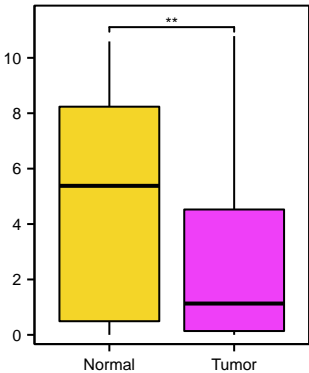

Supplement: Supplementary file 8 [file DataSheet_8.zip › ACTN2.pdf]

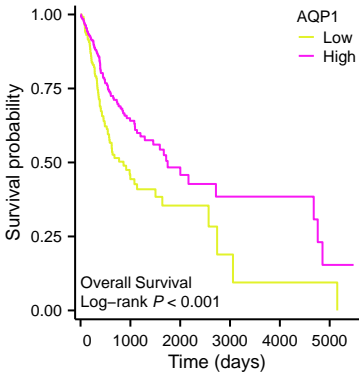

Supplement: Supplementary file 8 [file DataSheet_8.zip › AQP1 1.pdf]

The expression of AQP1  
 $\text{Log}_2(\text{TPM}+1)$

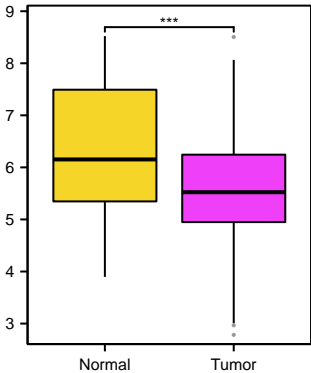

Supplement: Supplementary file 8 [file DataSheet_8.zip › AQP1.pdf]

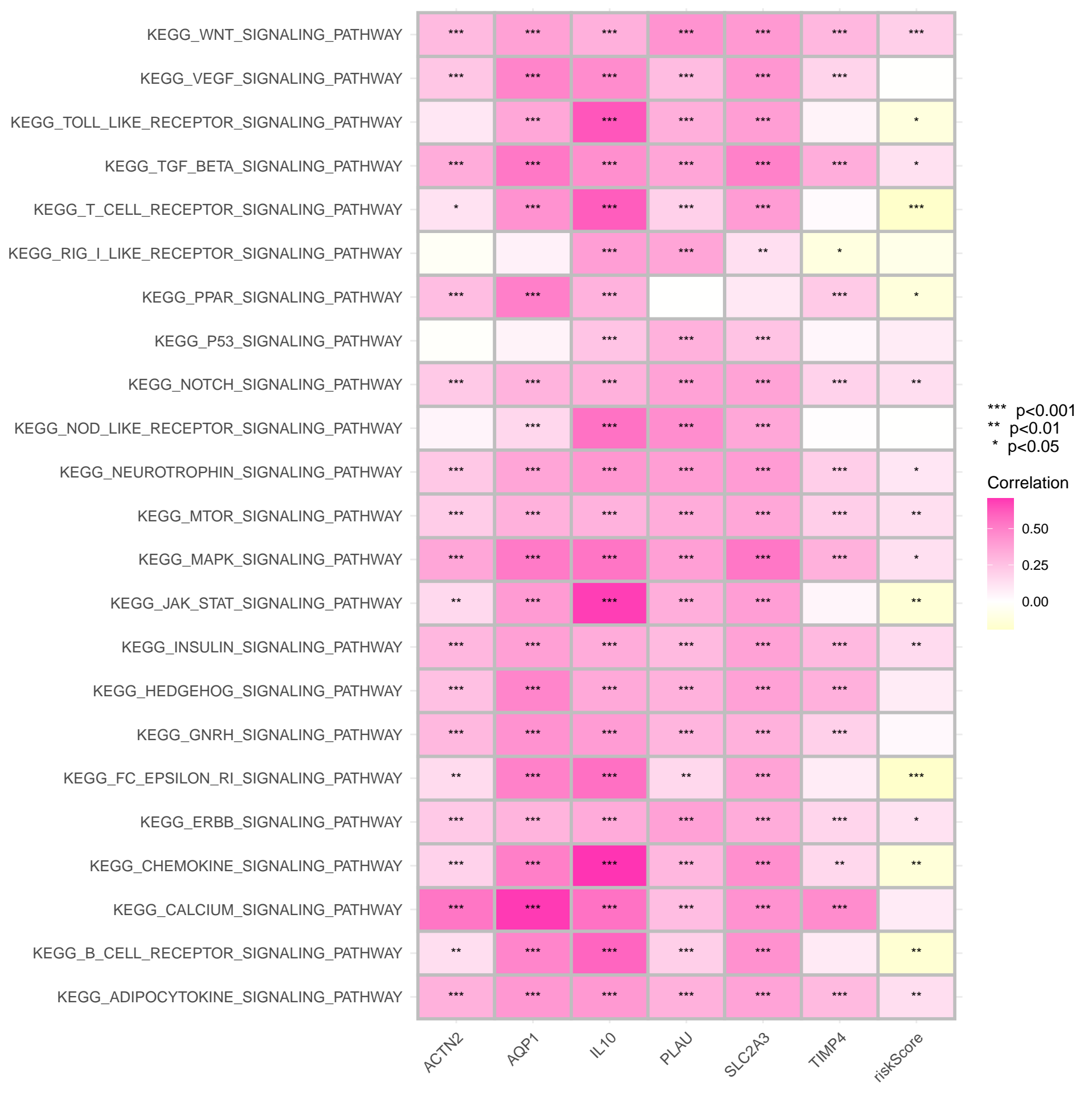

Supplement: Supplementary file 8 [file DataSheet_8.zip › GSVAcor.pdf]

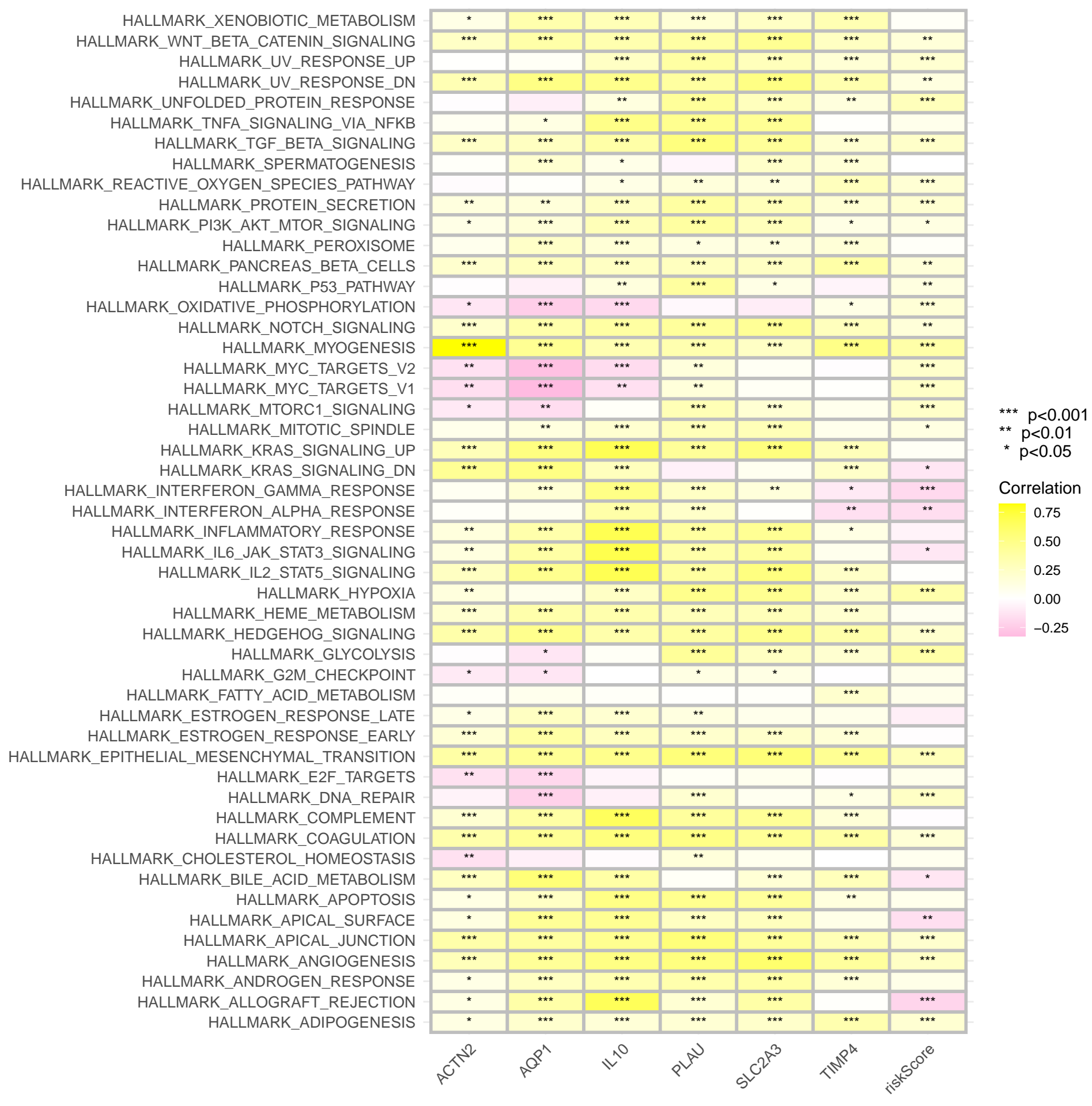

Supplement: Supplementary file 8 [file DataSheet_8.zip › Hallmark.pdf]

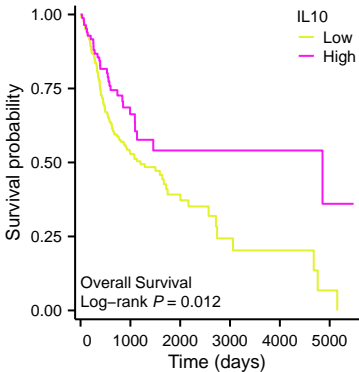

Supplement: Supplementary file 8 [file DataSheet_8.zip › IL10 1.pdf]

The expression of IL10  
 $\text{Log}_2(\text{TPM}+1)$

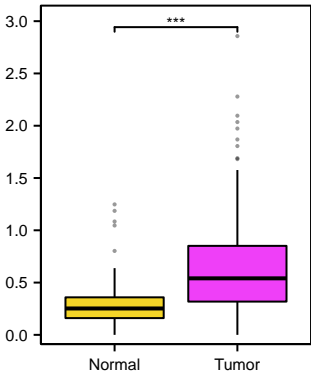

Supplement: Supplementary file 8 [file DataSheet_8.zip › IL10.pdf]

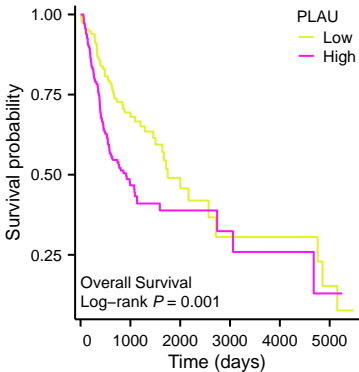

Supplement: Supplementary file 8 [file DataSheet_8.zip › PLAU 1.pdf]

The expression of PLAU  
 $\text{Log}_2(\text{TPM}+1)$

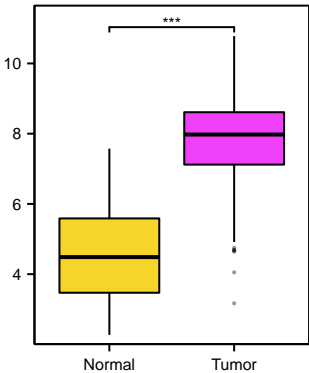

Supplement: Supplementary file 8 [file DataSheet_8.zip › PLAU.pdf]

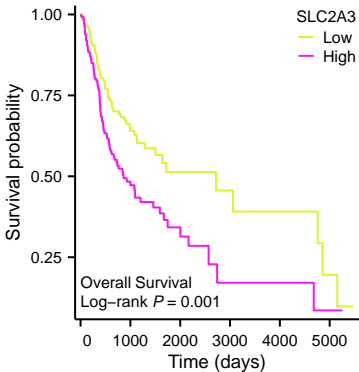

Supplement: Supplementary file 8 [file DataSheet_8.zip › SLC2A3 1.pdf]

The expression of SLC2A3  
 $\text{Log}_2(\text{TPM}+1)$

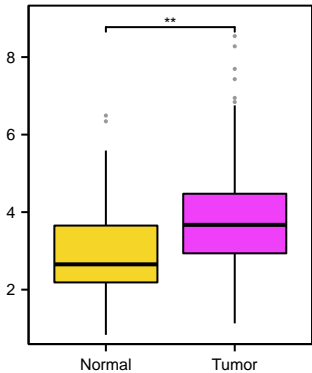

Supplement: Supplementary file 8 [file DataSheet_8.zip › SLC2A3.pdf]

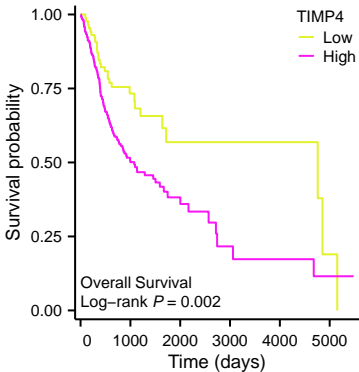

Supplement: Supplementary file 8 [file DataSheet_8.zip › TIMP4 1.pdf]

The expression of TIMP4  
 $\text{Log}_2(\text{TPM}+1)$

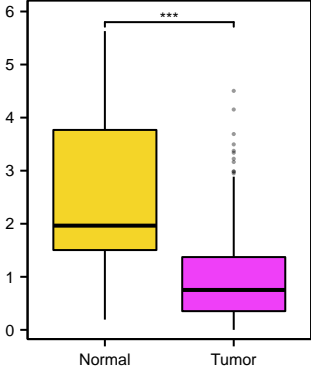

Supplement: Supplementary file 8 [file DataSheet_8.zip › TIMP4.pdf]
